# The evolution of reversible plasticity in stable environments

**DOI:** 10.1093/evlett/qraf015

**Published:** 2025-07-08

**Authors:** Nicole Walasek, Karthik Panchanathan, Willem E Frankenhuis

**Affiliations:** Evolutionary and Population Biology, Institute for Biodiversity and Ecosystem Dynamics, University of Amsterdam, Amsterdam, The Netherlands; Department of Anthropology, University of Missouri, Columbia, Missouri, United States; Evolutionary and Population Biology, Institute for Biodiversity and Ecosystem Dynamics, University of Amsterdam, Amsterdam, The Netherlands; Max Planck Institute for the Study of Crime, Security and Law, Freiburg, Germany

**Keywords:** phenotypic plasticity, reversible plasticity, stable environments, evolution, development

## Abstract

Reversible plasticity, i.e., the ability to deconstruct phenotypic specializations based on environmental conditions, is widespread in nature. Despite its ubiquity, few mathematical models have explored the evolutionary selection pressures that favor trait reversibility. Therefore, many scenarios remain to be examined. In particular, existing theory has modeled trait development as an instantaneous process. These models do not capture the fact that trait development is often a constructive process, in which phenotypes incrementally adjust to local ecologies. Here, we present an optimality model of the evolution of reversible plasticity in which organisms build traits incrementally. In our model, organisms repeatedly sample cues to infer the environmental state—which can vary between generations but not within generations—and incrementally tailor their phenotypes to match their environments. Organisms also have the option to deconstruct phenotypic adjustments. We investigate two different modes of phenotypic deconstruction: Organisms can either deconstruct phenotypic adjustments incrementally or completely deconstruct all phenotypic adjustments in one time period. We highlight two results. First, early-life sensitive periods in construction precede mid-ontogeny sensitive periods in deconstruction. Intriguingly, although organisms typically only deconstruct toward the end of ontogeny, environmental cues in mid-ontogeny have the strongest impact on deconstruction. Second, in contrast to previous models, we find that reversibility often evolves in environments that are stable within generations. Thus, our model shows that reversibility does not require environmental change during development—as long as organisms are initially uncertain about environmental conditions. Our model provides new insights into the capacity for reversibility in species that have evolved in ontogenetically stable environments.

## Introduction

The ability to deconstruct phenotypic specializations, a process called *reversible phenotypic plasticity*, is widespread (Nettle & Bateson, 2015; Stearns, 1989; West-Eberhard, 2003). Reversible phenotypic plasticity has been modeled as a lifelong capacity to completely reverse phenotypes (Burggren, 2020; Piersma & Drent, 2003). Not all empirical examples of reversibility work in this way. The freshwater snail *Helisoma trivolis* can develop shell thickness to guard against predators, but can also reverse course and reduce thickness if conditions change (Hoverman & Relyea, 2007). However, the complete reversal of shell thickening only occurs if predators are removed early in ontogeny. In the water flea *Daphnia barbata*, reversibility of defensive morphologies is specific to predator and trait. *Daphnia* exposed to tadpoles (*Triops cancriformis*)—but not to an aquatic insect predator (*Notonecta glauca*)—can deconstruct defensive morphologies (Herzog et al., 2016). And, reversibility is higher for tail-spine shape compared to helmet shape. These examples raise intriguing questions about variation in reversibility: Why are some traits completely reversible, while others are not? Why do some organisms retain reversibility across the lifespan, while in others, reversibility is limited to specific developmental windows?

These questions have not received sufficient theoretical investigation (Piersma & Drent, 2003). Existing models focus on understanding the conditions that favor phenotypic reversibility over irreversibility (Botero et al., 2015; Gabriel, 2005; Gabriel et al., 2005; Pfab et al., 2016; Ratikainen & Kokko, 2019; Utz et al., 2014). These models suggest that reversible plasticity evolves when environmental conditions change across an organism’s life. Stable environments disfavor reversibility as the benefits rarely outweigh the costs. Notably, all but one of these models assume that construction and deconstruction are instantaneous, such that organisms can immediately attain the optimal phenotype for a specific environmental state. The exception is a model that allows for both incremental phenotypic construction and deconstruction (Pfab et al., 2016). Here, the slower pace of incremental deconstruction limits the benefits of reversibility: Reversibility only outperforms irreversibility if organisms can match their current environment before it changes again.

A small number of models have explored how reversible plasticity changes across ontogeny (Fischer et al., 2014; Stamps & Krishnan, 2014, 2017). These models assume that organisms can completely and instantaneously reverse development. They study when during ontogeny environmental cues have the largest impact on phenotypic changes, resulting in sensitive periods. These models observe the highest levels of bidirectional plasticity at the onset of ontogeny, regardless of whether the environment is stable (Stamps & Krishnan, 2014, 2017) or not (Fischer et al., 2014).

The existing theoretical framework—centered on instantaneous trait development—fails to account for the full spectrum of reversible plasticity observed in nature. It remains an open question how incremental development shapes sensitive periods in phenotype construction and deconstruction. Or, when we should expect complete versus partial reversibility. Here, we address these questions, drawing on an optimality modeling framework we developed to study the evolution and development of sensitive periods (Frankenhuis & Panchanathan, 2011a; Panchanathan & Frankenhuis, 2016; Walasek et al., 2022a, 2022b). Our modeling framework assumes incremental phenotypic specializations. In this paper, we extend our framework by allowing organisms to deconstruct previous phenotypic increments. We consider two modes of deconstruction: Organisms can either incrementally undo phenotypic increments or completely undo them in one time period. Our model is the first to simultaneously consider sensitive periods for phenotype construction and deconstruction. Further contrasting previous work, we assume that the environment remains stable across an organism’s lifetime, but that organisms cannot directly perceive the state. Instead, they sample cues to learn about their environment. Through this setup, we explore whether and when during ontogeny reversible plasticity evolves in a stable environment.

### Methods: environment and organism

The ecology consists of an infinite number of discrete, nonoverlapping patches. Each patch is characterized by one of two environmental states, ${{E}_0}$ (e.g., dangerous) or ${{E}_1}$ (e.g., safe). After birth, organisms randomly disperse into patches, the state of which is stable across time and unknown to the organisms. Organisms are equipped with an evolutionary prior distribution over possible environmental states, denoted by $P( {{{E}_0}} )$ and $P( {{{E}_1}} )$, indicating the initial probability of dispersing into either state (McNamara & Houston, 1980; Mangel, 1990). In line with previous models, we explore low (0.5), moderate (0.7), and high (0.9) priors (Frankenhuis & Panchanathan, 2011a; Panchanathan & Frankenhuis, 2016; Walasek et al., 2022a, 2022b).

During ontogeny, organisms sample cost-free cues (${{C}_0}$ or ${{C}_1}$) in order to infer their environmental state and make adaptive phenotypic decisions. We explore three different durations of ontogeny: 5, 10, and 20 time periods. Ontogeny is followed by adulthood during which organisms accrue fitness but cannot make phenotypic adjustments. How well a cue predicts the environmental state depends on the cue reliability. The cue reliability, $P( {{{C}_x}|{{E}_x}} )$, corresponds to the probability of observing a cue of a specific type conditioned on being in the corresponding environmental state. The probability of observing the opposite cue is given by $P( {{{C}_y}|{{E}_x}} ) = 1 - P( {{{C}_x}|{{E}_x}} )$. Cue reliabilities are symmetric, such that $P( {{{C}_0}|{{E}_0}} ) = P( {{{C}_1}|{{E}_1}} ).$ These probabilities are fixed across an organism’s lifetime. Organisms use cues to update their beliefs about the state of the environment through Bayesian inference (Dall et al., 2015; McNamara & Houston, 1980; McNamara et al., 2006; Stamps & Frankenhuis, 2016; Trimmer et al., 2011). In line with previous models, we explore low (0.55), moderate (0.75), and high (0.95) cue reliabilities.

Our model assumes that organisms are adapted to the structure of the environment—a common assumption across sensitive period models (reviewed in Fawcett & Frankenhuis, 2015; Frankenhuis & Fraley, 2017; Frankenhuis & Walasek, 2020; Walasek et al., 2024). Operationally, this means that organisms develop as if they “know” the prior and cue reliability. This assumption would hold if these parameters were fixed across previous generations. For example, innate predator recognition observed across several species suggests that organisms are adapted to the long-term probability and characteristics of predators (Mezrai et al., 2020; Veen et al., 2000): Observing predators often leads organisms to adjust not only their immediate responses, but also their developmental trajectory—as though these observations provide information about the broader environment rather than the current situation alone. However, if environmental parameters were variable across generations, organisms would need to “estimate” them based on experience (i.e., learn their values within a given generation)—a scenario we do not explore here.

Each environmental state, ${{E}_0}$ and ${{E}_1}$, is associated with an optimal phenotype, ${{P}_0}$ and ${{P}_1}$. We assume that ${{P}_0}$ (e.g., defensive armor) and ${{P}_1}$ (e.g., ornaments to attract mates) are independent and orthogonal trait dimensions. However, the dichotomous nature of the environment creates inherent trade-offs between the two phenotypic targets. During each time period, organisms choose from the following five options: (1) incremental development toward ${{P}_0}$, (2) incremental development toward ${{P}_1}$, (3) deconstruct previous increments toward ${{P}_0}$, (4) deconstruct previous increments toward ${{P}_1}$, or (5) wait and forgo phenotypic changes. We assume that organisms choose the phenotypic option that maximizes long-term expected fitness. In the event of ties among options, organisms choose at random between these options.

We develop two versions of the same model, assuming two different modes of deconstruction: Incremental deconstruction allows organisms to only discard one specialization at a time. Complete deconstruction allows organisms to discard all specializations from one phenotypic target in a single time period. In both versions, phenotypic construction and deconstruction are cost-free. However, the finite duration of ontogeny imposes constraints: Organisms need to balance time invested into plasticity versus specialization.

### Methods: fitness and optimal policies

Mature organisms accrue fitness during adulthood based on how well their phenotype matches their environmental state. Perfectly matched organisms attain maximum fitness, while partially mismatched organisms suffer fitness penalties. To illustrate, suppose an organism at the end of ontogeny receives the cue sequence ${{D}_{{{T}_{\mathrm{ ont}}}}}$, forms posterior estimates $P( {{{E}_{0,{{T}_{\mathrm{ ont}}}}}{\mathrm{|}}{{D}_{{{T}_{\mathrm{ ont}}}}}} )$ and $P( {{{E}_{1,\ {{T}_{\mathrm{ ont}}}\ }}{\mathrm{|}}{{D}_{{{T}_{\mathrm{ ont}}}}}} )$, and develops the phenotype ${{Y}_{\mathrm{ mat}}} = \{ {{{y}_{0,{{T}_{\mathrm{ ont}}}}},{{y}_{1,{{T}_{\mathrm{ ont}}}}}} \}$. We assume that fitness consists of three additive components: baseline fitness (${{\pi }_0}$), rewards for correct phenotypic specializations ($\phi $), and penalties for incorrect phenotypic specializations ($\psi $). An organism that completes ontogeny without specializations toward either phenotypic target achieves baseline fitness. We denote the mapping from phenotypic increments to rewards and penalties by $f( y )$, where $y$ can refer to both ${{y}_0}$ and ${{y}_1}$, and derive the following expressions for expected rewards and penalties at the end of ontogeny:


(1) \begin{eqnarray*}
\phi \left( {{{Y}_{\mathrm{ mat}}}} \right) {=} \ P\left( {{{E}_{0,{{T}_{\mathrm{ ont}}}}}{\mathrm{|}}{{D}_{{{T}_{\mathrm{ ont}}}}}} \right) \bullet f\left( {{{y}_{0,{{T}_{\mathrm{ ont}}}}}} \right) {+} \ P\left( {{{E}_{1,{{T}_{\mathrm{ ont}}}}}{\mathrm{|}}{{D}_{{{T}_{\mathrm{ ont}}}}}} \right) \bullet f\left( {{{y}_{1,{{T}_{\mathrm{ ont}}}}}} \right),
\end{eqnarray*}

(2) \begin{eqnarray*}
\psi \left( {{{Y}_{\mathrm{ mat}}}} \right) {=} P\left( {{{E}_{0,\ {{T}_{\mathrm{ ont}}}}}{\mathrm{|}}{{D}_{{{T}_{\mathrm{ ont}}}}}} \right) \bullet f\left( {{{y}_{1,{{T}_{\mathrm{ ont}}}}}} \right) {+} \ P\left( {{{E}_{1,{{T}_{\mathrm{ ont}}}}}{\mathrm{|}}{{D}_{{{T}_{\mathrm{ ont}}}}}} \right) \bullet f\left( {{{y}_{0,{{T}_{\mathrm{ ont}}}}}} \right).
\end{eqnarray*}

In line with previous models, we set baseline fitness to 0. Expected fitness $\pi ( {{{Y}_{\mathrm{ mat}}}} )$ then corresponds to the difference between expected rewards and penalties:


(3) \begin{eqnarray*}
\pi \left( {{{Y}_{\mathrm{ mat}}}} \right) = {\mathrm{\ }}\phi \left( {{{Y}_{\mathrm{ mat}}}} \right) - \ \psi \left( {{{Y}_{\mathrm{ mat}}}} \right).
\end{eqnarray*}

We map phenotypes onto fitness rewards and penalties in three ways: linear, increasing, and diminishing (Table 1; ESM 1, Supplementary Figure S1.2). Across these mappings, the highest possible fitness remains constant. With diminishing returns, few initial specializations have a major impact on fitness. Conversely, with increasing returns, a larger number of specializations is required to substantially influence fitness. We assign equal weights to rewards gained from correct specializations and the penalties incurred from incorrect ones.

**Table 1.** Mappings between phenotype and fitness rewards and penalties.

Mapping	Formula	Normalization constant
Linear	$f( y ) = y$	–
Diminishing	$f( y ) = \alpha ( {1 - {{e}^{ - \beta y}}} )$	$\beta = 0.2,\ \alpha = \frac{{{{T}_{\mathrm{ ont}}}}}{{1 - {{e}^{ - \beta ( {{{T}_{\mathrm{ ont}}}} )}}}}\ $
Increasing	$f( y ) = \alpha ( {{{e}^{\beta y}} - 1} )$	$\beta = 0.2,\ \alpha = \frac{{{{T}_{\mathrm{ ont}}}}}{{{{e}^{\beta ( {{{T}_{\mathrm{ ont}}}} )\ - \ 1}}}}$

*Note*. The normalization constants for the diminishing and increasing mappings ensure that the maximally attainable fitness corresponds to *T*_ont_.

We use stochastic dynamic programming to compute optimal developmental policies for each combination of prior, cue reliability, and reward–penalty mapping (McNamara & Houston, 1980; Mangel & Clark, 1988; see ESM 1 for details). The optimal policy determines the best, fitness-maximizing, phenotypic choice for every possible developmental state (i.e., sampled cues and current phenotype). This approach ignores how plasticity is instantiated (e.g., genetic correlates of plasticity).

### Methods: analyses

For each combination of parameter values, we quantify plasticity by simulating 10,000 organisms that receive cues and develop according to the optimal policy. At each time step $t$, we clone the set of organisms. From this point onward, the original organisms and their clones are exposed to opposite cues: When one organism samples ${{C}_0}$, its clone samples ${{C}_1}$. For each time step $t$, we quantify plasticity as the normalized, average phenotypic distance between the original organisms and their clones at the end of ontogeny. We compute three phenotypic “distance” measures (see Table 2 and ESM 1 for details): total phenotypic distance (“total plasticity”), distance in construction (“plasticity in construction”), and distance in deconstruction (“plasticity in deconstruction”). Large phenotypic distances indicate high levels of plasticity. In ESM 1, we describe variations of this base paradigm for quantifying plasticity (i.e., less extreme treatment of cues, temporary separation of clones, and distance measurement right after separation).

**Table 2.** Plasticity measures.

Type of plasticity	Phenotypic distance between original organisms (O) and their clones (C)
Total plasticity	Normalized Euclidean distance between the number of specializations toward either phenotypic target (i.e., ${{y}_0}$ and ${{y}_1}$) remaining after deconstruction: $d( {O,\ C} ) = \ \frac{{\sqrt {{{{( {{{y}_{0,O}} - {{y}_{0,C}}} )}}^2} + {{{( {{{y}_{1,O}} - {{y}_{1,C}}} )}}^2}} }}{{2*\sqrt {{{T}_{\mathrm{ Ont}}}} }}$
Plasticity in construction	Normalized Euclidean distance between the number of time periods spent constructing toward each phenotypic target (i.e., ${{y}_{0C}}$ and ${{y}_{1C}}$): $d( {O,\ C} ) = \ \frac{{\sqrt {{{{( {{{y}_{0C,O}} - {{y}_{0C,C}}} )}}^2} + {{{( {{{y}_{1C,O}} - {{y}_{1C,C}}} )}}^2}} }}{{2*\sqrt {{{T}_{\mathrm{ Ont}}}} }}$
Plasticity in deconstruction	Normalized Euclidean distance between the number of time periods spent deconstructing toward each phenotypic target (i.e., ${{y}_{0D}}$ and ${{y}_{1D}}$): $d( {O,\ C} ) = \ \frac{{\sqrt {{{{( {{{y}_{0D,O}} - {{y}_{0D,C}}} )}}^2} + {{{( {{{y}_{1D,O}} - {{y}_{1D,C}}} )}}^2}} }}{{2*\sqrt {\frac{1}{2}*{{T}_{\mathrm{ Ont}}}} }}$

*Note*. Plasticity corresponds to the average, normalized phenotypic distance between separated clones simulated from the optimal policy.

For each combination of parameter values, we evaluate the fitness advantage of phenotypic deconstruction by comparing the fitness of the optimal policy with the fitness of (1) optimal policies that are only capable of incremental, irreversible construction (derived from Panchanathan & Frankenhuis, 2016); (2) specialists that fully specialize toward the phenotypic target matching the most likely environmental state according to the prior distribution (in the case of uninformative priors (0.5), half the population fully specializes toward ${{P}_0}$ and the other half toward ${{P}_1}$); and (3) generalists that specialize halfway toward each phenotypic target (Frankenhuis & Panchanathan, 2011a).

Lastly, we use the optimal policies to simulate distributions of mature phenotypes. Based on these distributions, we can quantify the prevalence of deconstruction across evolutionary ecologies. We can also see whether a population tends to develop more specialized or generalist phenotypes, and how much organisms tend to deconstruct (partial versus full) depending on environmental conditions. Our Python 3.10 code is available on GitHub (https://github.com/Nicole-Walasek/reversible_plasticity).

## Results

This section is organized around the central question of whether and when during ontogeny reversible plasticity—modeled as deconstruction—evolves. First, we outline under what conditions deconstruction evolves in our model. Then, we illustrate when, during ontogeny, cues have the largest impact on phenotype construction and deconstruction.

## Under what conditions does deconstruction evolve?

### Deconstruction increases with uncertainty

**Figure 1.**
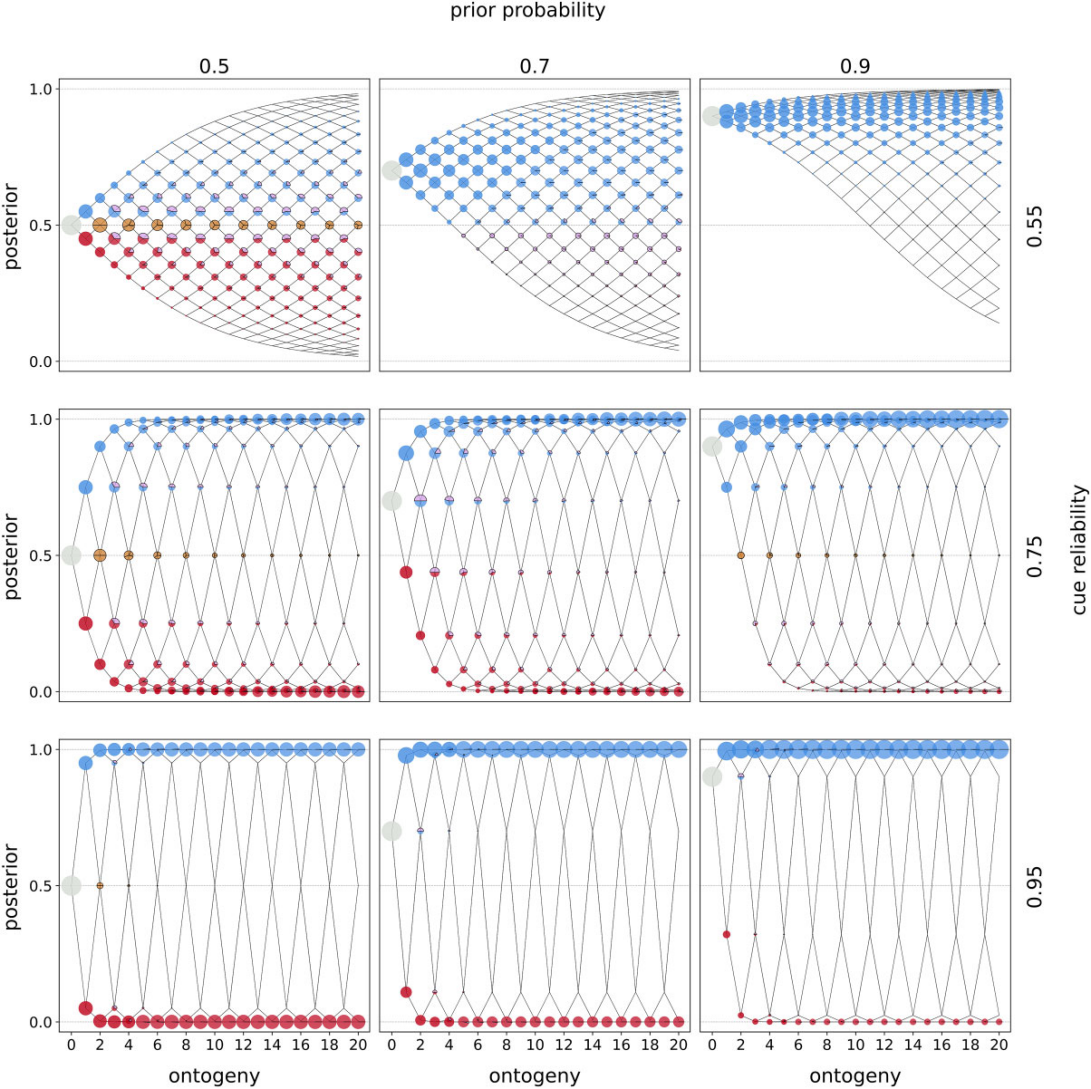
*Environmental uncertainty favors deconstruction*—optimal policies. Columns indicate the prior estimate of being in ${{E}_1}$ and rows indicate the cue reliability. Within each panel, the horizontal axis denotes ontogeny duration and the vertical axis the posterior estimates of being in ${{E}_1}$. The entire population starts ontogeny with zero cues sampled, and the prior estimate is indicated by the row (gray circle). In each time period, organisms sample a cue (${{C}_0}$ or ${{C}_1}$), update their estimate, and make a phenotypic decision (colored circles). The lines indicate developmental trajectories through this decision space, with lines branching upwards indicating the sampling of ${{C}_1}$ and lines branching downwards indicating the sampling of ${{C}_o}$. Circle colors denote the optimal, fitness-maximizing phenotypic choice in each state. Pies indicate cases in which organisms with the same posterior estimates make different phenotypic decisions. Circles or pies with a black outline emphasize cases in which organisms deconstructed. The area of a circle (pie piece) is proportional to the probability of reaching that particular state. Colors indicate the following phenotypic decisions: Black corresponds to waiting, red to constructing ${{P}_0}$, light red to deconstructing ${{P}_1}$, blue to constructing ${{P}_1}$, light blue to deconstructing ${{P}_0}$, purple to ties without deconstruction, light purple (with a black outline added for emphasis) to ties with deconstruction, and lastly, ochre corresponds to a tie between all options. Note that pure deconstruction was never the optimal choice here, which is why light red and light blue circles are not depicted. This figure represents incremental deconstruction and linear rewards and penalties. We show optimal policies for all other reward–penalty mappings in the ESM 2 (Supplementary Figures S2.1–S2.9 for incremental deconstruction and Supplementary Figures S2.10–S2.18 for complete deconstruction). In ESM 2, we show the same optimal policies with a more fine-grained distinction of ties between specific phenotypic decisions (e.g., between constructing ${{P}_1}$ and deconstructing ${{P}_0}$).

Our results show that both incremental deconstruction and complete deconstruction evolve across a range of parameter values (Figure 1; ESM 2, Supplementary Figure S2.19). Comparing optimal policies with incremental and complete deconstruction, we observe that the former only favors deconstruction in ties with other phenotypic choices, such as between specializing toward ${{P}_0}$ and deconstructing a specialization toward ${{P}_1}$ (ESM 2, Supplementary Figure S2.1). In contrast, complete deconstruction favors deconstruction alone (i.e., not in ties) as the fitness-maximizing choice at the end of ontogeny (ESM 2, Supplementary Figure S2.10). Qualitative patterns are otherwise similar across modes of deconstruction.

The prevalence of deconstruction typically decreases as the prior or cue reliability increases. Organisms engage more in construction compared to deconstruction when they are more certain about environmental conditions. Thus, deconstruction is less likely to evolve when organisms can easily infer the environmental state.

We also observe interactions between priors and cue reliabilities. When priors are informative (0.7 and 0.9), deconstruction is most common at moderate cue reliability (0.75). When faced with low cue reliability (0.55), organisms specialize based on their prior estimates, largely ignoring cues. When cue reliability is high (0.95), organisms specialize according to cues with little need for revision. Moderately reliable cues (0.75) mark a sweet spot: Cues are sufficiently informative for organisms to invest in specialization yet leave enough uncertainty to necessitate potential revision of estimates. These conditions favor the occasional deconstruction of specializations.

### Deconstruction frequently evolves when ontogeny is short

**Figure 2.**
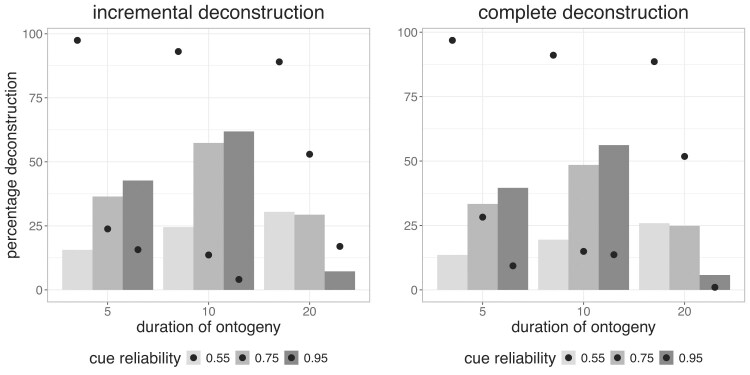
*Shorter ontogeny durations favor higher and more consistent levels of deconstruction across priors*—average levels and % change in deconstruction across priors. The left panel illustrates results for incremental deconstruction and the right panel for complete deconstruction. The horizontal axis displays different durations of ontogeny, and the color of the bars indicates different cue reliabilities. The vertical axis shows the average amount of deconstruction across priors (in %): For a given ontogeny duration and cue reliability, the bar depicts the average level, and the points depict the average % change in deconstruction across priors.

We find that deconstruction frequently evolves across all three durations of ontogeny (5, 10, and 20 time periods; see ESM 3). However, the prevalence of deconstruction depends on the cue reliability. When cue reliability is low (0.55), the amount of both incremental and complete deconstruction remains qualitatively similar across all ontogeny durations (Figure 2). With moderately (0.75) reliable cues, shorter ontogeny durations (5 or 10 time periods) favor a relatively constant proportion of organisms deconstructing, irrespective of their priors (Figure 2). In contrast, when ontogeny is long (20 time periods), deconstruction decreases as priors become more informative (ESM 2, Supplementary Figure S2.19). The reason for this difference is that shorter ontogenies offer fewer opportunities to sample cues. Therefore, despite access to informative cues, a larger proportion of organisms remains uncertain throughout ontogeny. This persistent uncertainty drives higher rates of deconstruction even with high priors (Figure 2). While high (0.95) cue reliability also results in consistent levels of deconstruction across priors, the overall amount of deconstruction is higher with shorter ontogeny durations.

### Deconstruction provides modest fitness benefits

#### *Overall fitness impact* 

**Figure 3.**
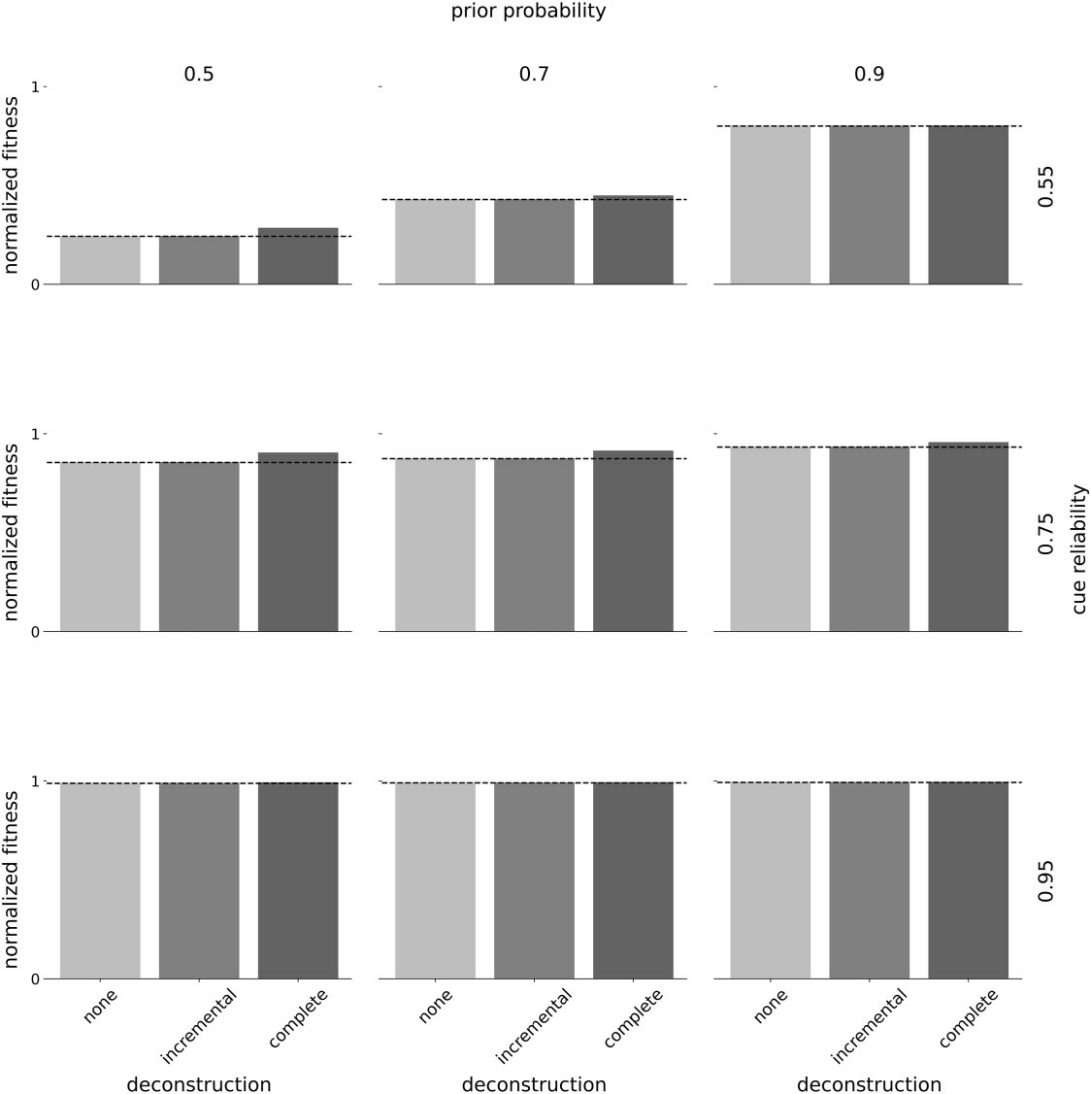
*Deconstruction offers small fitness benefits*—normalized fitness of optimal policies with and without deconstruction. Columns indicate the prior estimate of being in ${{E}_1}$ and rows indicate the cue reliability. Within each panel, the horizontal axis denotes the mode of deconstruction: none, incremental, or complete. The vertical axis denotes fitness achieved by the respective optimal policy, normalized to range between 0 and 1. To facilitate visual comparison across policies, we additionally plot the fitness of the policy without deconstruction as a black, striped, horizontal line. This figure represents linear rewards and penalties. We show fitness for all other reward–penalty mappings and in comparison with two nonplastic strategies (specialists and generalists) in the ESM 2, Supplementary Figures S2.55–S2.63.

Deconstruction provides modest fitness benefits compared to plastic policies without deconstruction (Figure 3) and nonplastic strategies (ESM 2, Supplementary Figure S2.55). With linear rewards and penalties, incremental deconstruction performs similarly to policies without deconstruction. Complete deconstruction offers a small fitness advantage over both these policies (i.e., with incremental deconstruction and without deconstruction). All optimal policies—with and without deconstruction—outperform nonplastic strategies (ESM 2, Supplementary Figures S2.55–S2.63). This fitness advantage grows larger as priors decrease and cue reliabilities increase: Under these conditions, plastic policies can leverage information about the environment obtained from cues.

#### *Conditions favoring complete deconstruction* 

The fitness benefit of complete deconstruction is largest when cues are not highly reliable (0.55 or 0.75) and priors are not highly informative (0.5 or 0.7). Under these conditions, a small proportion of organisms achieve “moderate” certainty about the true environmental state toward the end of ontogeny (Figure 1). Complete deconstruction allows these organisms to fully discard specializations for the alternative environmental state (ESM 2, Supplementary Figure S2.46). This also explains the lack of an advantage of incremental deconstruction in most conditions: Incremental deconstruction is time-consuming such that organisms are typically only able to partially deconstruct specializations toward the suboptimal phenotype within the given time frame (ESM 2, Supplementary Figure S2.37).

#### *Performance under different reward–penalty mappings* 

When the penalties for a few incorrect specializations exceed rewards for a few correct ones, both incremental and complete deconstruction outperform policies and strategies incapable of deconstruction (ESM 2, Supplementary Figures S2.55–S2.63). This occurs under linear rewards/diminishing penalties, increasing rewards/diminishing penalties, and increasing rewards/linear penalties (ESM 2). In these unforgiving ecologies, a combination of waiting and deconstruction often produces mature organisms with zero specializations (ESM 2, Supplementary Figures S2.37–S2.54). These unspecialized organisms try to avoid costly penalties for incorrect specializations, sometimes leading them to deconstruct specializations toward the optimal phenotype. Notably, complete deconstruction consistently outperforms incremental deconstruction. Results from the other two mappings (increasing rewards/increasing penalties and diminishing rewards/diminishing penalties) are qualitatively identical to those of linear rewards and linear penalties.

## When do cues have the largest impact on construction and deconstruction?

Patterns of sensitive periods in both construction and deconstruction are qualitatively similar across different durations of ontogeny (5, 10, and 20 time periods; ESM 3), different treatments of cues (i.e., the extent to which cues between separated organisms differ; ESM 4, Supplementary Figures S4.1–S4.3), and different reward–penalty mappings (ESM 2, Supplementary Figures S2.64–S2.72).

### Sensitive periods for construction evolve at the onset of ontogeny

**Figure 4.**
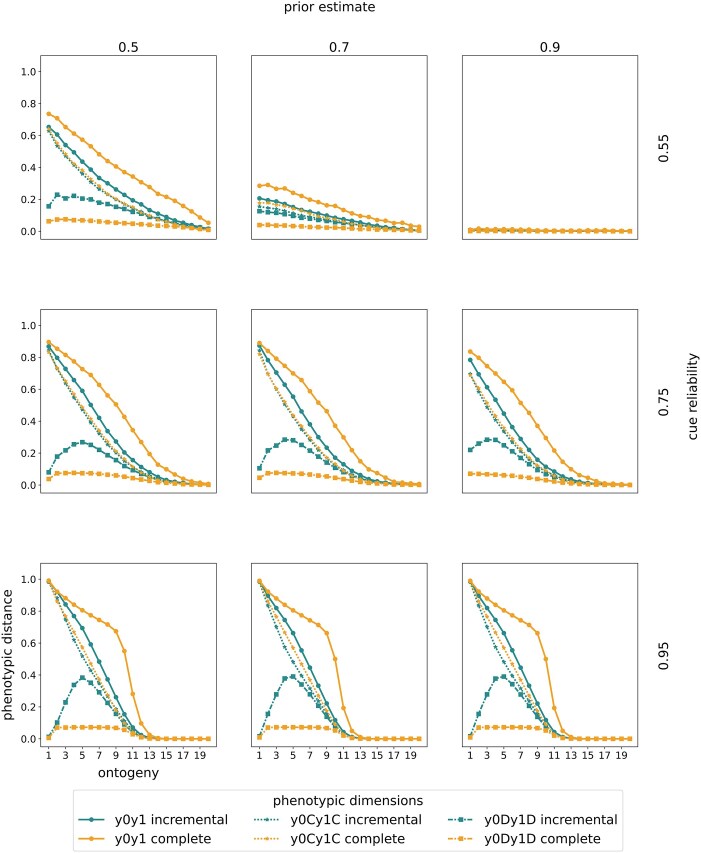
*Early-life sensitive periods in construction precede mid-ontogeny sensitive periods in deconstruction*—changes in plasticity. Columns indicate the prior estimate of being in ${{E}_1}$ and rows indicate the cue reliability. Within each panel, the horizontal axis denotes ontogeny. The vertical axis denotes the normalized average phenotypic distance (a measure of plasticity) across 10,000 pairs of simulated clones measured at the end of ontogeny. A specific point on any of the curves corresponds to a simulation experiment in which clones have been separated at the time point indicated by the horizontal axis. We show results from a model with incremental deconstruction in teal and with complete deconstruction in yellow. For each mode of deconstruction, we present three types of plasticity curves. First, we show plasticity in construction (dotted lines and stars) as the Euclidean distance between the number of time points spent constructing ${{P}_0}$ and ${{P}_1}$ (denoted by ${{y}_{0C}}{{y}_{1C}}$). Second, we show plasticity in deconstruction (dash–dotted lines and squares) as the Euclidean distance between the number of time points spent deconstructing ${{P}_0}$ and ${{P}_1}$ (denoted by ${{y}_{0D}}{{y}_{1D}}$). Third, we show total plasticity (solid lines and circles) as the Euclidean distance between the number of specialization steps toward either target (after accounting for deconstruction; denoted by ${{y}_0}{{y}_1}$). We normalize phenotypic distance in construction and total phenotypes by dividing by the maximally possible Euclidean distance (see Table 2). This figure represents linear rewards and penalties. We show changes in plasticity for all other reward–penalty mappings in ESM 2, Supplementary Figures S2.64–S2.72.

We observe that plasticity in construction is highest at the beginning of ontogeny and gradually declines over time (Figure 4). An exception occurs when priors are high (0.9) and cue reliability is low (0.55). Then, selection favors no plasticity: Organisms ignore uninformative cues and fully specialize based on their priors.

Patterns of plasticity in construction are remarkably similar across different modes of deconstruction (Figure 4). Additionally, these patterns are similar to those produced by policies without deconstruction (Panchanathan & Frankenhuis, 2016). This consistency across policies suggests that the evolution of sensitive periods for construction is independent of whether a species evolves the ability to deconstruct.

### Sensitive periods for deconstruction typically evolve in mid-ontogeny

Across reward–penalty mappings and durations of ontogeny, sensitive periods for deconstruction typically evolve in mid-ontogeny. However, in some cases, we also observe sensitive periods for deconstruction at the onset and even toward the end of ontogeny.

#### *Early-ontogeny sensitive periods* 

When cue reliability is low (0.55), we observe early-ontogeny sensitive periods for deconstruction (except with high priors) with a wide plasticity window. This pattern is driven by a larger proportion of the population remaining uncertain about their environment (Figure 1). Uncertain organisms typically accrue specializations toward both phenotypic targets. As some organisms achieve more certain posteriors, they deconstruct specializations toward the less likely environmental state. Plasticity in deconstruction gradually declines as ontogeny proceeds because there are fewer opportunities for organisms to revise estimates and deconstruct.

#### *Mid-ontogeny sensitive periods* 

When cues are moderately or highly reliable (0.75 or 0.95), we observe mid-ontogeny sensitive periods for deconstruction (Figure 4). Plasticity in deconstruction shows an inverted U-shape: first increasing, then decreasing. Here, organisms exhibit the largest differences in deconstruction after they have begun specializing toward one environmental state. Higher cue reliability leads to earlier specialization and higher peaks in deconstruction plasticity.

#### *Late-ontogeny sensitive periods* 

With temporary separation, we observe sensitive periods for deconstruction toward the end of ontogeny (ESM 4, Supplementary Figures S4.4–S4.5). Organisms temporarily exposed to diverging cues do not immediately alter their phenotypes. Instead, they deconstruct specializations toward the end of ontogeny after becoming more certain of the state of their environment. Here, the effect of cues on deconstruction is often largest toward the end of ontogeny. These findings highlight the need for long-term measurements in observing the effects of temporary separation on phenotypic differences.

#### *Differences between modes of deconstruction* 

We observe quantitative, but not qualitative, differences between incremental and complete deconstruction. Complete deconstruction favors shallow peaks in plasticity. With complete deconstruction, even a few time periods spent deconstructing can significantly affect phenotypic development. With incremental deconstruction, a substantially larger time investment is needed to achieve the same phenotypic outcome. Consequently, patterns of total plasticity deviate notably from plasticity in construction when deconstruction is complete.

## Discussion

We explored the evolution of reversible plasticity when organisms incrementally develop phenotypes in a stable environment. We operationalized reversibility as the ability to deconstruct phenotypic adjustments, exploring two modes of deconstruction: Organisms can either incrementally deconstruct phenotypic adjustments over time or completely deconstruct phenotypic adjustments instantaneously. We highlight four insights and close with caveats and future directions.

### Deconstruction can evolve in stable environments

Existing theory has shown that reversibility evolves in environments that change within generations (Botero et al., 2015; Gabriel, 2005; Gabriel et al., 2005; Pfab et al., 2016; Ratikainen & Kokko, 2019; Utz et al., 2014). Here, reversibility is adaptive because it allows organisms to revise their phenotypes when environmental conditions change. Our model shows that reversibility can evolve even if environments remain constant, so long as organisms are uncertain about the underlying environment. When organisms have to simultaneously learn about their environment while gradually adapting their phenotype, organisms are bound to misspecialize sometimes. This uncertainty from “within” the organism is sufficient to necessitate phenotypic revisions, making reversibility adaptive. This insight resonates with previous work showing that uncertainty in environmental conditions alone can result in individual differences in plasticity (Frankenhuis & Panchanathan, 2011b).

### Shorter ontogeny increases the frequency of deconstruction

Our model shows that shorter durations of ontogeny select for reversibility across a wider range of environmental conditions. All else equal, shorter durations of ontogeny imply shorter individual lifespans. Traditionally, reversibility was thought to primarily benefit long-lived species and to evolve alongside longevity (Hoffmann & Bridle, 2021; Ratikainen & Kokko, 2019). Notably, this work assumes that organisms can make phenotypic adjustments throughout their lifespan. In contrast, our model assumes that organisms can adjust phenotypes only during ontogeny. Under these conditions, the ability to deconstruct may not be limited to long-lived species. Reversible anti-predator defenses in short-lived *Daphnia* and snails fit this finding (Herzog et al., 2016; Hoverman & Relyea, 2007). Although typically only found in long-lived vertebrates, one study observed reversible brain plasticity in insects: Sexually reproducing ants (*Harpegnathos saltator*) show reversible changes in brain volume when switching between forager and reproductive worker phenotypes in adulthood (Penick et al., 2021). However, in contrast to our model, in these ants, trait development is possible across the entire lifespan. Together, these examples suggest that reversibility may evolve across a range of life histories.

### Complete deconstruction offers larger fitness benefits

Consistent with previous models, in our model, complete deconstruction provides a larger fitness benefit compared to incremental deconstruction (Gabriel, 2005; Gabriel et al., 2005; Pfab et al., 2016; Utz et al., 2014). Previous models show that the fitness benefit of reversible plasticity is largest with short response lags—the interval between inferring environmental changes and reversing phenotypes. Although we did not explicitly model response lags, our incremental deconstruction is analogous to lagged reversal and complete deconstruction to immediate reversal. Incremental deconstruction can thus be viewed as a less efficient form of reversibility: Miscalibration is more costly because it takes longer to undo incorrect specializations, often resulting only in partial reversibility. This highlights that reversibility may be limited by organismal (e.g., developmental, physiological, morphological) constraints imposed on traits. Empirical patterns dovetail with these results. As discussed earlier, a study in *D. barbata* showed higher reversibility for adjusting tail-spine shape compared to helmets (Herzog et al., 2016). The lower reversibility of helmets is thought to result from greater physiological constraints on their adjustment compared to tail-spine shape (Utz et al., 2014). Further supporting this pattern, reversibility is more common in behavioral than in morphological traits (Burggren, 2020).

Deconstruction costs and constraints are not the only factors shaping variation in reversible plasticity. *D. barbata* also showed predator-dependent differences in reversibility in the same defensive trait (Herzog et al., 2016): *Daphnia* exposed to tadpoles (*T. cancriformis)* can reverse defensive structures, while *Daphnia* exposed to backswimmers (*N. glauca*; an aquatic insect) cannot. Deconstruction costs cannot explain this difference, because the same trait is being deconstructed in both cases. Our modeling suggests that these predator-dependent differences in reversibility may be linked to the varying fitness costs of incorrect specializations. Our model shows that deconstruction offers the greatest fitness advantages in unforgiving environments where even a few incorrect specializations impose large fitness costs. Therefore, we may expect variation in reversibility when maladaptations to one predator type are more costly than those to another. In their *Daphnia* study, the authors do not provide data on the relative fitness costs of maladaptive specializations for each predator type (Herzog et al., 2016). However, they do note that the removal of predator kairomones is less informative in the case of an aquatic insect with the ability to fly (*N. glauca*), compared to a water-bound tadpole (*T. cancriformis*). The difference in cue reliabilities may explain why reversibility is higher in response to the removal of tadpole kairomones. Our model supports this explanation by showing that reversibility increases with cue reliability. This explanation does not, however, exclude the possibility that organisms may exhibit increased levels of reversibility in response to the more costly predator. Future work could test this hypothesis in *Daphnia* or some other short-lived species using experimental evolution methods (English & Barreaux, 2020).

### Mid-ontogeny sensitive periods for deconstruction

In our model, sensitive periods for deconstruction typically peak during the middle of ontogeny. When cues are informative, deconstruction plasticity increases at first, gradually decreases, and eventually disappears—a process resulting in a critical period (during which plasticity reaches zero; Knudsen, 2004). When cues are less informative, organisms remain uncertain across most of ontogeny. Under these conditions, plasticity in deconstruction tends to be higher at the onset of ontogeny, while also spanning most of ontogeny. These results align with existing models showing that reversibility, defined as the lifelong ability to adjust phenotypes, tends to be favored in environments that change slowly or predictably (Botero et al., 2015; McDermott & Safran, 2021; Ratikainen & Kokko, 2019). By contrast, irreversible plasticity, defined as the ability to make phenotypic adjustments during a delineated time window, should be favored in stable environments (Dupont et al., 2024). All else equal, changing environments are more likely to induce uncertainty about environmental conditions. Thus, summarizing across models, we can identify environmental uncertainty as a driver of prolonged windows of reversibility.

Our model adds to a growing list of models, which, despite different assumptions, produce early-ontogeny sensitive periods. Some of these models assume instantaneous development, such that organisms can express any phenotype at any time (Fischer et al., 2014; Stamps & Krishnan, 2014, 2017). Others assume incremental development without the ability to deconstruct phenotypes (English et al., 2016; Frankenhuis & Panchanathan, 2011a; Panchanathan & Frankenhuis, 2016; Walasek et al., 2022a, 2022b). This robustness across diverse models suggests that selection pressures on early-life sensitive periods are likely widespread across traits and species.

## Caveats and future directions

We outline four future directions that address caveats of our current model. First, models of reversibility may incorporate neurophysiological (Gabard-Durnam & McLaughlin, 2019; Reh et al., 2020) and genetic mechanisms (Kovuri et al., 2023) known to shape plasticity. This would shed light on the relative contribution of neurophysiological, genetic, and environmental factors for maintaining reversible plasticity. We note that models incorporating mechanistic constraints lose some of their generalizability as they become increasingly tailored to specific taxa or species.

Second, future extensions may expand the range of explored scenarios within our present model. Such extensions may incorporate a continuum of environmental states rather than a dichotomous environment (e.g., as in Stamps & Krishnan, 2014, 2017) or costs of plastic adjustments (e.g., as in Fischer et al., 2014). The latter extension would allow us to explore asymmetric costs of construction and deconstruction. It is also possible to consider scenarios in which deconstruction is not only cost-free but also frees up energy that organisms can use to improve the functioning of other traits (e.g., sea squirts resorbing their own nervous system after becoming attached to a rock).

Third, future theory can extend the present work by incorporating environmental changes within an organism’s lifespan. We expect larger fitness benefits of reversibility when environmental conditions change both between and within generations. A previous model has found that rapidly changing environmental conditions favor sensitive periods toward the end of ontogeny (Walasek et al., 2022b). In such conditions, organisms benefit from relying on the most recent information to predict their adult environment. However, if organisms can deconstruct specializations, plasticity in construction can be higher earlier in ontogeny and organisms might instead use late-ontogeny cues for deconstruction. This would imply a link between the evolution of sensitive periods for construction and deconstruction in changing environments.

Fourth, future modeling may help us understand in which conditions natural selection favors the deconstruction of initial specializations if the availability of information about the environment changes across ontogeny. For example, some humans are born with severely impaired vision (congenital cataracts), which can be restored later in life (Bruns & Röder, 2023; Sourav et al., 2024). These individuals develop compensatory abilities early in life in other sensory systems (e.g., hearing). After vision is restored, these compensatory abilities are retained in some cases and “deconstructed” in others. Future models might provide guidance in understanding how the nature and timing of inputs contributes to such differences.

Besides laying the groundwork for future modeling, our findings highlight the need for conceptual revision. Most studies to date have focused on lifelong and fully reversible plasticity, often neglecting temporary and partial reversibility. Echoing previous work (Burggren, 2020; Herzog et al., 2016), our model illustrates how adopting a broader definition of reversibility can capture striking variation across different species and traits. Equipped with this broader definition, we can move toward a unifying theoretical framework that captures the full range of reversible plasticity across the tree of life.

## Supplementary Material

qraf015_Supplemental_File

## Data Availability

No data were collected or used for this paper. Our Python 3.10 code is available on GitHub (https://github.com/Nicole-Walasek/reversible_plasticity).

## References

[bib1] Botero C. A., Weissing F. J., Wright J., Rubenstein D. R. (2015). Evolutionary tipping points in the capacity to adapt to environmental change. Proceedings of the National Academy of Sciences, 112(1), 184–189. 10.1073/pnas.1408589111 PMC4291647 25422451

[bib2] Bruns P., Röder B. (2023). Development and experience-dependence of multisensory spatial processing. Trends in Cognitive Sciences, 27, 961. 10.1016/j.tics.2023.04.012 37208286

[bib3] Burggren W. W. (2020). Phenotypic switching resulting from developmental plasticity: Fixed or reversible?. Frontiers in Physiology, 10, 1–13. 10.3389/fphys.2019.01634 PMC6987144 32038303

[bib4] Dall S. R. X., McNamara J. M., Leimar O. (2015). Genes as cues: Phenotypic integration of genetic and epigenetic information from a Darwinian perspective. Trends in Ecology & Evolution, 30(6), 327–333. 10.1016/j.tree.2015.04.002 25944666

[bib5] Dupont L., Thierry M., Zinger L., Legrand D., Jacob S. (2024). Beyond reaction norms: The temporal dynamics of phenotypic plasticity. Trends in Ecology & Evolution, 39(1), 41–51. 10.1016/j.tree.2023.08.014 37718228

[bib6] English S., Barreaux A. M. (2020). The evolution of sensitive periods in development: Insights from insects. Current Opinion in Behavioral Sciences, 36, 71–78. 10.1016/j.cobeha.2020.07.009

[bib7] English S., Fawcett T. W., Higginson A. D., Trimmer P. C., Uller T. (2016). Adaptive use of information during growth can explain long-term effects of early life experiences. The American Naturalist, 187(5), 620–632. 10.1086/685644 27104994

[bib8] Fawcett T. W., Frankenhuis W. E. (2015). Adaptive explanations for sensitive windows in development. Frontiers in Zoology, 12(Suppl 1), S3. 10.1186/1742-9994-12-S1-S3 26816521 PMC4722342

[bib9] Fischer B., van Doorn G. S., Dieckmann U., Taborsky B. (2014). The evolution of age-dependent plasticity. The American Naturalist, 183(1), 108–125. 10.1086/674008 24334740

[bib10] Frankenhuis W. E., Fraley R. C. (2017). What do evolutionary models teach us about sensitive periods in psychological development?. European Psychologist, 22(3), 141–150. 10.1027/1016-9040/a000265

[bib11] Frankenhuis W. E., Panchanathan K. (2011a). Balancing sampling and specialization: An adaptationist model of incremental development. Proceedings of the Royal Society B: Biological Sciences, 278(1724), 3558–3565. 10.1098/rspb.2011.0055 PMC3189362 21490018

[bib12] Frankenhuis W. E., Panchanathan K. (2011b). Individual differences in developmental plasticity may result from stochastic sampling. Perspectives on Psychological Science, 6(4), 336–347. 10.1177/1745691611412602 26167787

[bib13] Frankenhuis W. E., Walasek N. (2020). Modeling the evolution of sensitive periods. Developmental Cognitive Neuroscience, 41, 100715. 10.1016/j.dcn.2019.100715 31999568 PMC6994616

[bib14] Gabard-Durnam L. J., McLaughlin K. A. (2019). Do sensitive periods exist for exposure to adversity?. Biological Psychiatry, 85(10), 789–791. 10.1016/j.biopsych.2019.03.975 31046937 PMC6580180

[bib15] Gabriel W. (2005). How stress selects for reversible phenotypic plasticity. Journal of Evolutionary Biology, 18(4), 873–883. 10.1111/j.1420-9101.2005.00959.x 16033559

[bib16] Gabriel W., Luttbeg B., Sih A., Tollrian R. (2005). Environmental tolerance, heterogeneity, and the evolution of reversible plastic responses. The American Naturalist, 166(3), 339–353. 10.1086/432558 16224689

[bib17] Herzog Q., Tittgen C., Laforsch C. (2016). Predator-specific reversibility of morphological defenses in *Daphnia barbata*. Journal of Plankton Research, 38(4), 771–780. 10.1093/plankt/fbw045

[bib18] Hoffmann A. A., Bridle J. (2021). The dangers of irreversibility in an age of increased uncertainty: Revisiting plasticity in invertebrates. Oikos, 2022(4), e08715. 10.1111/oik.08715

[bib19] Hoverman J. T., Relyea R. A. (2007). How flexible is phenotypic plasticity? Developmental windows for trait induction and reversal. Ecology, 88(3), 693–705. 10.1890/05-1697. 17503597

[bib20] Knudsen E. I. (2004). Sensitive periods in the development of the brain and behavior. Journal of Cognitive Neuroscience, 16(8), 1412–1425. 10.1162/0898929042304796 15509387

[bib21] Kovuri P., Yadav A., Sinha H. (2023). Role of genetic architecture in phenotypic plasticity. Trends in Genetics, 39(9), 703–714. 10.1016/j.tig.2023.04.002 37173192

[bib24] McDermott M. T., Safran R. J. (2021). Sensitive periods during the development and expression of vertebrate sexual signals: A systematic review. Ecology and Evolution, 11(21), 14416–14432. 10.1002/ece3.8203 34765116 PMC8571593

[bib25] McNamara J. M., Green R. F., Olsson O. (2006). Bayes’ theorem and its applications in animal behaviour. Oikos, 112(2), 243–251. 10.1111/j.0030-1299.2006.14228.x

[bib26] McNamara J. M., Houston A. (1980). The application of statistical decision theory to animal behaviour. Journal of Theoretical Biology, 85(4), 673–690. 10.1016/0022-5193(80)90265-9 7442286

[bib22] Mangel M. (1990). Dynamic information in uncertain and changing worlds. Journal of Theoretical Biology, 146(3), 317–332. 10.1016/S0022-5193(05)80742-8 2259206

[bib23] Mangel M., Clark C. W. (1988). Dynamic modeling in behavioral ecology. Princeton University Press.

[bib27] Mezrai N., Arduini L., Dickel L., Chiao C.-C., Darmaillacq A.-S. (2020). Awareness of danger inside the egg: Evidence of innate and learned predator recognition in cuttlefish embryos. Learning & Behavior, 48(4), 401–410. 10.3758/s13420-020-00424-7 32221844

[bib28] Nettle D., Bateson M. (2015). Adaptive developmental plasticity: What is it, how can we recognize it and when can it evolve?. Proceedings of the Royal Society B: Biological Sciences, 282(1812), 20151005. 10.1098/rspb.2015.1005 PMC4528519 26203000

[bib29] Panchanathan K., Frankenhuis W. E. (2016). The evolution of sensitive periods in a model of incremental development. Proceedings of the Royal Society B: Biological Sciences, 283(1823), 20152439. 10.1098/rspb.2015.2439 PMC4795025 26817766

[bib30] Penick C. A., Ghaninia M., Haight K. L., Opachaloemphan C., Yan H., Reinberg D., Liebig J. (2021). Reversible plasticity in brain size, behaviour and physiology characterizes caste transitions in a socially flexible ant (*Harpegnathos sa l tator*). Proceedings of the Royal Society B: Biological Sciences, 288(1948), 20210141. 10.1098/rspb.2021.0141 PMC8059678 33849311

[bib31] Pfab F., Gabriel W., Utz M. (2016). Reversible phenotypic plasticity with continuous adaptation. Journal of Mathematical Biology, 72(1–2), 435–466. 10.1007/s00285-015-0890-3 25980476

[bib32] Piersma T., Drent J. (2003). Phenotypic flexibility and the evolution of organismal design. Trends in Ecology & Evolution, 18(5), 228–233. 10.1016/S0169-5347(03)00036-3

[bib33] Ratikainen I. I., Kokko H. (2019). The coevolution of lifespan and reversible plasticity. Nature Communications, 10(1), 1–7. 10.1038/s41467-019-08502-9 PMC6358619 30710077

[bib34] Reh R. K., Dias B. G., Nelson C. A., Kaufer D., Werker J. F., Kolb B., Levine J. D., Hensch T. K. (2020). Critical period regulation across multiple timescales. Proceedings of the National Academy of Sciences, 117, 23242. 10.1073/pnas.1820836117 PMC7519216 32503914

[bib35] Sourav S., Kekunnaya R., Bottari D., Shareef I., Pitchaimuthu K., Röder B. (2024). Sound suppresses earliest visual cortical processing after sight recovery in congenitally blind humans. Communications Biology, 7(1), 1–14. 10.1038/s42003-023-05749-3 38253781 PMC10803735

[bib36] Stamps J. A., Frankenhuis W. E. (2016). Bayesian models of development. Trends in Ecology & Evolution, 31, 260–268. 10.1016/j.tree.2016.01.012 26896042

[bib37] Stamps J. A., Krishnan V. V. (2014). Combining information from ancestors and personal experiences to predict individual differences in developmental trajectories. The American Naturalist, 184(5), 647–657. 10.1086/678116 25325748

[bib38] Stamps J. A., Krishnan V. V. (2017). Age-dependent changes in behavioural plasticity: Insights from Bayesian models of development. Animal Behaviour, 126, 53–67. 10.1016/j.anbehav.2017.01.013

[bib39] Stearns S. C. (1989). The evolutionary significance of phenotypic plasticity. Bioscience, 39(7), 436–445. 10.2307/1311135

[bib40] Trimmer P. C., Houston A. I., Marshall J. A. R., Mendl M. T., Paul E. S., McNamara J. M. (2011). Decision-making under uncertainty: Biases and Bayesians. Animal Cognition, 14(4), 465–476. 10.1007/s10071-011-0387-4 21360119

[bib41] Utz M., Jeschke J. M., Loeschcke V., Gabriel W. (2014). Phenotypic plasticity with instantaneous but delayed switches. Journal of Theoretical Biology, 340, 60–72. 10.1016/j.jtbi.2013.08.038 24041594

[bib42] Veen T., Richardson D. S., Blaakmeer K., Komdeur J. (2000). Experimental evidence for innate predator recognition in the Seychelles warbler. Proceedings of the Royal Society of London. Series B: Biological Sciences, 267(1459), 2253–2258. 10.1098/rspb.2000.1276 PMC1690806 11413640

[bib43] Walasek N., Frankenhuis W. E., Panchanathan K. (2022a). An evolutionary model of sensitive periods when the reliability of cues varies across ontogeny. Behavioral Ecology, 33(1), 101–114. 10.1093/beheco/arab113 35197808 PMC8857937

[bib44] Walasek N., Frankenhuis W. E., Panchanathan K. (2022b). Sensitive periods, but not critical periods, evolve in a fluctuating environment: A model of incremental development. Proceedings of the Royal Society B: Biological Sciences, 289(1969), 20212623. 10.1098/rspb.2021.2623 PMC8848242 35168396

[bib45] Walasek N., Panchanathan K., Frankenhuis W. E. (2024). The evolution of sensitive periods beyond early ontogeny: Bridging theory and data. Functional Ecology, Advance online publication. 10.1111/1365-2435.14615

[bib46] West-Eberhard M. J. (2003). Developmental plasticity and evolution. Oxford University Press.

