# Supplementary material for: The evolution of reversible plasticity in stable environments
Source: Evol Lett. 2025 Jul 8;9(4):491–501. doi: 10.1093/evlett/qraf015 (PMC12448235; doi:10.1093/evlett/qraf015)
Supplement: qraf015_Supplemental_File [file qraf015_supplemental_file.pdf]

Electronic Supplementary Material

The evolution of reversible plasticity in stable environments

ESM 1	Dynamic programming	Variables and explanation	2-3
		Decisions under uncertainty	3-4
		Fitness functions	4-6
		Optimal decisions	6-7
		Quantifying plasticity	7-8
ESM 2	Main plots for all penalty and reward functions	Optimal policies (incremental deconstruction)	9-19
		Optimal policies (complete deconstruction)	20-31
		Triangular distributions of mature phenotypes (incremental deconstruction)	32-40
		Triangular distributions of mature phenotypes (complete deconstruction)	41-49
		Fine-grained distributions of mature phenotypes (incremental deconstruction)	50-58
		Fine-grained distributions of mature phenotypes (complete deconstruction)	59-67
		Normalized fitness	68-76
		Changes in plasticity	77-90
ESM 3	Comparison of 5 and 10 time periods of on ontogeny	5 time periods	91-97
		10 time periods	98-104
ESM 4	Different paradigms for quantifying plasticity (linear rewards and penalties)		105-112

ESM 1 – Dynamic programming

a) Variables and explanation

Environmental variable	Explanation
E_0	Environment 0
E_1	Environment 1
P_0	Optimal phenotype for E_0
P_1	Optimal phenotype for E_1
C_0	Cue indicating E_0
C_1	Cue indicating E_1
D_t	$D_t = \{c_0, c_1\}$, denotes the cue set sampled until time period t where c_0, c_1 indicate the number of cues of each kind (C_0 or C_1).
t	Current time period ranges from $t = 0$ (birth) until T_{Ont} (the end of ontogeny).
T_{ont}	Duration of ontogeny, i.e. ontogeny lasts for 5, 10, or 20 time periods in this model

In each time period (from 1 until T_{Ont}) organisms first sample a cue and then make a phenotypic decision. Organisms can choose one of five options: (1) incrementally develop towards P_0, (2) incrementally develop towards P_1, (3) deconstruct previously developed phenotypic specializations towards P_0, (4) deconstruct previously developed phenotypic specializations towards P_1, or (5) wait and forgo phenotypic changes. We developed two versions of the same model, assuming two different modes of phenotypic deconstruction. In the first version, we assume phenotypes can be incrementally deconstructed: During any specific time period, an organism can only deconstruct one phenotypic adjustment from one phenotypic target. In the second model, we assume complete deconstruction: During any time period, an organism can choose to discard all previously developed specializations from one phenotypic target (Figure S1.1). For both modes of deconstruction, the state of an organism at any time period t is characterized by a 9-tuple $(D_t, y_{0C}, y_{1C}, y_{0D}, y_{1D}, y_0, y_1, y_w, t)$.

Variable	Explanation
y_{0C}	Number of times spent constructing P_0
y_{1C}	Number of times spent constructingP_1
y_{0D}	Number of times spent deconstructing P_0
y_{1D}	Number of times spent deconstructingP_1
y_0	Total number of specialization steps towards P_0 (accounts for deconstruction)
y_1	Total number of specialization steps towards P_1(accounts for deconstruction)
y_w	Number of time steps spent waiting

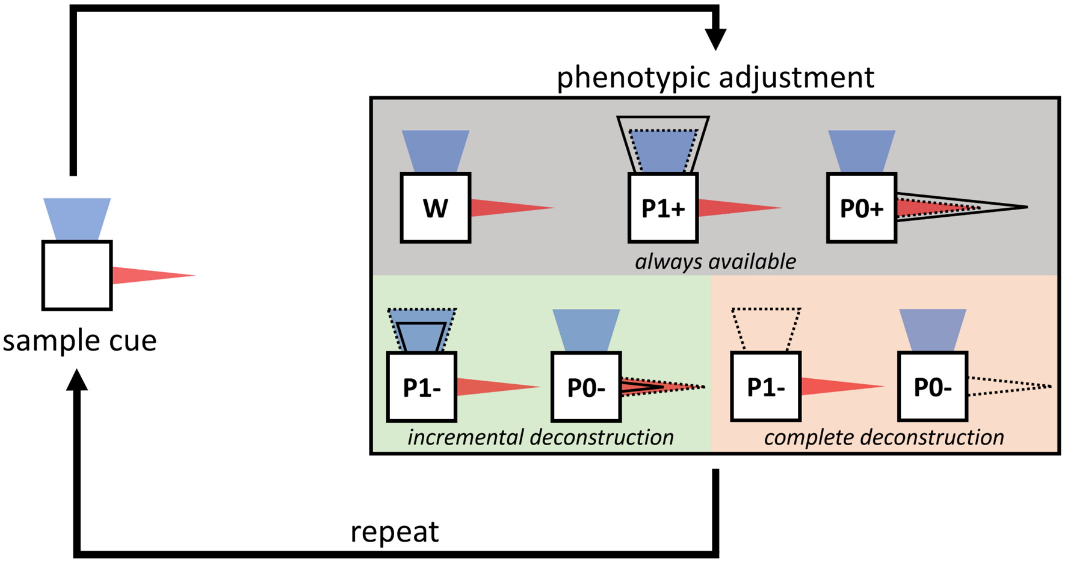

Figure S1.1: Overview of possible developmental decisions. We explore two models with different modes of deconstruction: incremental (green box) and complete (peach box). In either model, at any time period t during ontogeny, organisms can choose one of five phenotypic options. Organisms can wait and forgo phenotypic adjustments or incrementally specialize towards either phenotypic target (red cone, P_0 or blue helmet, P_1). These options are always available in both models (grey box). When deconstruction is incremental, organisms also choose to undo one phenotypic adjustment from either phenotypic target. When deconstruction is abrupt, organisms can choose to fully discard all phenotypic adjustments from either phenotypic target.

b) Decisions under uncertainty

Organisms use Bayesian inference to update their initial prior estimate of the environmental state based on the sampled cues.

Parameters for Bayesian inference	Explanation
$P(E_0)$	Prior probability of E_0
$P(E_1)$	Prior probability of E_1
$P(C_0\|E_0)$	Cue reliability; conditional probability of receiving C_0 in E_0
$P(C_1\|E_1)$	Cue reliability; conditional probability of receiving C_1 in E_1
$P(E_0\|D_t)$	Posterior probability of E_0 after having sampled D_t
$P(E_1\|D_t)$	Posterior probability of E_1 after having sampled D_t
$P(D_t\|E_0)$	Probability of observing the current cue set in E_0. We use the binomial distribution to compute this probability.
$P(D_t\|E_1)$	Probability of observing the current cue set in E_1. We use the binomial distribution to compute this probability.

According to the laws of probability it holds that:

$$P(E_0) + P(E_1) = 1$$
$$P(E_0|D_t) + P(E_1|D_t) = 1$$
$$P(C_0|E_0) + P(C_1|E_0) = 1$$
$$P(C_1|E_1) + P(C_0|E_1) = 1$$

Further, we assume that $P(C_0|E_0) = P(C_1|E_1)$.

Based on this, we compute the posterior probabilities $P(E_0|D_t)$ and $P(E_1|D_t)$ according to:

$$P(E_0|D_t) = \frac{P(D_t|E_0) * P(E_0)}{P(D_t|E_0) * P(E_0) + P(D_t|E_1) * P(E_1)}$$

$$P(E_1|D_t) = \frac{P(D_t|E_1) * P(E_1)}{P(D_t|E_0) * P(E_0) + P(D_t|E_1) * P(E_1)}$$

c) Fitness functions

We denote the mature phenotype at the end of ontogeny by $Y_{mat} = (y_0, y_1, T_{ont})$.

Functions and constants	Explanation
$\phi(Y_{mat})$	Expected, fitness reward at the end of ontogeny
$\psi(Y_{mat})$	Expected, fitness penalty at the end of ontogeny
$\pi(Y_{mat})$	Expected fitness at the end of ontogeny
π_0	Baseline fitness (hypothetical range is [-inf,+inf]; we have set it to zero in line with previous work)
$f(y)$	Mapping between phenotypic increments and fitness rewards (or penalties)

Fitness consequences of phenotypic decisions are not accrued throughout ontogeny but only at the end of ontogeny. The fitness difference from baseline corresponds to the total rewards for correct specializations minus penalties from incorrect specializations, where each correct increment results in a marginal gain and each incorrect increment results in a marginal penalty. We studied three mappings between correct (or incorrect) phenotypic development and fitness rewards (or penalties).

Suppose a mature organism is in the following state at the end of ontogeny $(D_{T_{ont}}, y_0, y_1, y_w, T_{ont})$ having sampled the sequence of cues $D_{T_{ont}}$ and developed the mature phenotype $Y_{mat} = \{y_0, y_1, T_{ont}\}$. Its posterior estimates $P(E_{0,T_{ont}}|D_{T_{ont}})$ and $P(E_{1,T_{ont}}|D_{T_{ont}})$ at the end of ontogeny reflect the probabilities of being in either environmental state at the end of ontogeny.

To compute rewards and penalties, we need to compute the expectation across both environmental states, weighted by how likely each state is as indicated by the posterior estimates. We denote the mapping from phenotypic increments to rewards and penalties by $f(y)$, where y can refer to both y_0 and y_1, and derive the following expressions for expected rewards and penalties at the end of ontogeny:

$$\phi(Y_{mat}) = P\left(E_{0,T_{ont}} \middle| D_{T_{ont}}\right) \bullet f(y_0) + P\left(E_{1,T_{ont}} \middle| D_{T_{ont}}\right) \bullet f(y_1)$$
$$\psi(Y_{mat}) = -\left(P\left(E_{0,T_{ont}} \middle| D_{T_{ont}}\right) \bullet f(y_1) + P\left(E_{1,T_{ont}} \middle| D_{T_{ont}}\right) \bullet f(y_0)\right)$$

Expected fitness $\pi(Y_{mat})$ corresponds to the sum of expected rewards and penalties, in addition to the baseline fitness:

$$\pi(Y_{mat}) = \pi_0 + \phi(Y_{mat}) + \psi(Y_{mat})$$
$$= \pi_0 + \left(P\left(E_{0,T_{ont}} \middle| D_{T_{ont}}\right) - P\left(E_{1,T_{ont}} \middle| D_{T_{ont}}\right)\right) * (f(y_0) - f(y_1))$$

Lastly, we present the three functional mappings between the realized phenotype and fitness rewards and penalties:

Returns on fitness - $f(y)$	Formula	Parameter settings to ensure that maximal rewards and penalties correspond to T_{ont}
linear	$f(y) = y$	-
diminishing	$f(y) = \alpha(1 - e^{-\beta y})$	$\beta = 0.2, \alpha = \frac{T_{ont}}{1 - e^{-\beta(T_{ont})}}$
increasing	$f(y) = \alpha(e^{\beta y} - 1)$	$\beta = 0.2, \alpha = \frac{T_{ont}}{e^{\beta(T_{ont}) - 1}}$

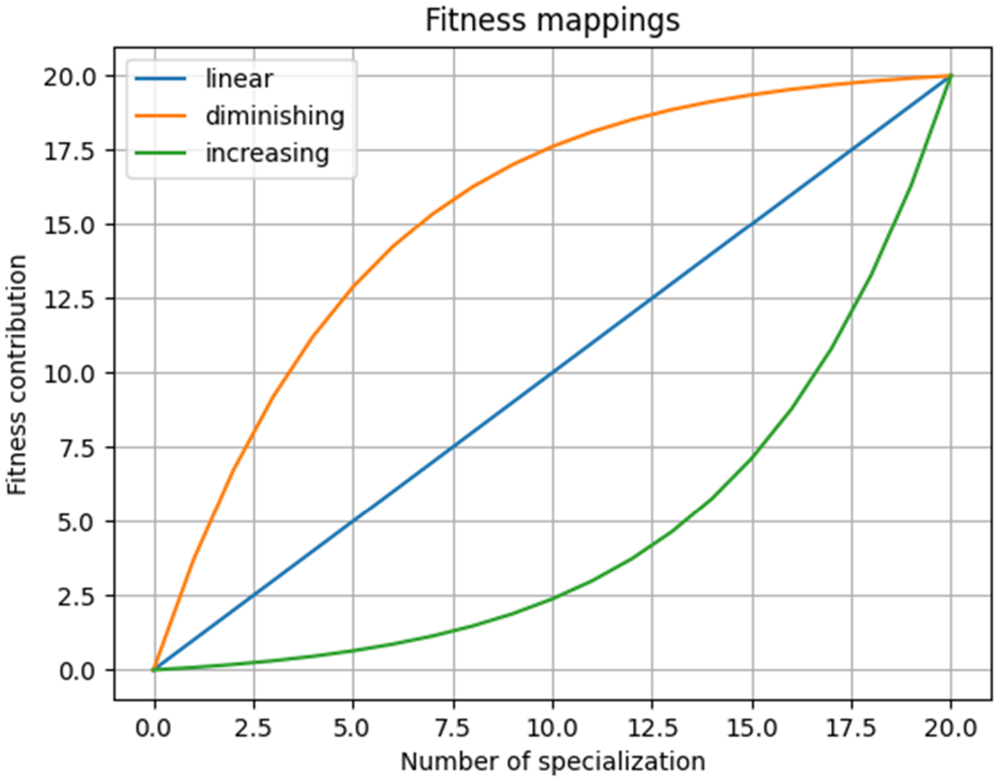

Figure S1.2: Mappings between specializations and fitness consequences.

d) Optimal decisions

In each time period, a developing organism can choose one of five options: increment one step on P_0, increment one step on P_1, decrement one step on P_0, decrement one step on P_1, or wait and forgo specialization. It chooses the option with the highest expected fitness at the end of ontogeny. In the event of a tie between two or all of the options the organism chooses amongst the current alternatives with equal probability.

$F(D_t, y_{0C}, y_{1C}, y_{0D}, y_{1D}, y_0, y_1, y_w, t, T_{ont})$ denotes the maximum expected fitness that can be attained across adulthood as a result of decisions made between t and T_{ont}, when the organism's current state after the last cue sampled is $(y_{0C}, y_{1C}, y_{0D}, y_{1D}, y_0, y_1, y_w)$ and the organisms chooses option a, so that:

$$F(D_t, y_{0C}, y_{1C}, y_{0D}, y_{1D}, y_0, y_1, y_w, t, T_{ont}) = \max_{a \epsilon \{0C,1C,0D,1D,w\}} F_a \text{ , where}$$

$$\begin{aligned}
F_{0C} &= F(D_{t+1}, y_{0C}+1, y_{1C}, y_{0D}, y_{1D}, y_0, y_1, y_w, t+1, T_{ont}),\\
F_{1C} &= F(D_{t+1}, y_{0C}, y_{1C}+1, y_{0D}, y_{1D}, y_0, y_1, y_w, t+1, T_{ont}),\\
F_{0D} &= F(D_{t+1}, y_{0C}, y_{1C}, y_{0D}+1, y_{1D}, y_0-x, y_1, y_w, t+1, T_{ont}),\\
F_{1D} &= F(D_{t+1}, y_{0C}, y_{1C}, y_{0D}, y_{1D}+1, y_0, y_1-x, y_w, t+1, T_{ont}),\\
F_w &= F(D_{t+1}, y_{0C}, y_{1C}, y_{0D}, y_{1D}, y_0, y_1, y_w+1, t+1, T_{ont}).
\end{aligned}$$

Note, the value of x depends on the mode of deconstruction. When deconstruction is incremental it corresponds to 1. When deconstruction is complete it corresponds to the current value of y_0 or y_1. Moreover, organisms can only choose to decrement one step on P_0 if $y_0 > 1$. Likewise, organisms can only choose to decrement one step on P_1 if $y_1 > 1$

We apply backwards induction to solve the dynamic programming equation $F(D_t, y_{0C}, y_{1C}, y_{0D}, y_{1D}, y_0, y_1, y_w, t, T_{ont})$ for all t. We start with $t = T_{ont}$:

$$F\left(D_{T_{ont}}, y_{0C}, y_{1C}, y_{0D}, y_{1D}, y_0, y_1, y_w, T_{ont}, T_{ont}\right) = \pi(Y_{mat}).$$

After calculating expected fitness at the end of ontogeny we continue by decrementing t. For each $t < T_{ont}$ we compute the a, which maximizes $F(D_{t+1}, y_{0C}, y_{1C}, y_{0D}, y_{1D}, y_0, y_1, y_w, t+1, T_{ont})$ in time period t.

e) Quantifying plasticity

To quantify plasticity, we simulate adoption experiments based on the optimal policies, mirroring empirical research methods. We simulate an organism that receives cues and develops according to the optimal policy. At some time period t during ontogeny, we clone the organism. The original and the clone are then separated. After separation, the original and its clone are exposed to the opposite cues: When the original samples C_0, the clone samples C_1, and vice versa (note: we discuss alternative treatments for divergent cues below). We quantify plasticity as the phenotypic distance between original and clone at the end of ontogeny. Large phenotypic distances indicate high levels of plasticity. For each developmental time period, we simulate 10.000 pairs to account for the stochasticity in sequences of cues. Plasticity then corresponds to the average phenotypic distance across pairs of clones separated during that time period.

For each pair of clones, we compute three phenotypic 'distance' measures: total phenotypic distance ('total plasticity'), distance in construction ('plasticity in construction'), and distance in deconstruction ('plasticity in deconstruction'); see table below. Our separate distance measures provide insights into when during ontogeny cues have the largest impact on (de)construction and how this plasticity shapes total phenotypic development. Distance in construction corresponds to the Euclidean distance between the number of time periods spent constructing towards each phenotypic target. Likewise, distance in deconstruction corresponds to the Euclidean distance between the number of time periods spent deconstructing. Total phenotypic distance then corresponds to the Euclidean distance between the number of specializations towards either phenotypic target (remaining after deconstruction). We normalize all distance measures to range between 0 and 1 by dividing each measure by the maximally attainable distance. Total phenotypic distance closely maps onto empirical measures of plasticity. Empiricists routinely measure plasticity as the phenotypic difference between individuals in a control and treatment group at the end of the observation period (Stamps & Luttbeg, 2022).

We explore alternative methods for measuring plasticity to mimic a broader range of empirical study paradigms and to gain insight into their robustness across different empirical scenarios. In line with previous work (Walasek et al., 2022a), we vary the 'base

paradigm' described above along three dimensions: treatment, exposure duration, and measurement time. *Treatment* refers to the degree of divergence between cues experienced by originals and their cloned counterparts. Besides the reciprocal opposite cues (base paradigm), we explore two less extreme treatments in which the clone receives cues representative of the opposite patch or uninformative cues (a form of informational deprivation). *Exposure duration* refers to the amount of time originals and clones are separated. Besides permanent separation (base paradigm), we explore temporary separation after which original and clone are reunited again. *Measurement time* refers to the developmental time period in which the phenotypic distance is measured. Besides measuring phenotypic distance at the end of ontogeny (base paradigm), we measure phenotypic distance immediately after the separation period. Note that varying the measurement time is only meaningful for temporary separation.

**Type of plasticity**	**Phenotypic distance between originals (O) and clones (C)**
Total plasticity	Normalized Euclidean distance between the number of specializations towards either phenotypic target (i.e. y_0 and y_1) remaining after deconstruction: $$d(O,C) = \frac{\sqrt{(y_{0,O} - y_{0,C})^2 + (y_{1,O} - y_{1,C})^2}}{2 * \sqrt{T_{Ont}}}$$
Plasticity in construction	Normalized Euclidean distance between the number of time periods spent constructing towards each phenotypic target (i.e. y_{0C} and y_{1C}): $$d(O,C) = \frac{\sqrt{(y_{0C,O} - y_{0C,C})^2 + (y_{1C,O} - y_{1C,C})^2}}{2 * \sqrt{T_{Ont}}}$$
Plasticity in deconstruction	Normalized Euclidean distance between the number of time periods spent deconstructing towards each phenotypic target (i.e. y_{0D} and y_{1D}): $$d(O,C) = \frac{\sqrt{(y_{0C,O} - y_{0C,C})^2 + (y_{1D,O} - y_{1D,C})^2}}{2 * \sqrt{\frac{1}{2} * T_{Ont}}}$$

Table S1.1: Quantifying plasticity.

ESM 2 - Main plots for all penalty and reward functions

Optimal Policies (incremental deconstruction)

Linear rewards and linear penalties

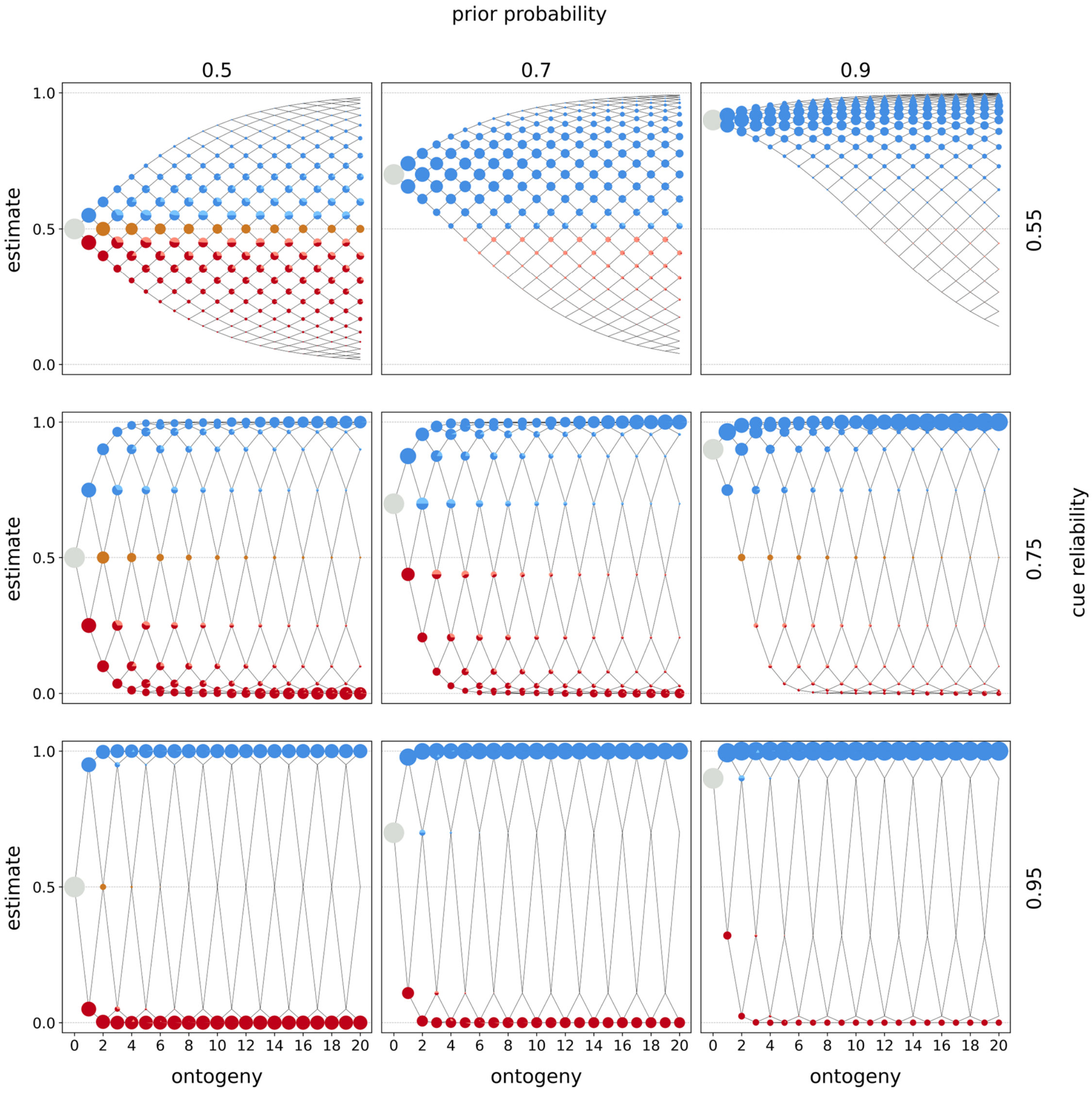

Figure S2.1: Optimal policies. Optimal policies are shown for a model with incremental deconstruction and linear rewards and penalties. Columns indicate the prior estimate of being in E_1 and rows indicate the cue reliability. Within each panel, the horizontal axis denotes ontogeny and the vertical axis the posterior estimates of being in E_1. The entire population starts ontogeny with zero cues sampled and the prior estimate indicated by the column (indicated by the grey circle). In each time period organisms sample a cue (either C_0 or C_1), update their estimate, and make a phenotypic decision (colored circles). Black lines indicate developmental trajectories through this decision space, with lines branching upwards indicating the sampling of C_1 and lines branching downwards indicating the sampling of C_o. Colors denote the optimal, fitness-maximizing phenotypic choice in each state. Pies indicate cases in which organisms with the same posterior estimates make different phenotypic decisions. The area of a circle (pie piece) is proportional the probability of reaching that particular state. Colors indicate the following phenotypic decisions: Black corresponds to waiting, red to constructing P_0, blue to constructing P_1, purple to deconstructing P_0, green to deconstructing P_1, light red to a tie between constructing P_0 and deconstructing P_1, light blue to a tie

between constructing P_1 and deconstructing P_0, brown to a tie between constructing either phenotypic target, yellow to a tie between deconstructing either target, grey to a tie between construction and waiting, dark grey to a tie between deconstruction and waiting, and lastly ochre to a tie between all options.

Linear rewards and increasing penalties

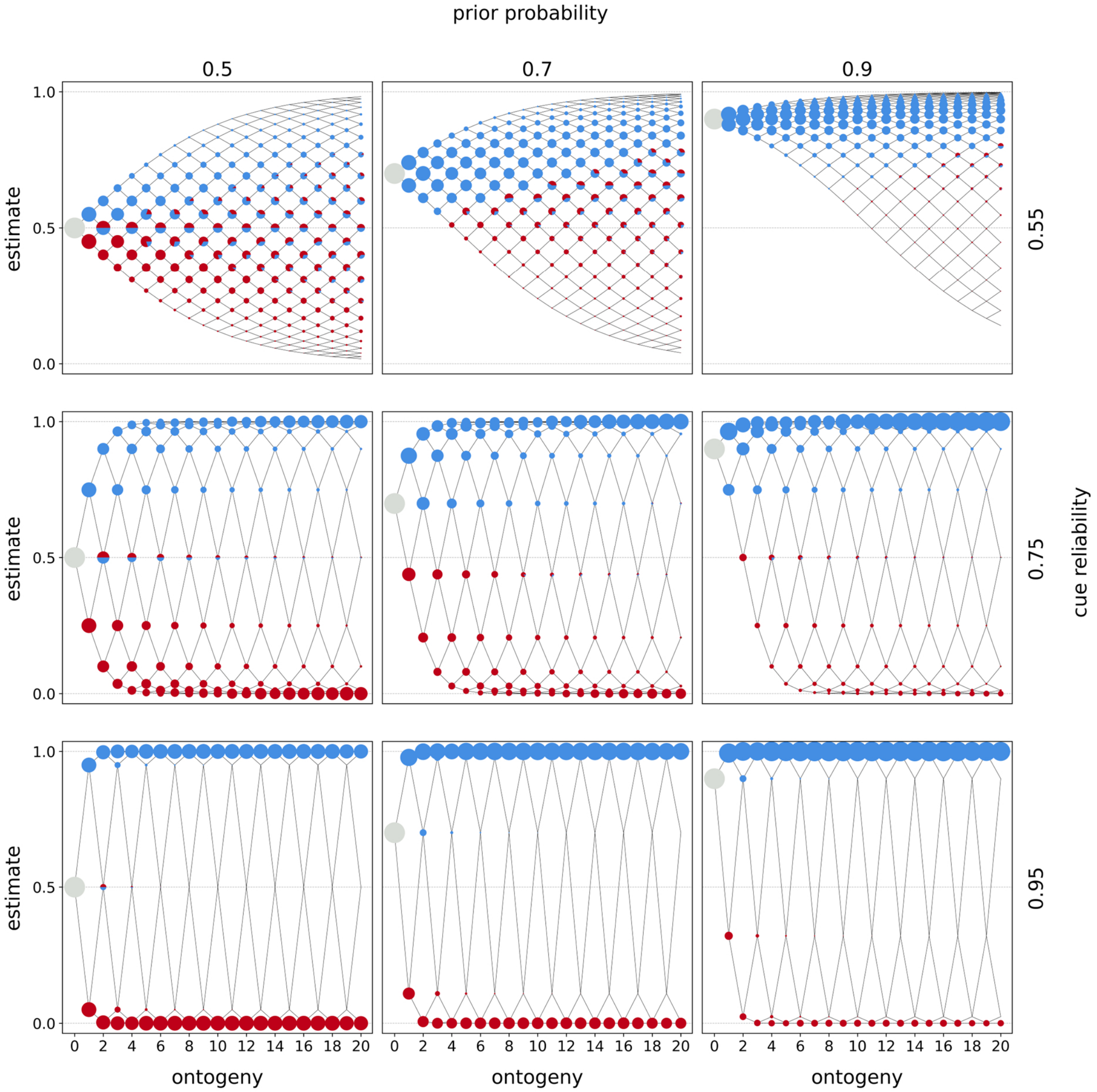

Figure S2.2: Optimal policies. Optimal policies are shown for a model with incremental deconstruction and linear rewards and increasing penalties. Columns indicate the prior estimate of being in E_1 and rows indicate the cue reliability. Within each panel, the horizontal axis denotes ontogeny and the vertical axis the posterior estimates of being in E_1. The entire population starts ontogeny with zero cues sampled and the prior estimate indicated by the column (indicated by the grey circle). In each time period organisms sample a cue (either C_0 or C_1), update their estimate, and make a phenotypic decision (colored circles). Black lines indicate developmental trajectories through this decision space, with lines branching upwards indicating the sampling of C_1 and lines branching downwards indicating the sampling of C_o. Colors denote the optimal, fitness-maximizing phenotypic choice in each state. Pies indicate cases in which organisms with the same posterior estimates make different phenotypic decisions. The area of a circle (pie piece) is proportional the probability of reaching that particular state. Colors indicate the following phenotypic

decisions: Black corresponds to waiting, red to constructing P_0, blue to constructing P_1, purple to deconstructing P_0, green to deconstructing P_1, light red to a tie between constructing P_0 and deconstructing P_1, light blue to a tie between constructing P_1 and deconstructing P_0, brown to a tie between constructing either phenotypic target, yellow to a tie between deconstructing either target, grey to a tie between construction and waiting, dark grey to a tie between deconstruction and waiting, and lastly ochre to a tie between all options.

Linear rewards and diminishing penalties

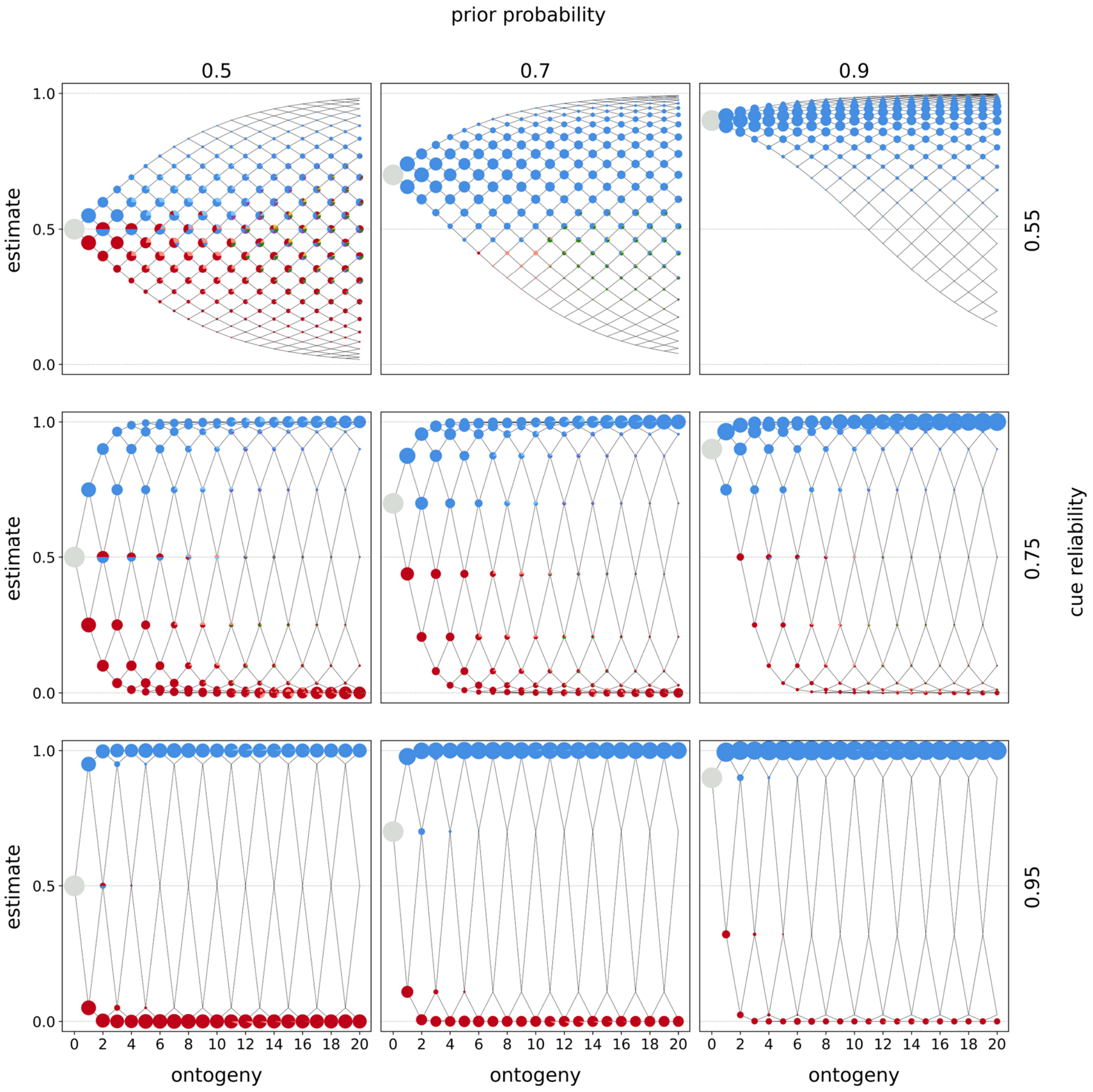

Figure S2.3: Optimal policies. Optimal policies are shown for a model with incremental deconstruction and linear rewards and diminishing penalties. Columns indicate the prior estimate of being in E_1 and rows indicate the cue reliability. Within each panel, the horizontal axis denotes ontogeny and the vertical axis the posterior estimates of being in E_1. The entire population starts ontogeny with zero cues sampled and the prior estimate indicated by the column (indicated by the grey circle). In each time period organisms sample a cue (either C_0 or C_1), update their estimate, and make a phenotypic decision (colored circles). Black lines indicate developmental trajectories through this decision space, with lines branching upwards indicating the sampling of C_1 and lines branching downwards indicating the sampling of C_o. Colors denote

the optimal, fitness-maximizing phenotypic choice in each state. Pies indicate cases in which organisms with the same posterior estimates make different phenotypic decisions. The area of a circle (pie piece) is proportional the probability of reaching that particular state. Colors indicate the following phenotypic decisions: Black corresponds to waiting, red to constructing P_0, blue to constructing P_1, purple to deconstructing P_0, green to deconstructing P_1, light red to a tie between constructing P_0 and deconstructing P_1, light blue to a tie between constructing P_1 and deconstructing P_0, brown to a tie between constructing either phenotypic target, yellow to a tie between deconstructing either target, grey to a tie between construction and waiting, dark grey to a tie between deconstruction and waiting, and lastly ochre to a tie between all options.

Increasing rewards and linear penalties

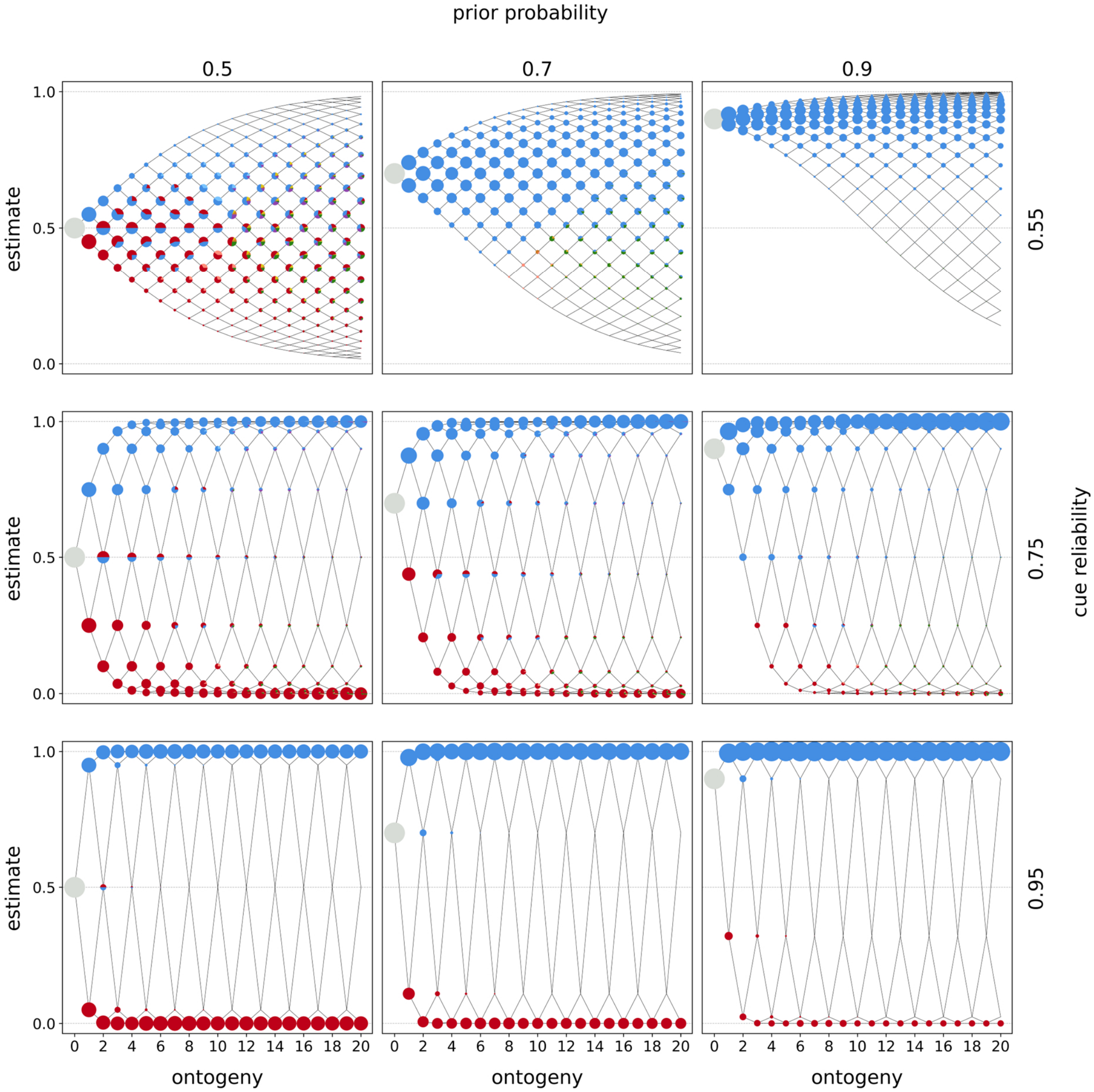

Figure S2.4: Optimal policies. Optimal policies are shown for a model with incremental deconstruction and increasing rewards and linear penalties. Columns indicate the prior estimate of being in E_1 and rows indicate the cue reliability. Within each panel, the horizontal axis denotes ontogeny and the vertical axis the posterior estimates of being in E_1. The entire population starts ontogeny with zero cues sampled and the prior estimate indicated by the column (indicated by the grey circle). In each time period organisms sample a cue (either C_0 or C_1), update their estimate, and make a phenotypic decision (colored circles). Black lines indicate developmental trajectories through this decision space, with lines branching upwards indicating the sampling of C_1 and lines branching downwards indicating the sampling of C_o. Colors denote the optimal, fitness-maximizing phenotypic choice in each state. Pies indicate cases in which organisms with the same posterior estimates make different phenotypic decisions. The area of a circle (pie piece) is proportional the probability of reaching that particular state. Colors indicate the following phenotypic decisions: Black corresponds to waiting, red to constructing P_0, blue to constructing P_1, purple to deconstructing P_0, green to deconstructing P_1, light red to a tie between constructing P_0 and deconstructing P_1, light blue to a tie between constructing P_1 and deconstructing P_0, brown to a tie between constructing either phenotypic target, yellow to a tie between deconstructing either target, grey to a tie between construction and waiting, dark grey to a tie between deconstruction and waiting, and lastly ochre to a tie between all options.

Increasing rewards and increasing penalties

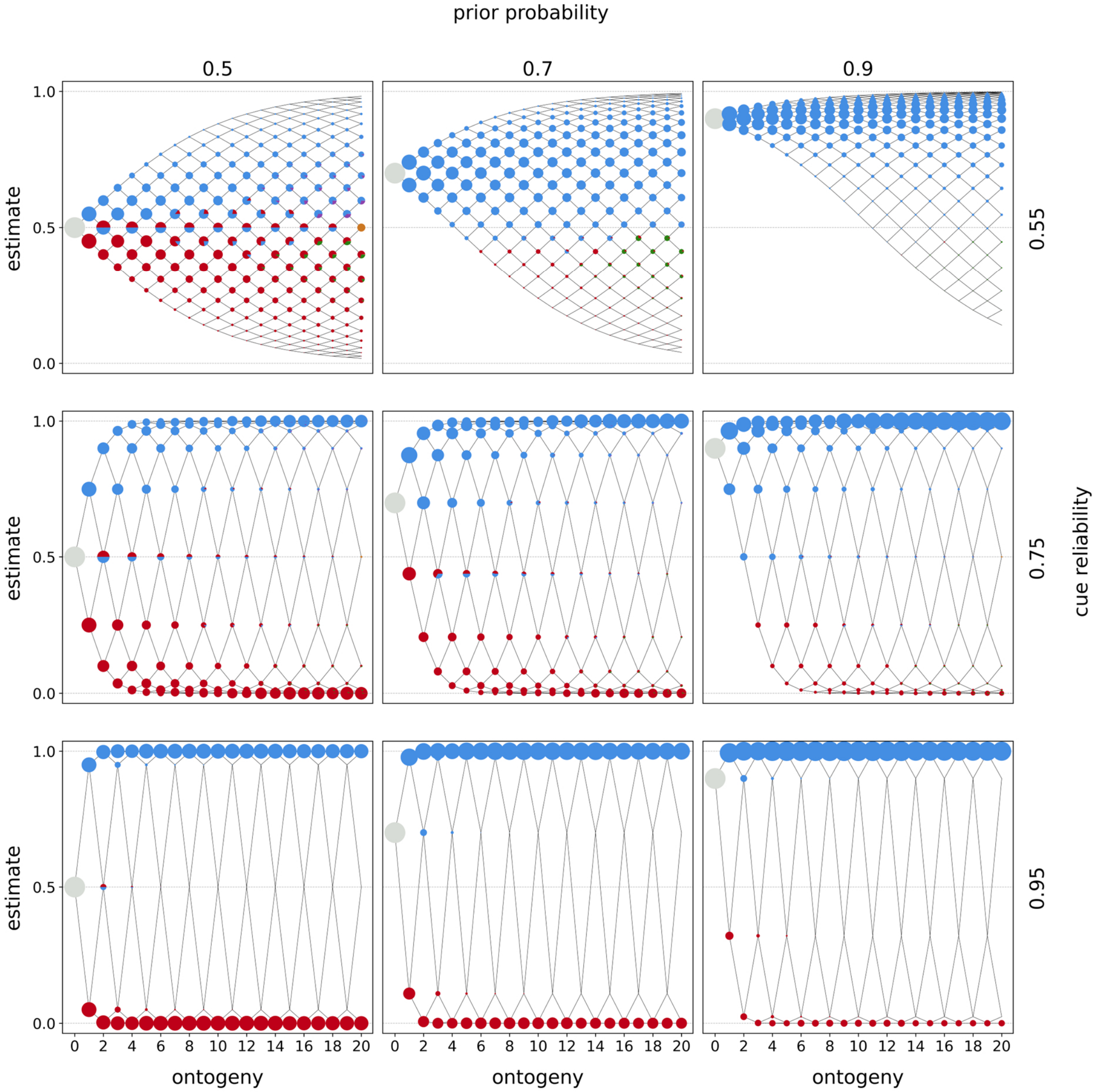

Figure S2.5: Optimal policies. Optimal policies are shown for a model with incremental deconstruction and increasing rewards and increasing penalties. Columns indicate the prior estimate of being in E_1 and rows indicate the cue reliability. Within each panel, the horizontal axis denotes ontogeny and the vertical axis the posterior estimates of being in E_1. The entire population starts ontogeny with zero cues sampled and the prior estimate indicated by the column (indicated by the grey circle). In each time period organisms sample a cue (either C_0 or C_1), update their estimate, and make a phenotypic decision (colored circles). Black lines indicate developmental trajectories through this decision space, with lines branching upwards indicating the sampling of C_1 and lines branching downwards indicating the sampling of C_o. Colors denote the optimal, fitness-maximizing phenotypic choice in each state. Pies indicate cases in which organisms with the same posterior estimates make different phenotypic decisions. The area of a circle (pie piece) is proportional the probability of reaching that particular state. Colors indicate the following phenotypic decisions: Black corresponds to waiting, red to constructing P_0, blue to constructing P_1, purple to deconstructing P_0, green to deconstructing P_1, light red to a tie between constructing P_0 and deconstructing P_1, light blue to a tie between constructing P_1 and deconstructing P_0, brown to a tie between constructing either phenotypic target, yellow to a tie between deconstructing either target, grey to a tie between

construction and waiting, dark grey to a tie between deconstruction and waiting, and lastly ochre to a tie between all options.

Increasing rewards and diminishing penalties

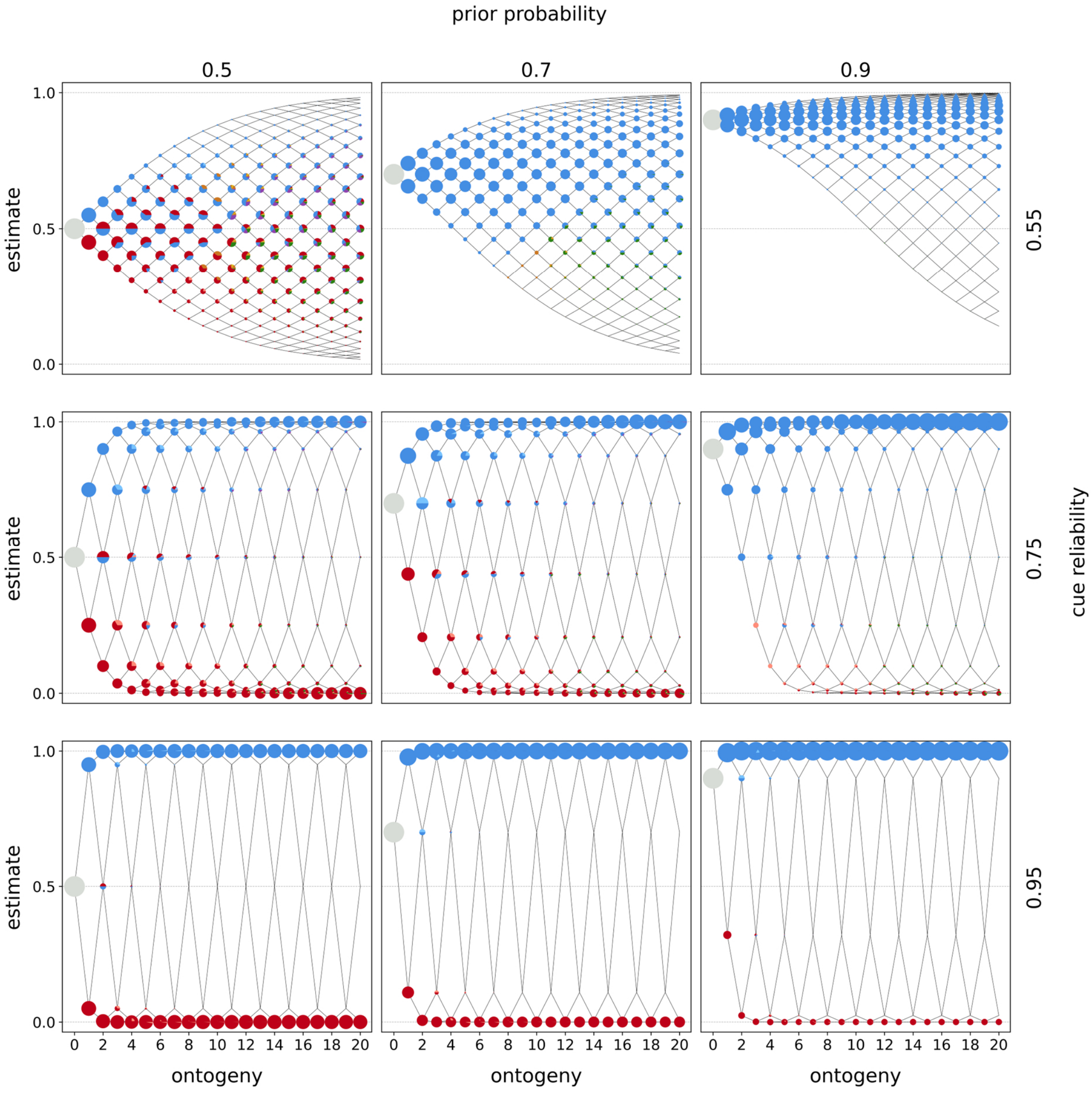

Figure S2.6: Optimal policies. Optimal policies are shown for a model with incremental deconstruction and increasing rewards and diminishing penalties. Columns indicate the prior estimate of being in E_1 and rows indicate the cue reliability. Within each panel, the horizontal axis denotes ontogeny and the vertical axis the posterior estimates of being in E_1. The entire population starts ontogeny with zero cues sampled and the prior estimate indicated by the column (indicated by the grey circle). In each time period organisms sample a cue (either C_0 or C_1), update their estimate, and make a phenotypic decision (colored circles). Black lines indicate developmental trajectories through this decision space, with lines branching upwards indicating the sampling of C_1 and lines branching downwards indicating the sampling of C_o. Colors denote the optimal, fitness-maximizing phenotypic choice in each state. Pies indicate cases in which organisms with the same posterior estimates make different phenotypic decisions. The area of a circle (pie piece) is proportional the probability of reaching that particular state. Colors indicate the following phenotypic decisions: Black corresponds to waiting, red to constructing P_0, blue to constructing P_1, purple to

deconstructing P_0, green to deconstructing P_1, light red to a tie between constructing P_0 and deconstructing P_1, light blue to a tie between constructing P_1 and deconstructing P_0, brown to a tie between constructing either phenotypic target, yellow to a tie between deconstructing either target, grey to a tie between construction and waiting, dark grey to a tie between deconstruction and waiting, and lastly ochre to a tie between all options.

Diminishing rewards and linear penalties

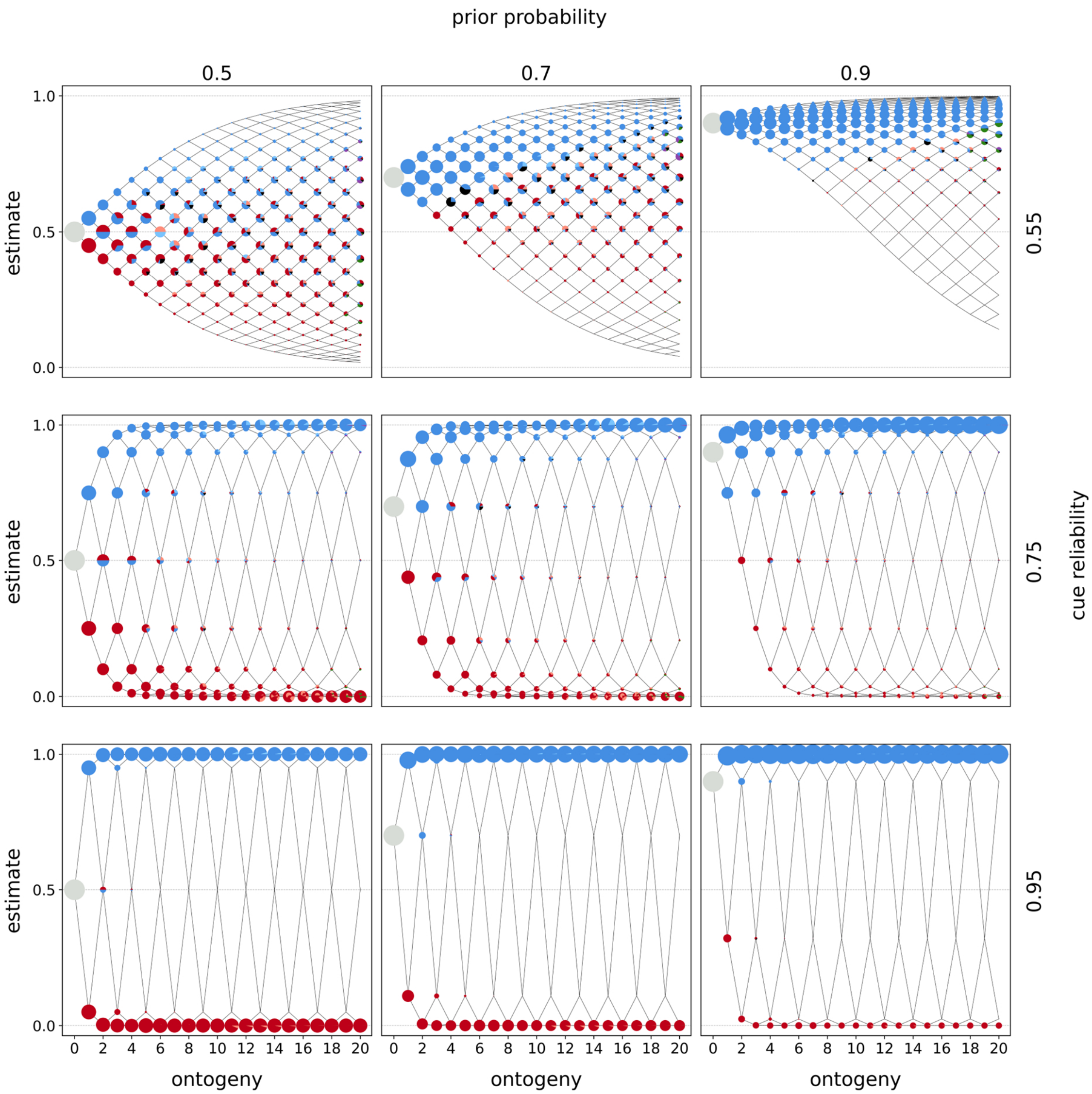

Figure S2.7: Optimal policies. Optimal policies are shown for a model with incremental deconstruction and diminishing rewards and linear penalties. Columns indicate the prior estimate of being in E_1 and rows indicate the cue reliability. Within each panel, the horizontal axis denotes ontogeny and the vertical axis the posterior estimates of being in E_1. The entire population starts ontogeny with zero cues sampled and the prior estimate indicated by the column (indicated by the grey circle). In each time period organisms sample a cue (either C_0 or C_1), update their estimate, and make a phenotypic decision (colored circles). Black lines indicate developmental trajectories through this decision space, with lines branching upwards indicating the sampling of C_1 and lines branching downwards indicating the sampling of C_o. Colors denote the optimal, fitness-maximizing phenotypic choice in each state. Pies indicate cases in which organisms with the same posterior estimates make different phenotypic decisions. The area of a circle (pie piece) is proportional the probability of reaching that particular state. Colors indicate the following phenotypic decisions: Black corresponds to waiting, red to constructing P_0, blue to constructing P_1, purple to deconstructing P_0, green to deconstructing P_1, light red to a tie between constructing P_0 and deconstructing P_1, light blue to a tie between constructing P_1 and deconstructing P_0, brown to a tie between constructing either phenotypic target, yellow to a tie between deconstructing either target, grey to a tie between construction and waiting, dark grey to a tie between deconstruction and waiting, and lastly ochre to a tie between all options.

Diminishing rewards and increasing penalties

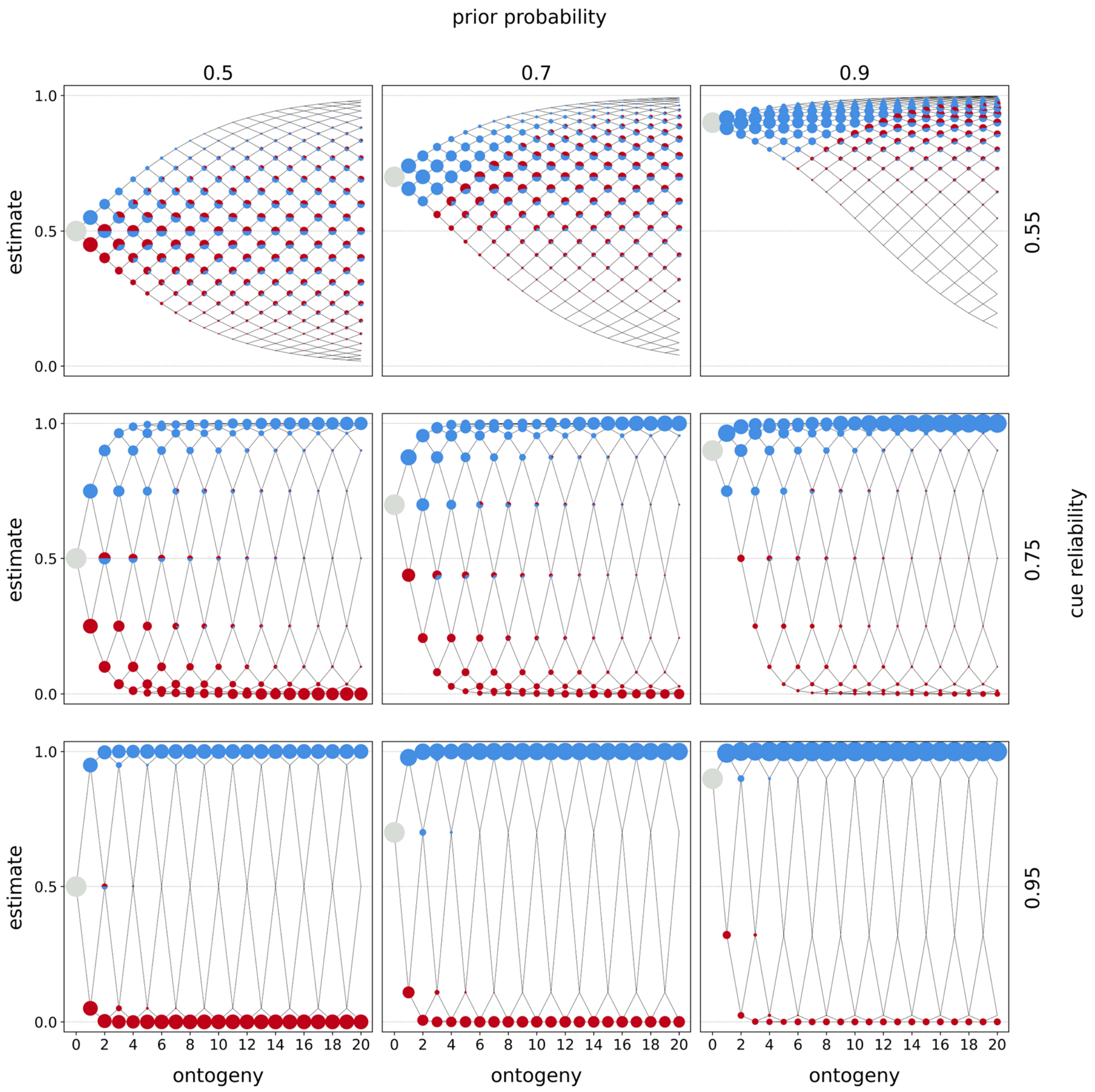

Figure S2.8: Optimal policies. Optimal policies are shown for a model with incremental deconstruction and diminishing rewards and increasing penalties. Columns indicate the prior estimate of being in E_1 and rows indicate the cue reliability. Within each panel, the horizontal axis denotes ontogeny and the vertical axis the posterior estimates of being in E_1. The entire population starts ontogeny with zero cues sampled and the prior estimate indicated by the column (indicated by the grey circle). In each time period organisms sample a cue (either C_0 or C_1), update their estimate, and make a phenotypic decision (colored circles). Black lines indicate developmental trajectories through this decision space, with lines branching upwards indicating the sampling of C_1 and lines branching downwards indicating the sampling of C_o. Colors denote the optimal, fitness-maximizing phenotypic choice in each state. Pies indicate cases in which organisms with the same posterior estimates make different phenotypic decisions. The area of a circle (pie piece) is proportional the probability of reaching that particular state. Colors indicate the following phenotypic decisions: Black corresponds to waiting, red to constructing P_0, blue to constructing P_1, purple to deconstructing P_0, green to deconstructing P_1, light red to a tie between constructing P_0 and deconstructing P_1, light blue to a tie between constructing P_1 and deconstructing P_0, brown to a tie between constructing either phenotypic target, yellow to a tie between deconstructing either target, grey to a tie between construction and waiting, dark grey to a tie between deconstruction and waiting, and lastly ochre to a tie between all options.

Diminishing rewards and diminishing penalties

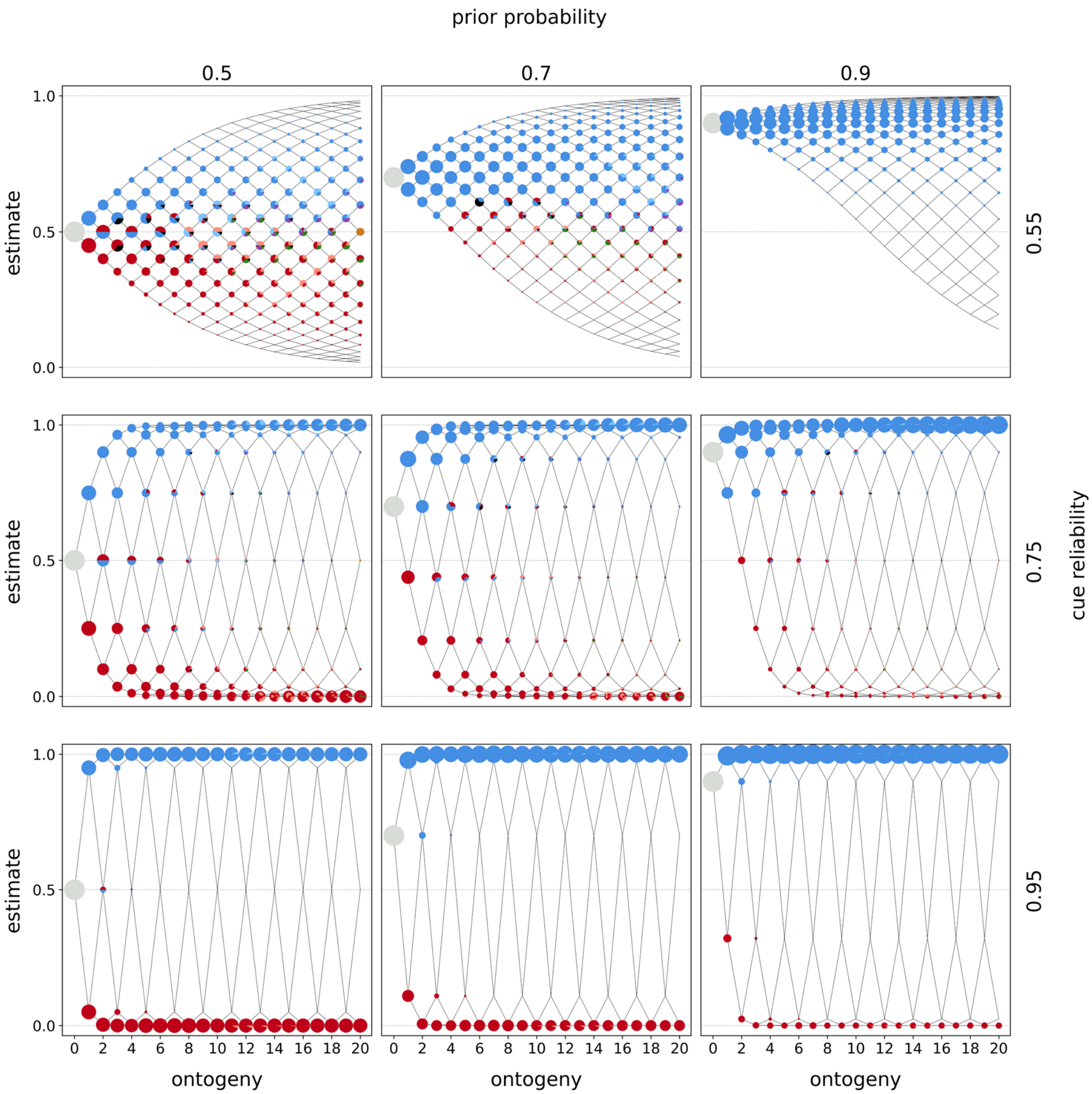

Figure S2.9: Optimal policies. Optimal policies are shown for a model with incremental deconstruction and diminishing rewards and diminishing penalties. Columns indicate the prior estimate of being in E_1 and rows indicate the cue reliability. Within each panel, the horizontal axis denotes ontogeny and the vertical axis the posterior estimates of being in E_1. The entire population starts ontogeny with zero cues sampled and the prior estimate indicated by the column (indicated by the grey circle). In each time period organisms sample a cue (either C_0 or C_1), update their estimate, and make a phenotypic decision (colored circles). Black lines indicate developmental trajectories through this decision space, with lines branching upwards indicating the sampling of C_1 and lines branching downwards indicating the sampling of C_o. Colors denote the optimal, fitness-maximizing phenotypic choice in each state. Pies indicate cases in which organisms with the same posterior estimates make different phenotypic decisions. The area of a circle (pie piece) is proportional the probability of reaching that particular state. Colors indicate the following phenotypic decisions: Black corresponds to waiting, red to constructing P_0, blue to constructing P_1, purple to deconstructing P_0, green to deconstructing P_1, light red to a tie between constructing P_0 and deconstructing P_1, light blue to a tie between constructing P_1 and deconstructing P_0, brown to a tie between constructing either phenotypic target, yellow to a tie between deconstructing either target, grey to a tie between construction and waiting, dark grey to a tie between deconstruction and waiting, and lastly ochre to a tie between all options.

Optimal Policies (complete deconstruction)

Linear rewards and linear penalties

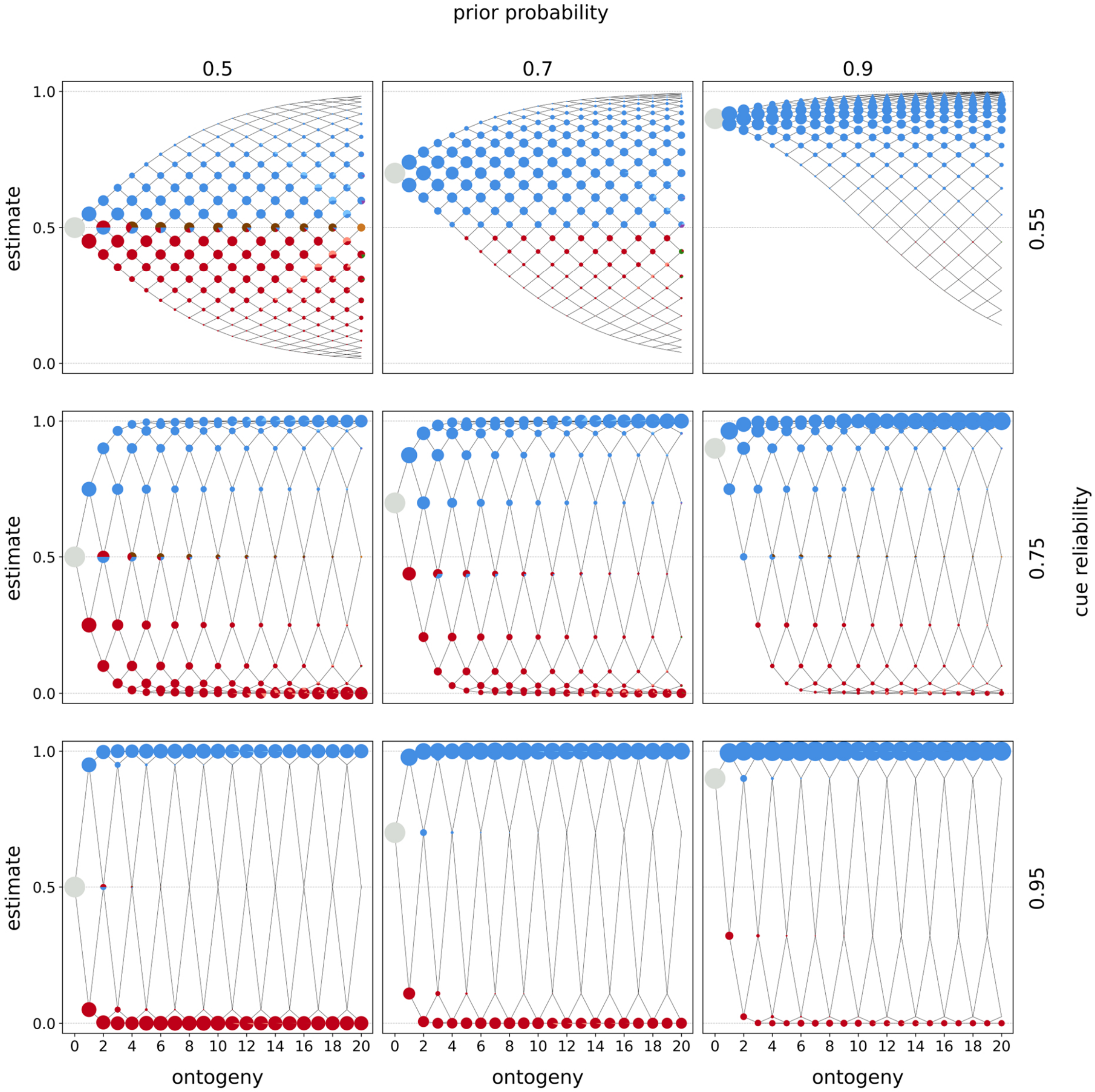

Figure S2.10: Optimal policies. Optimal policies are shown for a model with complete deconstruction and linear rewards and penalties. Columns indicate the prior estimate of being in E_1 and rows indicate the cue reliability. Within each panel, the horizontal axis denotes ontogeny and the vertical axis the posterior estimates of being in E_1. The entire population starts ontogeny with zero cues sampled and the prior estimate indicated by the column (indicated by the grey circle). In each time period organisms sample a cue (either C_0 or C_1), update their estimate, and make a phenotypic decision (colored circles). Black lines indicate developmental trajectories through this decision space, with lines branching upwards indicating the sampling of C_1 and lines branching downwards indicating the sampling of C_o. Colors denote the optimal, fitness-maximizing phenotypic choice in each state. Pies indicate cases in which organisms with the same posterior estimates make different phenotypic decisions. The area of a circle (pie piece) is proportional the probability of reaching that particular state. Colors indicate the following phenotypic decisions: Black corresponds to waiting, red to constructing P_0, blue to constructing P_1, purple to deconstructing P_0, green to deconstructing P_1, light red to a tie between constructing P_0 and deconstructing P_1, light blue to a tie between constructing P_1 and deconstructing P_0, brown to a tie between constructing either phenotypic

target, yellow to a tie between deconstructing either target, grey to a tie between construction and waiting, dark grey to a tie between deconstruction and waiting, and lastly ochre to a tie between all options.

Linear rewards and increasing penalties

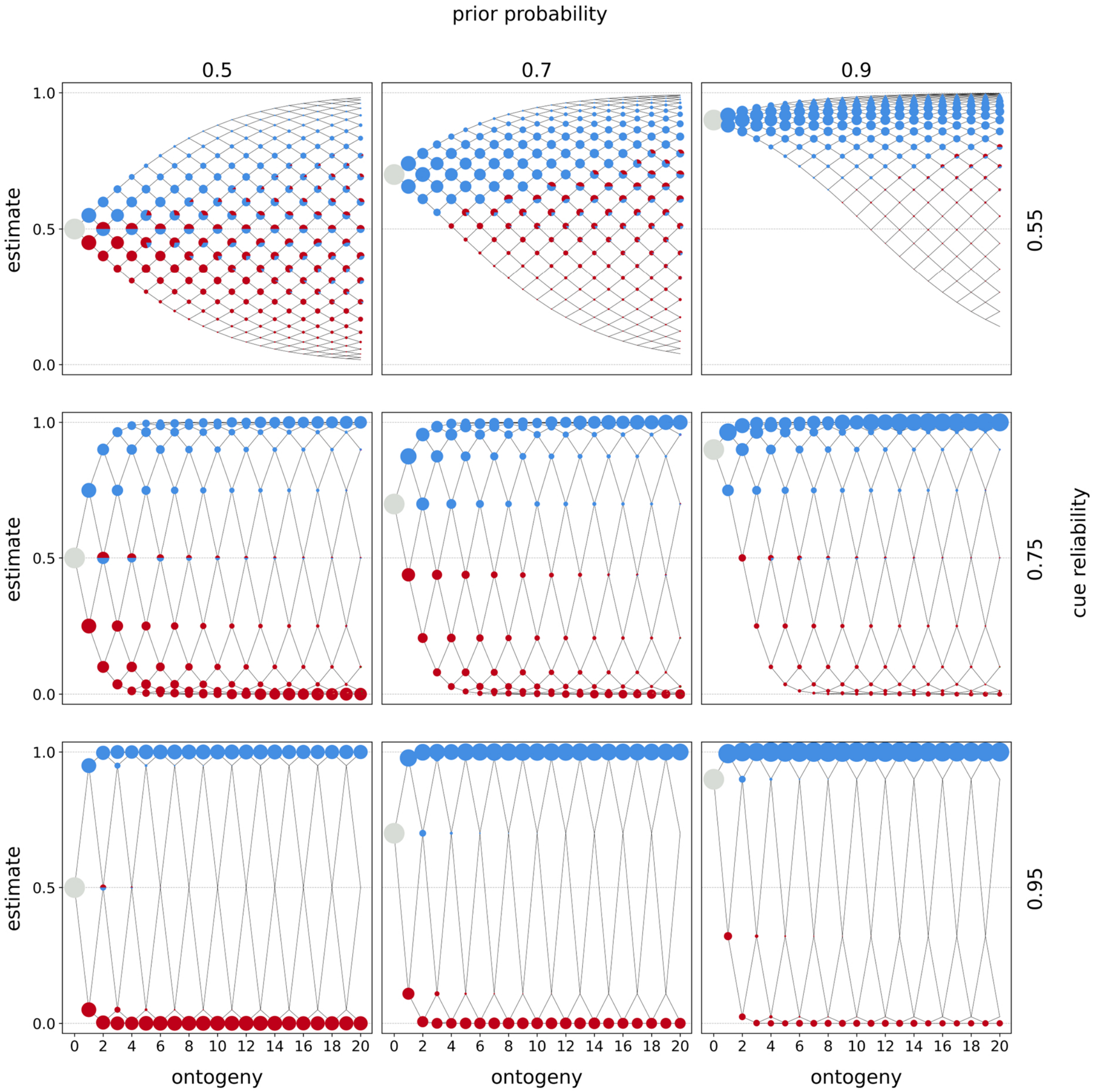

Figure S2.11: Optimal policies. Optimal policies are shown for a model with complete deconstruction and linear rewards and increasing penalties. Columns indicate the prior estimate of being in E_1 and rows indicate the cue reliability. Within each panel, the horizontal axis denotes ontogeny and the vertical axis the posterior estimates of being in E_1. The entire population starts ontogeny with zero cues sampled and the prior estimate indicated by the column (indicated by the grey circle). In each time period organisms sample a cue (either C_0 or C_1), update their estimate, and make a phenotypic decision (colored circles). Black lines indicate developmental trajectories through this decision space, with lines branching upwards indicating the sampling of C_1 and lines branching downwards indicating the sampling of C_o. Colors denote the optimal, fitness-maximizing phenotypic choice in each state. Pies indicate cases in which organisms with the same posterior estimates make different phenotypic decisions. The area of a circle (pie piece) is proportional the probability of reaching that particular state. Colors indicate the following phenotypic decisions: Black corresponds to waiting, red to constructing P_0, blue to constructing P_1, purple to

deconstructing P_0, green to deconstructing P_1, light red to a tie between constructing P_0 and deconstructing P_1, light blue to a tie between constructing P_1 and deconstructing P_0, brown to a tie between constructing either phenotypic target, yellow to a tie between deconstructing either target, grey to a tie between construction and waiting, dark grey to a tie between deconstruction and waiting, and lastly ochre to a tie between all options.

Linear rewards and diminishing penalties

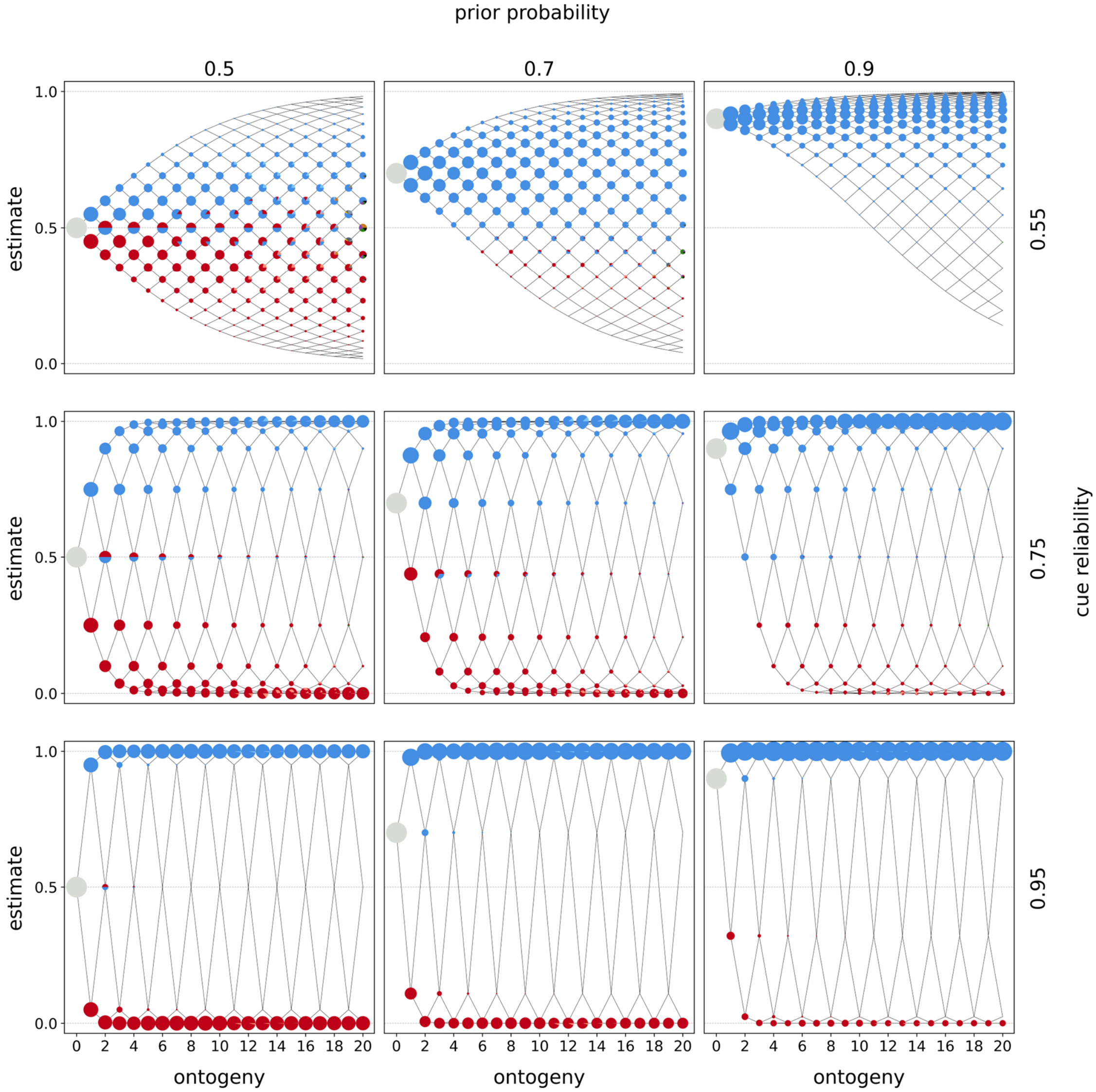

Figure S2.12: Optimal policies. Optimal policies are shown for a model with complete deconstruction and linear rewards and diminishing penalties. Columns indicate the prior estimate of being in E_1 and rows indicate the cue reliability. Within each panel, the horizontal axis denotes ontogeny and the vertical axis the posterior estimates of being in E_1. The entire population starts ontogeny with zero cues sampled and the prior estimate indicated by the column (indicated by the grey circle). In each time period organisms sample a cue (either C_0 or C_1), update their estimate, and make a phenotypic decision (colored circles). Black lines indicate developmental trajectories through this decision space, with lines branching upwards indicating the sampling of C_1 and lines branching downwards indicating the sampling of C_o. Colors denote the optimal, fitness-maximizing phenotypic choice in each state. Pies indicate cases in which organisms

with the same posterior estimates make different phenotypic decisions. The area of a circle (pie piece) is proportional the probability of reaching that particular state. Colors indicate the following phenotypic decisions: Black corresponds to waiting, red to constructing P_0, blue to constructing P_1, purple to deconstructing P_0, green to deconstructing P_1, light red to a tie between constructing P_0 and deconstructing P_1, light blue to a tie between constructing P_1 and deconstructing P_0, brown to a tie between constructing either phenotypic target, yellow to a tie between deconstructing either target, grey to a tie between construction and waiting, dark grey to a tie between deconstruction and waiting, and lastly ochre to a tie between all options.

Increasing rewards and linear penalties

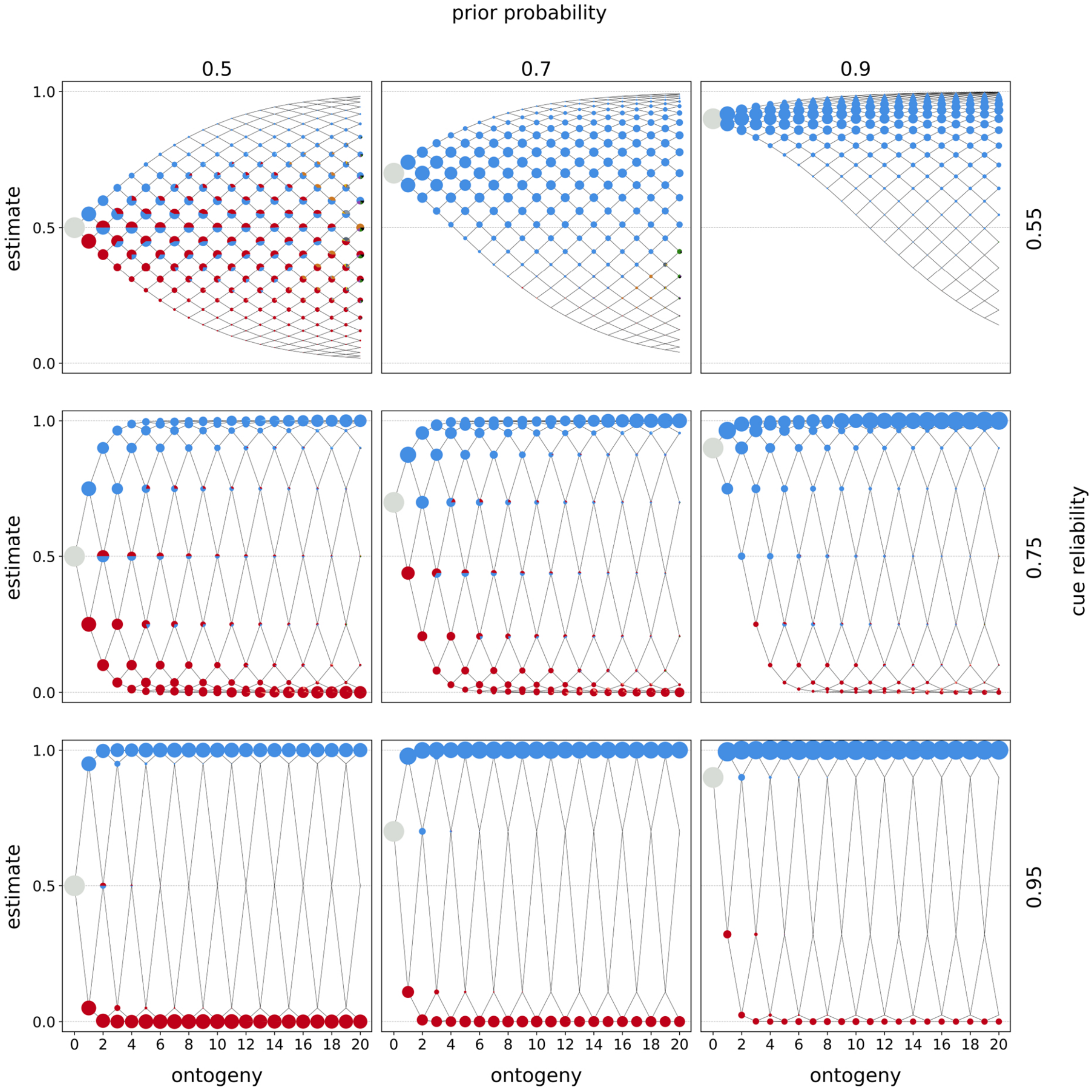

Figure S2.13: Optimal policies. Optimal policies are shown for a model with complete deconstruction and increasing rewards and linear penalties. Columns indicate the prior estimate of being in E_1 and rows indicate the cue reliability. Within each panel, the horizontal axis denotes ontogeny and the vertical axis the posterior estimates of being in E_1. The entire population starts ontogeny with zero cues sampled and the prior estimate indicated by the column (indicated by the grey circle). In each time period organisms sample a cue (either C_0 or C_1), update their estimate, and make a phenotypic decision (colored circles). Black lines indicate developmental trajectories through this decision space, with lines branching upwards indicating the sampling of C_1 and lines branching downwards indicating the sampling of C_o. Colors denote the optimal, fitness-maximizing phenotypic choice in each state. Pies indicate cases in which organisms with the same posterior estimates make different phenotypic decisions. The area of a circle (pie piece) is proportional the probability of reaching that particular state. Colors indicate the following phenotypic decisions: Black corresponds to waiting, red to constructing P_0, blue to constructing P_1, purple to deconstructing P_0, green to deconstructing P_1, light red to a tie between constructing P_0 and deconstructing P_1, light blue to a tie between constructing P_1 and deconstructing P_0, brown to a tie between constructing either phenotypic target, yellow to a tie between deconstructing either target, grey to a tie between construction and waiting, dark grey to a tie between deconstruction and waiting, and lastly ochre to a tie between all options.

Increasing rewards and increasing penalties

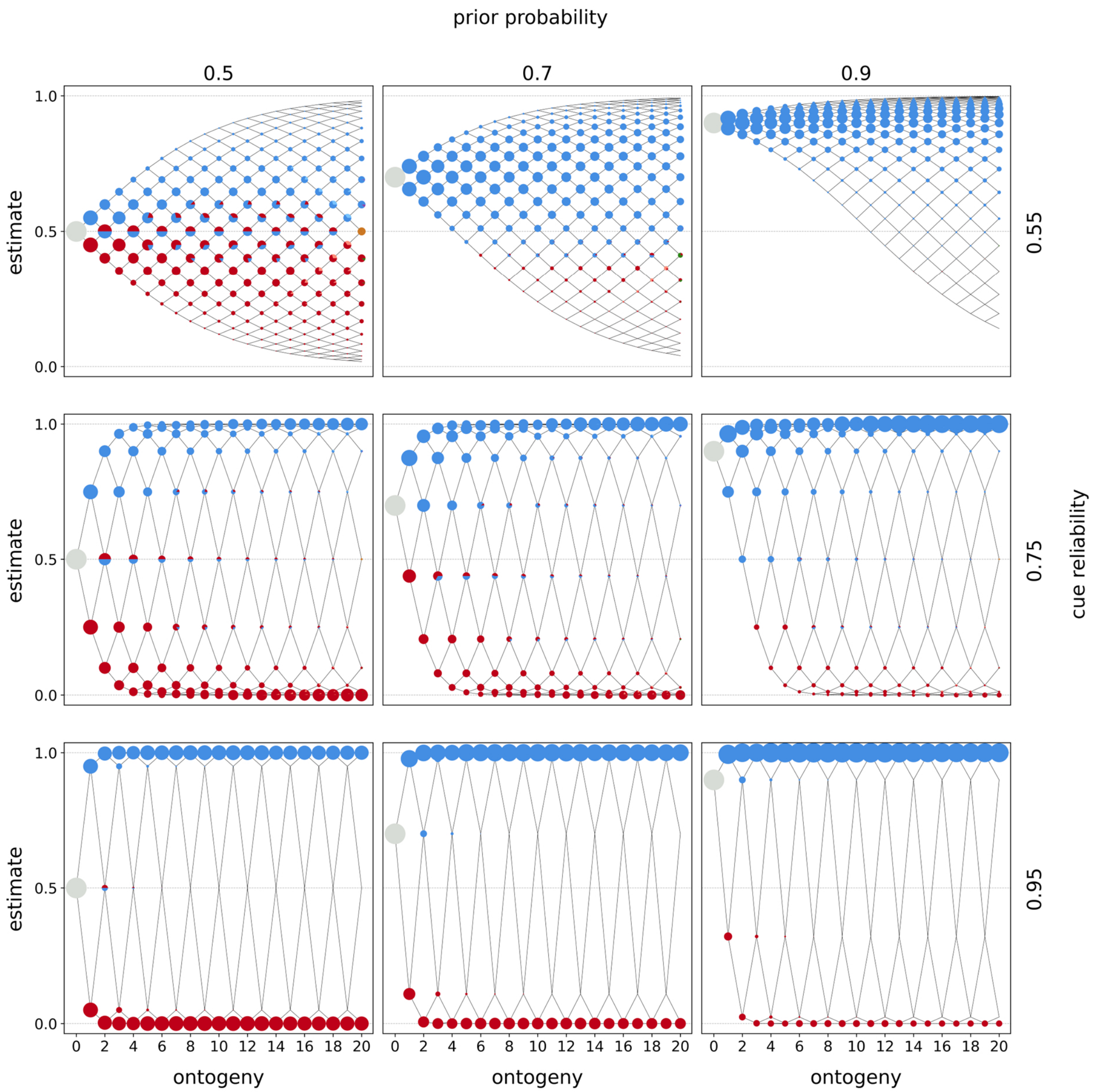

Figure S2.14: Optimal policies. Optimal policies are shown for a model with complete deconstruction and increasing rewards and increasing penalties. Columns indicate the prior estimate of being in E_1 and rows indicate the cue reliability. Within each panel, the horizontal axis denotes ontogeny and the vertical axis the posterior estimates of being in E_1. The entire population starts ontogeny with zero cues sampled and the prior estimate indicated by the column (indicated by the grey circle). In each time period organisms sample a cue (either C_0 or C_1), update their estimate, and make a phenotypic decision (colored circles). Black lines indicate developmental trajectories through this decision space, with lines branching upwards indicating the sampling of C_1 and lines branching downwards indicating the sampling of C_o. Colors denote the optimal, fitness-maximizing phenotypic choice in each state. Pies indicate cases in which organisms with the same posterior estimates make different phenotypic decisions. The area of a circle (pie piece) is proportional the probability of reaching that particular state. Colors indicate the following phenotypic decisions: Black corresponds to waiting, red to constructing P_0, blue to constructing P_1, purple to deconstructing P_0, green to deconstructing P_1, light red to a tie between constructing P_0 and deconstructing P_1, light blue to a tie between constructing P_1 and deconstructing P_0, brown to a tie between constructing either phenotypic target, yellow to a tie between deconstructing either target, grey to a tie between

construction and waiting, dark grey to a tie between deconstruction and waiting, and lastly ochre to a tie between all options.

Increasing rewards and diminishing penalties

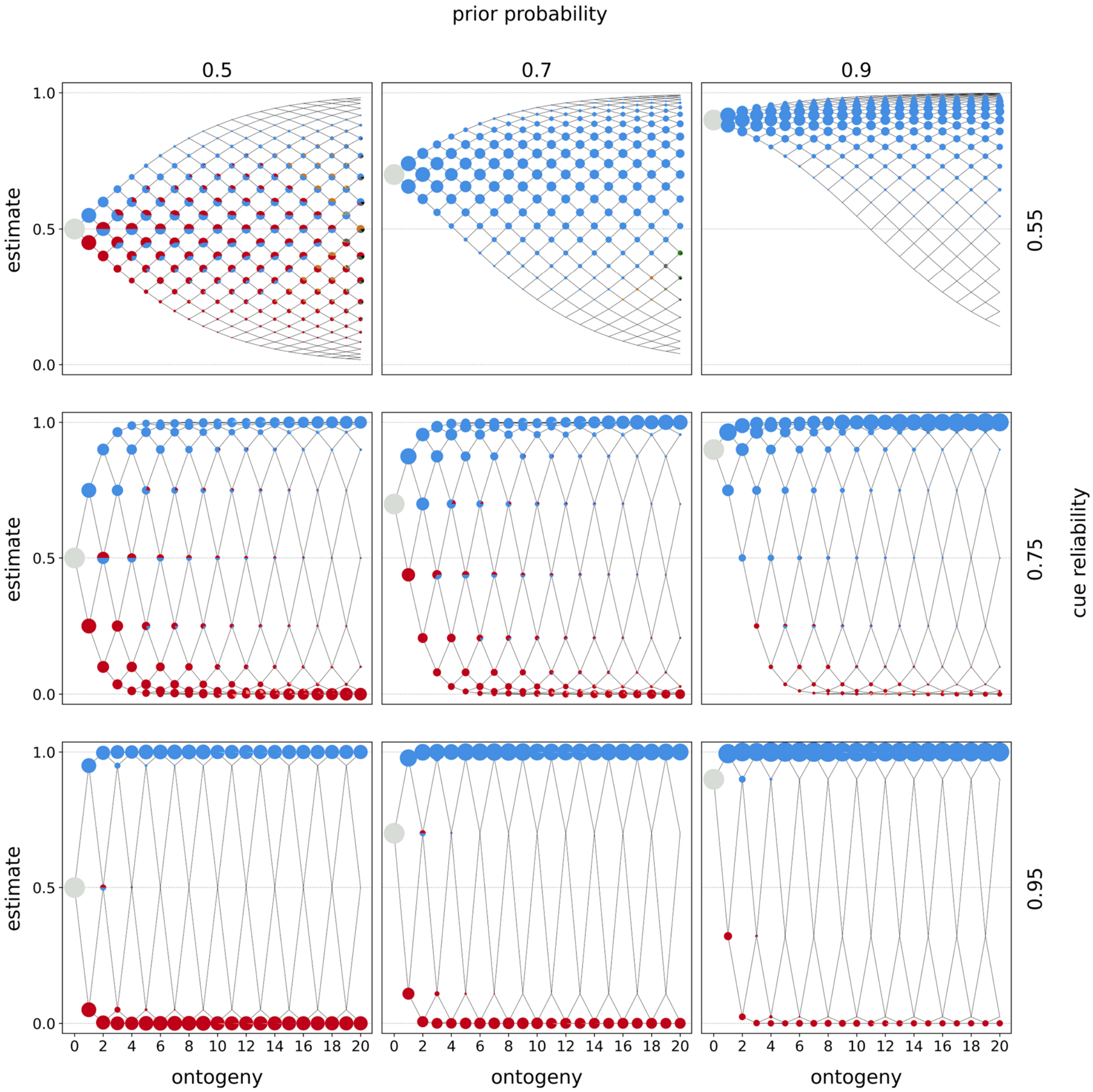

Figure S2.15: Optimal policies. Optimal policies are shown for a model with complete deconstruction and increasing rewards and diminishing penalties. Columns indicate the prior estimate of being in E_1 and rows indicate the cue reliability. Within each panel, the horizontal axis denotes ontogeny and the vertical axis the posterior estimates of being in E_1. The entire population starts ontogeny with zero cues sampled and the prior estimate indicated by the column (indicated by the grey circle). In each time period organisms sample a cue (either C_0 or C_1), update their estimate, and make a phenotypic decision (colored circles). Black lines indicate developmental trajectories through this decision space, with lines branching upwards indicating the sampling of C_1 and lines branching downwards indicating the sampling of C_o. Colors denote the optimal, fitness-maximizing phenotypic choice in each state. Pies indicate cases in which organisms with the same posterior estimates make different phenotypic decisions. The area of a circle (pie piece) is proportional the probability of reaching that particular state. Colors indicate the following phenotypic decisions: Black corresponds to waiting, red to constructing P_0, blue to constructing P_1, purple to

deconstructing P_0, green to deconstructing P_1, light red to a tie between constructing P_0 and deconstructing P_1, light blue to a tie between constructing P_1 and deconstructing P_0, brown to a tie between constructing either phenotypic target, yellow to a tie between deconstructing either target, grey to a tie between construction and waiting, dark grey to a tie between deconstruction and waiting, and lastly ochre to a tie between all options.

Diminishing rewards and linear penalties

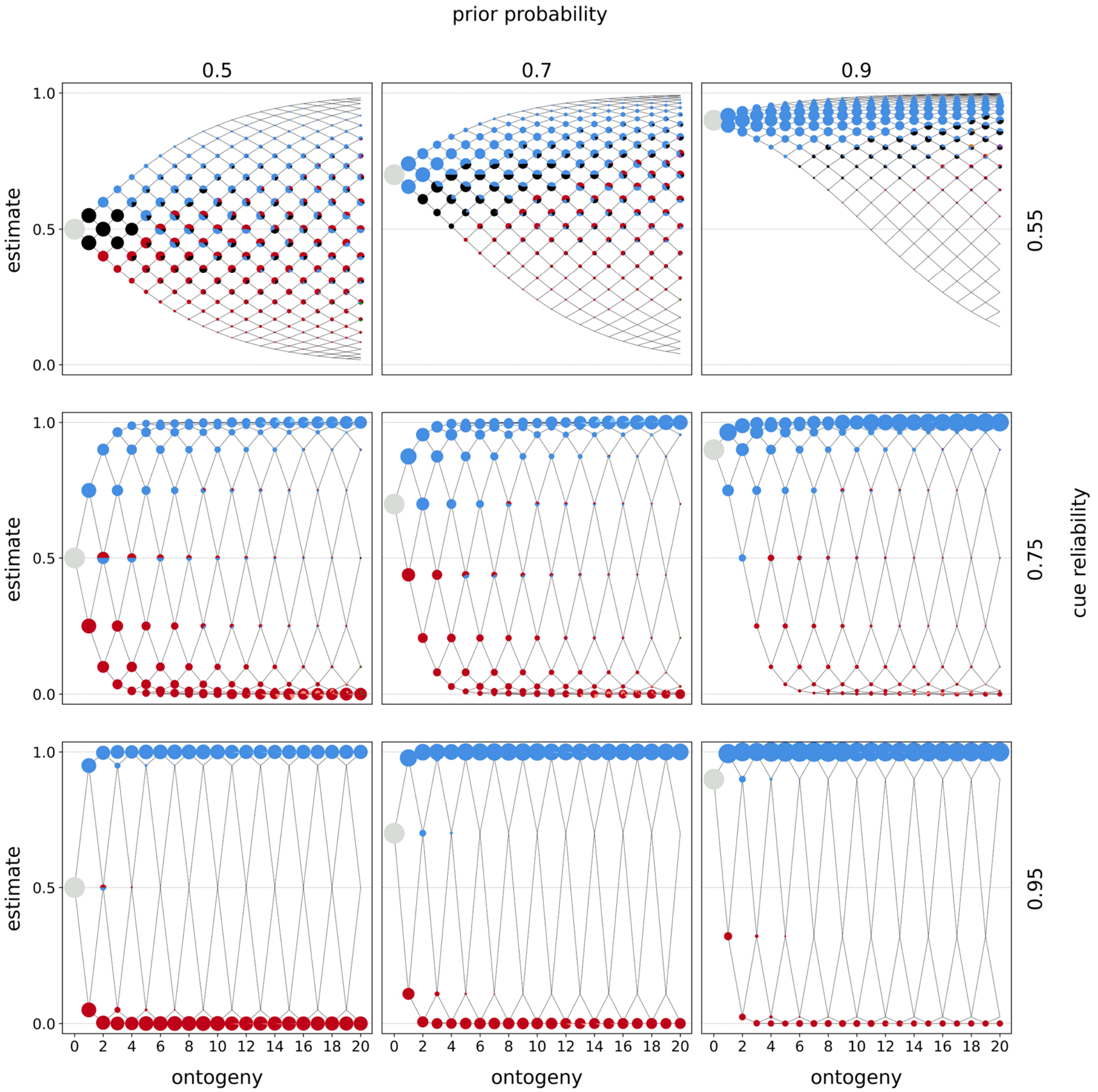

Figure S2.16: Optimal policies. Optimal policies are shown for a model with complete deconstruction and diminishing rewards and linear penalties. Columns indicate the prior estimate of being in E_1 and rows indicate the cue reliability. Within each panel, the horizontal axis denotes ontogeny and the vertical axis the posterior estimates of being in E_1. The entire population starts ontogeny with zero cues sampled and the prior estimate indicated by the column (indicated by the grey circle). In each time period organisms sample a cue (either C_0 or C_1), update their estimate, and make a phenotypic decision (colored circles). Black lines indicate developmental trajectories through this decision space, with lines branching upwards indicating the sampling of C_1 and lines branching downwards indicating the sampling of C_o. Colors denote the optimal, fitness-maximizing phenotypic choice in each state. Pies indicate cases in which organisms with the same posterior estimates make different phenotypic decisions. The area of a circle (pie piece) is proportional the probability of reaching that particular state. Colors indicate the following phenotypic decisions: Black corresponds to waiting, red to constructing P_0, blue to constructing P_1, purple to deconstructing P_0, green to deconstructing P_1, light red to a tie between constructing P_0 and deconstructing P_1, light blue to a tie between constructing P_1 and deconstructing P_0, brown to a tie between constructing either phenotypic target, yellow to a tie between deconstructing either target, grey to a tie between construction and waiting, dark grey to a tie between deconstruction and waiting, and lastly ochre to a tie between all options.

Diminishing rewards and increasing penalties

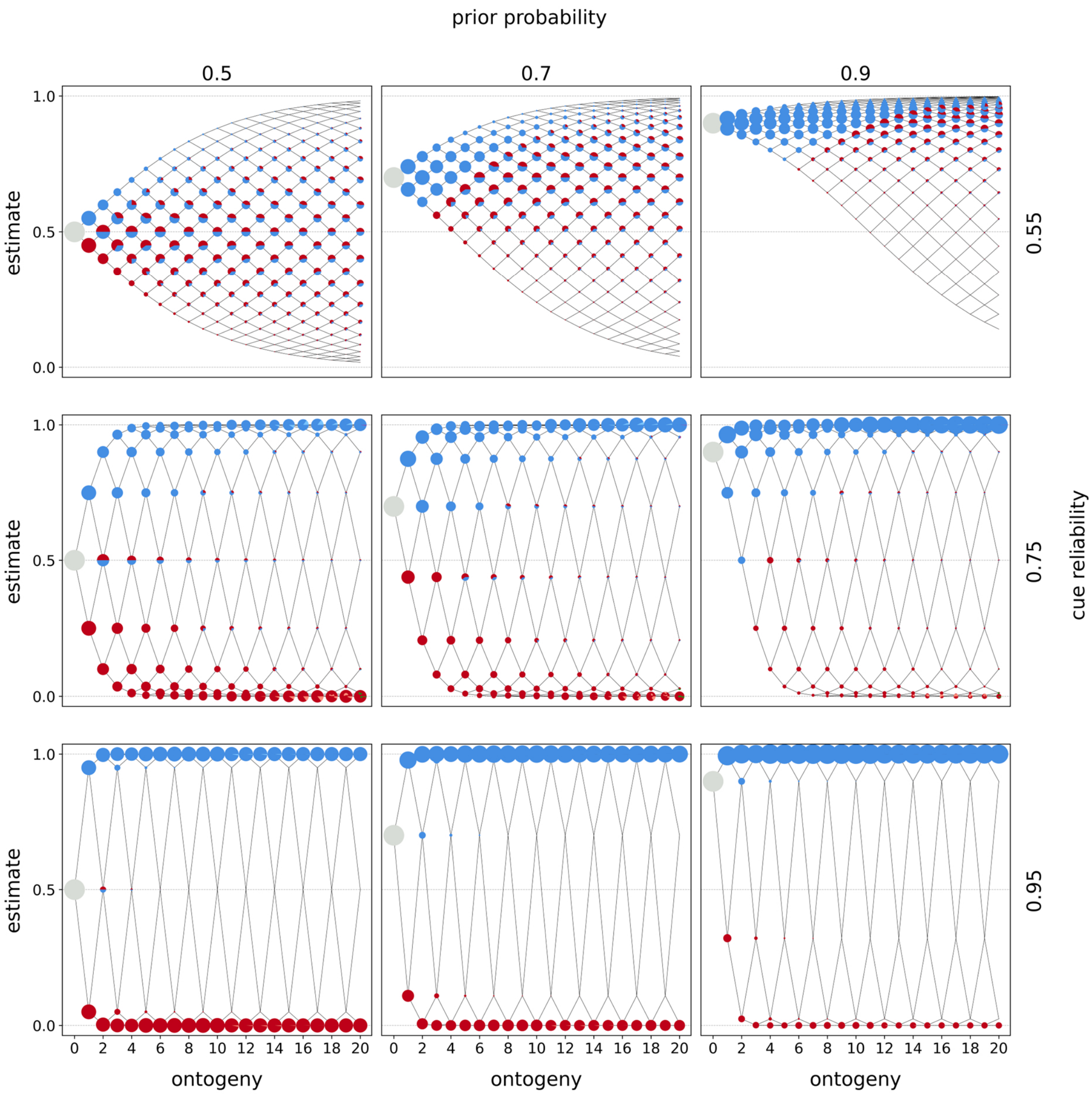

Figure S2.17: Optimal policies. Optimal policies are shown for a model with complete deconstruction and diminishing rewards and increasing penalties. Columns indicate the prior estimate of being in E_1 and rows indicate the cue reliability. Within each panel, the horizontal axis denotes ontogeny and the vertical axis the posterior estimates of being in E_1. The entire population starts ontogeny with zero cues sampled and the prior estimate indicated by the column (indicated by the grey circle). In each time period organisms sample a cue (either C_0 or C_1), update their estimate, and make a phenotypic decision (colored circles). Black lines indicate developmental trajectories through this decision space, with lines branching upwards indicating the sampling of C_1 and lines branching downwards indicating the sampling of C_o. Colors denote the optimal, fitness-maximizing phenotypic choice in each state. Pies indicate cases in which organisms with the same posterior estimates make different phenotypic decisions. The area of a circle (pie piece) is proportional the probability of reaching that particular state. Colors indicate the following phenotypic decisions: Black corresponds to waiting, red to constructing P_0, blue to constructing P_1, purple to deconstructing P_0, green to deconstructing P_1, light red to a tie between constructing P_0 and deconstructing P_1, light blue to a tie between constructing P_1 and deconstructing P_0, brown to a tie between constructing either phenotypic target, yellow to a tie between deconstructing either target, grey to a tie between

construction and waiting, dark grey to a tie between deconstruction and waiting, and lastly ochre to a tie between all options.

Diminishing rewards and diminishing penalties

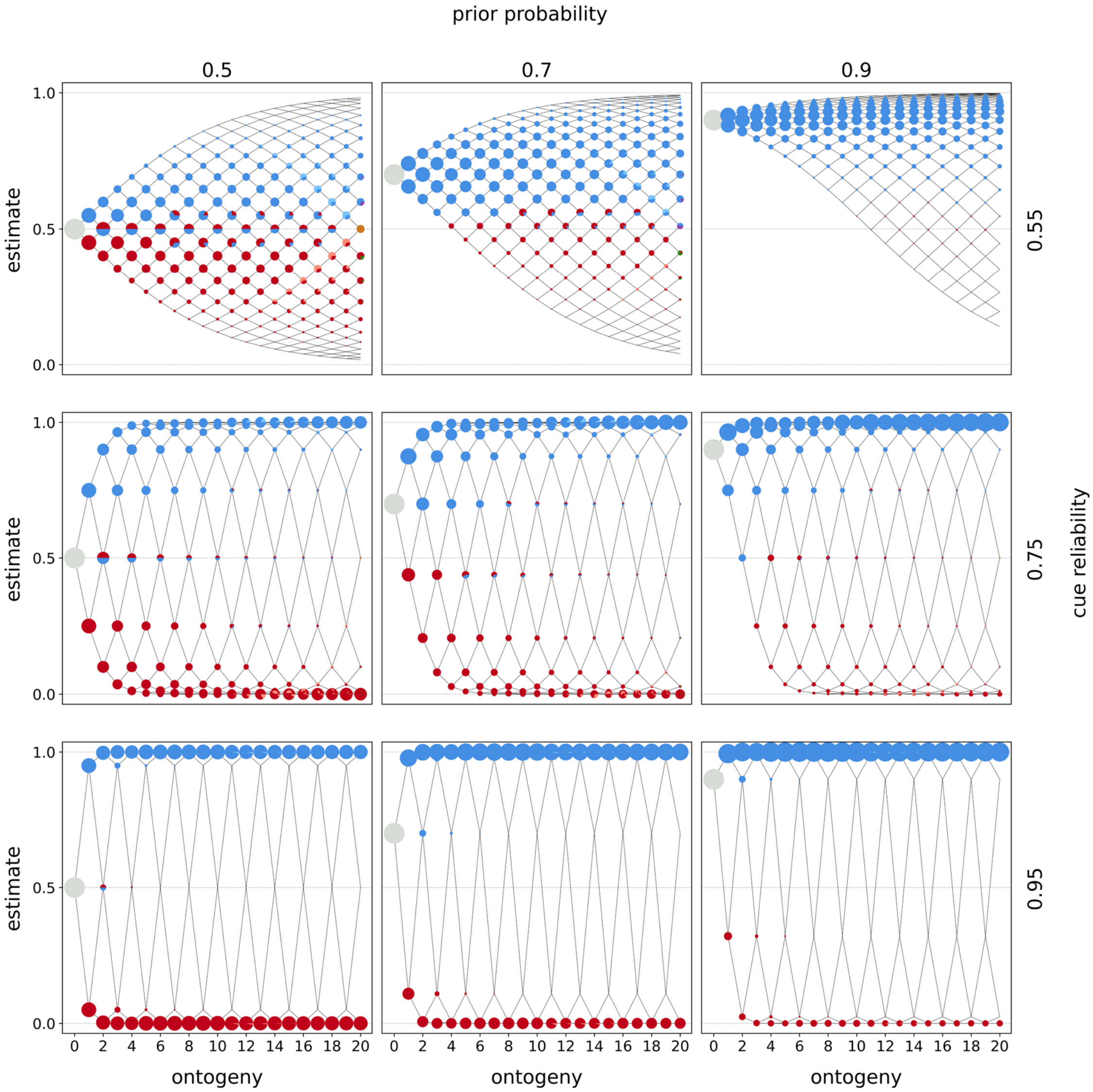

Figure S2.18: Optimal policies. Optimal policies are shown for a model with complete deconstruction and diminishing rewards and diminishing penalties. Columns indicate the prior estimate of being in E_1 and rows indicate the cue reliability. Within each panel, the horizontal axis denotes ontogeny and the vertical axis the posterior estimates of being in E_1. The entire population starts ontogeny with zero cues sampled and the prior estimate indicated by the column (indicated by the grey circle). In each time period organisms sample a cue (either C_0 or C_1), update their estimate, and make a phenotypic decision (colored circles). Black lines indicate developmental trajectories through this decision space, with lines branching upwards indicating the sampling of C_1 and lines branching downwards indicating the sampling of C_o. Colors denote the optimal, fitness-maximizing phenotypic choice in each state. Pies indicate cases in which organisms with the same posterior estimates make different phenotypic decisions. The area of a circle (pie piece) is proportional the probability of reaching that particular state. Colors indicate the following phenotypic decisions: Black corresponds to waiting, red to constructing P_0, blue to constructing P_1, purple to

deconstructing P_0, green to deconstructing P_1, light red to a tie between constructing P_0 and deconstructing P_1, light blue to a tie between constructing P_1 and deconstructing P_0, brown to a tie between constructing either phenotypic target, yellow to a tie between deconstructing either target, grey to a tie between construction and waiting, dark grey to a tie between deconstruction and waiting, and lastly ochre to a tie between all options.

Triangular distributions of mature phenotypes (incremental deconstruction)

Linear rewards and linear penalties

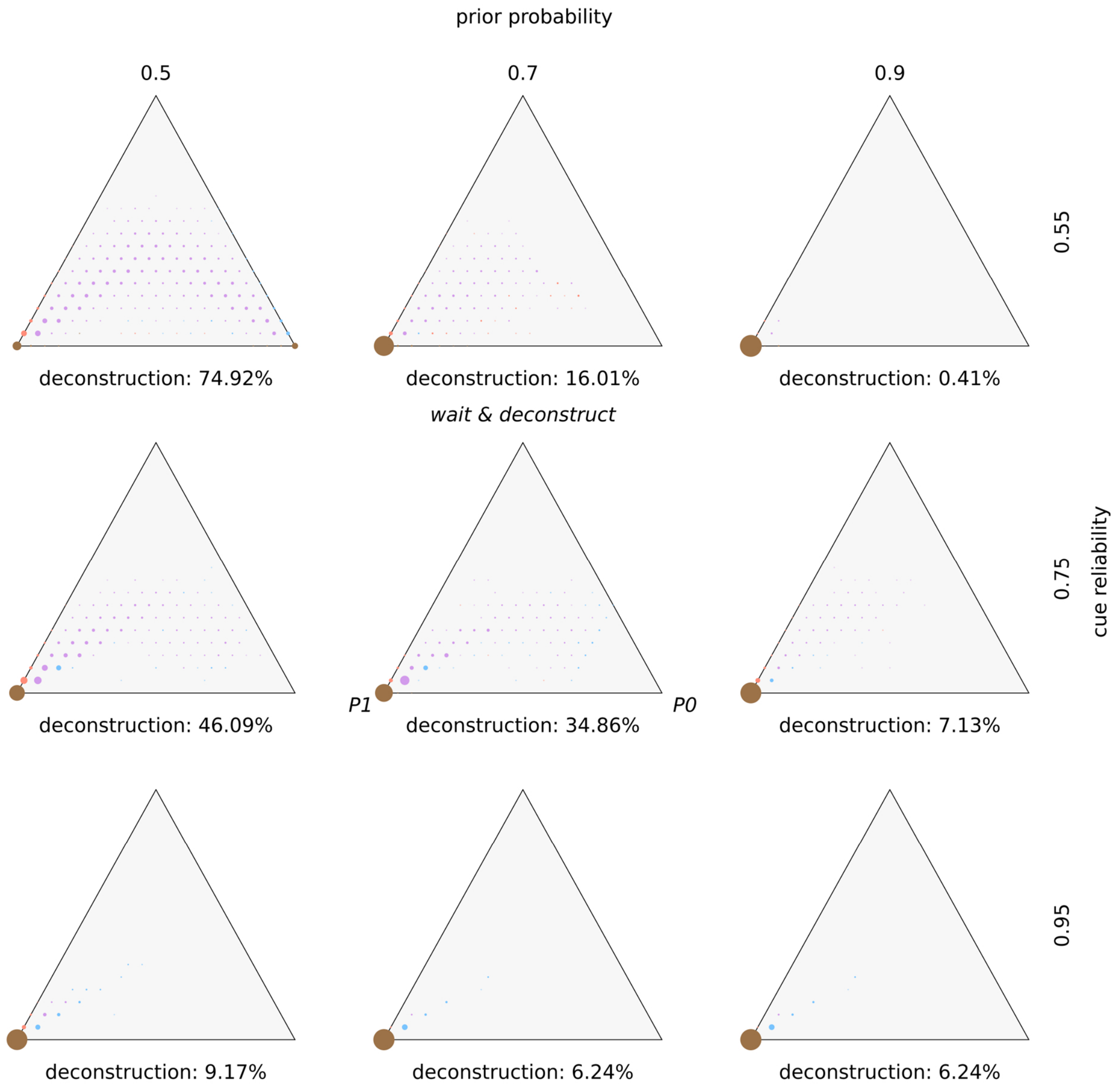

Figure S2.19: Distributions of mature phenotypes (incremental deconstruction, linear rewards and penalties). Columns indicate the prior estimate of being in E_1 and rows indicate the cue reliability. The populations of mature phenotypes have been simulated in E_1. The horizontal label of each panel indicates the percentage of mature phenotypes that have deconstructed at some point during ontogeny. Each triangle depicts the distribution of mature phenotypes. The number of time periods spent specializing towards P_1 and P_0, the number of time periods spent deconstructing P_1 and P_0, and the number of time periods spent waiting make up a phenotype. A circle's position indicates the phenotypic composition: The left and right vertices correspond to organisms who fully specialized towards P_1 or P_0. The top vertex corresponds to organisms who only waited and/or deconstructed. Circles on the outer boundary indicate mixtures between two of the vertices and circles inside indicate mixtures of all three. The area of a circles is proportional to number of mature organisms with that phenotype. Colors differentiate between phenotypic decisions to deconstruct: brown indicates no deconstruction, light red indicates deconstruction of P_1, light blue indicates deconstruction of P_0, and light purple indicates organisms who deconstructed both P_1 and P_0.

Linear rewards and increasing penalties

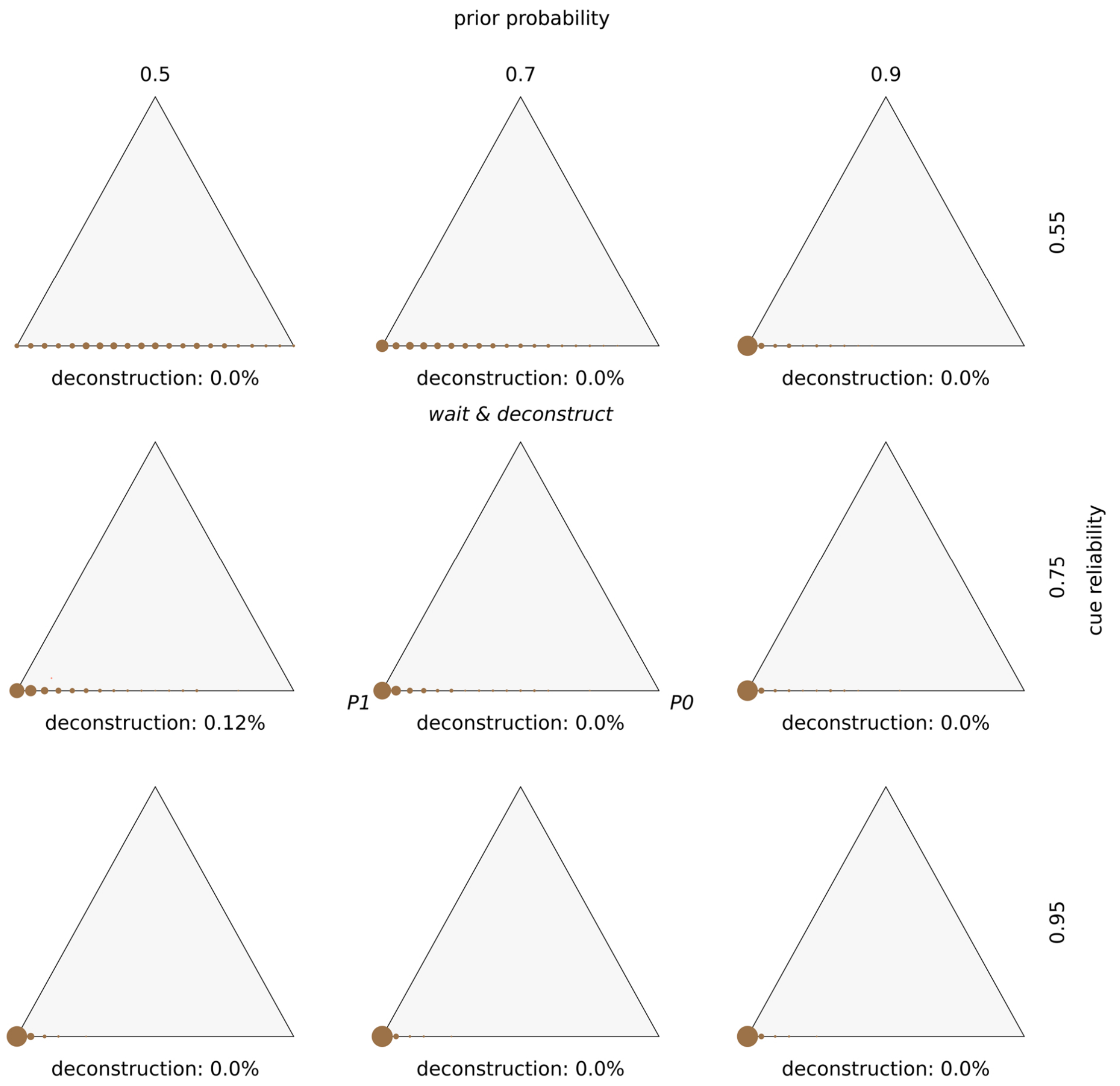

Figure S2.20: Distributions of mature phenotypes (incremental deconstruction, linear rewards and increasing penalties). Columns indicate the prior estimate of being in E_1 and rows indicate the cue reliability. The populations of mature phenotypes have been simulated in E_1. The horizontal label of each panel indicates the percentage of mature phenotypes that have deconstructed at some point during ontogeny. Each triangle depicts the distribution of mature phenotypes. The number of time periods spent specializing towards P_1 and P_0, the number of time periods spent deconstructing P_1 and P_0, and the number of time periods spent waiting make up a phenotype. A circle's position indicates the phenotypic composition: The left and right vertices correspond to organisms who fully specialized towards P_1 or P_0. The top vertex corresponds to organisms who only waited and/or deconstructed. Circles on the outer boundary indicate mixtures between two of the vertices and circles inside indicate mixtures of all three. The area of a circles is proportional to number of mature organisms with that phenotype. Colors differentiate between phenotypic decisions to deconstruct: brown indicates no deconstruction, light red indicates deconstruction of P_1, light blue indicates deconstruction of P_0, and light purple indicates organisms who deconstructed both P_1 and P_0.

Linear rewards and diminishing penalties

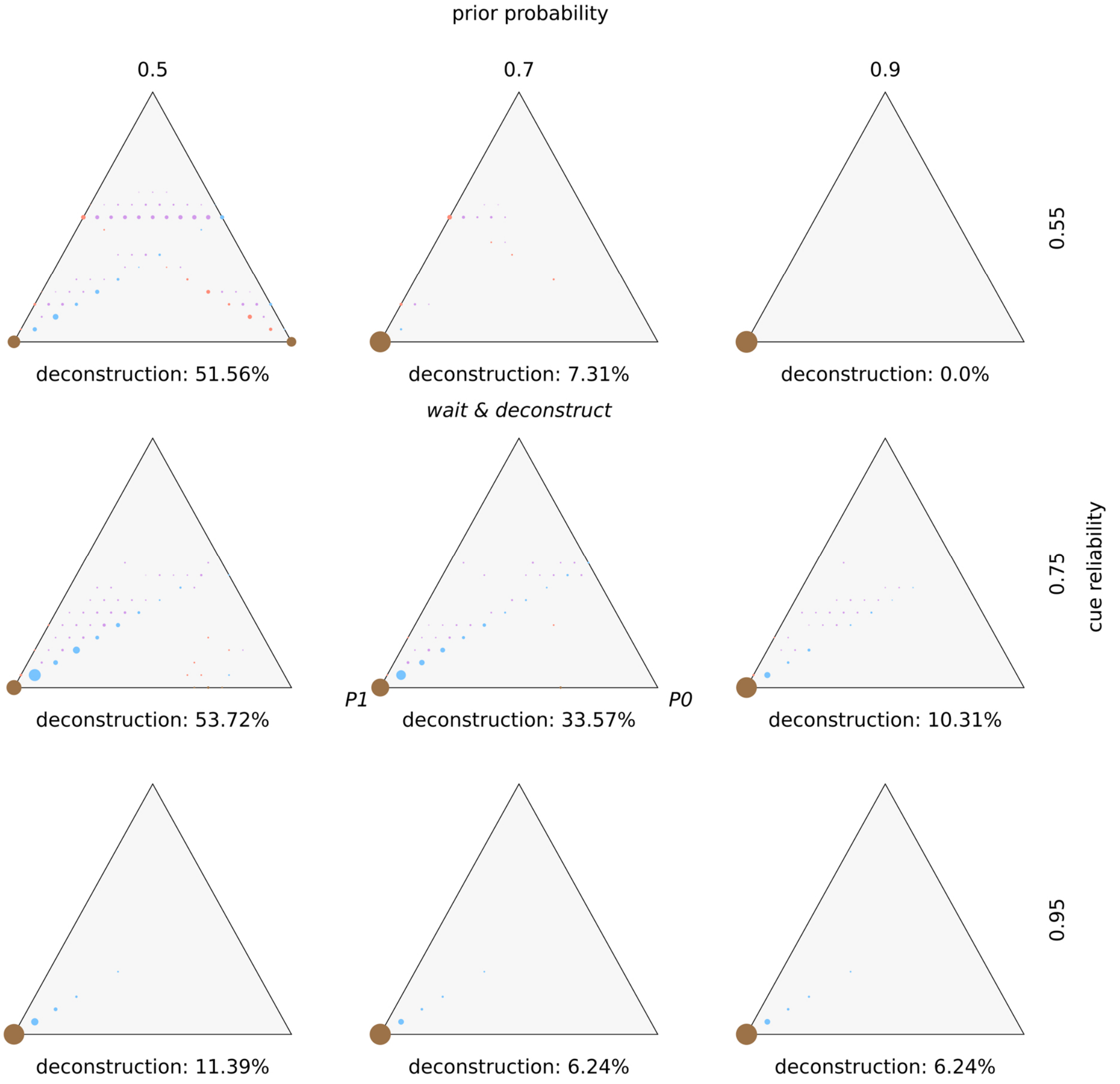

Figure S2.21: Distributions of mature phenotypes (incremental deconstruction, linear rewards and diminishing penalties). Columns indicate the prior estimate of being in E_1 and rows indicate the cue reliability. The populations of mature phenotypes have been simulated in E_1. The horizontal label of each panel indicates the percentage of mature phenotypes that have deconstructed at some point during ontogeny. Each triangle depicts the distribution of mature phenotypes. The number of time periods spent specializing towards P_1 and P_0, the number of time periods spent deconstructing P_1 and P_0, and the number of time periods spent waiting make up a phenotype. A circle's position indicates the phenotypic composition: The left and right vertices correspond to organisms who fully specialized towards P_1 or P_0. The top vertex corresponds to organisms who only waited and/or deconstructed. Circles on the outer boundary indicate mixtures between two of the vertices and circles inside indicate mixtures of all three. The area of a circles is proportional to number of mature organisms with that phenotype. Colors differentiate between phenotypic decisions to deconstruct: brown indicates no deconstruction, light red indicates deconstruction of P_1, light blue indicates deconstruction of P_0, and light purple indicates organisms who deconstructed both P_1 and P_0.

Increasing rewards and linear penalties

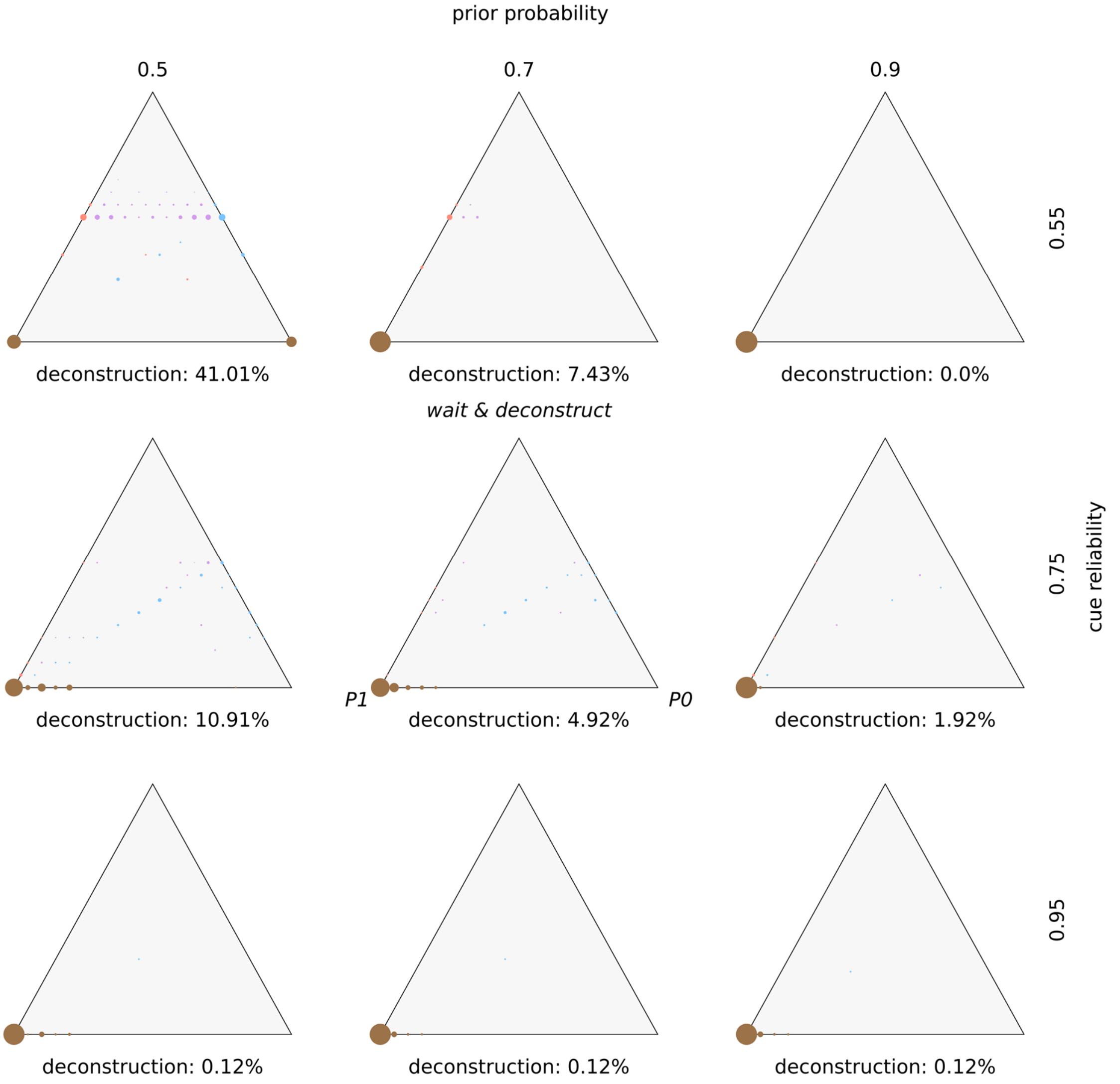

Figure S2.22: Distributions of mature phenotypes (incremental deconstruction, increasing rewards and linear penalties). Columns indicate the prior estimate of being in E_1 and rows indicate the cue reliability. The populations of mature phenotypes have been simulated in E_1. The horizontal label of each panel indicates the percentage of mature phenotypes that have deconstructed at some point during ontogeny. Each triangle depicts the distribution of mature phenotypes. The number of time periods spent specializing towards P_1 and P_0, the number of time periods spent deconstructing P_1 and P_0, and the number of time periods spent waiting make up a phenotype. A circle's position indicates the phenotypic composition: The left and right vertices correspond to organisms who fully specialized towards P_1 or P_0. The top vertex corresponds to organisms who only waited and/or deconstructed. Circles on the outer boundary indicate mixtures between two of the vertices and circles inside indicate mixtures of all three. The area of a circles is proportional to number of mature organisms with that phenotype. Colors differentiate between phenotypic decisions to deconstruct: brown indicates no deconstruction, light red indicates deconstruction of P_1, light blue indicates deconstruction of P_0, and light purple indicates organisms who deconstructed both P_1 and P_0.

Increasing rewards and increasing penalties

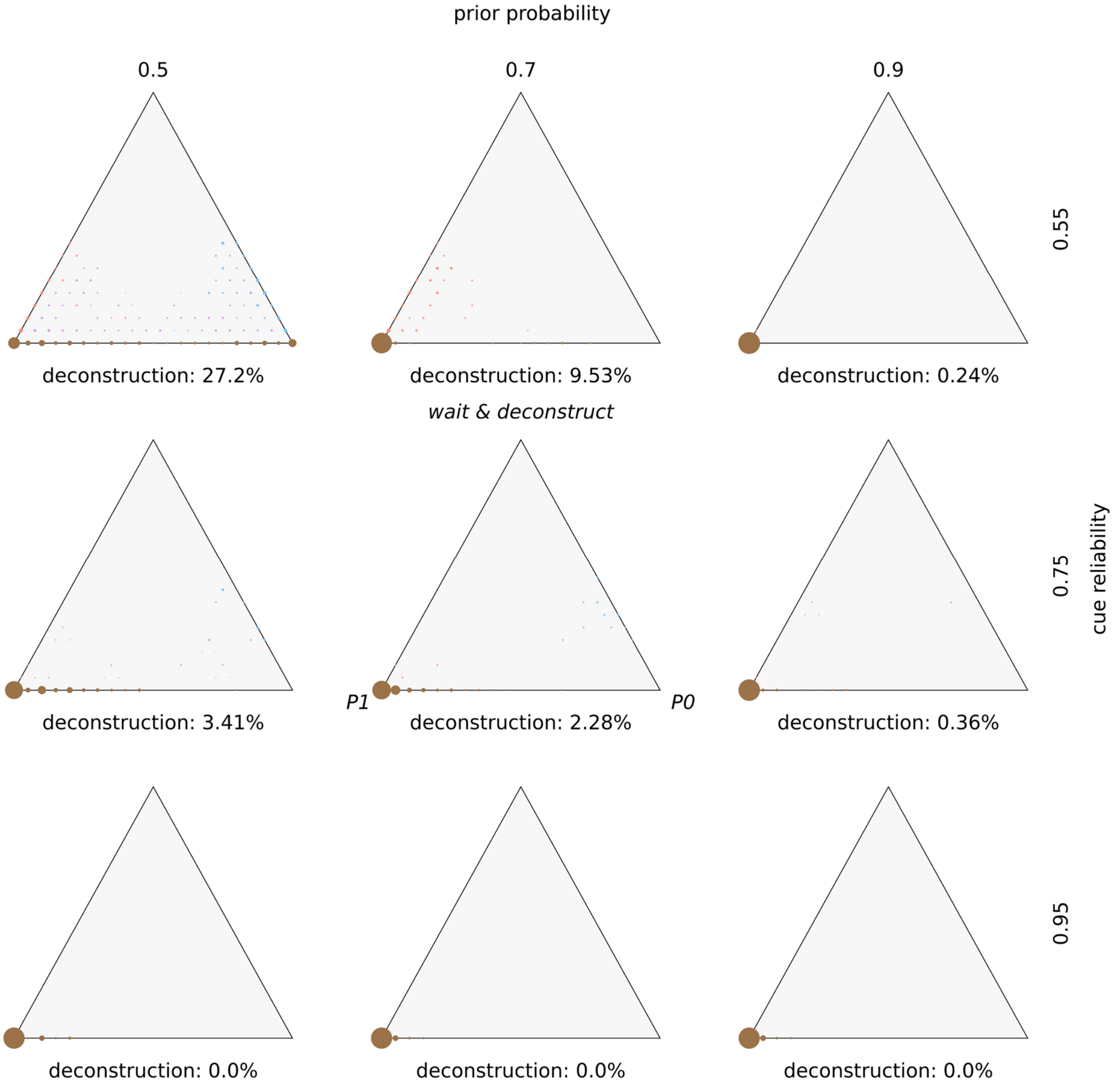

Figure S2.23: Distributions of mature phenotypes (incremental deconstruction, increasing rewards and increasing penalties). Columns indicate the prior estimate of being in E_1 and rows indicate the cue reliability. The populations of mature phenotypes have been simulated in E_1. The horizontal label of each panel indicates the percentage of mature phenotypes that have deconstructed at some point during ontogeny. Each triangle depicts the distribution of mature phenotypes. The number of time periods spent specializing towards P_1 and P_0, the number of time periods spent deconstructing P_1 and P_0, and the number of time periods spent waiting make up a phenotype. A circle's position indicates the phenotypic composition: The left and right vertices correspond to organisms who fully specialized towards P_1 or P_0. The top vertex corresponds to organisms who only waited and/or deconstructed. Circles on the outer boundary indicate mixtures between two of the vertices and circles inside indicate mixtures of all three. The area of a circles is proportional to number of mature organisms with that phenotype. Colors differentiate between phenotypic decisions to deconstruct: brown indicates no deconstruction, light red indicates deconstruction of P_1, light blue indicates deconstruction of P_0, and light purple indicates organisms who deconstructed both P_1 and P_0.

Increasing rewards and diminishing penalties

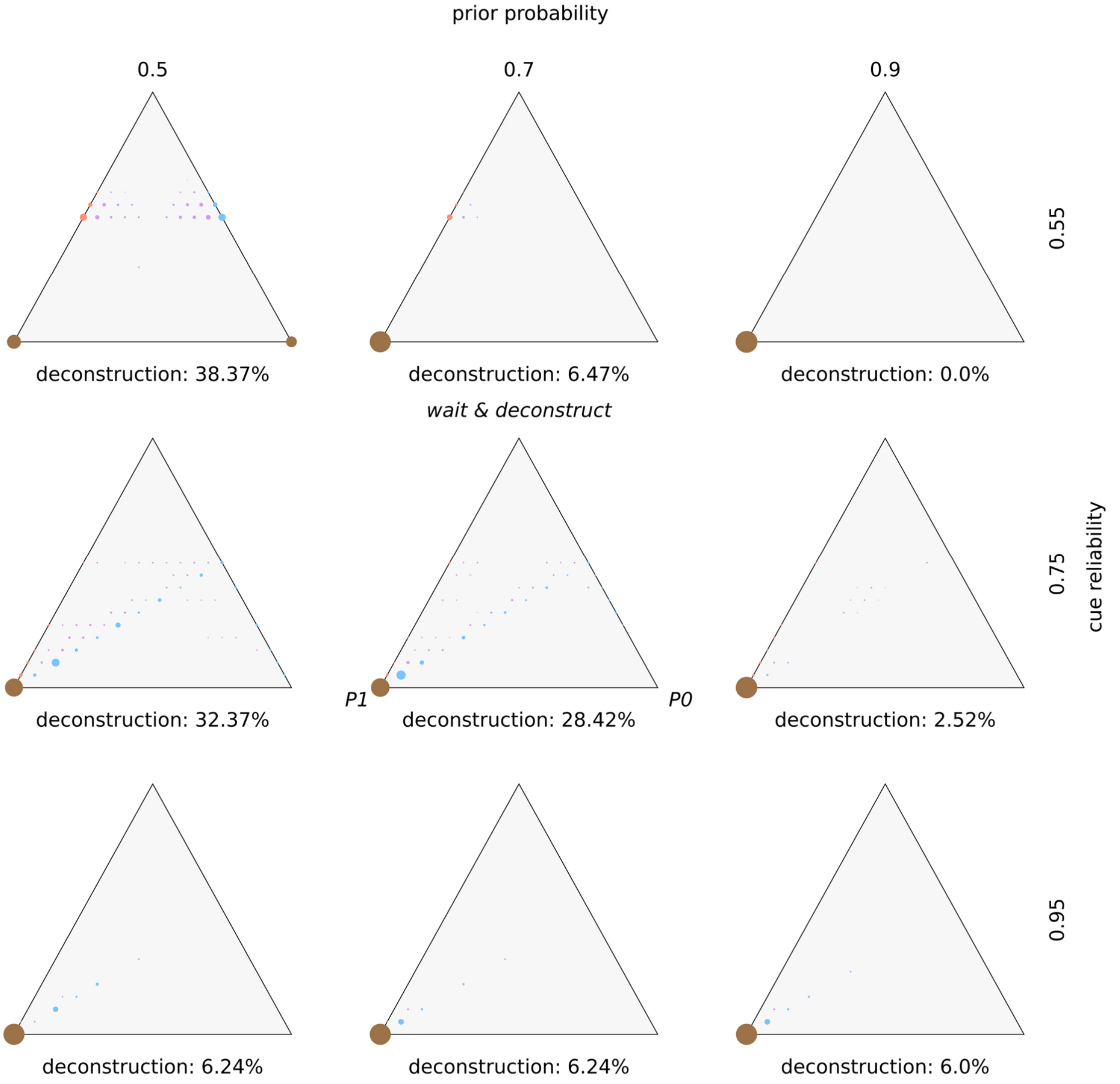

Figure S2.24: Distributions of mature phenotypes (incremental deconstruction, increasing rewards and diminishing penalties). Columns indicate the prior estimate of being in E_1 and rows indicate the cue reliability. The populations of mature phenotypes have been simulated in E_1. The horizontal label of each panel indicates the percentage of mature phenotypes that have deconstructed at some point during ontogeny. Each triangle depicts the distribution of mature phenotypes. The number of time periods spent specializing towards P_1 and P_0, the number of time periods spent deconstructing P_1 and P_0, and the number of time periods spent waiting make up a phenotype. A circle's position indicates the phenotypic composition: The left and right vertices correspond to organisms who fully specialized towards P_1 or P_0. The top vertex corresponds to organisms who only waited and/or deconstructed. Circles on the outer boundary indicate mixtures between two of the vertices and circles inside indicate mixtures of all three. The area of a circles is proportional to number of mature organisms with that phenotype. Colors differentiate between phenotypic decisions to deconstruct: brown indicates no deconstruction, light red indicates deconstruction of P_1, light blue indicates deconstruction of P_0, and light purple indicates organisms who deconstructed both P_1 and P_0.

Diminishing rewards and linear penalties

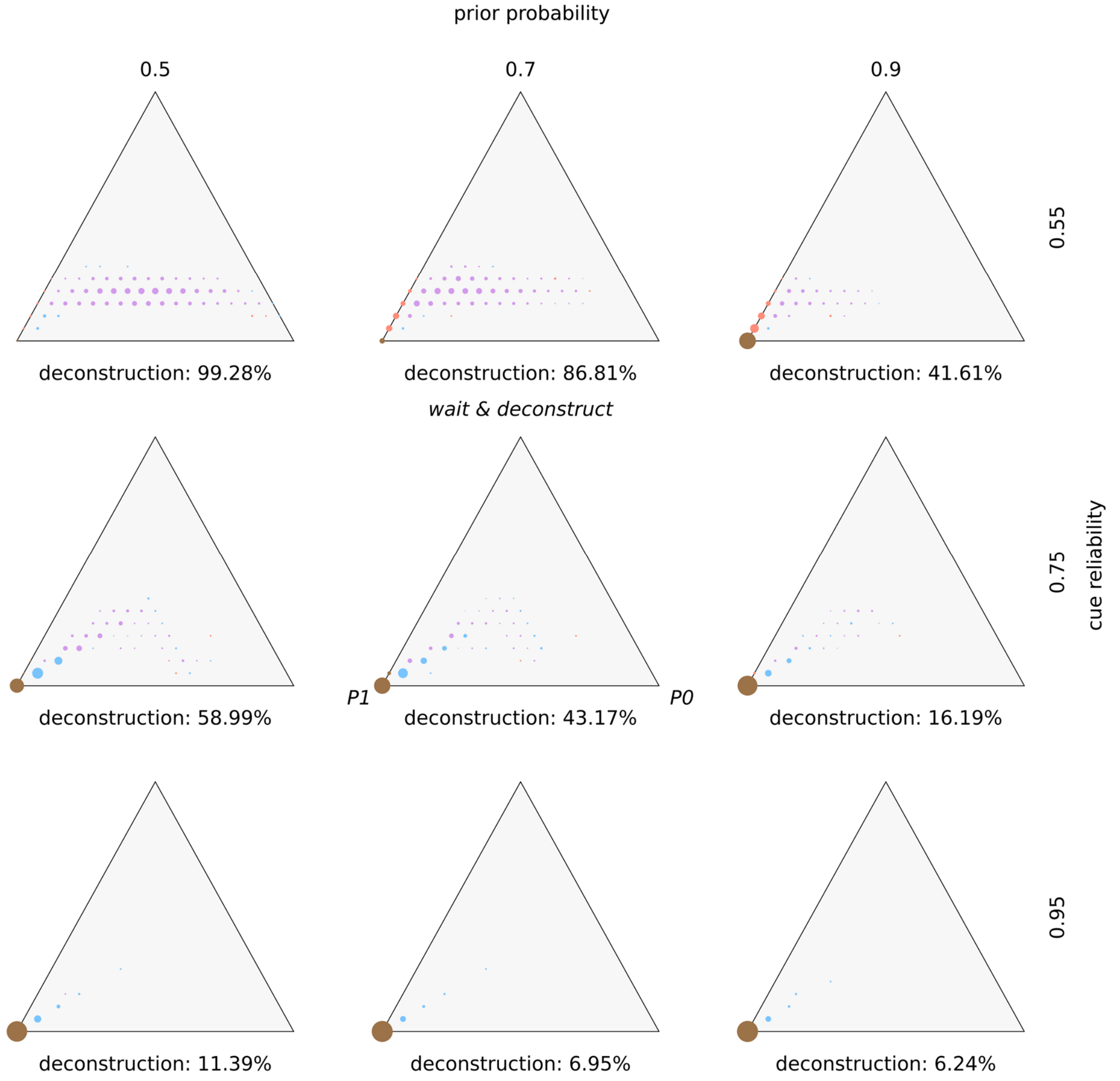

Figure S2.25: Distributions of mature phenotypes (incremental deconstruction, diminishing rewards and linear penalties). Columns indicate the prior estimate of being in E_1 and rows indicate the cue reliability. The populations of mature phenotypes have been simulated in E_1. The horizontal label of each panel indicates the percentage of mature phenotypes that have deconstructed at some point during ontogeny. Each triangle depicts the distribution of mature phenotypes. The number of time periods spent specializing towards P_1 and P_0, the number of time periods spent deconstructing P_1 and P_0, and the number of time periods spent waiting make up a phenotype. A circle's position indicates the phenotypic composition: The left and right vertices correspond to organisms who fully specialized towards P_1 or P_0. The top vertex corresponds to organisms who only waited and/or deconstructed. Circles on the outer boundary indicate mixtures between two of the vertices and circles inside indicate mixtures of all three. The area of a circles is proportional to number of mature organisms with that phenotype. Colors differentiate between phenotypic decisions to deconstruct: brown indicates no deconstruction, light red indicates deconstruction of P_1, light blue indicates deconstruction of P_0, and light purple indicates organisms who deconstructed both P_1 and P_0.

Diminishing rewards and increasing penalties

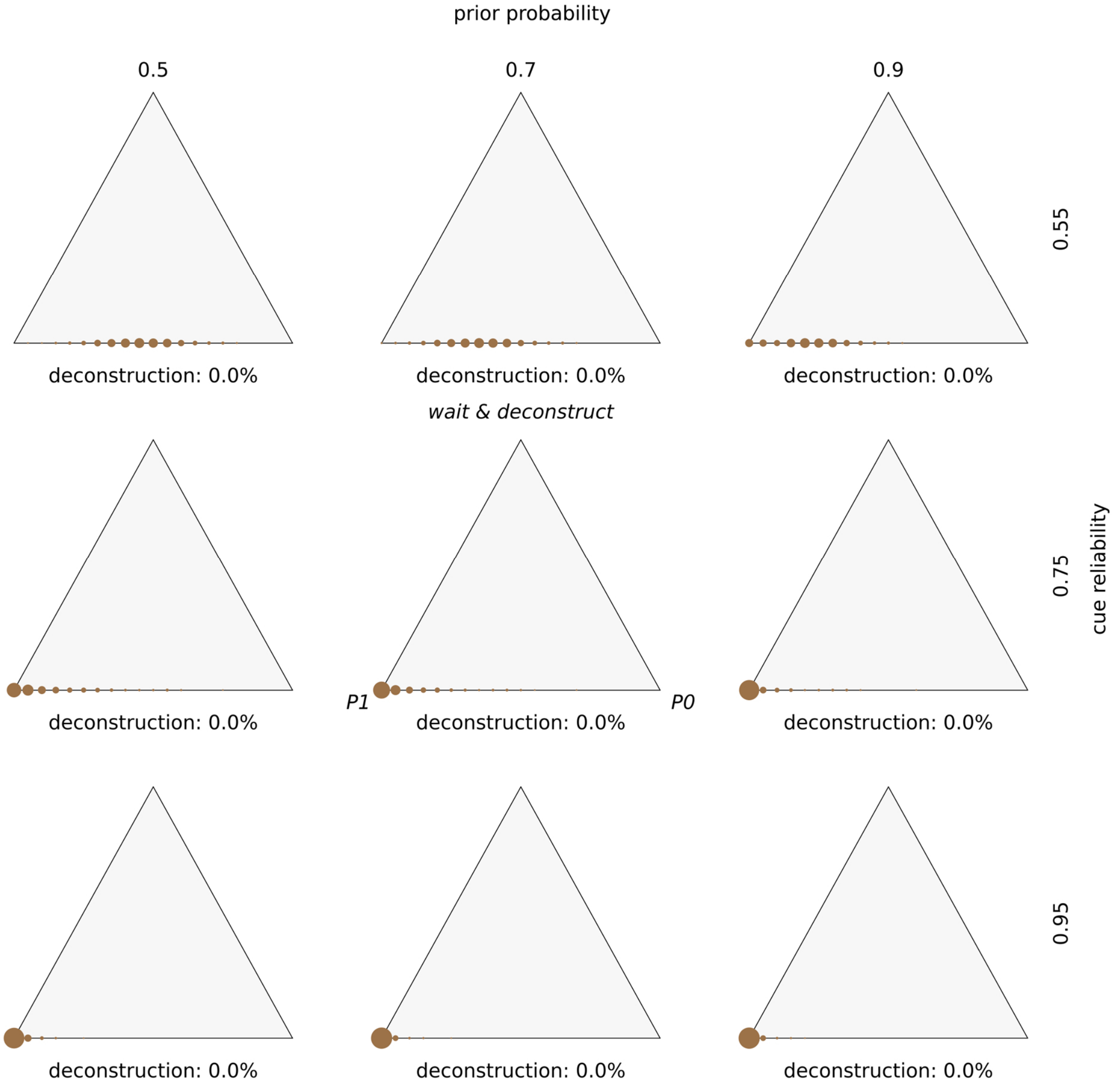

Figure S2.26: Distributions of mature phenotypes (incremental deconstruction, diminishing rewards and increasing penalties). Columns indicate the prior estimate of being in E_1 and rows indicate the cue reliability. The populations of mature phenotypes have been simulated in E_1. The horizontal label of each panel indicates the percentage of mature phenotypes that have deconstructed at some point during ontogeny. Each triangle depicts the distribution of mature phenotypes. The number of time periods spent specializing towards P_1 and P_0, the number of time periods spent deconstructing P_1 and P_0, and the number of time periods spent waiting make up a phenotype. A circle's position indicates the phenotypic composition: The left and right vertices correspond to organisms who fully specialized towards P_1 or P_0. The top vertex corresponds to organisms who only waited and/or deconstructed. Circles on the outer boundary indicate mixtures between two of the vertices and circles inside indicate mixtures of all three. The area of a circles is proportional to number of mature organisms with that phenotype. Colors differentiate between phenotypic decisions to deconstruct: brown indicates no deconstruction, light red indicates deconstruction of P_1, light blue indicates deconstruction of P_0, and light purple indicates organisms who deconstructed both P_1 and P_0.

Diminishing rewards and diminishing penalties

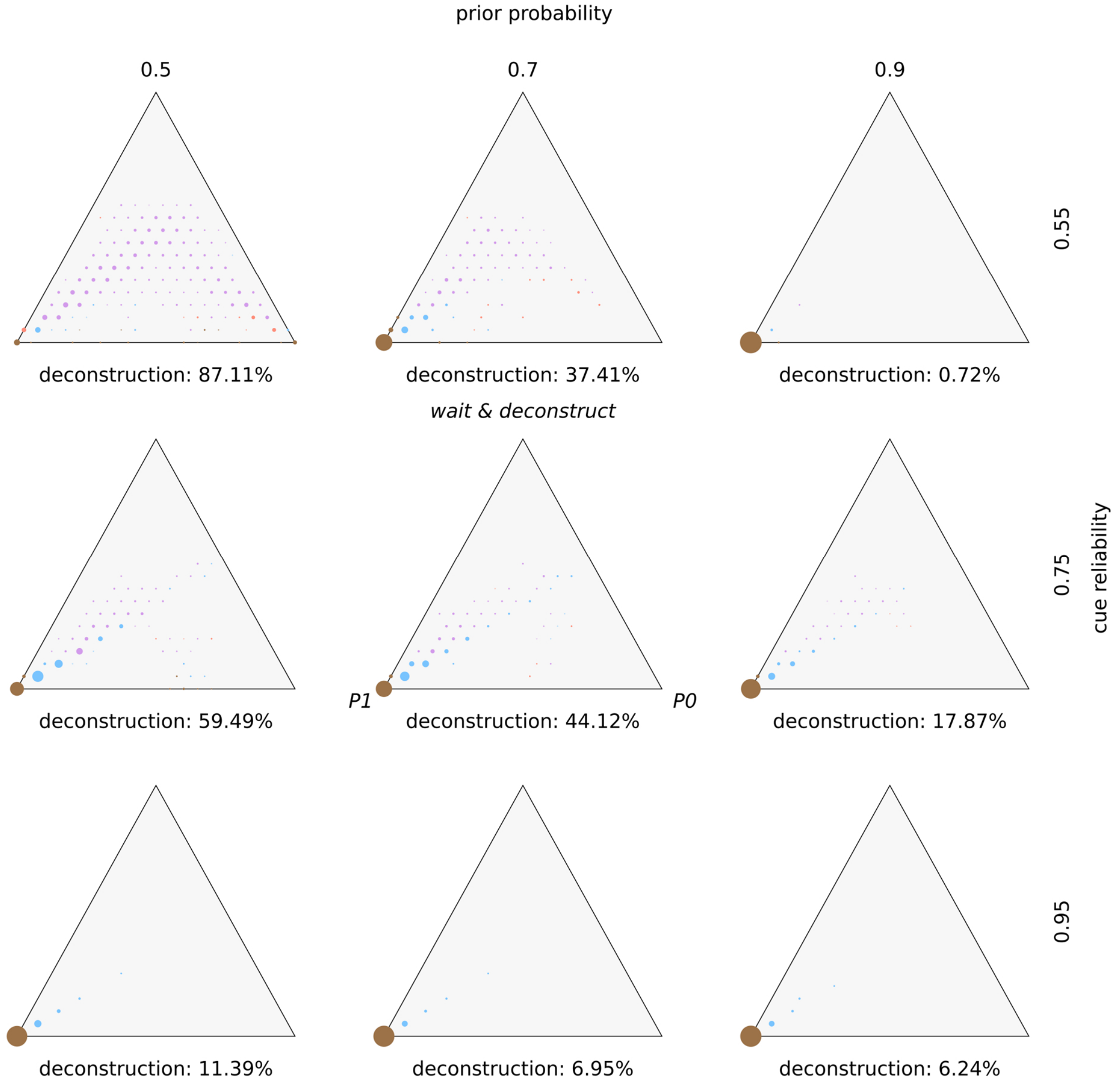

Figure S2.27: Distributions of mature phenotypes (incremental deconstruction, diminishing rewards and diminishing penalties). Columns indicate the prior estimate of being in E_1 and rows indicate the cue reliability. The populations of mature phenotypes have been simulated in E_1. The horizontal label of each panel indicates the percentage of mature phenotypes that have deconstructed at some point during ontogeny. Each triangle depicts the distribution of mature phenotypes. The number of time periods spent specializing towards P_1 and P_0, the number of time periods spent deconstructing P_1 and P_0, and the number of time periods spent waiting make up a phenotype. A circle's position indicates the phenotypic composition: The left and right vertices correspond to organisms who fully specialized towards P_1 or P_0. The top vertex corresponds to organisms who only waited and/or deconstructed. Circles on the outer boundary indicate mixtures between two of the vertices and circles inside indicate mixtures of all three. The area of a circles is proportional to number of mature organisms with that phenotype. Colors differentiate between phenotypic decisions to deconstruct: brown indicates no deconstruction, light red indicates deconstruction of P_1, light blue indicates deconstruction of P_0, and light purple indicates organisms who deconstructed both P_1 and P_0.

Triangular distributions of mature phenotypes (complete deconstruction)

Linear rewards and linear penalties

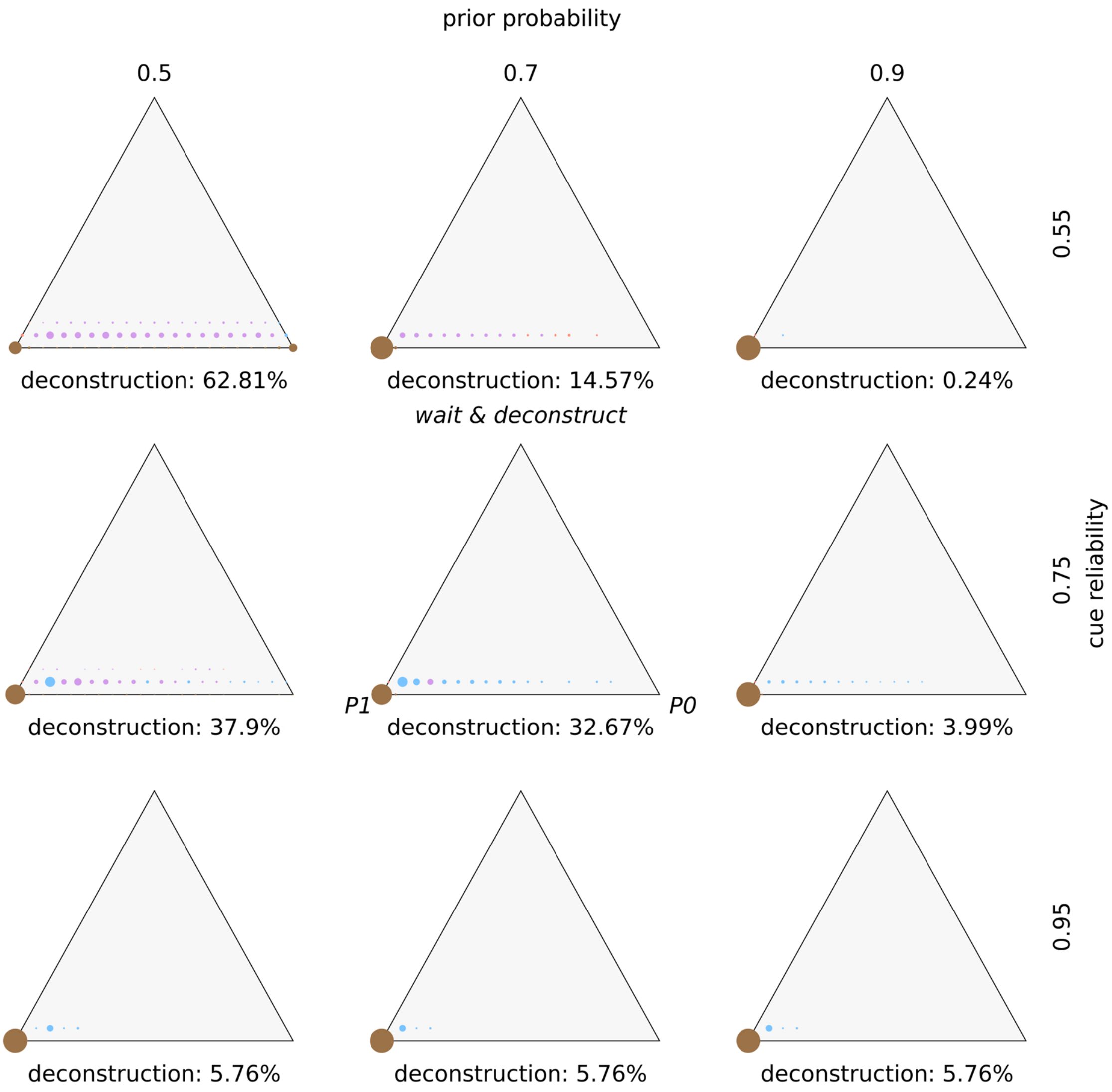

Figure S2.28: Distributions of mature phenotypes (complete deconstruction, linear rewards and penalties). Columns indicate the prior estimate of being in E_1 and rows indicate the cue reliability. The populations of mature phenotypes have been simulated in E_1. The horizontal label of each panel indicates the percentage of mature phenotypes that have deconstructed at some point during ontogeny. Each triangle depicts the distribution of mature phenotypes. The number of time periods spent specializing towards P_1 and P_0, the number of time periods spent deconstructing P_1 and P_0, and the number of time periods spent waiting make up a phenotype. A circle's position indicates the phenotypic composition: The left and right vertices correspond to organisms who fully specialized towards P_1 or P_0. The top vertex corresponds to organisms who only waited and/or deconstructed. Circles on the outer boundary indicate mixtures between two of the vertices and circles inside indicate mixtures of all three. The area of a circles is proportional to number of mature organisms with that phenotype. Colors differentiate between phenotypic decisions to deconstruct: brown indicates no deconstruction, light red indicates deconstruction of P_1, light blue indicates deconstruction of P_0, and light purple indicates organisms who deconstructed both P_1 and P_0.

Linear rewards and increasing penalties

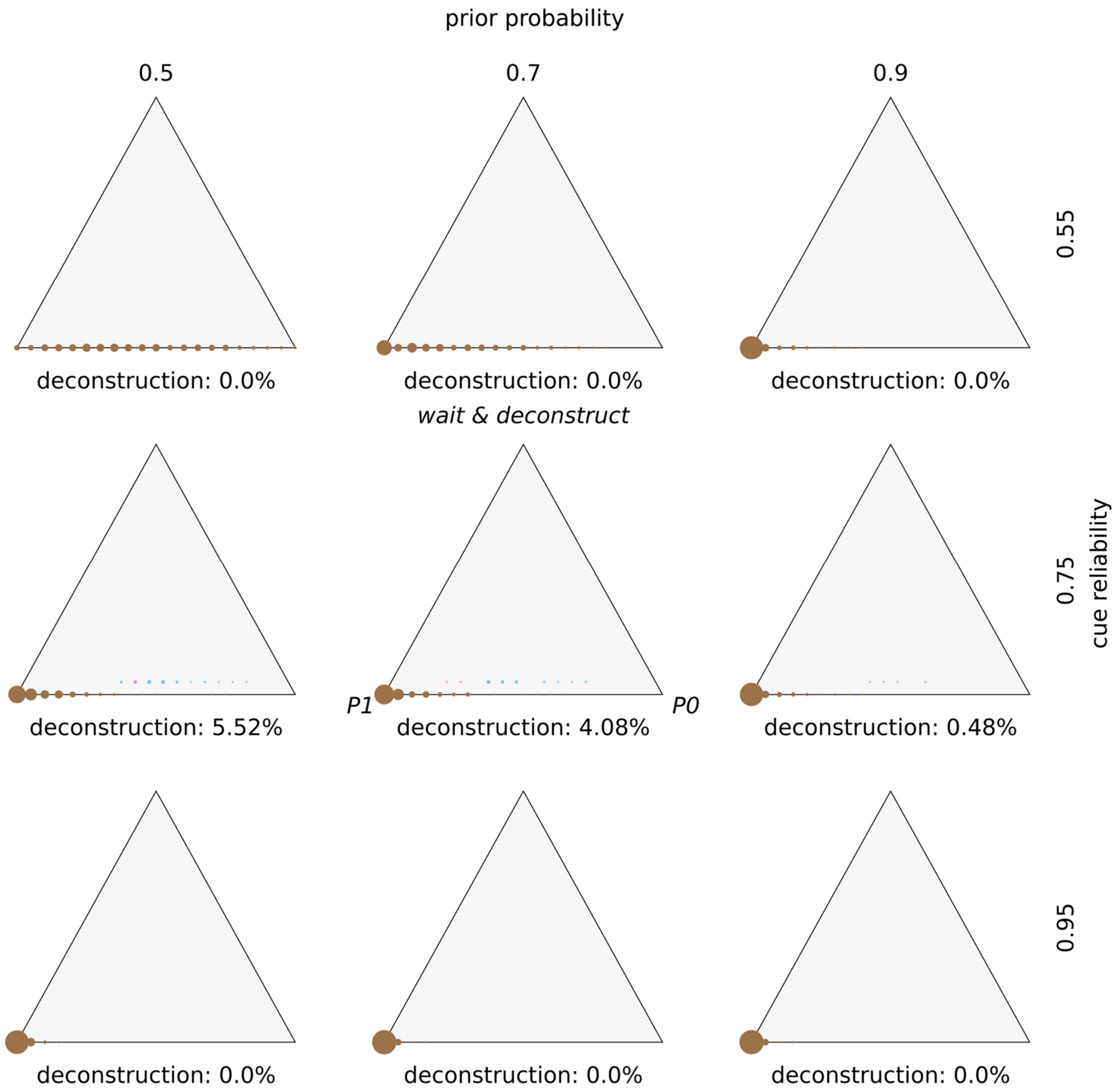

Figure S2.29: Distributions of mature phenotypes (complete deconstruction, linear rewards and increasing penalties). Columns indicate the prior estimate of being in E_1 and rows indicate the cue reliability. The populations of mature phenotypes have been simulated in E_1. The horizontal label of each panel indicates the percentage of mature phenotypes that have deconstructed at some point during ontogeny. Each triangle depicts the distribution of mature phenotypes. The number of time periods spent specializing towards P_1 and P_0, the number of time periods spent deconstructing P_1 and P_0, and the number of time periods spent waiting make up a phenotype. A circle's position indicates the phenotypic composition: The left and right vertices correspond to organisms who fully specialized towards P_1 or P_0. The top vertex corresponds to organisms who only waited and/or deconstructed. Circles on the outer boundary indicate mixtures between two of the vertices and circles inside indicate mixtures of all three. The area of a circles is proportional to number of mature organisms with that phenotype. Colors differentiate between phenotypic decisions to deconstruct: brown indicates no deconstruction, light red indicates deconstruction of P_1, light blue indicates deconstruction of P_0, and light purple indicates organisms who deconstructed both P_1 and P_0.

Linear rewards and diminishing penalties

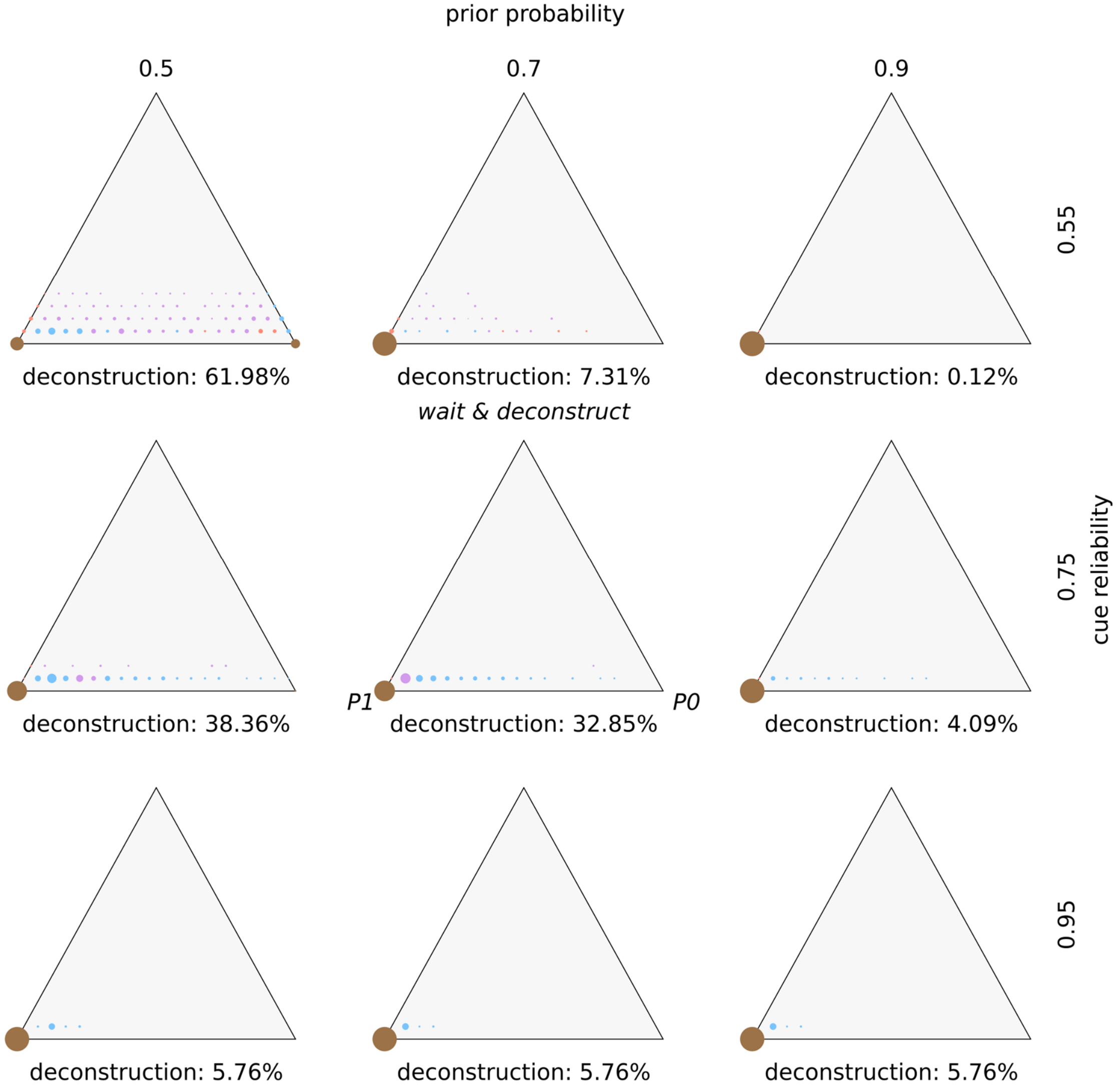

Figure S2.30: Distributions of mature phenotypes (complete deconstruction, linear rewards and diminishing penalties). Columns indicate the prior estimate of being in E_1 and rows indicate the cue reliability. The populations of mature phenotypes have been simulated in E_1. The horizontal label of each panel indicates the percentage of mature phenotypes that have deconstructed at some point during ontogeny. Each triangle depicts the distribution of mature phenotypes. The number of time periods spent specializing towards P_1 and P_0, the number of time periods spent deconstructing P_1 and P_0, and the number of time periods spent waiting make up a phenotype. A circle's position indicates the phenotypic composition: The left and right vertices correspond to organisms who fully specialized towards P_1 or P_0. The top vertex corresponds to organisms who only waited and/or deconstructed. Circles on the outer boundary indicate mixtures between two of the vertices and circles inside indicate mixtures of all three. The area of a circles is proportional to number of mature organisms with that phenotype. Colors differentiate between phenotypic decisions to deconstruct: brown indicates no deconstruction, light red indicates deconstruction of P_1, light blue indicates deconstruction of P_0, and light purple indicates organisms who deconstructed both P_1 and P_0.

Increasing rewards and linear penalties

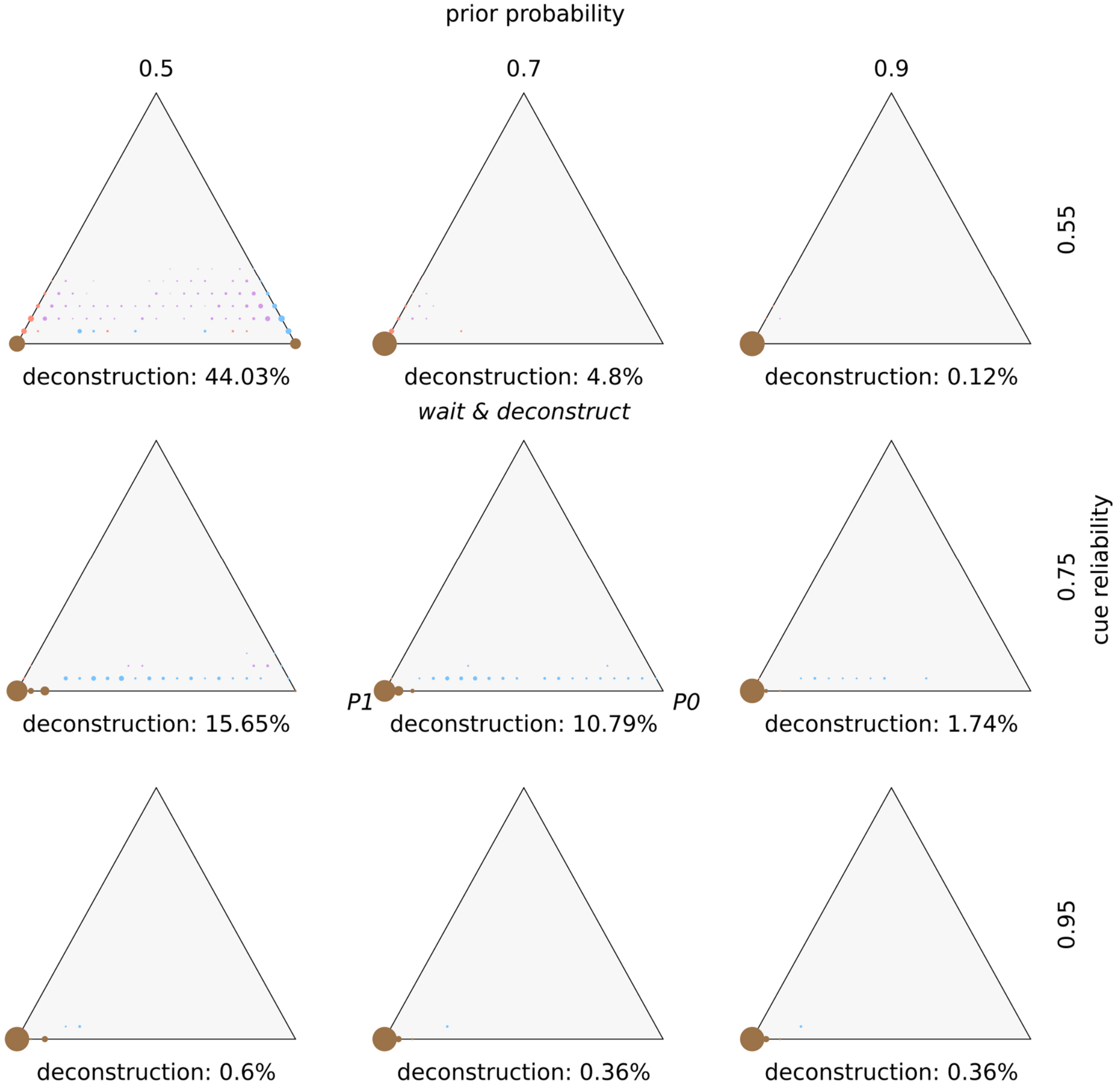

Figure S2.31: Distributions of mature phenotypes (complete deconstruction, increasing rewards and linear penalties). Columns indicate the prior estimate of being in E_1 and rows indicate the cue reliability. The populations of mature phenotypes have been simulated in E_1. The horizontal label of each panel indicates the percentage of mature phenotypes that have deconstructed at some point during ontogeny. Each triangle depicts the distribution of mature phenotypes. The number of time periods spent specializing towards P_1 and P_0, the number of time periods spent deconstructing P_1 and P_0, and the number of time periods spent waiting make up a phenotype. A circle's position indicates the phenotypic composition: The left and right vertices correspond to organisms who fully specialized towards P_1 or P_0. The top vertex corresponds to organisms who only waited and/or deconstructed. Circles on the outer boundary indicate mixtures between two of the vertices and circles inside indicate mixtures of all three. The area of a circles is proportional to number of mature organisms with that phenotype. Colors differentiate between phenotypic decisions to deconstruct: brown indicates no deconstruction, light red indicates deconstruction of P_1, light blue indicates deconstruction of P_0, and light purple indicates organisms who deconstructed both P_1 and P_0.

Increasing rewards and increasing penalties

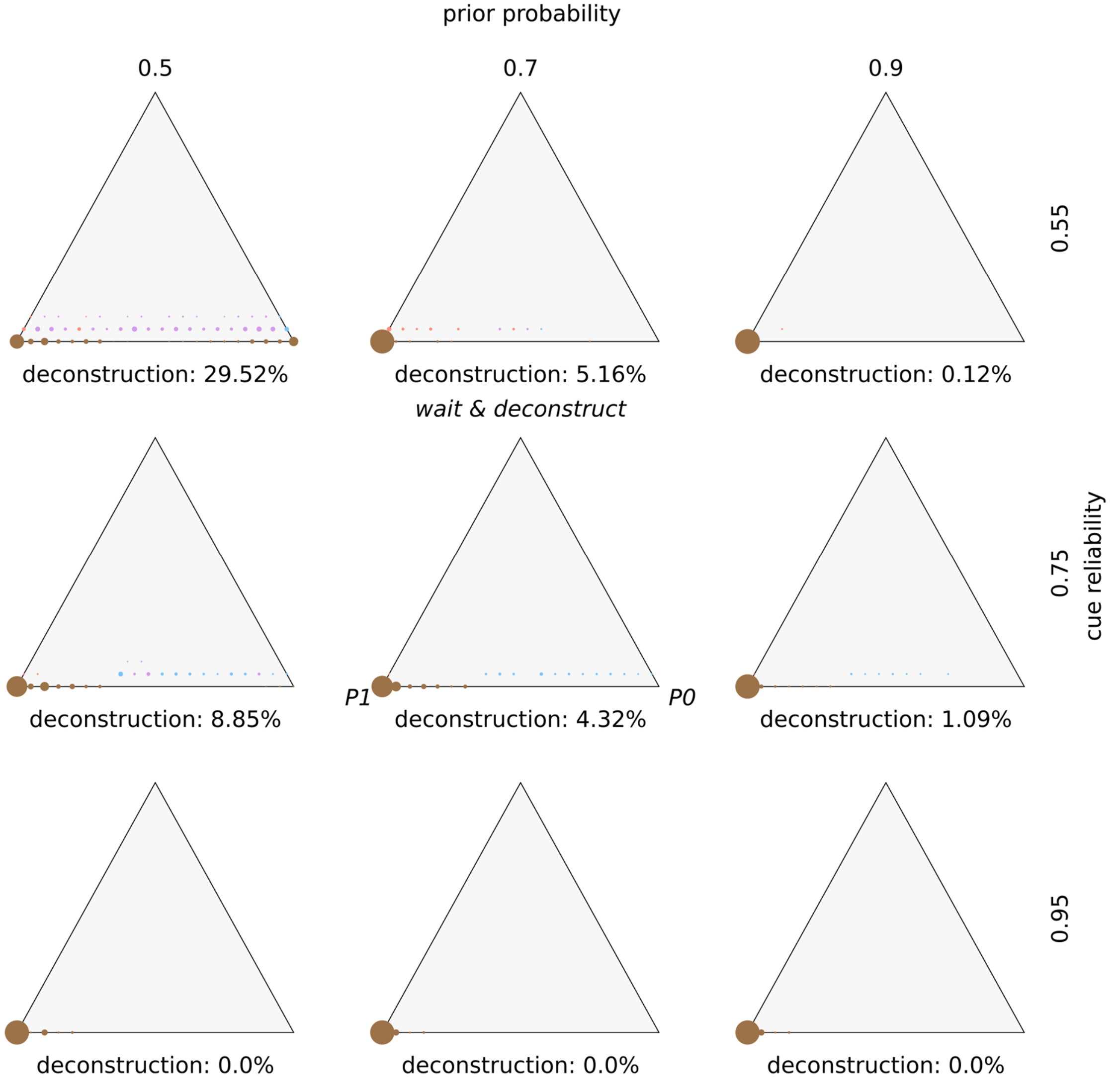

Figure S2.32: Distributions of mature phenotypes (complete deconstruction, increasing rewards and increasing penalties). Columns indicate the prior estimate of being in E_1 and rows indicate the cue reliability. The populations of mature phenotypes have been simulated in E_1. The horizontal label of each panel indicates the percentage of mature phenotypes that have deconstructed at some point during ontogeny. Each triangle depicts the distribution of mature phenotypes. The number of time periods spent specializing towards P_1 and P_0, the number of time periods spent deconstructing P_1 and P_0, and the number of time periods spent waiting make up a phenotype. A circle's position indicates the phenotypic composition: The left and right vertices correspond to organisms who fully specialized towards P_1 or P_0. The top vertex corresponds to organisms who only waited and/or deconstructed. Circles on the outer boundary indicate mixtures between two of the vertices and circles inside indicate mixtures of all three. The area of a circles is proportional to number of mature organisms with that phenotype. Colors differentiate between phenotypic decisions to deconstruct: brown indicates no deconstruction, light red indicates deconstruction of P_1, light blue indicates deconstruction of P_0, and light purple indicates organisms who deconstructed both P_1 and P_0.

Increasing rewards and diminishing penalties

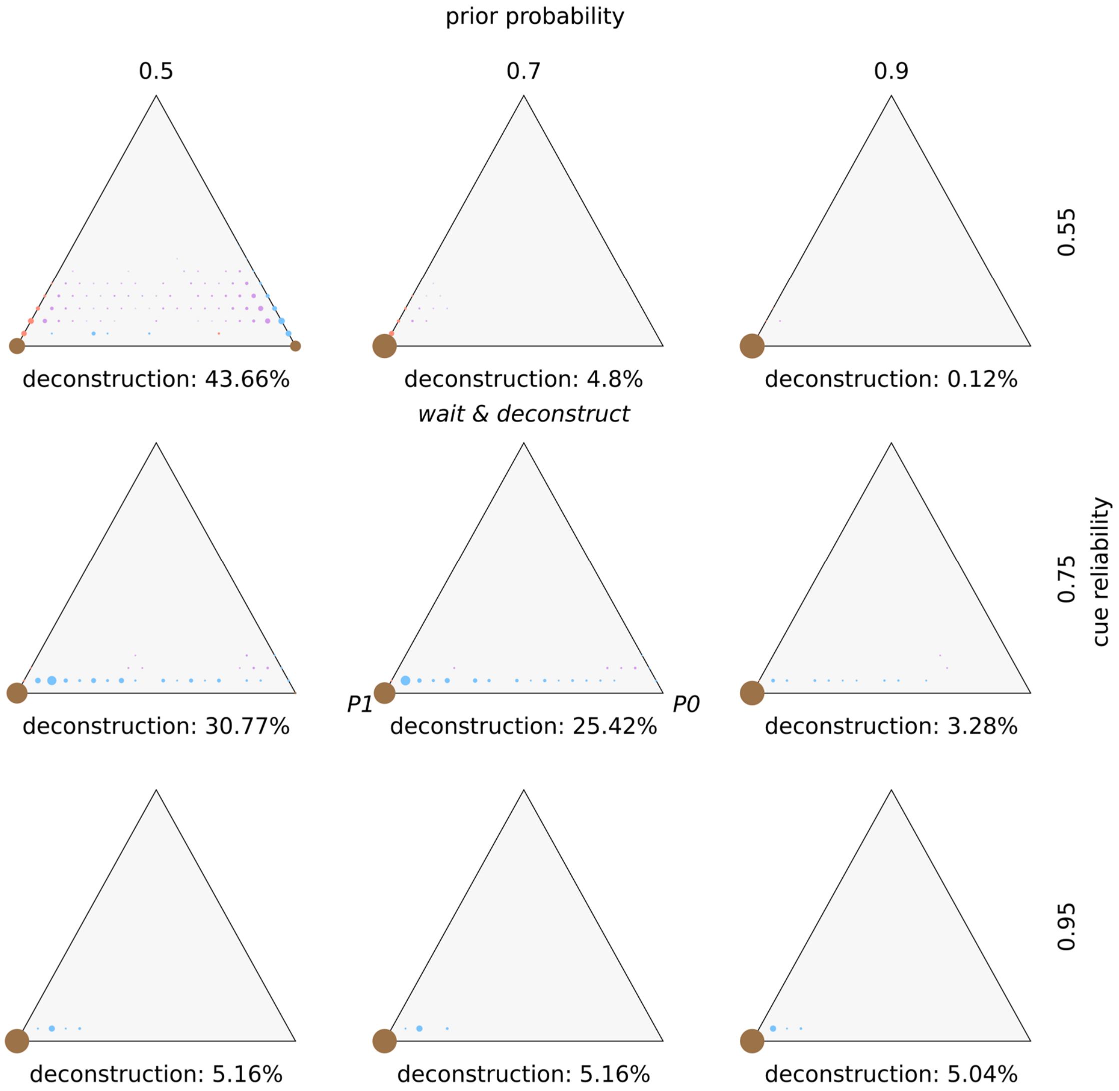

Figure S2.33: Distributions of mature phenotypes (complete deconstruction, increasing rewards and diminishing penalties). Columns indicate the prior estimate of being in E_1 and rows indicate the cue reliability. The populations of mature phenotypes have been simulated in E_1. The horizontal label of each panel indicates the percentage of mature phenotypes that have deconstructed at some point during ontogeny. Each triangle depicts the distribution of mature phenotypes. The number of time periods spent specializing towards P_1 and P_0, the number of time periods spent deconstructing P_1 and P_0, and the number of time periods spent waiting make up a phenotype. A circle's position indicates the phenotypic composition: The left and right vertices correspond to organisms who fully specialized towards P_1 or P_0. The top vertex corresponds to organisms who only waited and/or deconstructed. Circles on the outer boundary indicate mixtures between two of the vertices and circles inside indicate mixtures of all three. The area of a circles is proportional to number of mature organisms with that phenotype. Colors differentiate between phenotypic decisions to deconstruct: brown indicates no deconstruction, light red indicates deconstruction of P_1, light blue indicates deconstruction of P_0, and light purple indicates organisms who deconstructed both P_1 and P_0.

Diminishing rewards and linear penalties

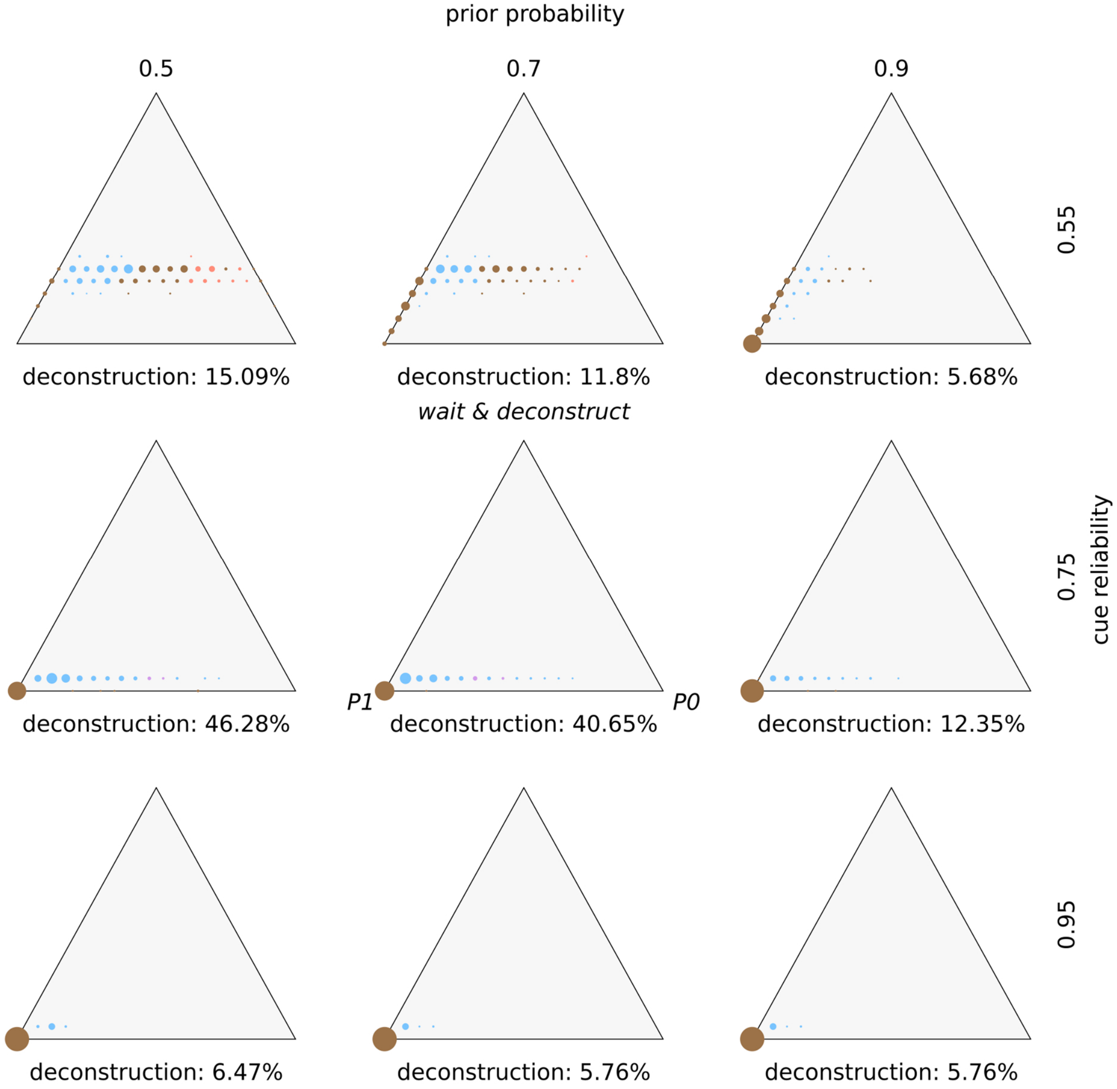

Figure S2.34: Distributions of mature phenotypes (complete deconstruction, diminishing rewards and linear penalties). Columns indicate the prior estimate of being in E_1 and rows indicate the cue reliability. The populations of mature phenotypes have been simulated in E_1. The horizontal label of each panel indicates the percentage of mature phenotypes that have deconstructed at some point during ontogeny. Each triangle depicts the distribution of mature phenotypes. The number of time periods spent specializing towards P_1 and P_0, the number of time periods spent deconstructing P_1 and P_0, and the number of time periods spent waiting make up a phenotype. A circle's position indicates the phenotypic composition: The left and right vertices correspond to organisms who fully specialized towards P_1 or P_0. The top vertex corresponds to organisms who only waited and/or deconstructed. Circles on the outer boundary indicate mixtures between two of the vertices and circles inside indicate mixtures of all three. The area of a circles is proportional to number of mature organisms with that phenotype. Colors differentiate between phenotypic decisions to deconstruct: brown indicates no deconstruction, light red indicates deconstruction of P_1, light blue indicates deconstruction of P_0, and light purple indicates organisms who deconstructed both P_1 and P_0.

Diminishing rewards and increasing penalties

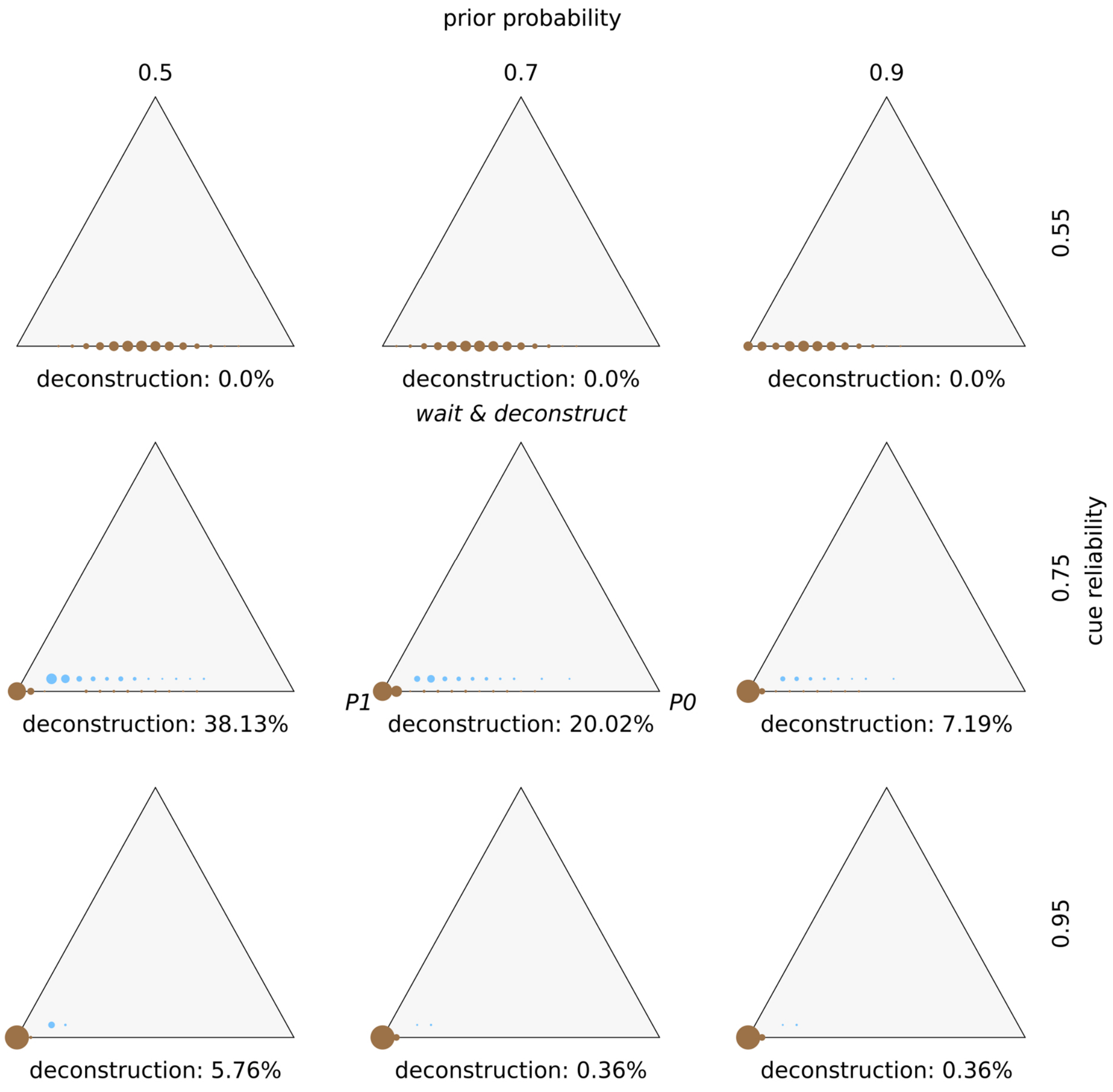

Figure S2.35: Distributions of mature phenotypes (complete deconstruction, diminishing rewards and increasing penalties). Columns indicate the prior estimate of being in E_1 and rows indicate the cue reliability. The populations of mature phenotypes have been simulated in E_1. The horizontal label of each panel indicates the percentage of mature phenotypes that have deconstructed at some point during ontogeny. Each triangle depicts the distribution of mature phenotypes. The number of time periods spent specializing towards P_1 and P_0, the number of time periods spent deconstructing P_1 and P_0, and the number of time periods spent waiting make up a phenotype. A circle's position indicates the phenotypic composition: The left and right vertices correspond to organisms who fully specialized towards P_1 or P_0. The top vertex corresponds to organisms who only waited and/or deconstructed. Circles on the outer boundary indicate mixtures between two of the vertices and circles inside indicate mixtures of all three. The area of a circles is proportional to number of mature organisms with that phenotype. Colors differentiate between phenotypic decisions to deconstruct: brown indicates no deconstruction, light red indicates deconstruction of P_1, light blue indicates deconstruction of P_0, and light purple indicates organisms who deconstructed both P_1 and P_0.

Diminishing rewards and diminishing penalties

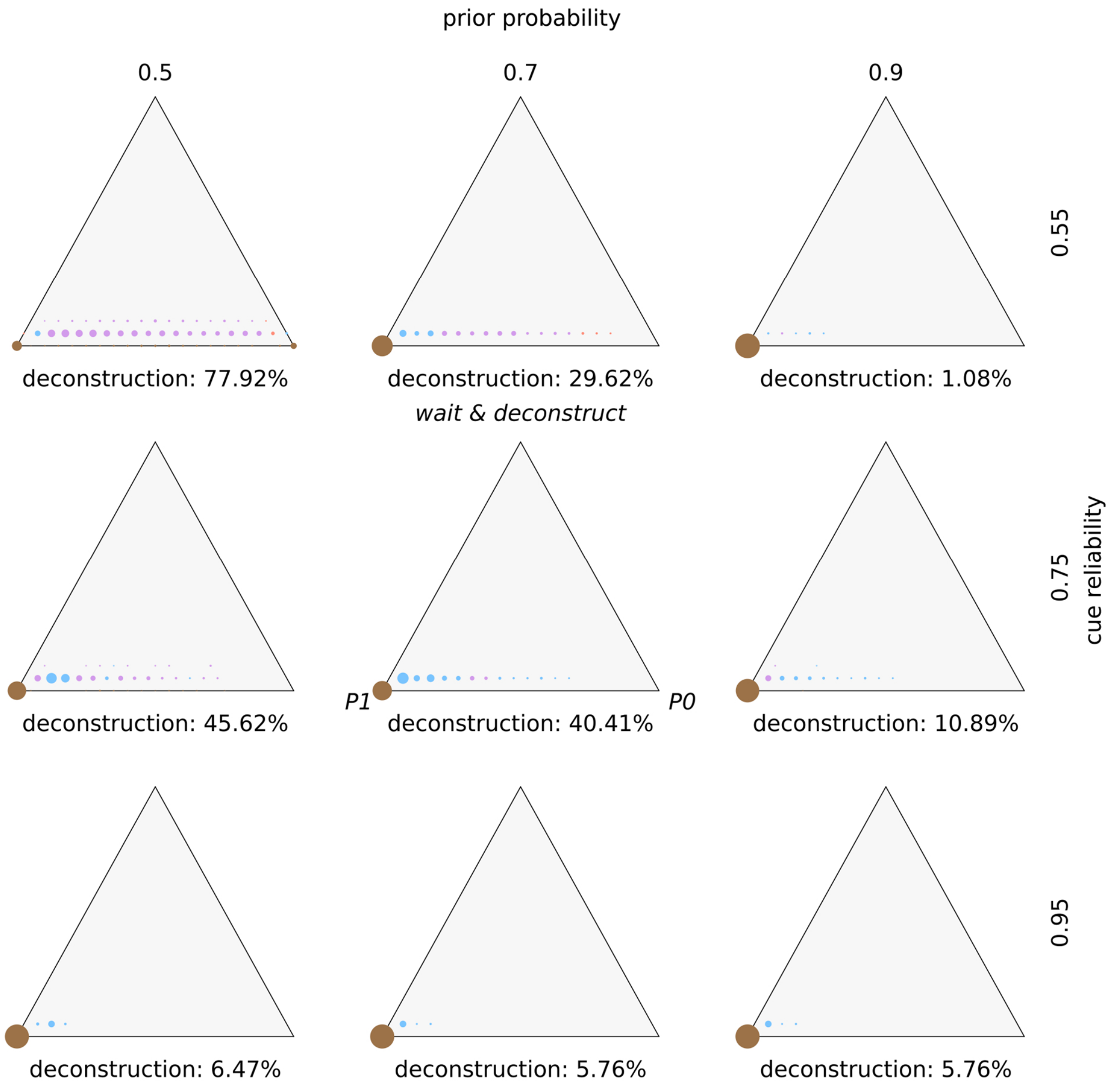

Figure S2.36: Distributions of mature phenotypes (complete deconstruction, diminishing rewards and diminishing penalties). Columns indicate the prior estimate of being in E_1 and rows indicate the cue reliability. The populations of mature phenotypes have been simulated in E_1. The horizontal label of each panel indicates the percentage of mature phenotypes that have deconstructed at some point during ontogeny. Each triangle depicts the distribution of mature phenotypes. The number of time periods spent specializing towards P_1 and P_0, the number of time periods spent deconstructing P_1 and P_0, and the number of time periods spent waiting make up a phenotype. A circle's position indicates the phenotypic composition: The left and right vertices correspond to organisms who fully specialized towards P_1 or P_0. The top vertex corresponds to organisms who only waited and/or deconstructed. Circles on the outer boundary indicate mixtures between two of the vertices and circles inside indicate mixtures of all three. The area of a circles is proportional to number of mature organisms with that phenotype. Colors differentiate between phenotypic decisions to deconstruct: brown indicates no deconstruction, light red indicates deconstruction of P_1, light blue indicates deconstruction of P_0, and light purple indicates organisms who deconstructed both P_1 and P_0.

Distributions of mature phenotypes (incremental deconstruction)

Linear rewards and linear penalties

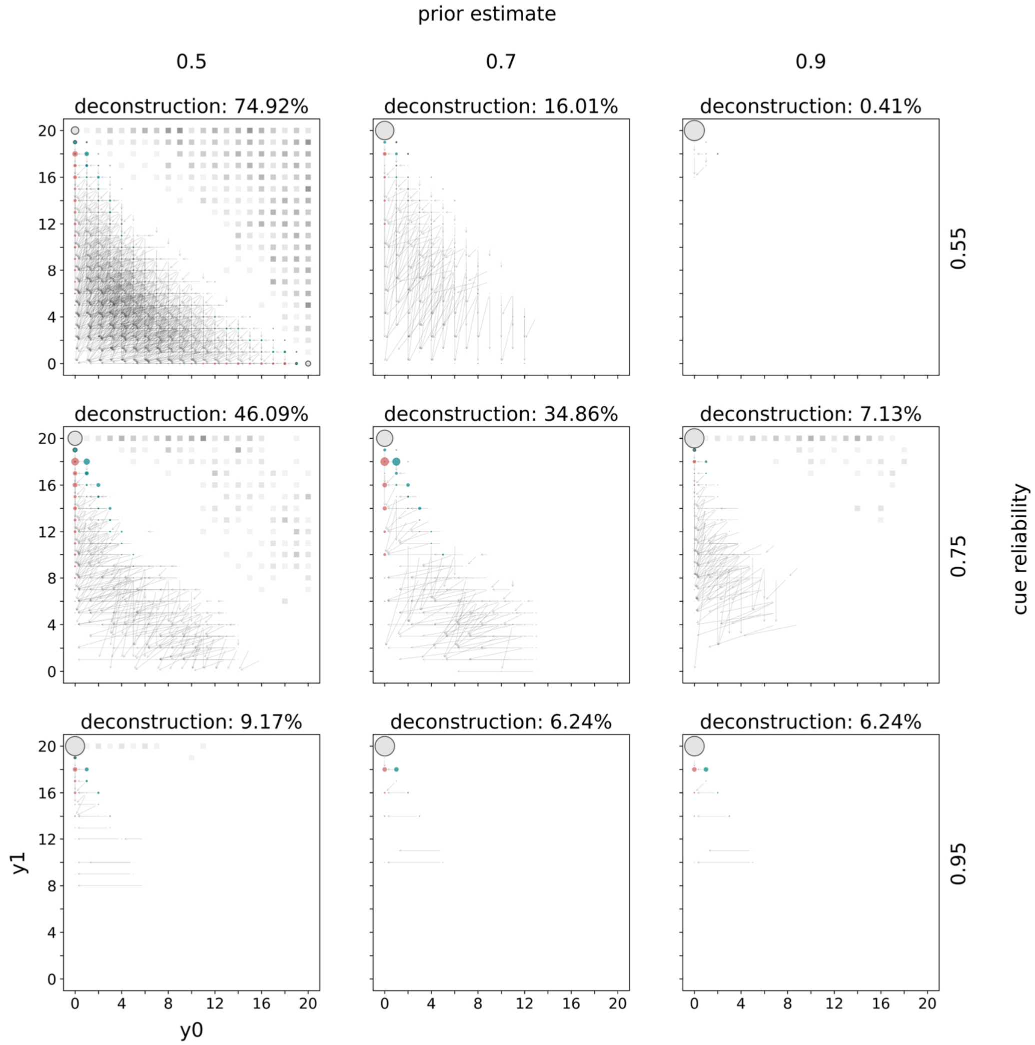

Figure S2.37: Distributions of mature phenotypes. Distributions of mature phenotypes are shown for a model with incremental deconstruction and linear rewards and penalties. Columns indicate the prior estimate of being in E_1 and rows indicate the cue reliability. The populations of mature phenotypes have been simulated in E_1. The title of each panel indicates the percentage of mature phenotypes that have deconstructed at some point during ontogeny. Within each panel the horizontal axis indicates the number of specializations towards E_0 and the vertical axis towards E_1. The lower triangle indicates how much mature phenotypes have constructed (teal circles) and what their phenotype looked like after deconstruction (red circles). Grey arrows connect phenotypes before (teal) and after (red) deconstruction. Grey circles with a black outline belong to mature phenotypes that never deconstructed. The area of a circle is proportional to the number mature organisms with this phenotype. The upper triangle indicates waiting. For each mature phenotype (after deconstruction) below the diagonal the corresponding square above the diagonal highlights the amount of waiting. The color intensity is proportional to the amount of waiting. Black squares indicate phenotypes that waited all of ontogeny (i.e. 20 time periods) and white squares phenotypes that never waited.

Linear rewards and increasing penalties

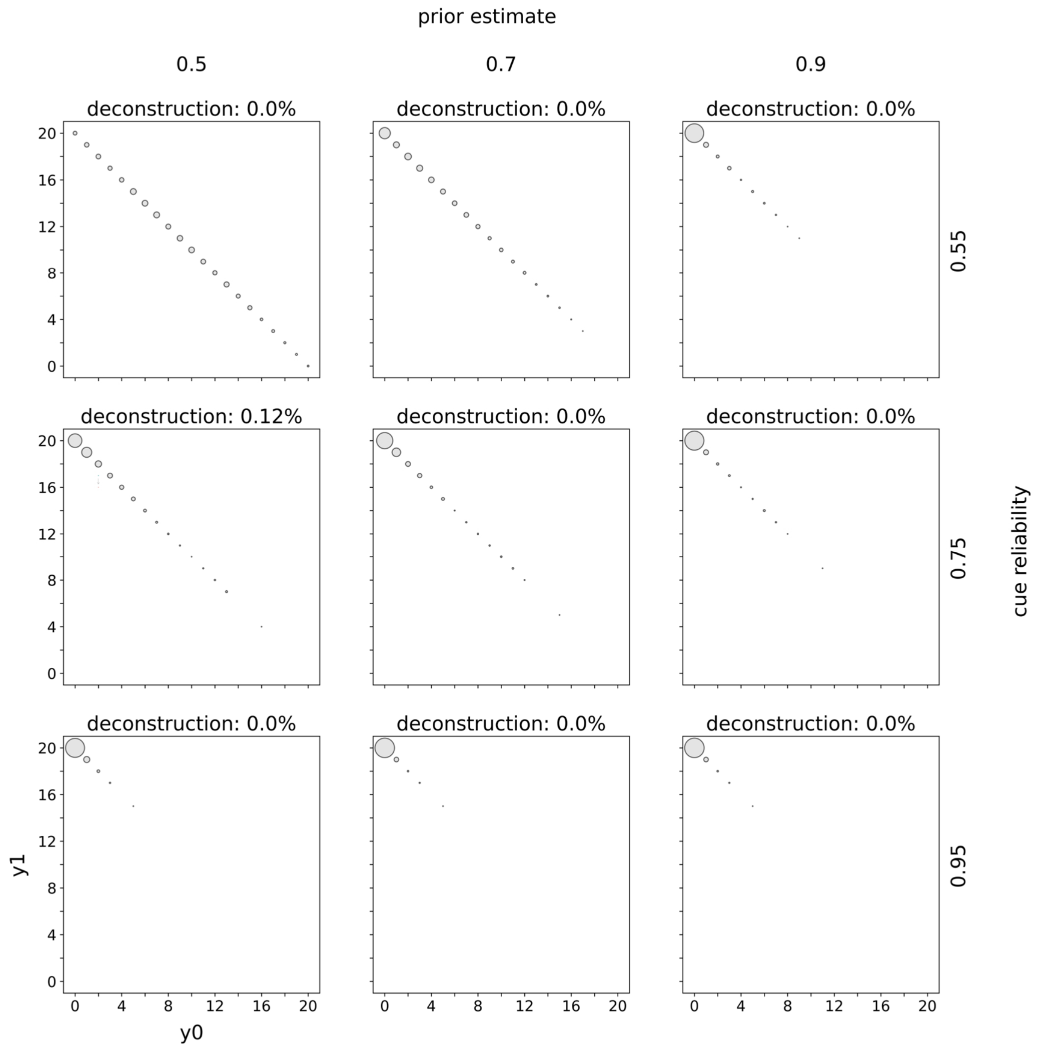

Figure S2.38: Distributions of mature phenotypes. Distributions of mature phenotypes are shown for a model with incremental deconstruction and linear rewards and increasing penalties. Columns indicate the prior estimate of being in E_1 and rows indicate the cue reliability. The populations of mature phenotypes have been simulated in E_1. The title of each panel indicates the percentage of mature phenotypes that have deconstructed at some point during ontogeny. Within each panel the horizontal axis indicates the number of specializations towards E_0 and the vertical axis towards E_1. The lower triangle indicates how much mature phenotypes have constructed (teal circles) and what their phenotype looked like after deconstruction (red circles). Grey arrows connect phenotypes before (teal) and after (red) deconstruction. Grey circles with a black outline belong to mature phenotypes that never deconstructed. The area of a circle is proportional to the number mature organisms with this phenotype. The upper triangle indicates waiting. For each mature phenotype (after deconstruction) below the diagonal the corresponding square above the diagonal highlights the amount of waiting. The color intensity is proportional to the amount of waiting. Black squares indicate phenotypes that waited all of ontogeny (i.e. 20 time periods) and white squares phenotypes that never waited.

Linear rewards and diminishing penalties

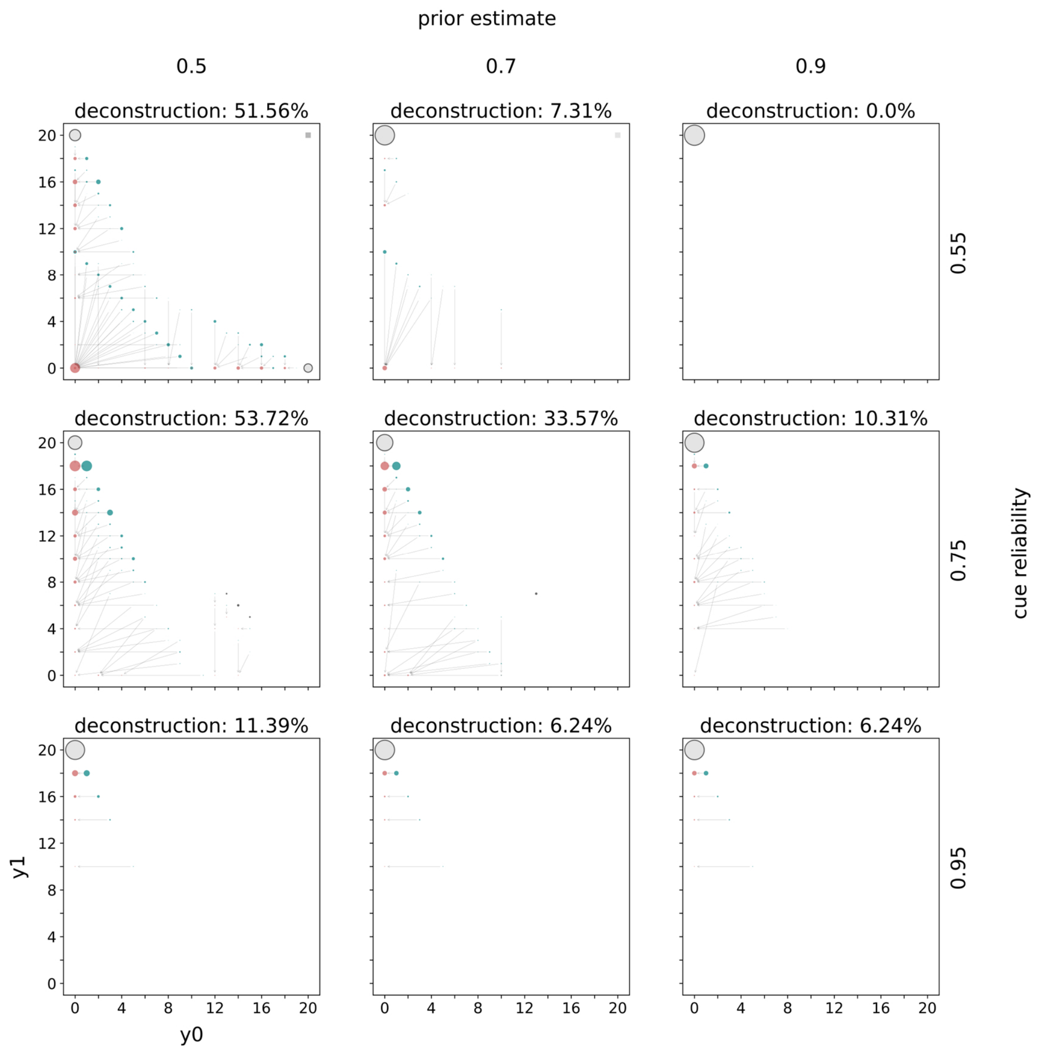

Figure S2.39: Distributions of mature phenotypes. Distributions of mature phenotypes are shown for a model with incremental deconstruction and linear rewards and diminishing penalties. Columns indicate the prior estimate of being in E_1 and rows indicate the cue reliability. The populations of mature phenotypes have been simulated in E_1. The title of each panel indicates the percentage of mature phenotypes that have deconstructed at some point during ontogeny. Within each panel the horizontal axis indicates the number of specializations towards E_0 and the vertical axis towards E_1. The lower triangle indicates how much mature phenotypes have constructed (teal circles) and what their phenotype looked like after deconstruction (red circles). Grey arrows connect phenotypes before (teal) and after (red) deconstruction. Grey circles with a black outline belong to mature phenotypes that never deconstructed. The area of a circle is proportional to the number mature organisms with this phenotype. The upper triangle indicates waiting. For each mature phenotype (after deconstruction) below the diagonal the corresponding square above the diagonal highlights the amount of waiting. The color intensity is proportional to the amount of waiting. Black squares indicate phenotypes that waited all of ontogeny (i.e. 20 time periods) and white squares phenotypes that never waited.

Increasing rewards and linear penalties

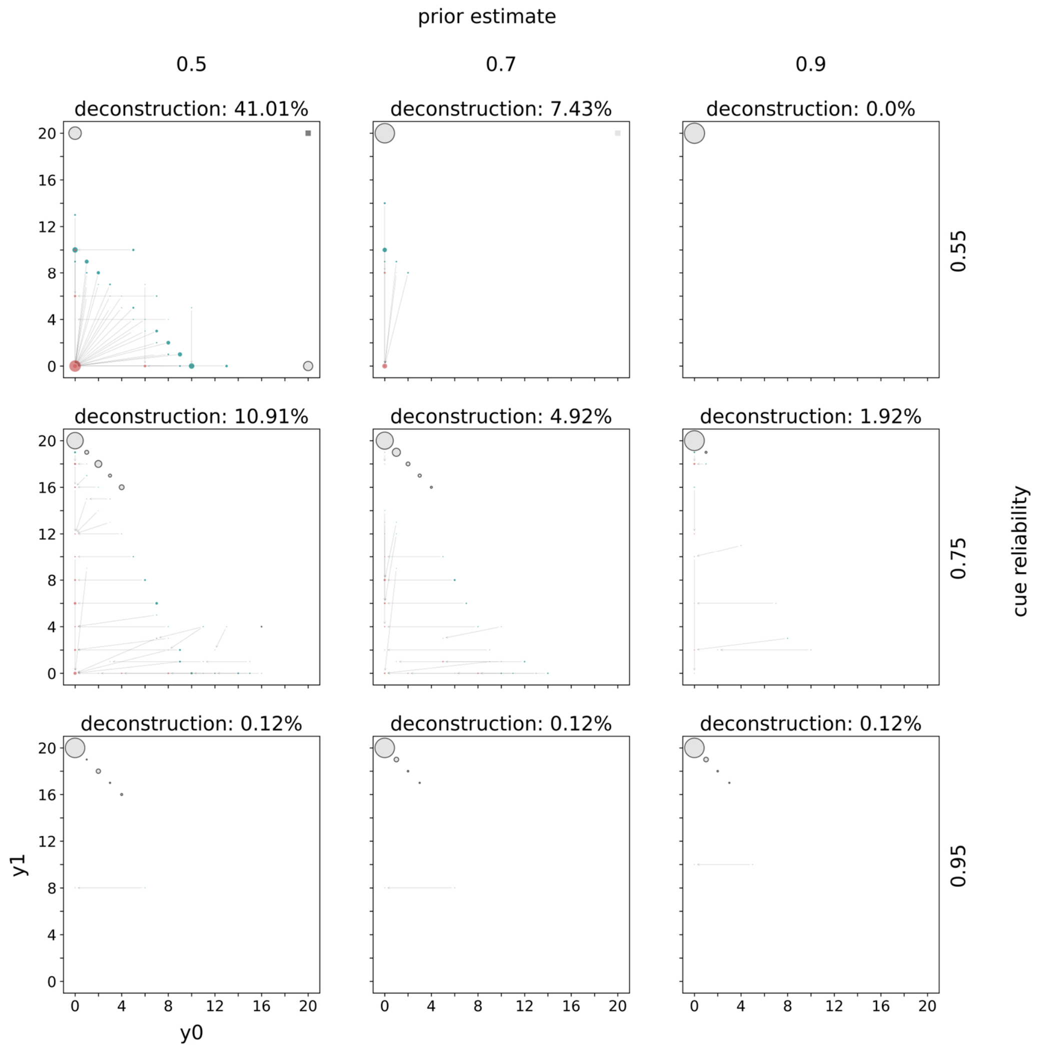

Figure S2.40: Distributions of mature phenotypes. Distributions of mature phenotypes are shown for a model with incremental deconstruction and increasing rewards and linear penalties. Columns indicate the prior estimate of being in E_1 and rows indicate the cue reliability. The populations of mature phenotypes have been simulated in E_1. The title of each panel indicates the percentage of mature phenotypes that have deconstructed at some point during ontogeny. Within each panel the horizontal axis indicates the number of specializations towards E_0 and the vertical axis towards E_1. The lower triangle indicates how much mature phenotypes have constructed (teal circles) and what their phenotype looked like after deconstruction (red circles). Grey arrows connect phenotypes before (teal) and after (red) deconstruction. Grey circles with a black outline belong to mature phenotypes that never deconstructed. The area of a circle is proportional to the number mature organisms with this phenotype. The upper triangle indicates waiting. For each mature phenotype (after deconstruction) below the diagonal the corresponding square above the diagonal highlights the amount of waiting. The color intensity is proportional to the amount of waiting. Black squares indicate phenotypes that waited all of ontogeny (i.e. 20 time periods) and white squares phenotypes that never waited.

Increasing rewards and increasing penalties

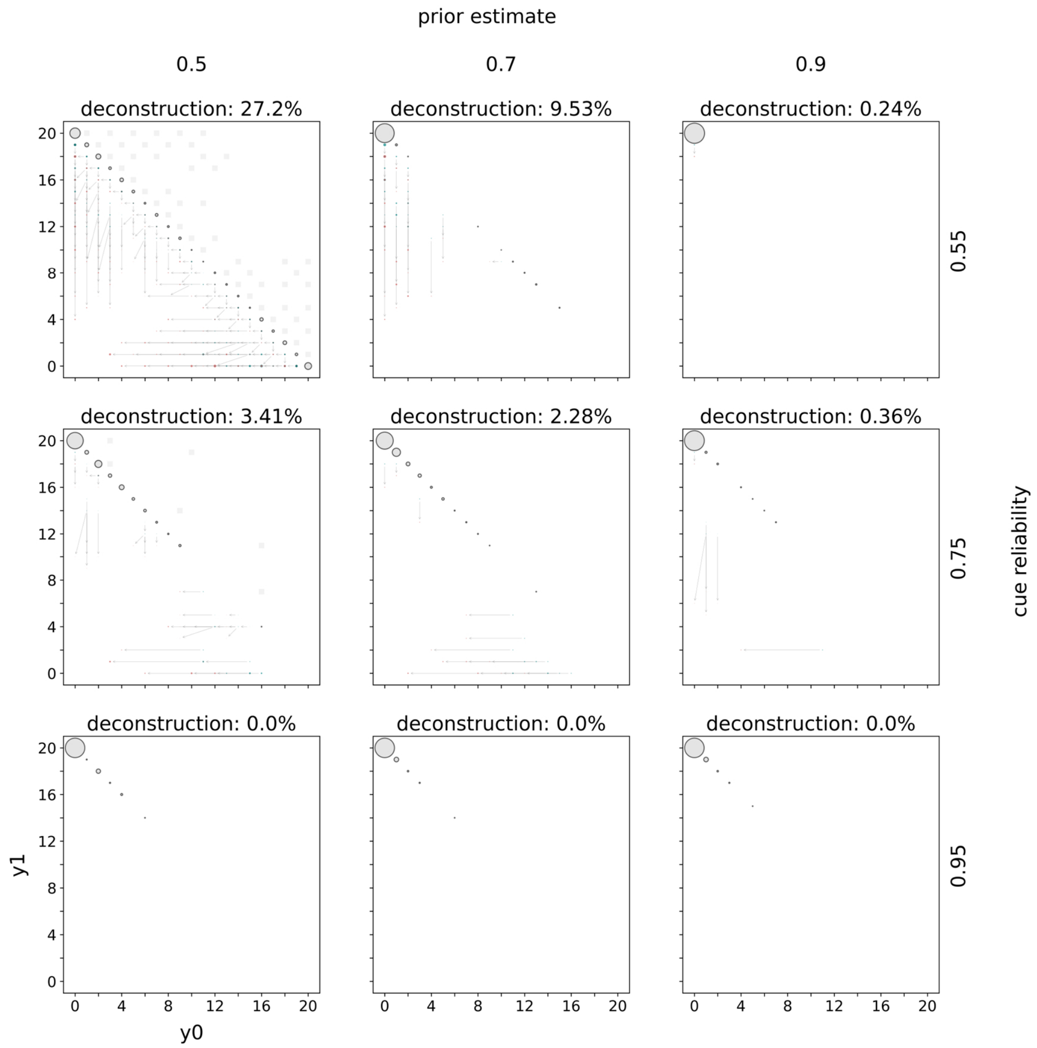

Figure S2.41: Distributions of mature phenotypes. Distributions of mature phenotypes are shown for a model with incremental deconstruction and increasing rewards and increasing penalties. Columns indicate the prior estimate of being in E_1 and rows indicate the cue reliability. The populations of mature phenotypes have been simulated in E_1. The title of each panel indicates the percentage of mature phenotypes that have deconstructed at some point during ontogeny. Within each panel the horizontal axis indicates the number of specializations towards E_0 and the vertical axis towards E_1. The lower triangle indicates how much mature phenotypes have constructed (teal circles) and what their phenotype looked like after deconstruction (red circles). Grey arrows connect phenotypes before (teal) and after (red) deconstruction. Grey circles with a black outline belong to mature phenotypes that never deconstructed. The area of a circle is proportional to the number mature organisms with this phenotype. The upper triangle indicates waiting. For each mature phenotype (after deconstruction) below the diagonal the corresponding square above the diagonal highlights the amount of waiting. The color intensity is proportional to the amount of waiting. Black squares indicate phenotypes that waited all of ontogeny (i.e. 20 time periods) and white squares phenotypes that never waited.

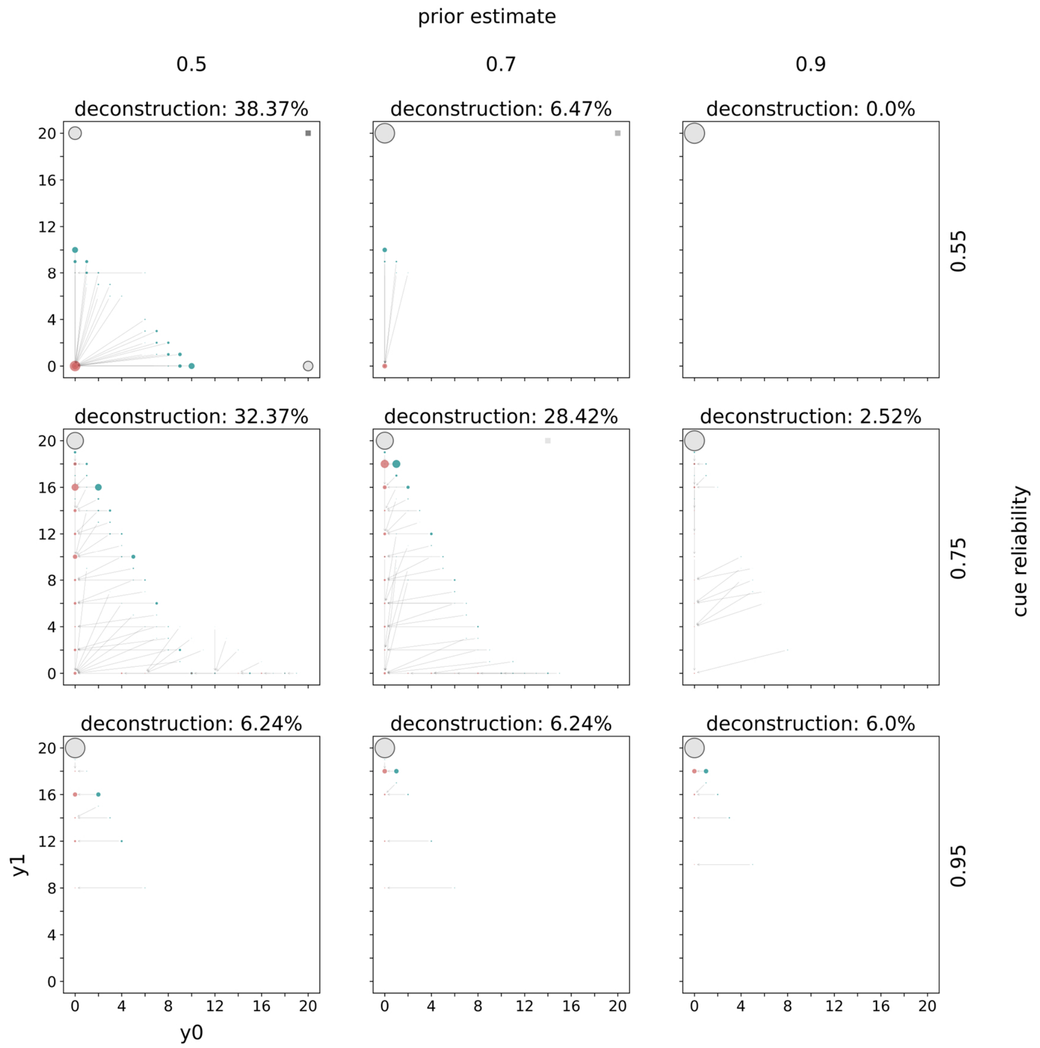

Figure S2.42: Distributions of mature phenotypes. Distributions of mature phenotypes are shown for a model with incremental deconstruction and increasing rewards and diminishing penalties. Columns indicate the prior estimate of being in E_1 and rows indicate the cue reliability. The populations of mature phenotypes have been simulated in E_1. The title of each panel indicates the percentage of mature phenotypes that have deconstructed at some point during ontogeny. Within each panel the horizontal axis indicates the number of specializations towards E_0 and the vertical axis towards E_1. The lower triangle indicates how much mature phenotypes have constructed (teal circles) and what their phenotype looked like after deconstruction (red circles). Grey arrows connect phenotypes before (teal) and after (red) deconstruction. Grey circles with a black outline belong to mature phenotypes that never deconstructed. The area of a circle is proportional to the number mature organisms with this phenotype. The upper triangle indicates waiting. For each mature phenotype (after deconstruction) below the diagonal the corresponding square above the diagonal highlights the amount of waiting. The color intensity is proportional to the amount of waiting. Black squares indicate phenotypes that waited all of ontogeny (i.e. 20 time periods) and white squares phenotypes that never waited.

Diminishing rewards and linear penalties

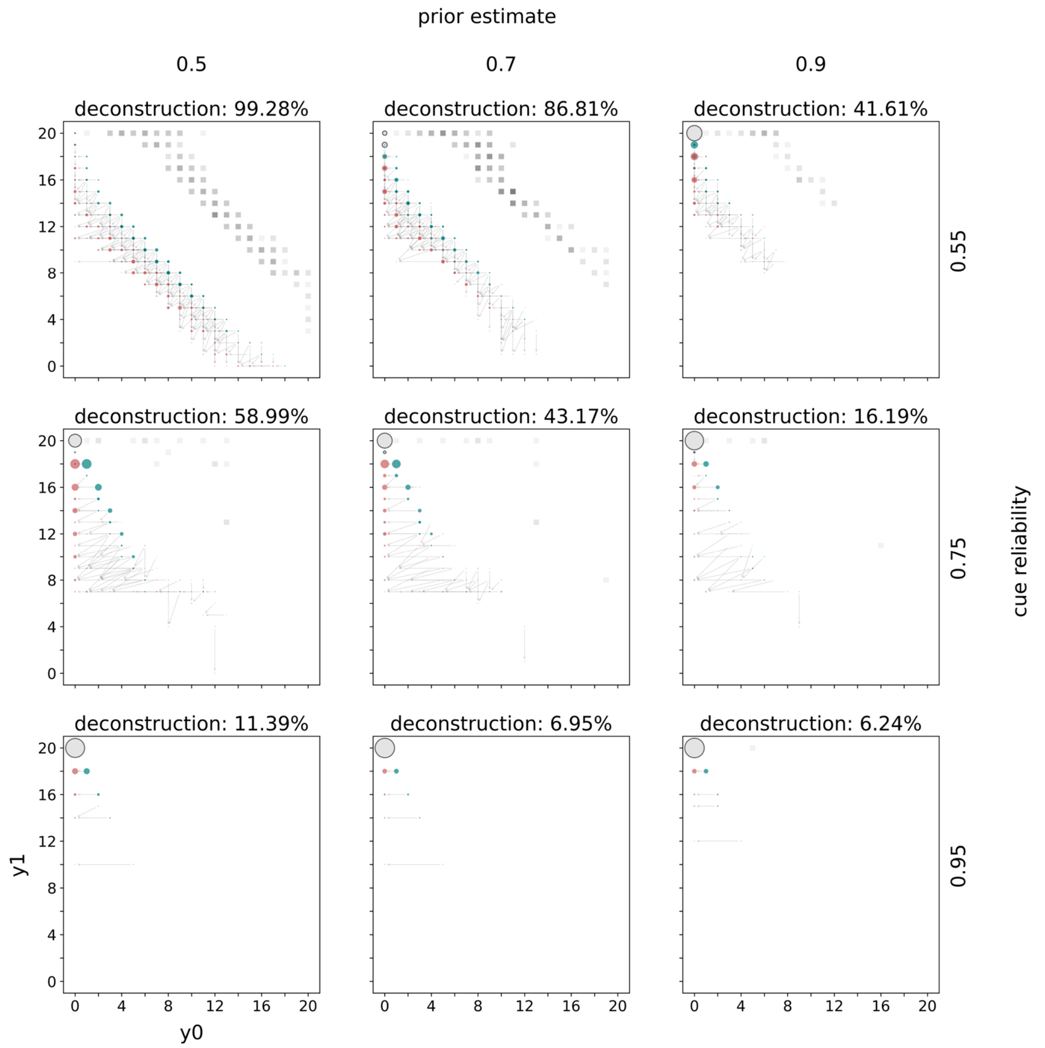

Figure S2.43: Distributions of mature phenotypes. Distributions of mature phenotypes are shown for a model with incremental deconstruction and diminishing rewards and linear penalties. COLUMNS indicate the prior estimate of being in E_1 and rows indicate the cue reliability. The populations of mature phenotypes have been simulated in E_1. The title of each panel indicates the percentage of mature phenotypes that have deconstructed at some point during ontogeny. Within each panel the horizontal axis indicates the number of specializations towards E_0 and the vertical axis towards E_1. The lower triangle indicates how much mature phenotypes have constructed (teal circles) and what their phenotype looked like after deconstruction (red circles). Grey arrows connect phenotypes before (teal) and after (red) deconstruction. Grey circles with a black outline belong to mature phenotypes that never deconstructed. The area of a circle is proportional to the number mature organisms with this phenotype. The upper triangle indicates waiting. For each mature phenotype (after deconstruction) below the diagonal the corresponding square above the diagonal highlights the amount of waiting. The color intensity is proportional to the amount of waiting. Black squares indicate phenotypes that waited all of ontogeny (i.e. 20 time periods) and white squares phenotypes that never waited.

Diminishing rewards and increasing penalties

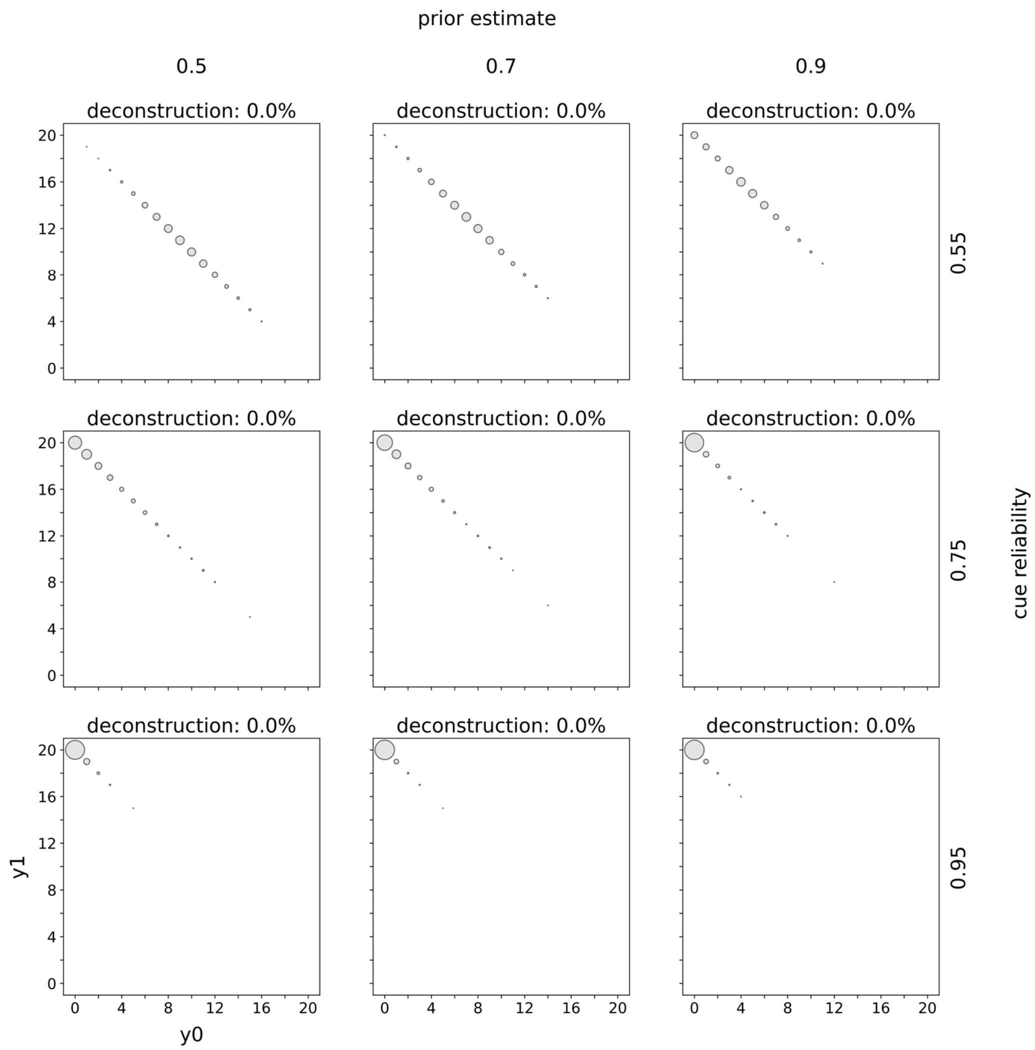

Figure S2.44: Distributions of mature phenotypes. Distributions of mature phenotypes are shown for a model with incremental deconstruction and diminishing rewards and increasing penalties. Columns indicate the prior estimate of being in E_1 and rows indicate the cue reliability. The populations of mature phenotypes have been simulated in E_1. The title of each panel indicates the percentage of mature phenotypes that have deconstructed at some point during ontogeny. Within each panel the horizontal axis indicates the number of specializations towards E_0 and the vertical axis towards E_1. The lower triangle indicates how much mature phenotypes have constructed (teal circles) and what their phenotype looked like after deconstruction (red circles). Grey arrows connect phenotypes before (teal) and after (red) deconstruction. Grey circles with a black outline belong to mature phenotypes that never deconstructed. The area of a circle is proportional to the number mature organisms with this phenotype. The upper triangle indicates waiting. For each mature phenotype (after deconstruction) below the diagonal the corresponding square above the diagonal highlights the amount of waiting. The color intensity is proportional to the amount of waiting. Black squares indicate phenotypes that waited all of ontogeny (i.e. 20 time periods) and white squares phenotypes that never waited.

Diminishing rewards and diminishing penalties

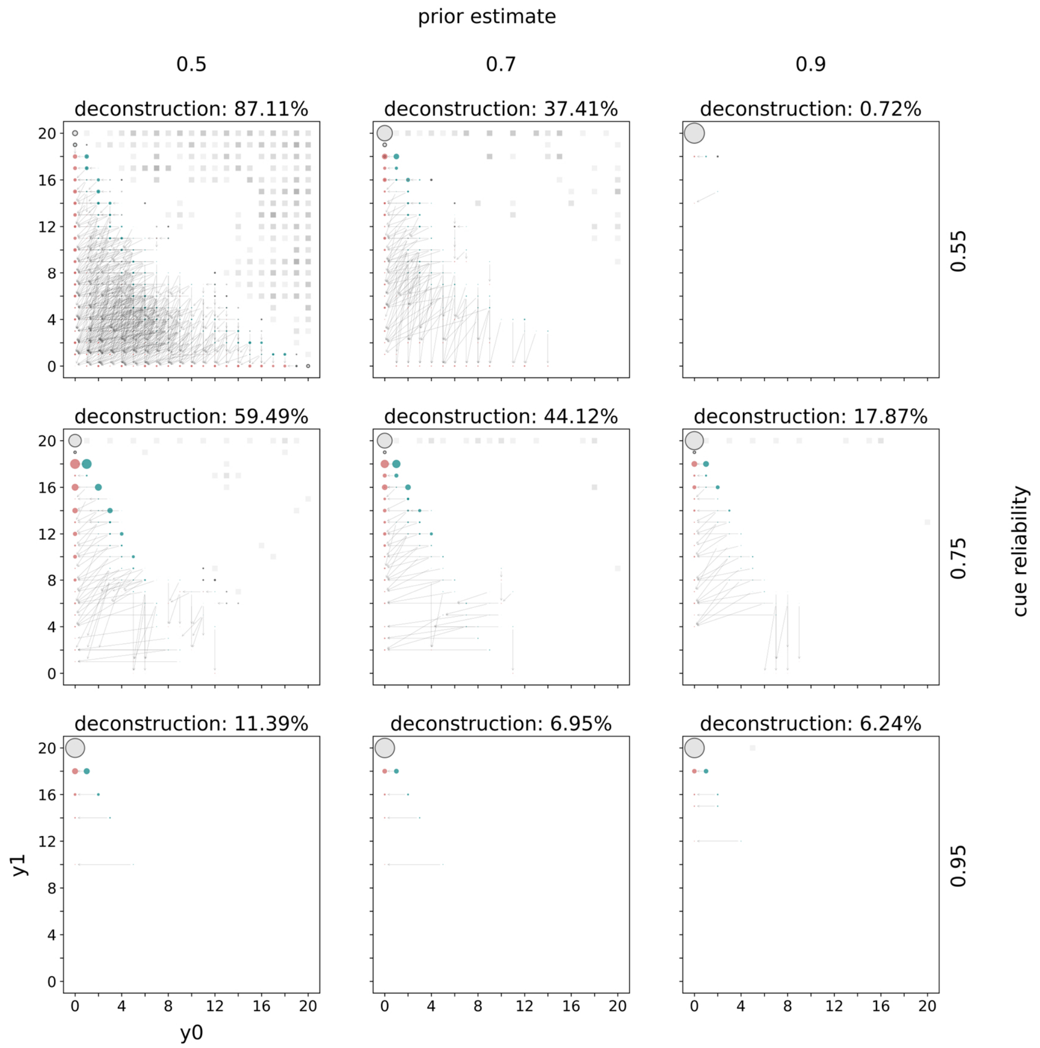

Figure S2.45: Distributions of mature phenotypes. Distributions of mature phenotypes are shown for a model with incremental deconstruction and diminishing rewards and diminishing penalties. Columns indicate the prior estimate of being in E_1 and rows indicate the cue reliability. The populations of mature phenotypes have been simulated in E_1. The title of each panel indicates the percentage of mature phenotypes that have deconstructed at some point during ontogeny. Within each panel the horizontal axis indicates the number of specializations towards E_0 and the vertical axis towards E_1. The lower triangle indicates how much mature phenotypes have constructed (teal circles) and what their phenotype looked like after deconstruction (red circles). Grey arrows connect phenotypes before (teal) and after (red) deconstruction. Grey circles with a black outline belong to mature phenotypes that never deconstructed. The area of a circle is proportional to the number mature organisms with this phenotype. The upper triangle indicates waiting. For each mature phenotype (after deconstruction) below the diagonal the corresponding square above the diagonal highlights the amount of waiting. The color intensity is proportional to the amount of waiting. Black squares indicate phenotypes that waited all of ontogeny (i.e. 20 time periods) and white squares phenotypes that never waited.

Distributions of mature phenotypes (complete deconstruction)

Linear rewards and linear penalties

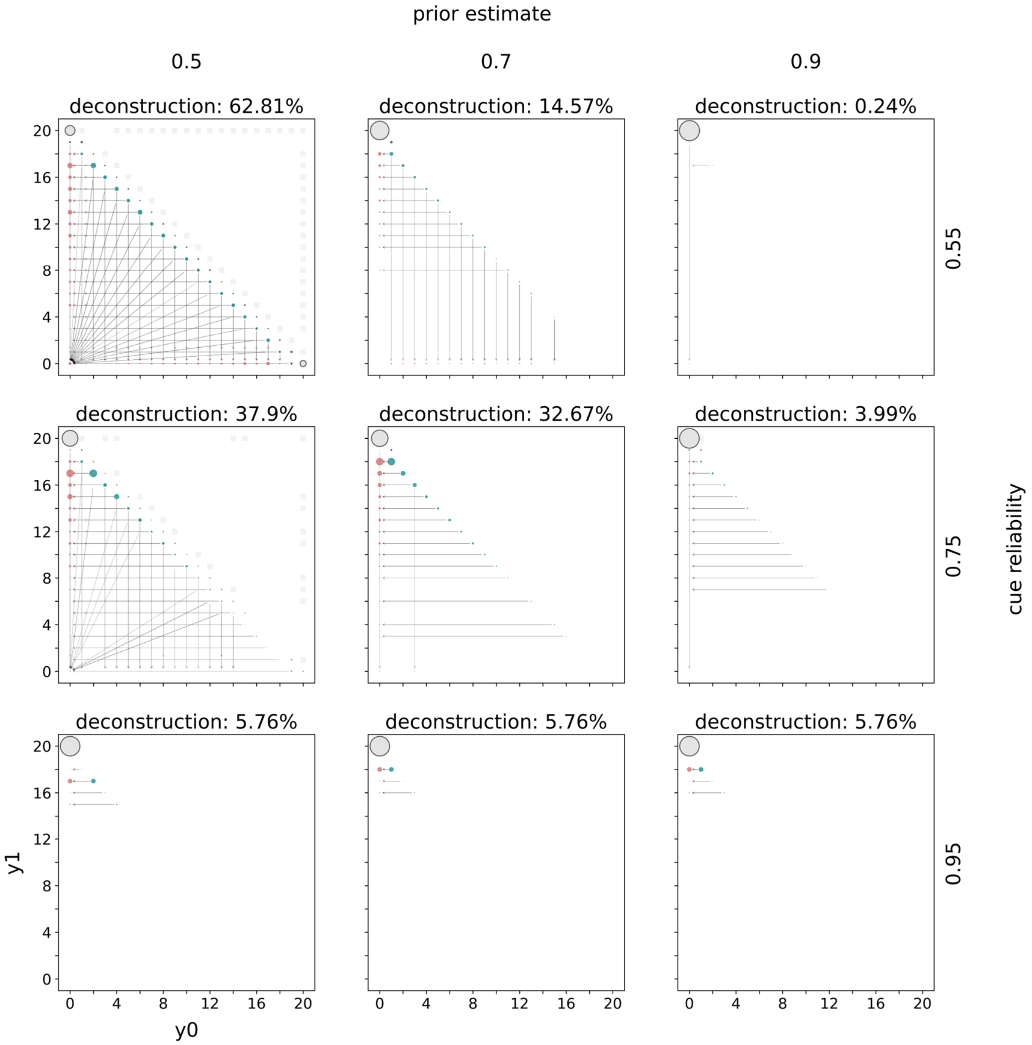

Figure S2.46: Distributions of mature phenotypes. Distributions of mature phenotypes are shown for a model with complete deconstruction and linear rewards and penalties. Columns indicate the prior estimate of being in E_1 and rows indicate the cue reliability. The populations of mature phenotypes have been simulated in E_1. The title of each panel indicates the percentage of mature phenotypes that have deconstructed at some point during ontogeny. Within each panel the horizontal axis indicates the number of specializations towards E_0 and the vertical axis towards E_1. The lower triangle indicates how much mature phenotypes have constructed (teal circles) and what their phenotype looked like after deconstruction (red circles). Grey arrows connect phenotypes before (teal) and after (red) deconstruction. Grey circles with a black outline belong to mature phenotypes that never deconstructed. The area of a circle is proportional to the number mature organisms with this phenotype. The upper triangle indicates waiting. For each mature phenotype (after deconstruction) below the diagonal the corresponding square above the diagonal highlights the amount of waiting. The color intensity is proportional to the amount of waiting. Black squares indicate phenotypes that waited all of ontogeny (i.e. 20 time periods) and white squares phenotypes that never waited.

Linear rewards and increasing penalties

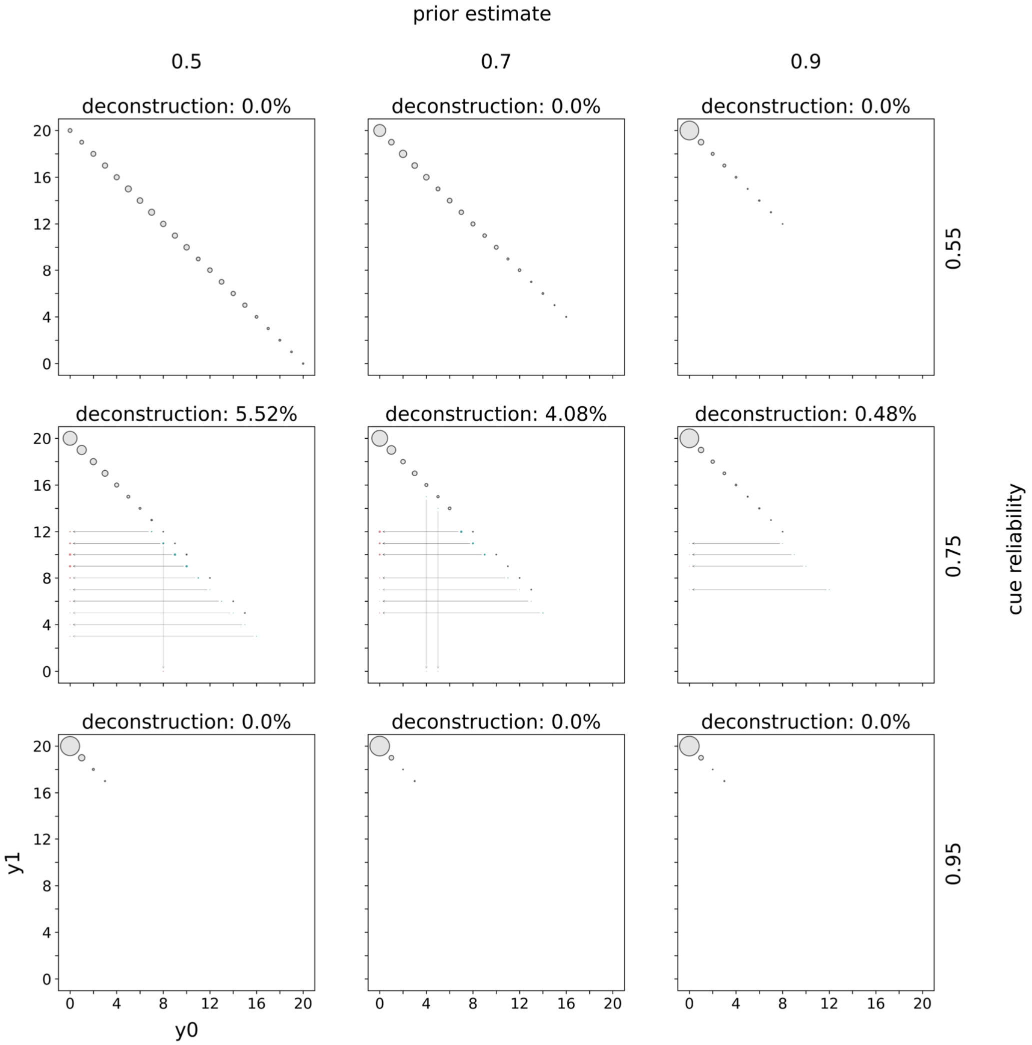

Figure S2.47: Distributions of mature phenotypes. Distributions of mature phenotypes are shown for a model with complete deconstruction and linear rewards and increasing penalties. Columns indicate the prior estimate of being in E_1 and rows indicate the cue reliability. The populations of mature phenotypes have been simulated in E_1. The title of each panel indicates the percentage of mature phenotypes that have deconstructed at some point during ontogeny. Within each panel the horizontal axis indicates the number of specializations towards E_0 and the vertical axis towards E_1. The lower triangle indicates how much mature phenotypes have constructed (teal circles) and what their phenotype looked like after deconstruction (red circles). Grey arrows connect phenotypes before (teal) and after (red) deconstruction. Grey circles with a black outline belong to mature phenotypes that never deconstructed. The area of a circle is proportional to the number mature organisms with this phenotype. The upper triangle indicates waiting. For each mature phenotype (after deconstruction) below the diagonal the corresponding square above the diagonal highlights the amount of waiting. The color intensity is proportional to the amount of waiting. Black squares indicate phenotypes that waited all of ontogeny (i.e. 20 time periods) and white squares phenotypes that never waited.

Linear rewards and diminishing penalties

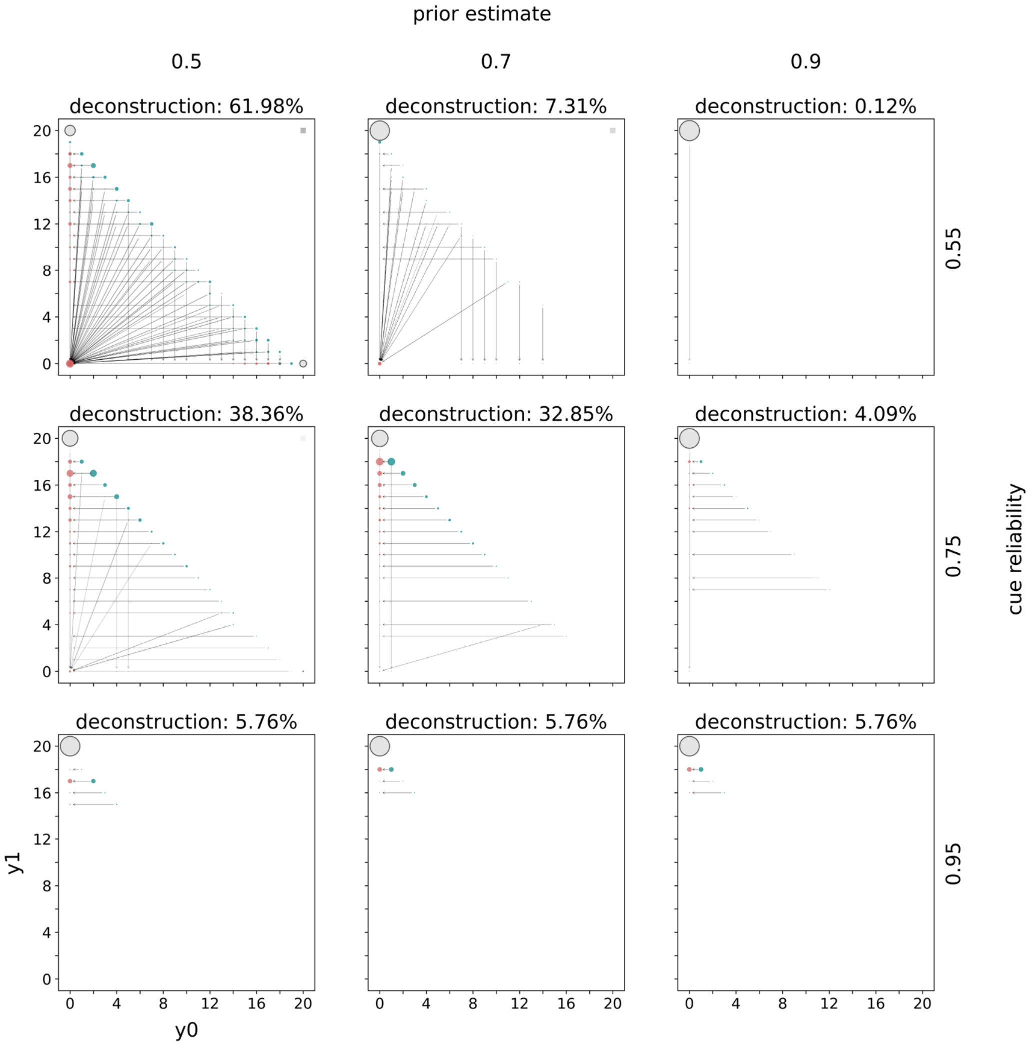

Figure S2.48: Distributions of mature phenotypes. Distributions of mature phenotypes are shown for a model with complete deconstruction and linear rewards and diminishing penalties. Columns indicate the prior estimate of being in E_1 and rows indicate the cue reliability. The populations of mature phenotypes have been simulated in E_1. The title of each panel indicates the percentage of mature phenotypes that have deconstructed at some point during ontogeny. Within each panel the horizontal axis indicates the number of specializations towards E_0 and the vertical axis towards E_1. The lower triangle indicates how much mature phenotypes have constructed (teal circles) and what their phenotype looked like after deconstruction (red circles). Grey arrows connect phenotypes before (teal) and after (red) deconstruction. Grey circles with a black outline belong to mature phenotypes that never deconstructed. The area of a circle is proportional to the number mature organisms with this phenotype. The upper triangle indicates waiting. For each mature phenotype (after deconstruction) below the diagonal the corresponding square above the diagonal highlights the amount of waiting. The color intensity is proportional to the amount of waiting. Black squares indicate phenotypes that waited all of ontogeny (i.e. 20 time periods) and white squares phenotypes that never waited.

Increasing rewards and linear penalties

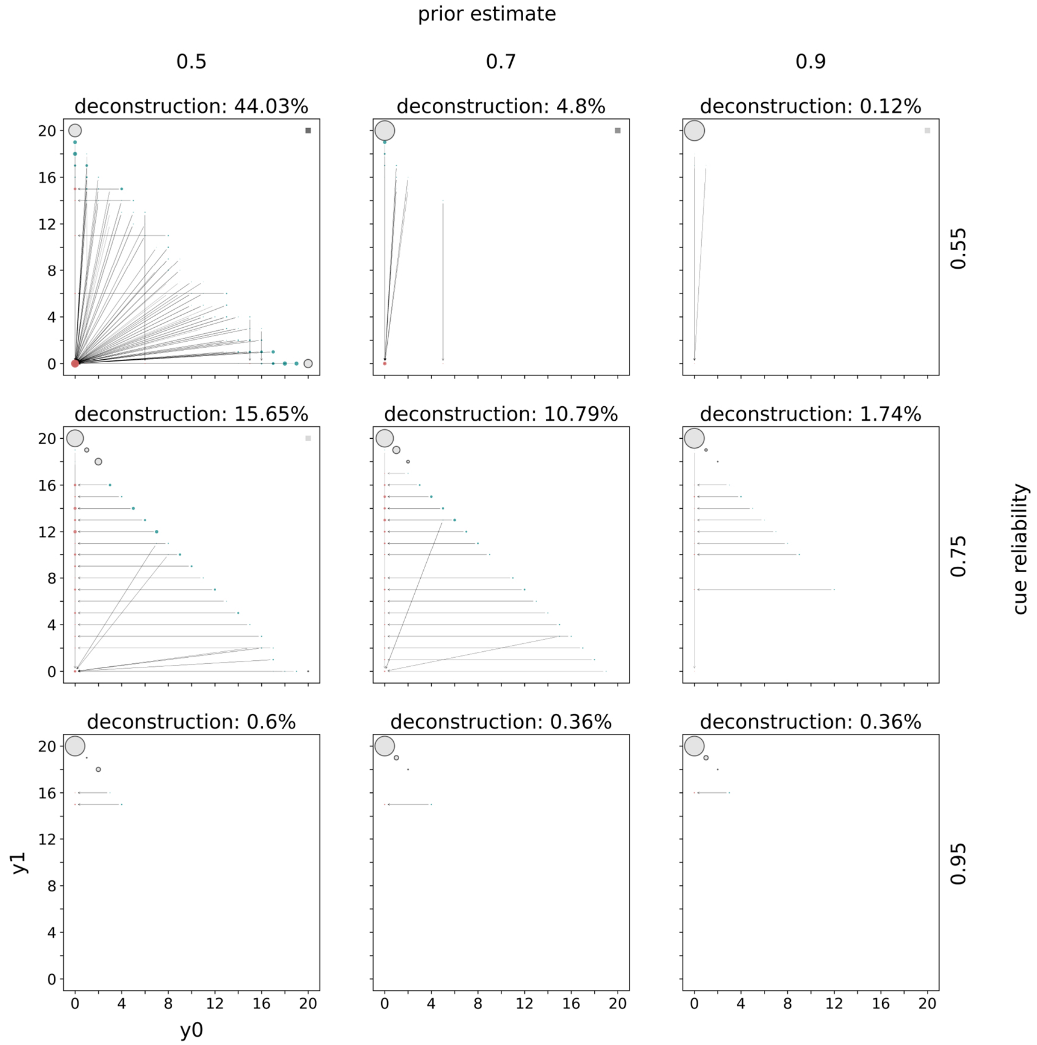

Figure S2.49: Distributions of mature phenotypes. Distributions of mature phenotypes are shown for a model with complete deconstruction and increasing rewards and linear penalties. Columns indicate the prior estimate of being in E_1 and rows indicate the cue reliability. The populations of mature phenotypes have been simulated in E_1. The title of each panel indicates the percentage of mature phenotypes that have deconstructed at some point during ontogeny. Within each panel the horizontal axis indicates the number of specializations towards E_0 and the vertical axis towards E_1. The lower triangle indicates how much mature phenotypes have constructed (teal circles) and what their phenotype looked like after deconstruction (red circles). Grey arrows connect phenotypes before (teal) and after (red) deconstruction. Grey circles with a black outline belong to mature phenotypes that never deconstructed. The area of a circle is proportional to the number mature organisms with this phenotype. The upper triangle indicates waiting. For each mature phenotype (after deconstruction) below the diagonal the corresponding square above the diagonal highlights the amount of waiting. The color intensity is proportional to the amount of waiting. Black squares indicate phenotypes that waited all of ontogeny (i.e. 20 time periods) and white squares phenotypes that never waited.

Increasing rewards and increasing penalties

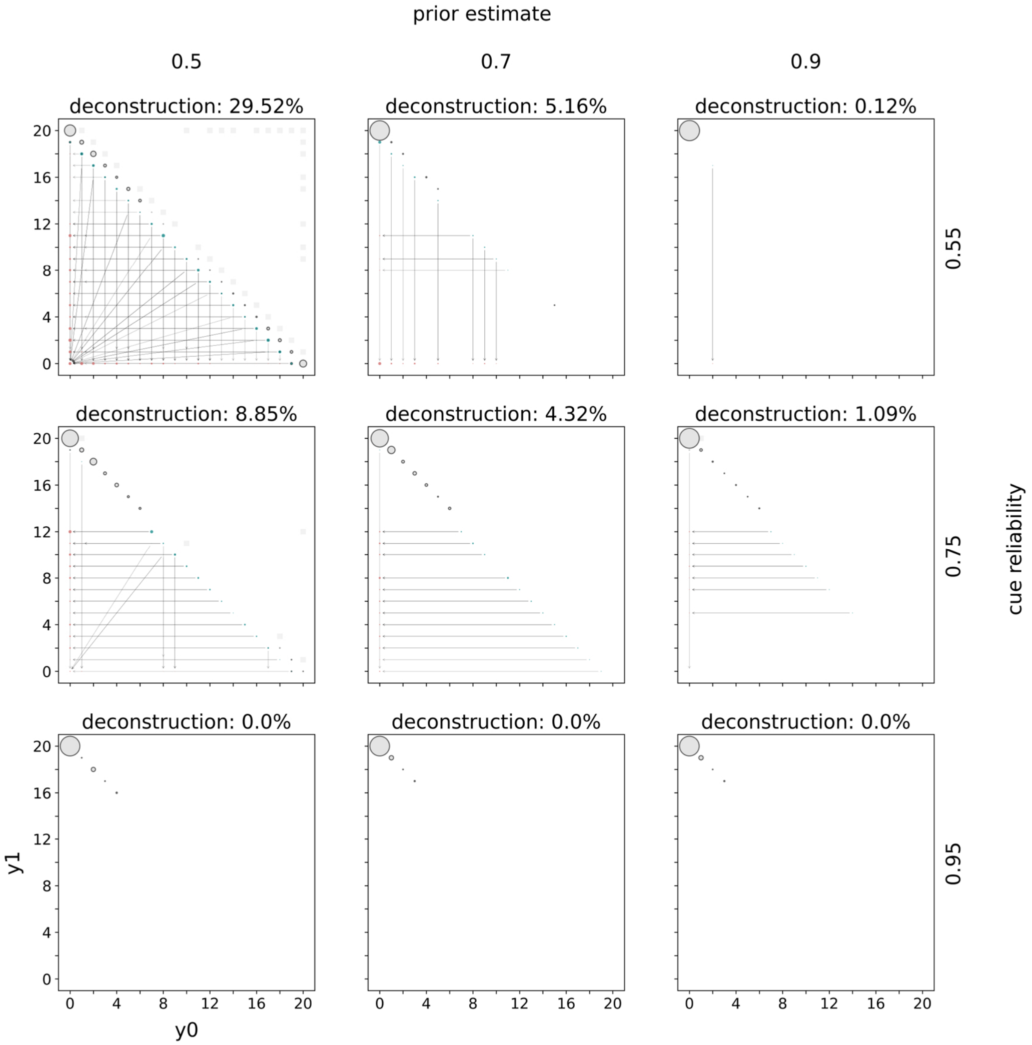

Figure S2.50: Distributions of mature phenotypes. Distributions of mature phenotypes are shown for a model with complete deconstruction and increasing rewards and increasing penalties. Columns indicate the prior estimate of being in E_1 and rows indicate the cue reliability. The populations of mature phenotypes have been simulated in E_1. The title of each panel indicates the percentage of mature phenotypes that have deconstructed at some point during ontogeny. Within each panel the horizontal axis indicates the number of specializations towards E_0 and the vertical axis towards E_1. The lower triangle indicates how much mature phenotypes have constructed (teal circles) and what their phenotype looked like after deconstruction (red circles). Grey arrows connect phenotypes before (teal) and after (red) deconstruction. Grey circles with a black outline belong to mature phenotypes that never deconstructed. The area of a circle is proportional to the number mature organisms with this phenotype. The upper triangle indicates waiting. For each mature phenotype (after deconstruction) below the diagonal the corresponding square above the diagonal highlights the amount of waiting. The color intensity is proportional to the amount of waiting. Black squares indicate phenotypes that waited all of ontogeny (i.e. 20 time periods) and white squares phenotypes that never waited.

Increasing rewards and diminishing penalties

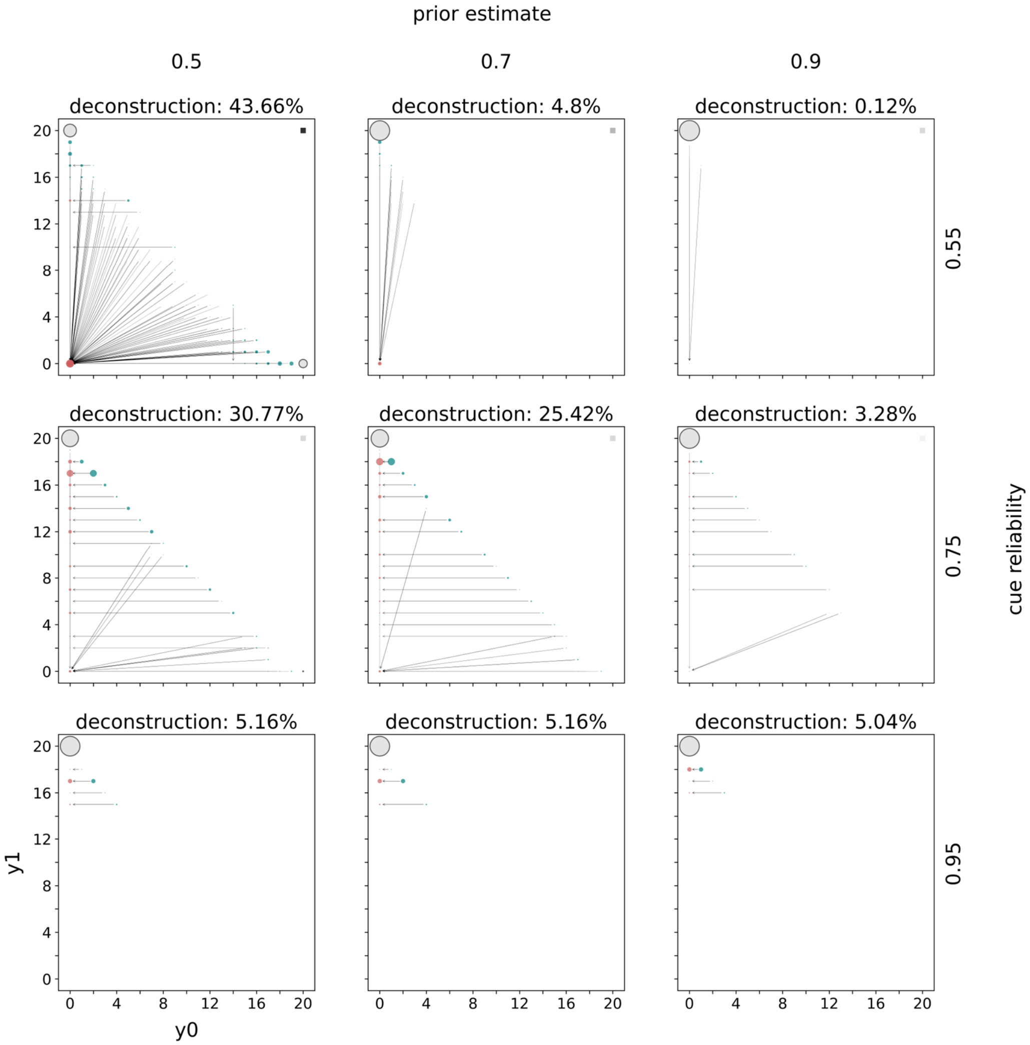

Figure S2.51: Distributions of mature phenotypes. Distributions of mature phenotypes are shown for a model with complete deconstruction and increasing rewards and diminishing penalties. Columns indicate the prior estimate of being in E_1 and rows indicate the cue reliability. The populations of mature phenotypes have been simulated in E_1. The title of each panel indicates the percentage of mature phenotypes that have deconstructed at some point during ontogeny. Within each panel the horizontal axis indicates the number of specializations towards E_0 and the vertical axis towards E_1. The lower triangle indicates how much mature phenotypes have constructed (teal circles) and what their phenotype looked like after deconstruction (red circles). Grey arrows connect phenotypes before (teal) and after (red) deconstruction. Grey circles with a black outline belong to mature phenotypes that never deconstructed. The area of a circle is proportional to the number mature organisms with this phenotype. The upper triangle indicates waiting. For each mature phenotype (after deconstruction) below the diagonal the corresponding square above the diagonal highlights the amount of waiting. The color intensity is proportional to the amount of waiting. Black squares indicate phenotypes that waited all of ontogeny (i.e. 20 time periods) and white squares phenotypes that never waited.

Diminishing rewards and linear penalties

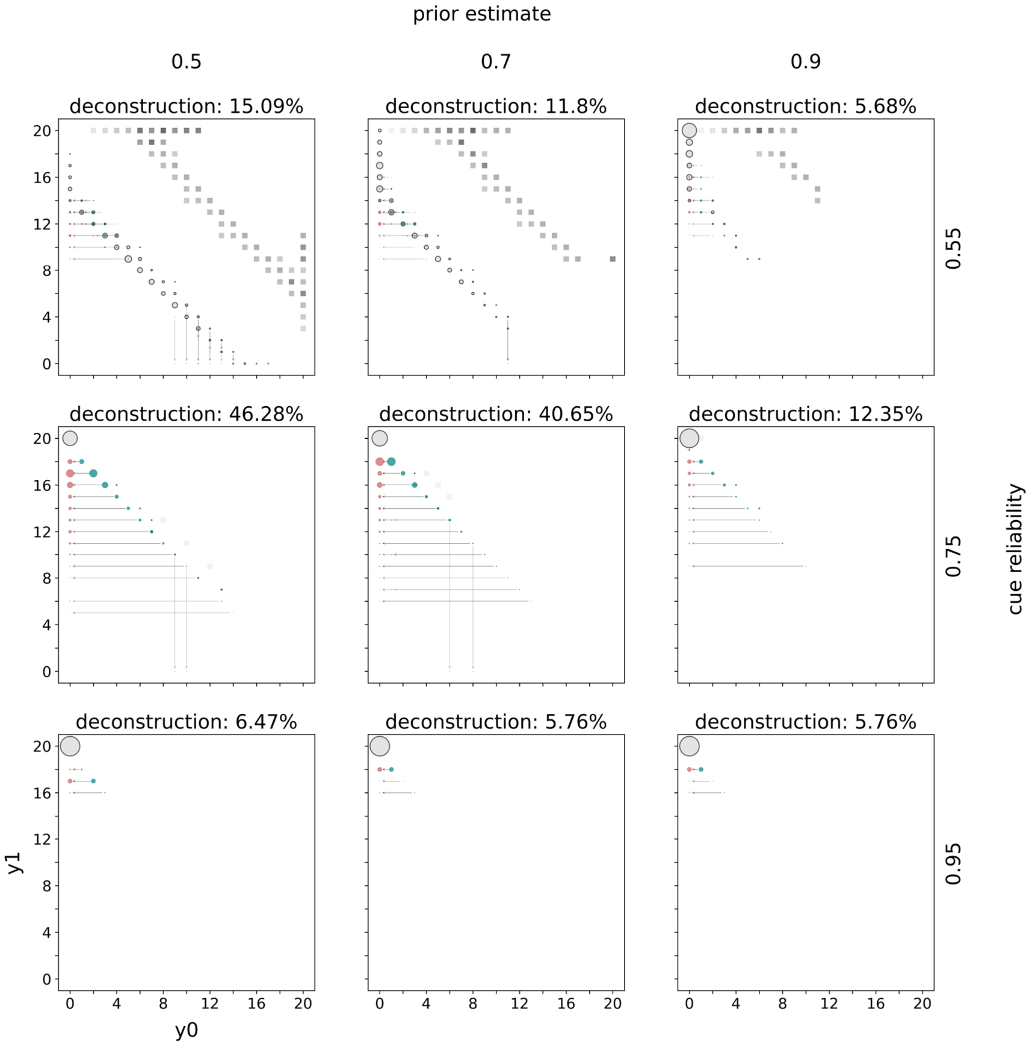

Figure S2.52: Distributions of mature phenotypes. Distributions of mature phenotypes are shown for a model with complete deconstruction and diminishing rewards and linear penalties. Columns indicate the prior estimate of being in E_1 and rows indicate the cue reliability. The populations of mature phenotypes have been simulated in E_1. The title of each panel indicates the percentage of mature phenotypes that have deconstructed at some point during ontogeny. Within each panel the horizontal axis indicates the number of specializations towards E_0 and the vertical axis towards E_1. The lower triangle indicates how much mature phenotypes have constructed (teal circles) and what their phenotype looked like after deconstruction (red circles). Grey arrows connect phenotypes before (teal) and after (red) deconstruction. Grey circles with a black outline belong to mature phenotypes that never deconstructed. The area of a circle is proportional to the number mature organisms with this phenotype. The upper triangle indicates waiting. For each mature phenotype (after deconstruction) below the diagonal the corresponding square above the diagonal highlights the amount of waiting. The color intensity is proportional to the amount of waiting. Black squares indicate phenotypes that waited all of ontogeny (i.e. 20 time periods) and white squares phenotypes that never waited.

Diminishing rewards and increasing penalties

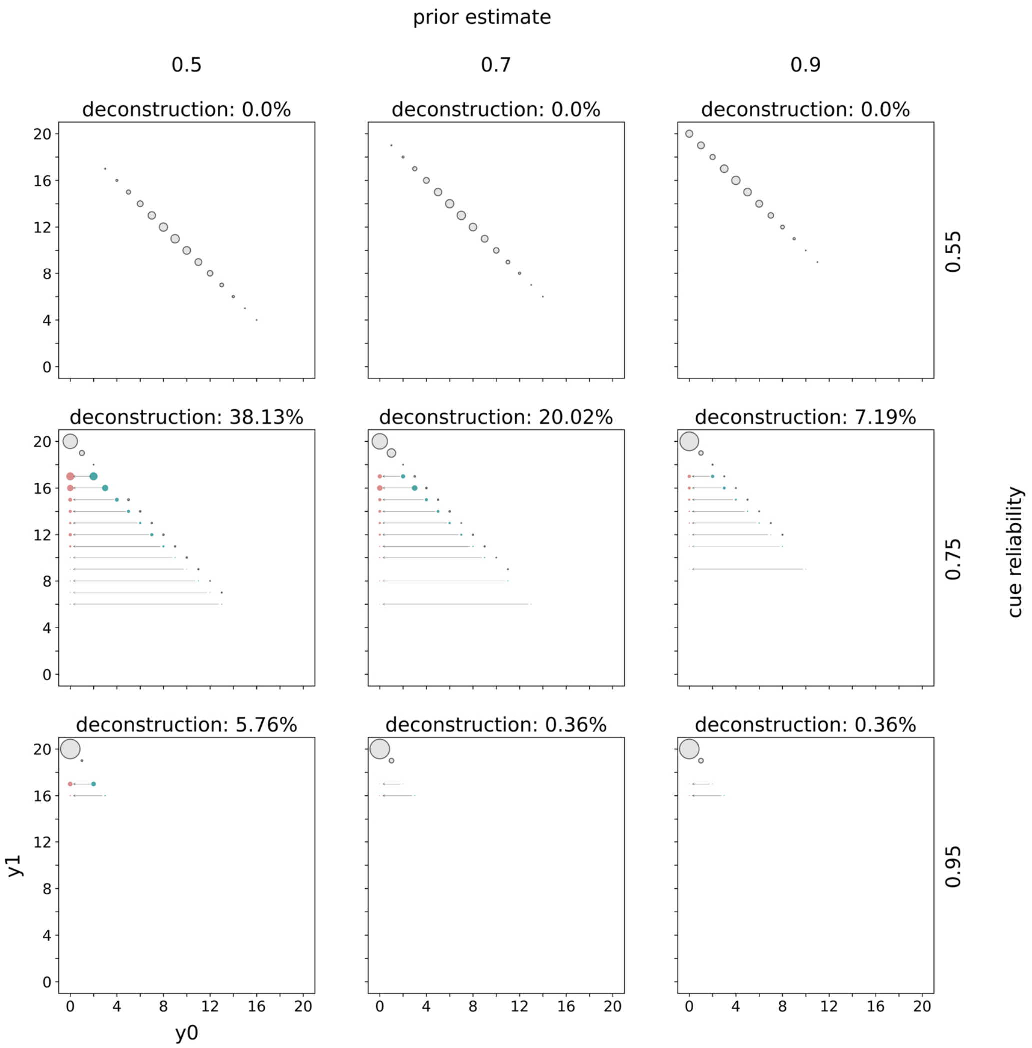

Figure S2.53: Distributions of mature phenotypes. Distributions of mature phenotypes are shown for a model with complete deconstruction and diminishing rewards and increasing penalties. Columns indicate the prior estimate of being in E_1 and rows indicate the cue reliability. The populations of mature phenotypes have been simulated in E_1. The title of each panel indicates the percentage of mature phenotypes that have deconstructed at some point during ontogeny. Within each panel the horizontal axis indicates the number of specializations towards E_0 and the vertical axis towards E_1. The lower triangle indicates how much mature phenotypes have constructed (teal circles) and what their phenotype looked like after deconstruction (red circles). Grey arrows connect phenotypes before (teal) and after (red) deconstruction. Grey circles with a black outline belong to mature phenotypes that never deconstructed. The area of a circle is proportional to the number mature organisms with this phenotype. The upper triangle indicates waiting. For each mature phenotype (after deconstruction) below the diagonal the corresponding square above the diagonal highlights the amount of waiting. The color intensity is proportional to the amount of waiting. Black squares indicate phenotypes that waited all of ontogeny (i.e. 20 time periods) and white squares phenotypes that never waited.

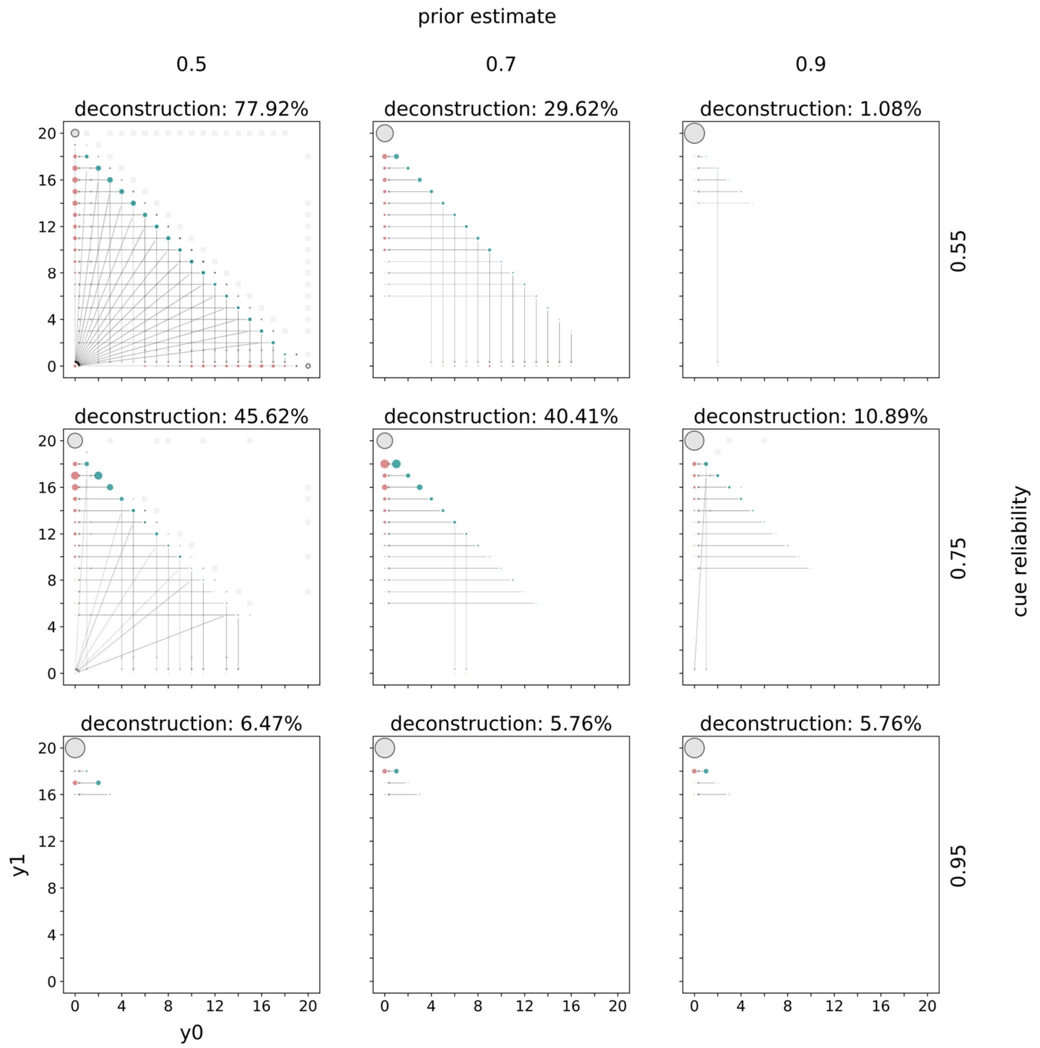

Figure S2.54: Distributions of mature phenotypes. Distributions of mature phenotypes are shown for a model with complete deconstruction and diminishing rewards and diminishing penalties. Columns indicate the prior estimate of being in E_1 and rows indicate the cue reliability. The populations of mature phenotypes have been simulated in E_1. The title of each panel indicates the percentage of mature phenotypes that have deconstructed at some point during ontogeny. Within each panel the horizontal axis indicates the number of specializations towards E_0 and the vertical axis towards E_1. The lower triangle indicates how much mature phenotypes have constructed (teal circles) and what their phenotype looked like after deconstruction (red circles). Grey arrows connect phenotypes before (teal) and after (red) deconstruction. Grey circles with a black outline belong to mature phenotypes that never deconstructed. The area of a circle is proportional to the number mature organisms with this phenotype. The upper triangle indicates waiting. For each mature phenotype (after deconstruction) below the diagonal the corresponding square above the diagonal highlights the amount of waiting. The color intensity is proportional to the amount of waiting. Black squares indicate phenotypes that waited all of ontogeny (i.e. 20 time periods) and white squares phenotypes that never waited.

Normalized fitness (incremental and complete deconstruction combined)

Linear rewards and linear penalties

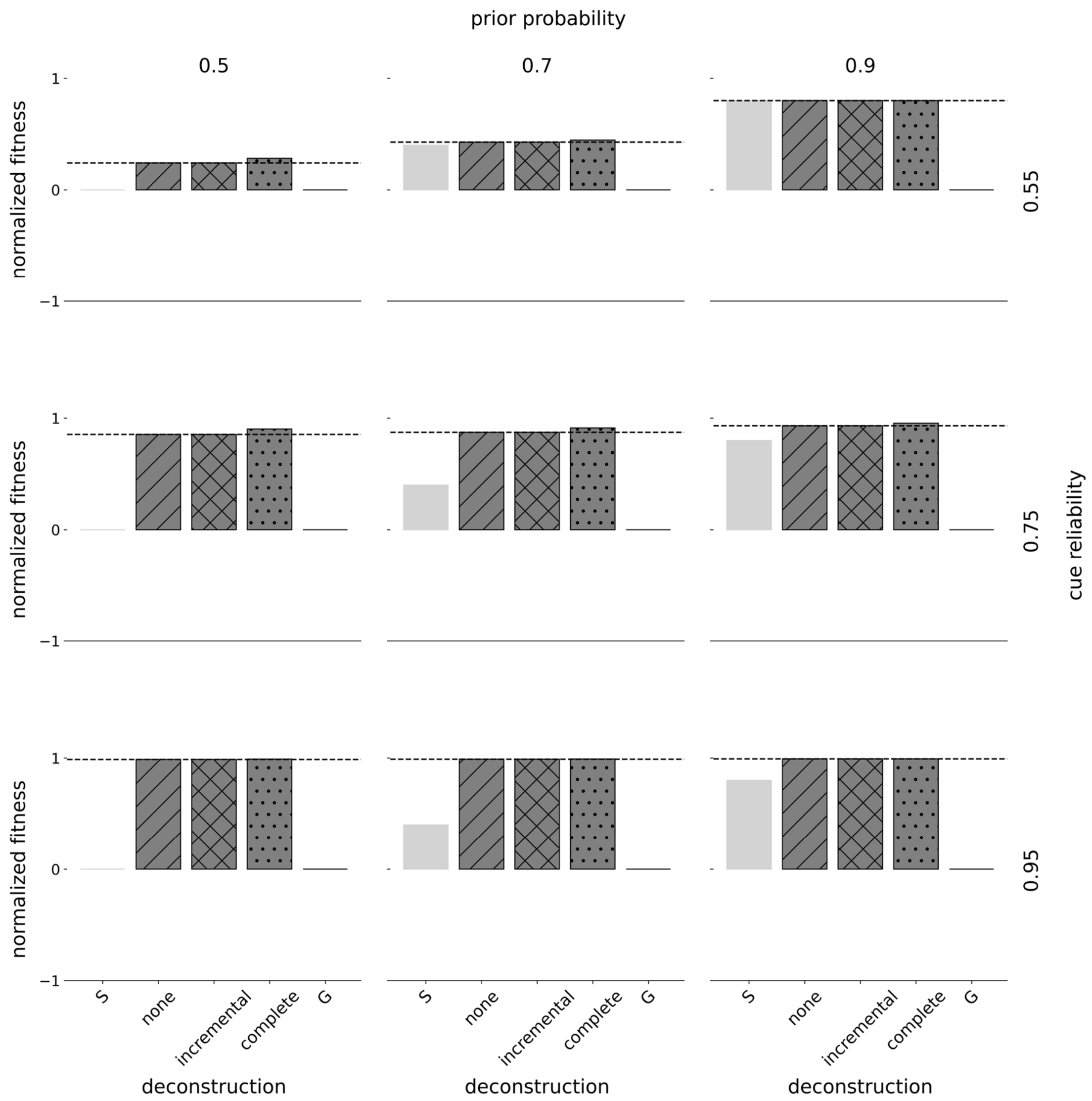

Figure S2.55: Normalized fitness. Normalized fitness is shown for linear rewards and linear penalties. Columns indicate the prior estimate of being in E_1 and rows indicate the cue reliability. Within each panel, the horizontal axis denotes the type of strategy where 'S' corresponds to a pure specialist strategy, 'O' to an optimal policy, and 'G' to a pure generalist strategy. The horizontal axis denotes fitness differences from baseline (corresponding to 0), normalized to range between -1 and 1. We show fitness of three different optimal policies: without deconstruction ('none'), with incremental deconstruction ('incremental'), and complete deconstruction ('complete'). Specialists always fully specialize according to the prior distribution. When priors are uninformative (0.5), half the population fully specializes towards P_0 and the other one towards P_1. Generalists always specialize halfway towards either phenotypic target.

Linear rewards and increasing penalties

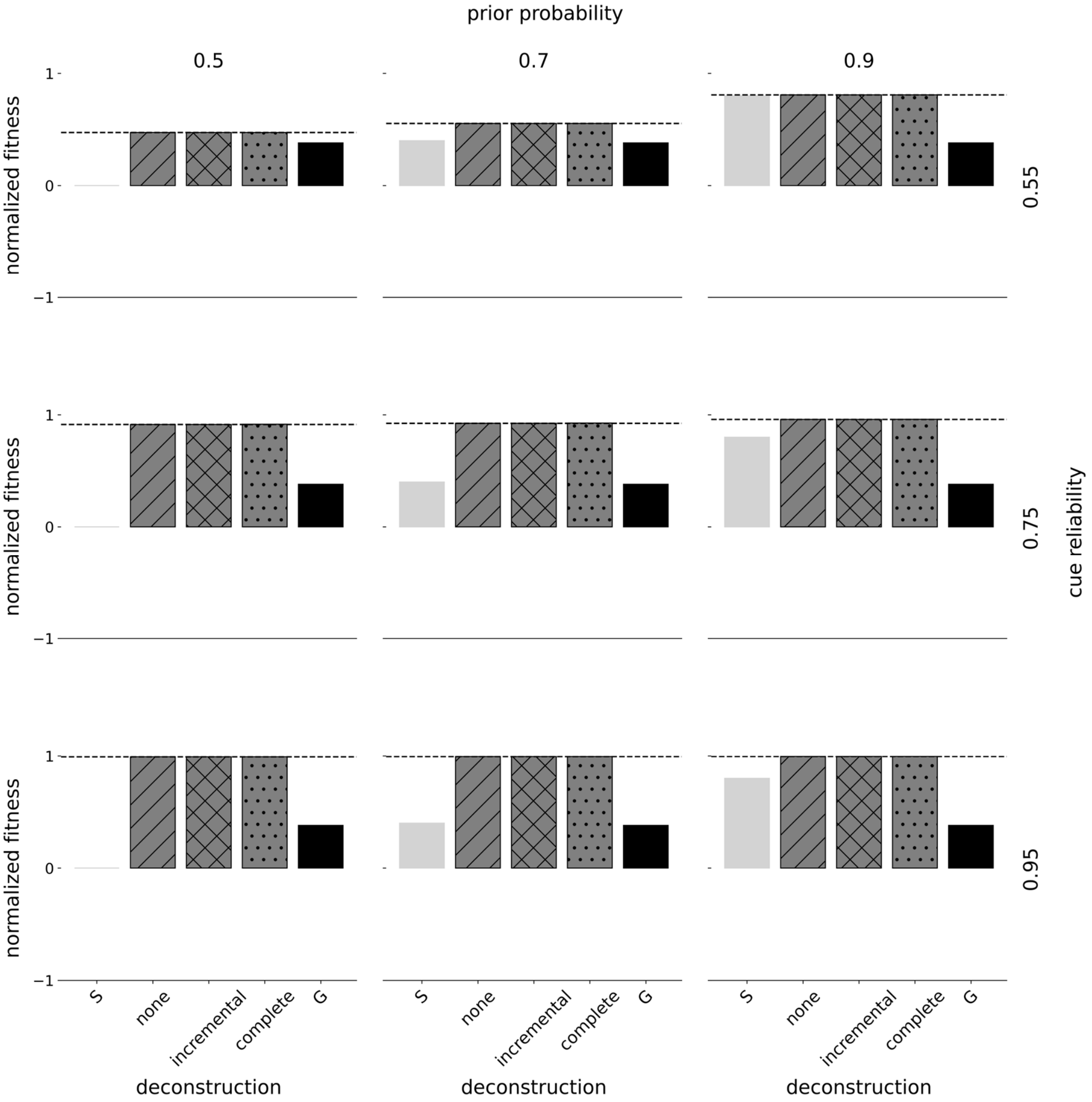

Figure S2.56: Normalized fitness. Normalized fitness is shown for linear rewards and increasing penalties. Columns indicate the prior estimate of being in E_1 and rows indicate the cue reliability. Within each panel, the horizontal axis denotes the type of strategy where ‘S’ corresponds to a pure specialist strategy, ‘O’ to an optimal policy, and ‘G’ to a pure generalist strategy. The horizontal axis denotes fitness differences from baseline (corresponding to 0), normalized to range between -1 and 1. We show fitness of three different optimal policies: without deconstruction (‘none’), with incremental deconstruction (‘incremental’), and complete deconstruction (‘complete’). Specialists always fully specialize according to the prior distribution. When priors are uninformative (0.5), half the population fully specializes towards P_0 and the other one towards P_1. Generalists always specialize halfway towards either phenotypic target.

Linear rewards and diminishing penalties

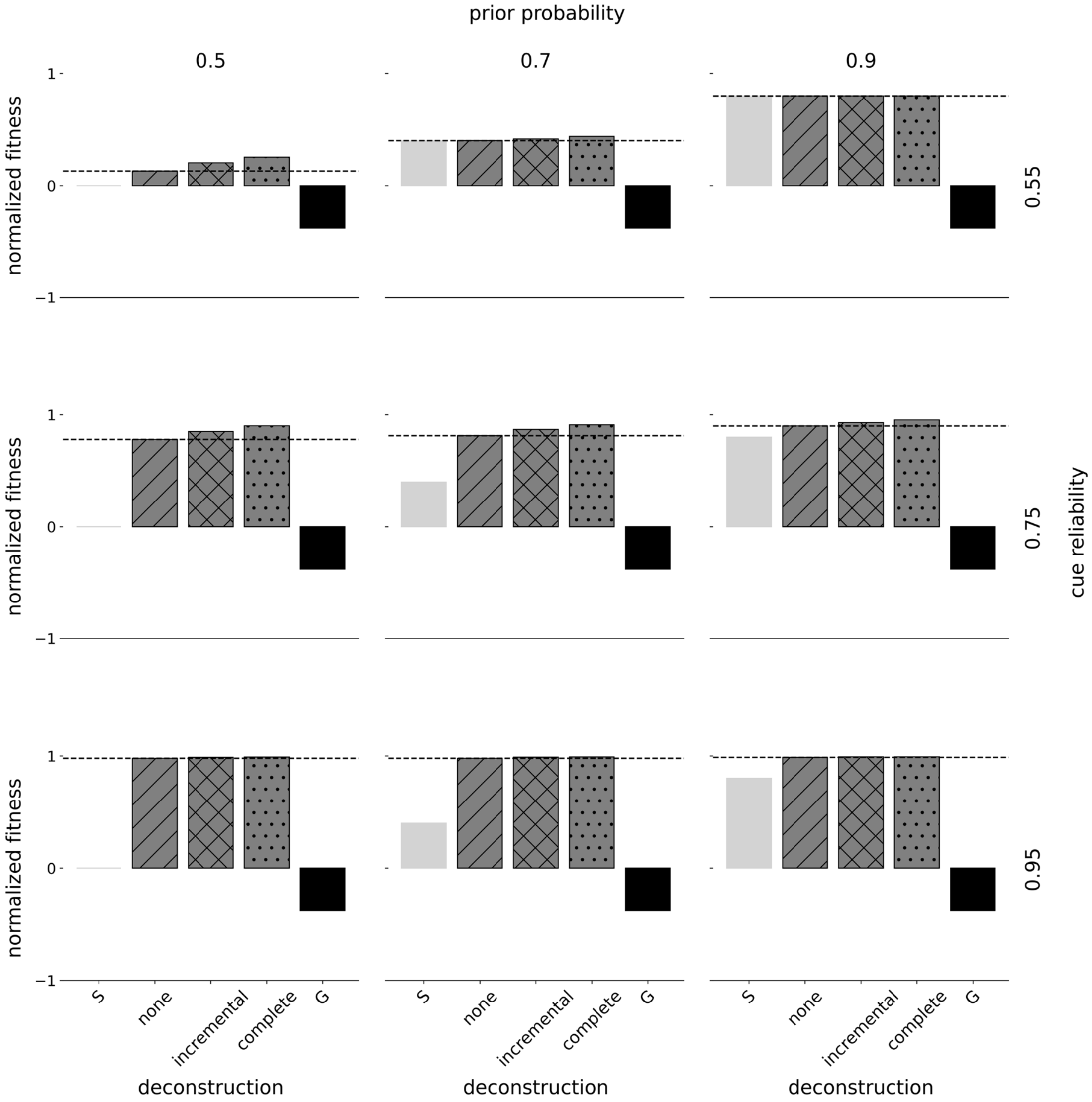

Figure S2.57: Normalized fitness. Normalized fitness is shown for linear rewards and diminishing penalties. Columns indicate the prior estimate of being in E_1 and rows indicate the cue reliability. Within each panel, the horizontal axis denotes the type of strategy where 'S' corresponds to a pure specialist strategy, 'O' to an optimal policy, and 'G' to a pure generalist strategy. The horizontal axis denotes fitness differences from baseline (corresponding to 0), normalized to range between -1 and 1. We show fitness of three different optimal policies: without deconstruction ('none'), with incremental deconstruction ('incremental'), and complete deconstruction ('complete'). Specialists always fully specialize according to the prior distribution. When priors are uninformative (0.5), half the population fully specializes towards P_0 and the other one towards P_1. Generalists always specialize halfway towards either phenotypic target.

Increasing rewards and linear penalties

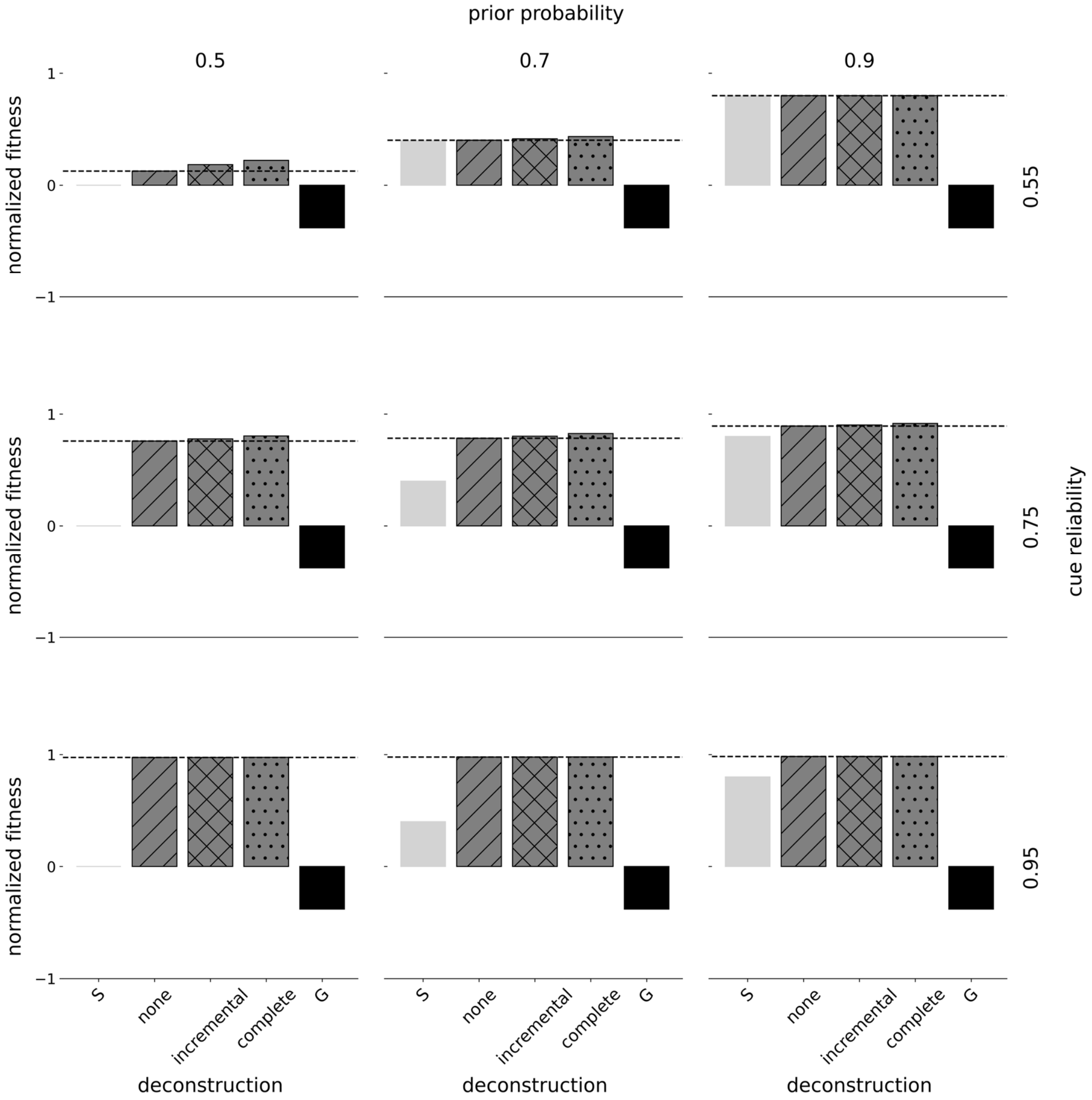

Figure S2.58: Normalized fitness. Normalized fitness is shown for increasing rewards and linear penalties. Columns indicate the prior estimate of being in E_1 and rows indicate the cue reliability. Within each panel, the horizontal axis denotes the type of strategy where ‘S’ corresponds to a pure specialist strategy, ‘O’ to an optimal policy, and ‘G’ to a pure generalist strategy. The horizontal axis denotes fitness differences from baseline (corresponding to 0), normalized to range between -1 and 1. We show fitness of three different optimal policies: without deconstruction (‘none’), with incremental deconstruction (‘incremental’), and complete deconstruction (‘complete’). Specialists always fully specialize according to the prior distribution. When priors are uninformative (0.5), half the population fully specializes towards P_0 and the other one towards P_1. Generalists always specialize halfway towards either phenotypic target.

Increasing rewards and increasing penalties

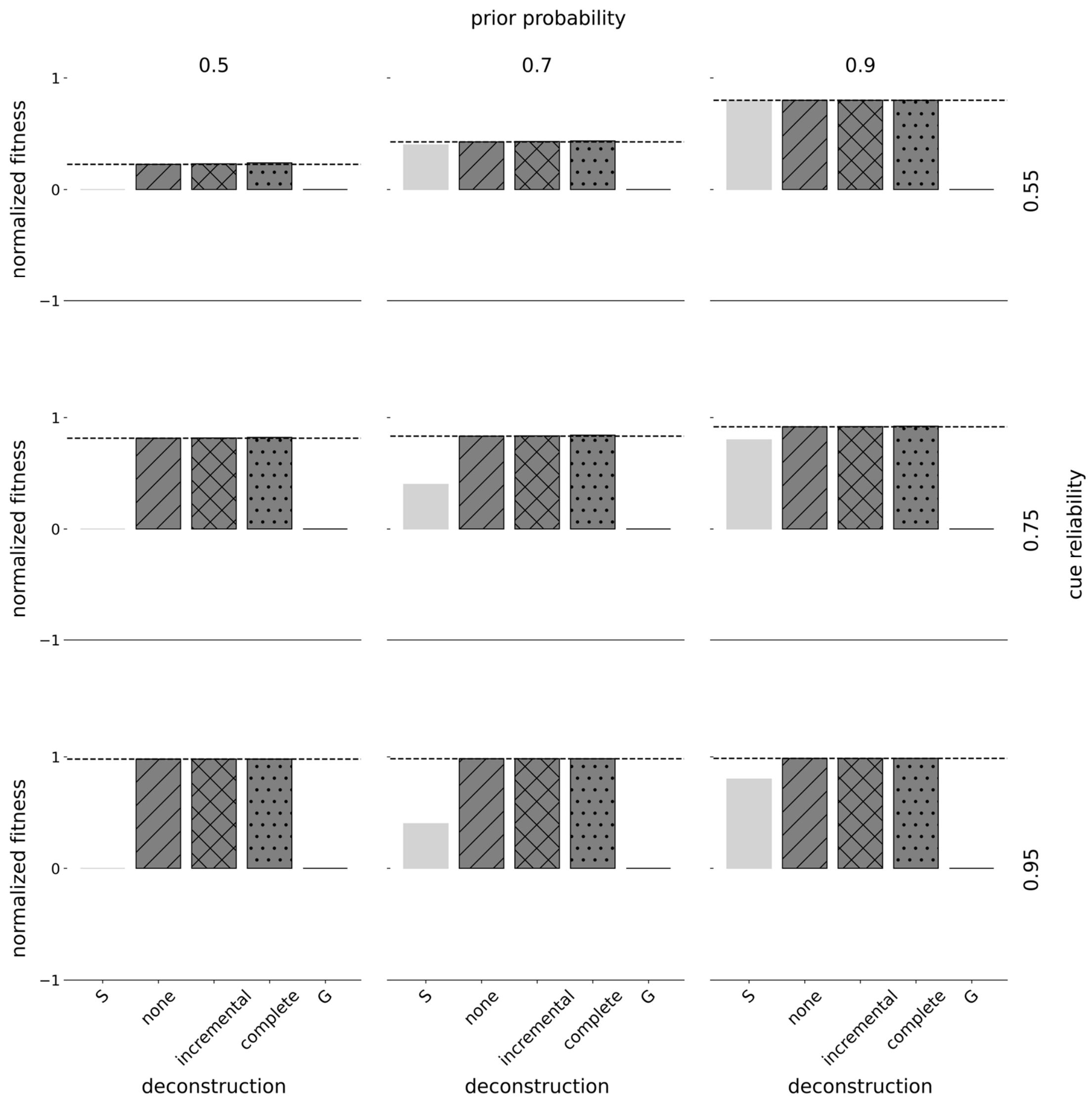

Figure S2.59: Normalized fitness. Normalized fitness is shown for increasing rewards and increasing penalties. Columns indicate the prior estimate of being in E_1 and rows indicate the cue reliability. Within each panel, the horizontal axis denotes the type of strategy where ‘S’ corresponds to a pure specialist strategy, ‘O’ to an optimal policy, and ‘G’ to a pure generalist strategy. The horizontal axis denotes fitness differences from baseline (corresponding to 0), normalized to range between -1 and 1. We show fitness of three different optimal policies: without deconstruction (‘none’), with incremental deconstruction (‘incremental’), and complete deconstruction (‘complete’). Specialists always fully specialize according to the prior distribution. When priors are uninformative (0.5), half the population fully specializes towards P_0 and the other one towards P_1. Generalists always specialize halfway towards either phenotypic target.

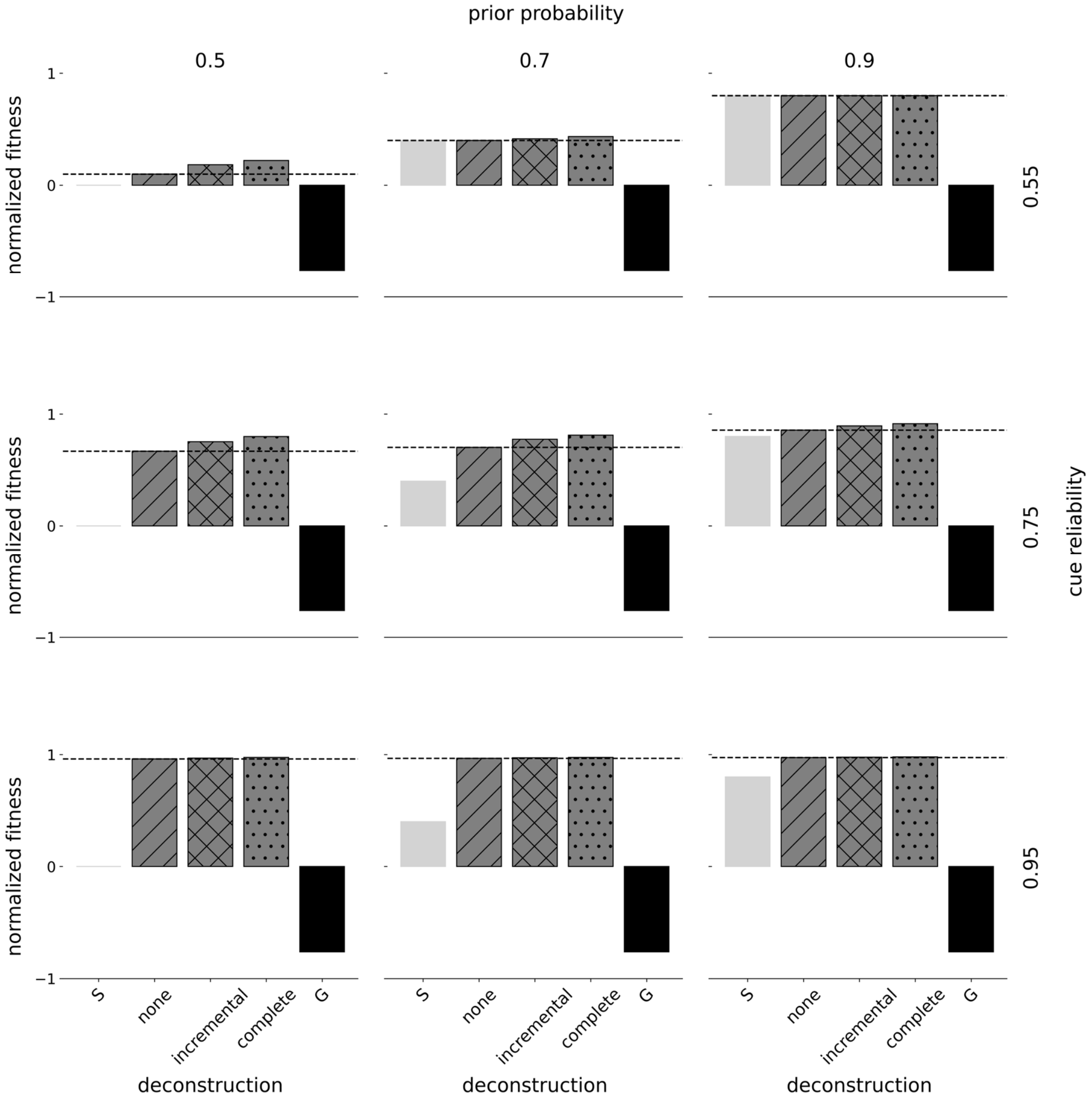

Figure S2.60: Normalized fitness. Normalized fitness is shown for increasing rewards and diminishing penalties. Columns indicate the prior estimate of being in E_1 and rows indicate the cue reliability. Within each panel, the horizontal axis denotes the type of strategy where 'S' corresponds to a pure specialist strategy, 'O' to an optimal policy, and 'G' to a pure generalist strategy. The horizontal axis denotes fitness differences from baseline (corresponding to 0), normalized to range between -1 and 1. We show fitness of three different optimal policies: without deconstruction ('none'), with incremental deconstruction ('incremental'), and complete deconstruction ('complete'). Specialists always fully specialize according to the prior distribution. When priors are uninformative (0.5), half the population fully specializes towards P_0 and the other one towards P_1. Generalists always specialize halfway towards either phenotypic target.

Diminishing rewards and linear penalties

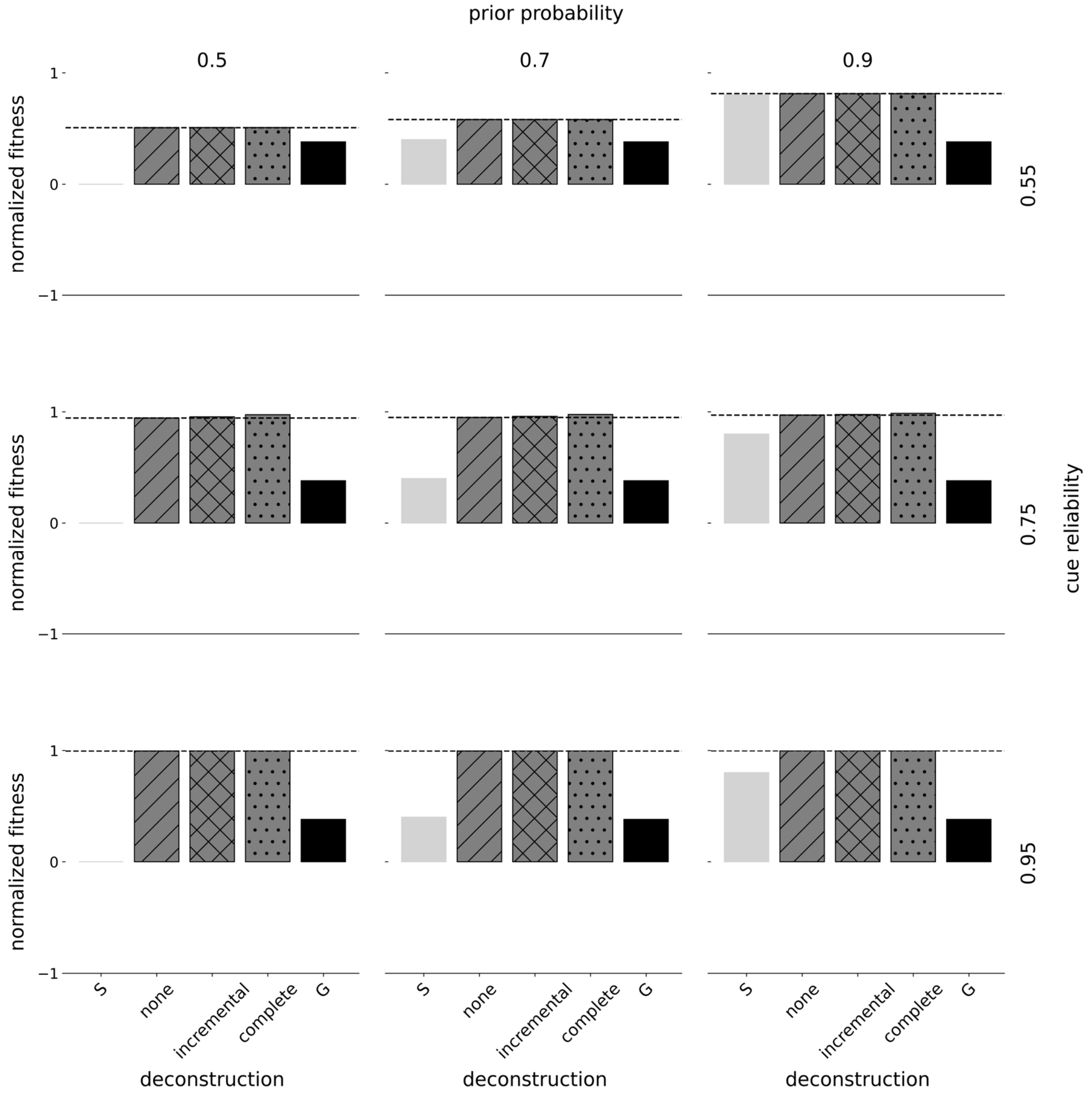

Figure S2.61: Normalized fitness. Normalized fitness is shown for diminishing rewards and linear penalties. Columns indicate the prior estimate of being in E_1 and rows indicate the cue reliability. Within each panel, the horizontal axis denotes the type of strategy where 'S' corresponds to a pure specialist strategy, 'O' to an optimal policy, and 'G' to a pure generalist strategy. The horizontal axis denotes fitness differences from baseline (corresponding to 0), normalized to range between -1 and 1. We show fitness of three different optimal policies: without deconstruction ('none'), with incremental deconstruction ('incremental'), and complete deconstruction ('complete'). Specialists always fully specialize according to the prior distribution. When priors are uninformative (0.5), half the population fully specializes towards P_0 and the other one towards P_1. Generalists always specialize halfway towards either phenotypic target.

Diminishing rewards and increasing penalties

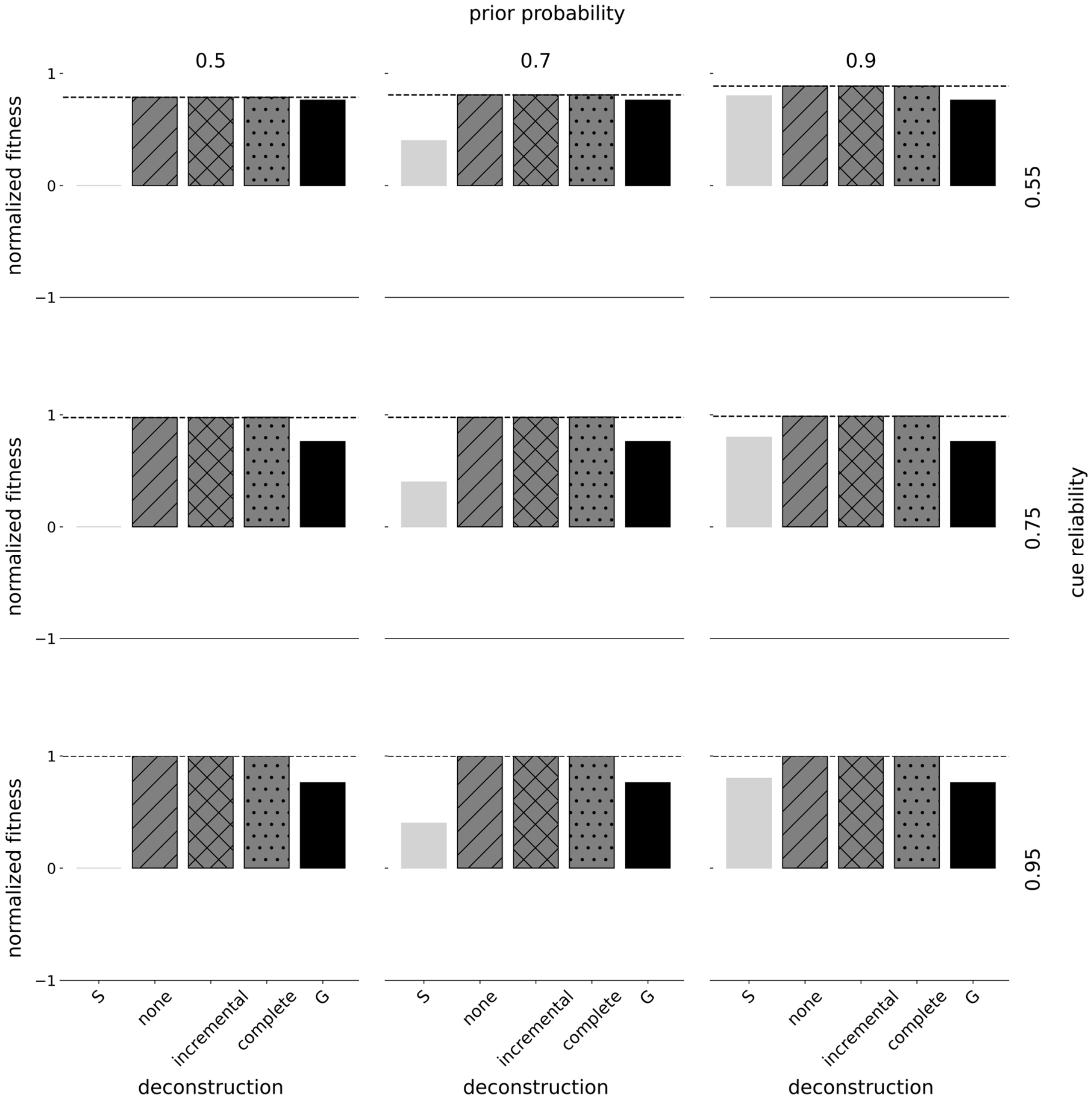

Figure S2.62: Normalized fitness. Normalized fitness is shown for diminishing rewards and increasing penalties. Columns indicate the prior estimate of being in E_1 and rows indicate the cue reliability. Within each panel, the horizontal axis denotes the type of strategy where 'S' corresponds to a pure specialist strategy, 'O' to an optimal policy, and 'G' to a pure generalist strategy. The horizontal axis denotes fitness differences from baseline (corresponding to 0), normalized to range between -1 and 1. We show fitness of three different optimal policies: without deconstruction ('none'), with incremental deconstruction ('incremental'), and complete deconstruction ('complete'). Specialists always fully specialize according to the prior distribution. When priors are uninformative (0.5), half the population fully specializes towards P_0 and the other one towards P_1. Generalists always specialize halfway towards either phenotypic target.

Diminishing rewards and diminishing penalties

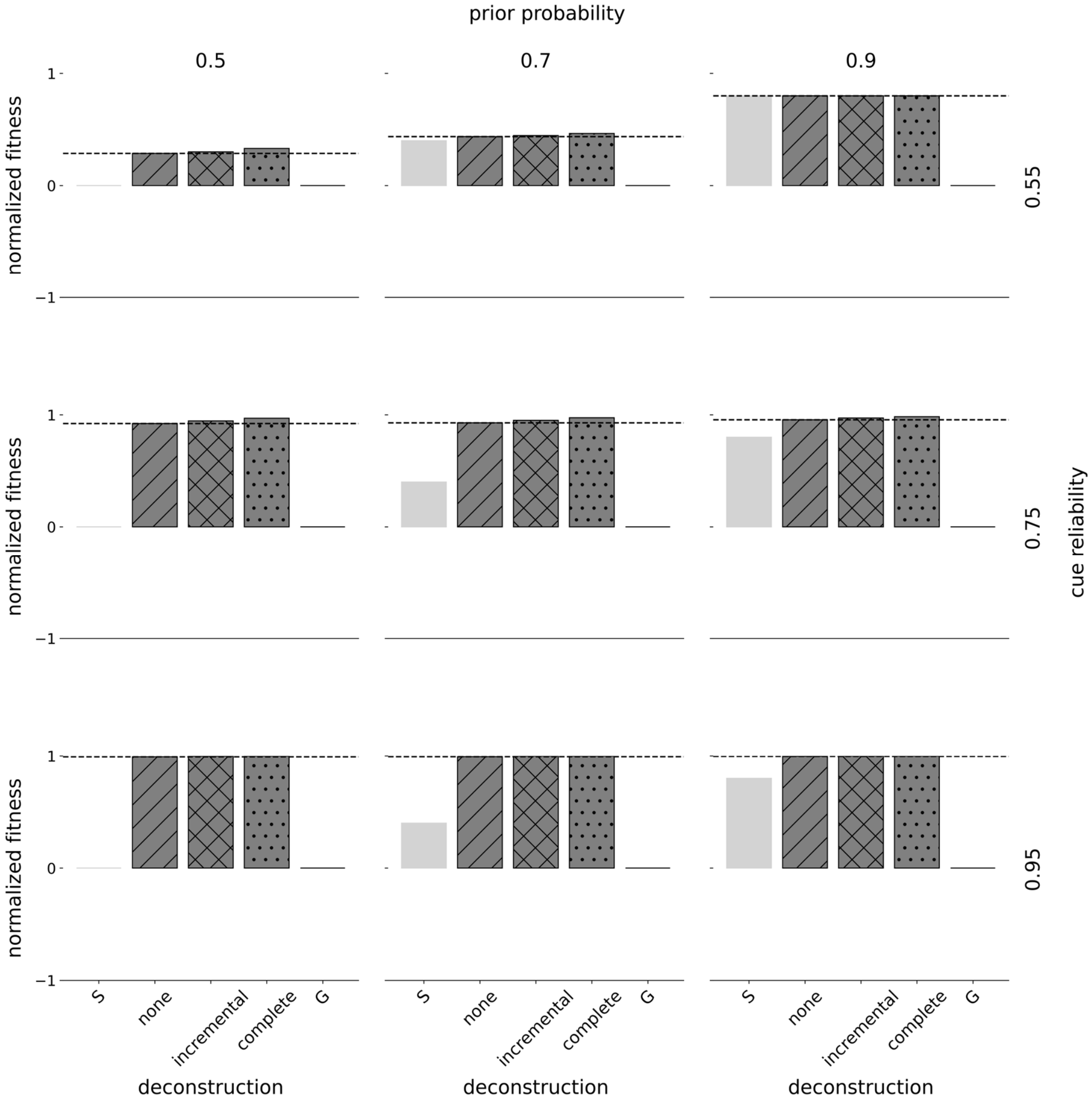

Figure S2.63: Normalized fitness. Normalized fitness is shown for diminishing rewards and diminishing penalties. Columns indicate the prior estimate of being in E_1 and rows indicate the cue reliability. Within each panel, the horizontal axis denotes the type of strategy where 'S' corresponds to a pure specialist strategy, 'O' to an optimal policy, and 'G' to a pure generalist strategy. The horizontal axis denotes fitness differences from baseline (corresponding to 0), normalized to range between -1 and 1. We show fitness of three different optimal policies: without deconstruction ('none'), with incremental deconstruction ('incremental'), and complete deconstruction ('complete'). Specialists always fully specialize according to the prior distribution. When priors are uninformative (0.5), half the population fully specializes towards P_0 and the other one towards P_1. Generalists always specialize halfway towards either phenotypic target.

Plasticity across ontogeny (incremental and complete deconstruction combined)

Linear rewards and linear penalties

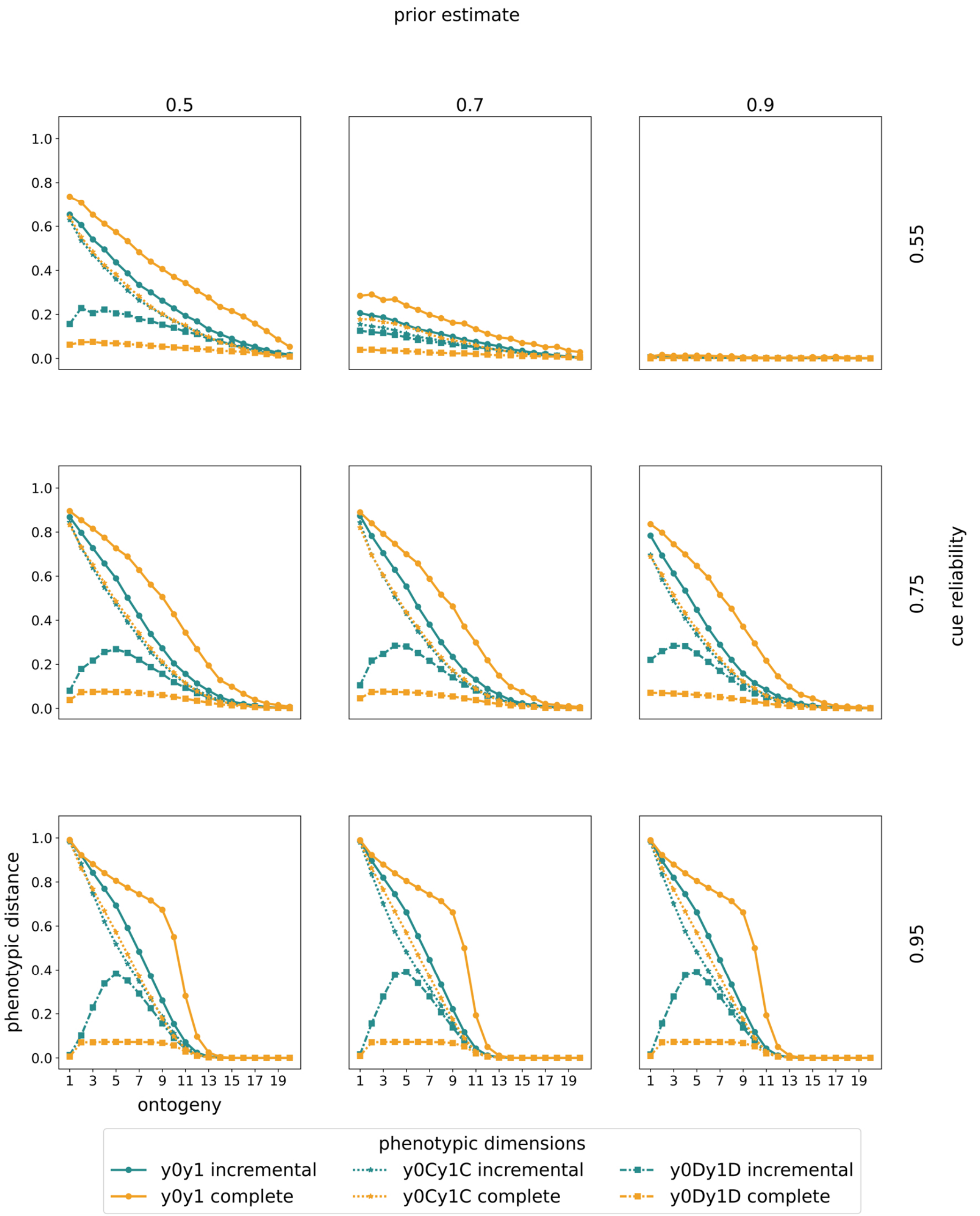

Figure S2.64: Changes in plasticity. Changes in plasticity are shown for linear rewards and linear penalties. Columns indicate the prior estimate of being in E_1 and rows indicate the cue reliability. Within each panel, the horizontal axis denotes ontogeny. The vertical axis denotes the normalized average phenotypic distance (our plasticity measure) across 10.000 pairs of simulated clones measured at the end of ontogeny. A specific point on any of the curves corresponds to a simulation experiment in which clones have been separated at the time point indicated by the horizontal axis. We show results from a model with incremental deconstruction in teal and with complete deconstruction in yellow. For each mode of deconstruction, we

present three types of plasticity curves. First, we show plasticity in construction (dotted lines and stars) as the Euclidean distance between the number of time points spent constructing. Second, we show plasticity in deconstruction (dash-dotted lines and squares) as the Euclidean distance between the number of time points spent deconstruction. Third, we show total plasticity (solid lines and circles) as the Euclidean distance between the number of specialization steps towards either target (after accounting for deconstruction). We normalize phenotypic distance in construction and total phenotypes by dividing by the maximally possible Euclidean distance, corresponding to $2 * \sqrt{T = 20}$. The normalization constant for deconstruction is $2 * \sqrt{T/2 = 10}$.

Linear rewards and increasing penalties

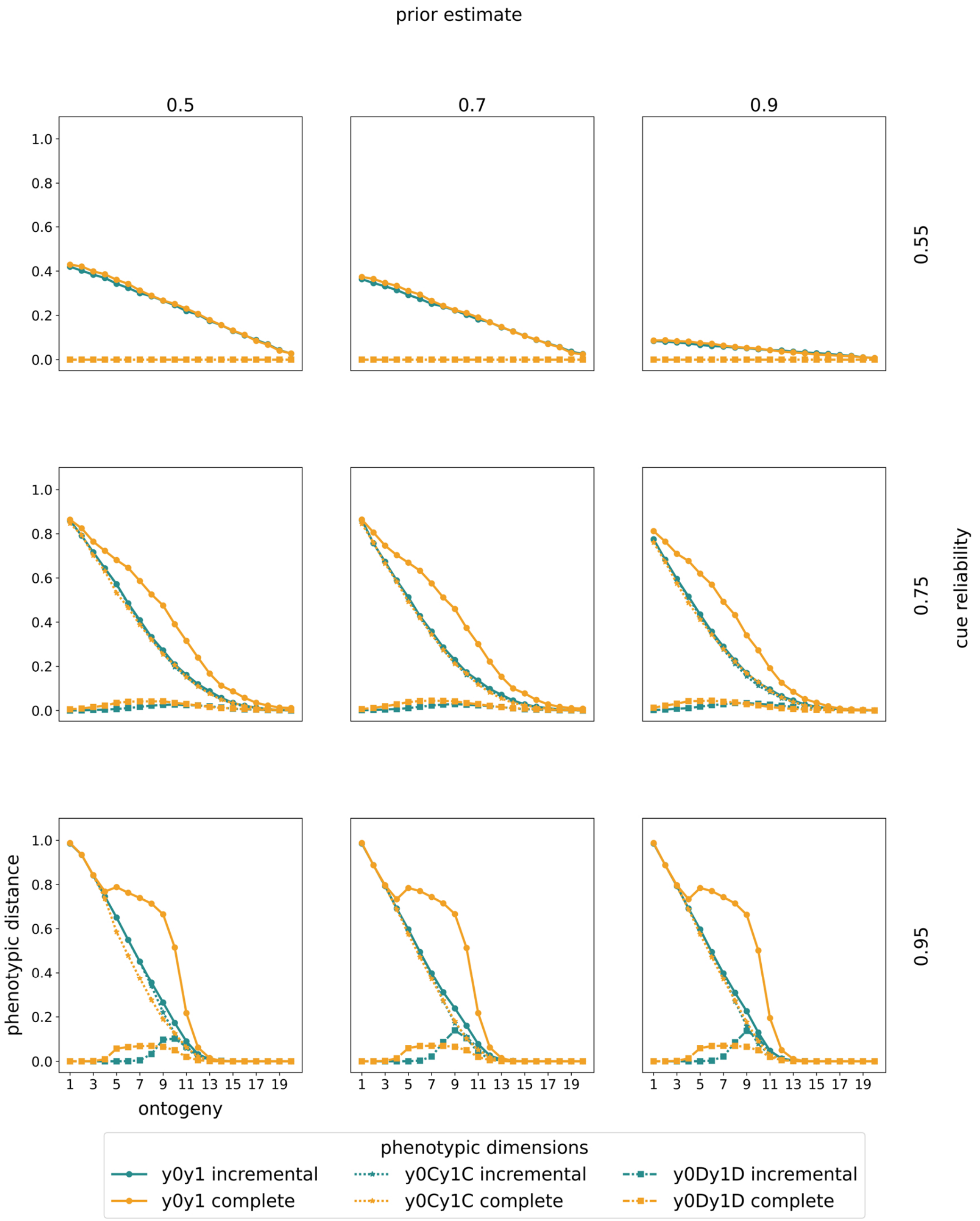

Figure S2.65: Changes in plasticity. Changes in plasticity are shown for linear rewards and increasing penalties. Columns indicate the prior estimate of being in E_1 and rows indicate the cue reliability. Within each panel, the horizontal axis denotes ontogeny. The vertical axis denotes the normalized average phenotypic distance (our plasticity measure) across 10.000 pairs of simulated clones measured at the end of ontogeny. A specific point on any of the curves corresponds to a simulation experiment in which clones have been separated at the time point indicated by the horizontal axis. We show results from a model with incremental deconstruction in teal and with complete deconstruction in yellow. For each mode of deconstruction, we present three types of plasticity curves. First, we show plasticity in construction (dotted lines and stars) as the Euclidean distance between the number of time points spent constructing. Second,

we show plasticity in deconstruction (dash-dotted lines and squares) as the Euclidean distance between the number of time points spent deconstruction. Third, we show total plasticity (solid lines and circles) as the Euclidean distance between the number of specialization steps towards either target (after accounting for deconstruction). We normalize phenotypic distance in construction and total phenotypes by dividing by the maximally possible Euclidean distance, corresponding to $2 * \sqrt{T = 20}$. The normalization constant for deconstruction is $2 * \sqrt{T/2 = 10}$.

Linear rewards and diminishing penalties

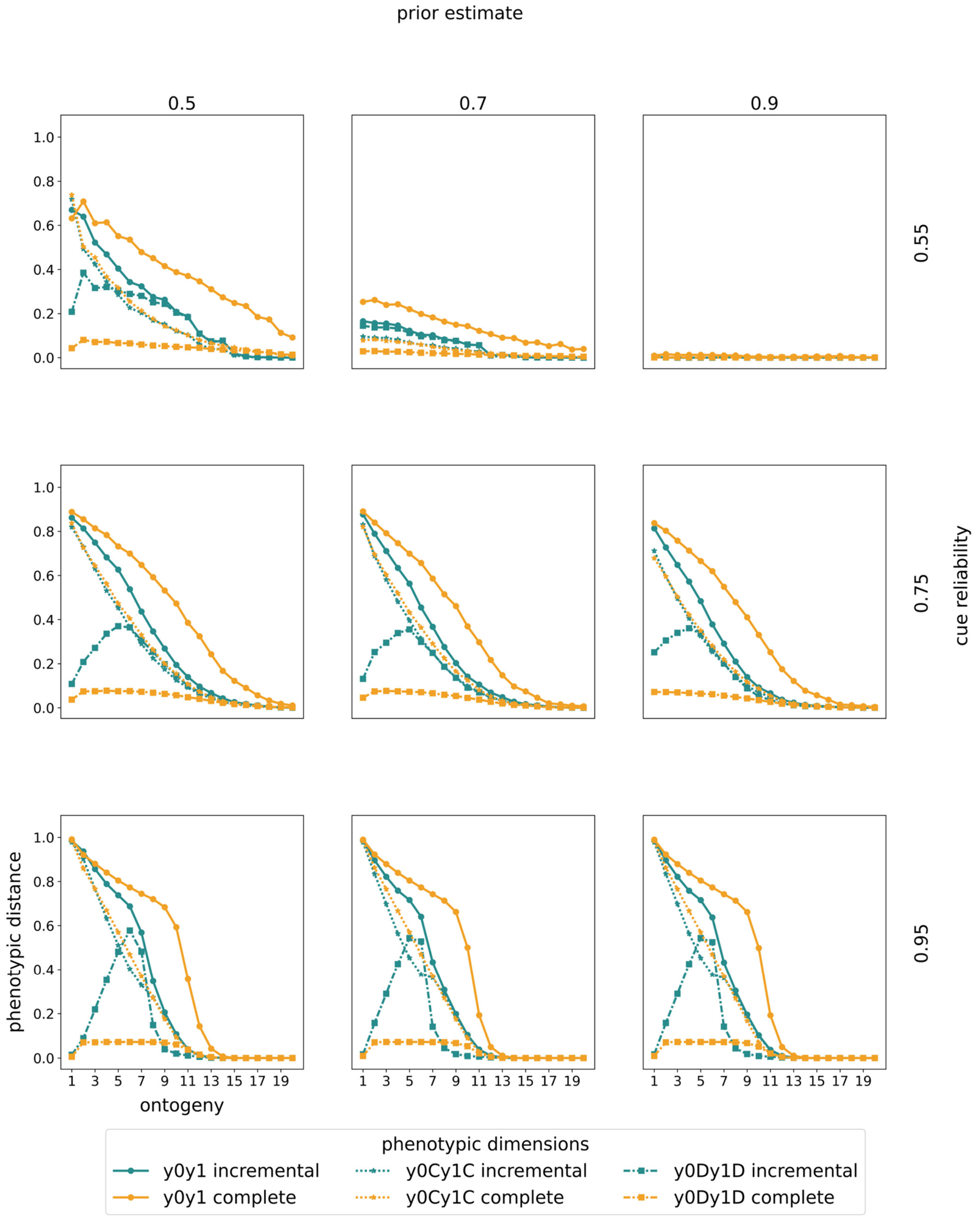

Figure S2.66: Changes in plasticity. Changes in plasticity are shown for linear rewards and diminishing penalties. Columns indicate the prior estimate of being in E_1 and rows indicate the cue reliability. Within each panel, the horizontal axis denotes ontogeny. The vertical axis denotes the normalized average phenotypic distance (our plasticity measure) across 10.000 pairs of simulated clones measured at the end of ontogeny. A specific point on any of the curves corresponds to a simulation experiment in which clones have been separated at the time point indicated by the horizontal axis. We show results from a model with incremental deconstruction in teal and with complete deconstruction in yellow. For each mode of deconstruction, we present three types of plasticity curves. First, we show plasticity in construction (dotted lines and stars) as the Euclidean distance between the number of time points spent constructing. Second, we show plasticity in deconstruction (dash-dotted lines and squares) as the Euclidean distance between the number of time points spent deconstruction. Third, we show total plasticity (solid lines and circles) as the Euclidean distance between the number of specialization steps towards either target (after accounting for deconstruction). We normalize phenotypic distance in construction and total phenotypes by dividing by the maximally possible Euclidean distance, corresponding to $2 * \sqrt{T = 20}$. The normalization constant for deconstruction is $2 * \sqrt{T/2 = 10}$.

Increasing rewards and linear penalties

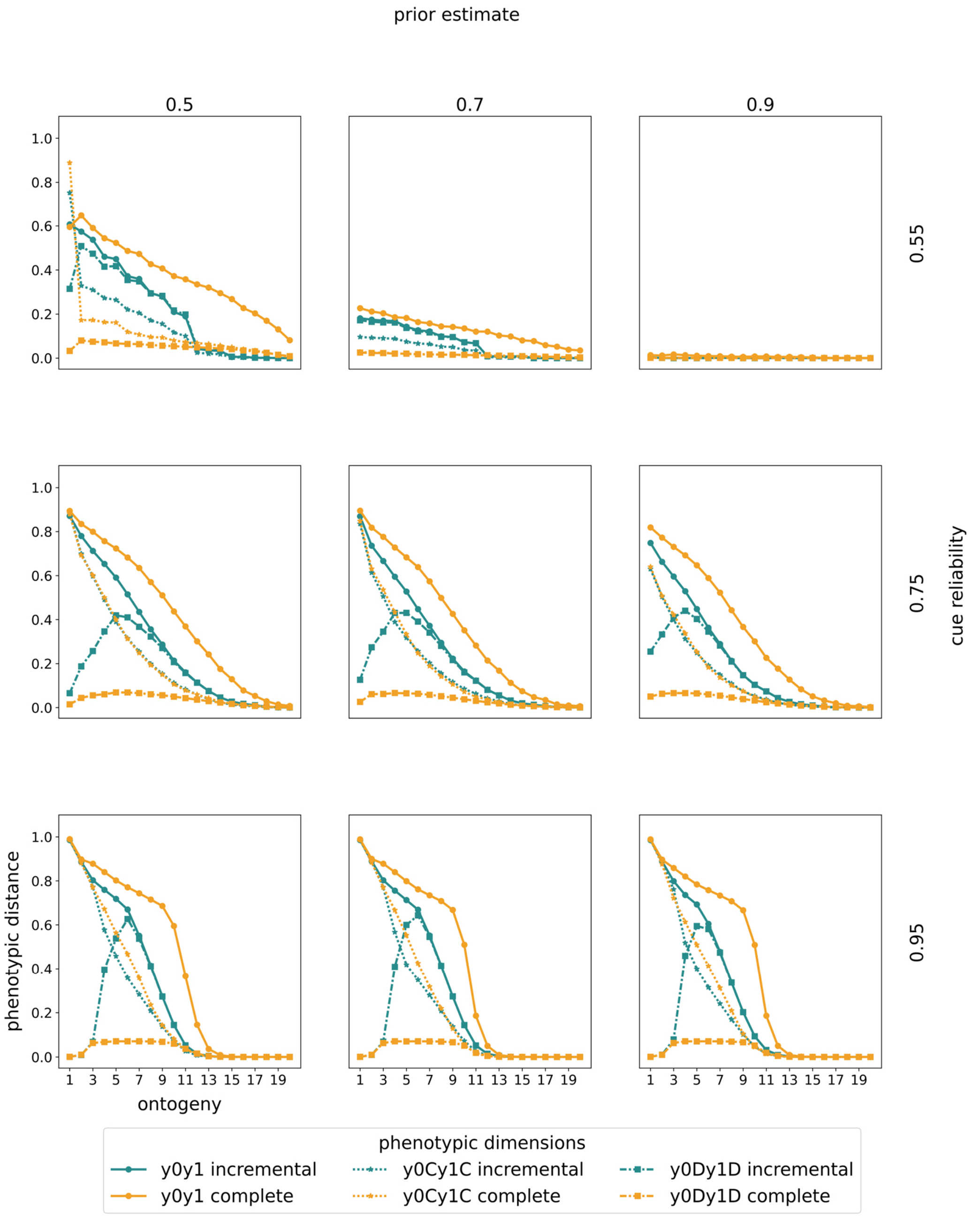

Figure S2.67: Changes in plasticity. Changes in plasticity are shown for increasing rewards and linear penalties. Columns indicate the prior estimate of being in E_1 and rows indicate the cue reliability. Within each panel, the horizontal axis denotes ontogeny. The vertical axis denotes the normalized average phenotypic distance (our plasticity measure) across 10.000 pairs of simulated clones measured at the end of ontogeny. A specific point on any of the curves corresponds to a simulation experiment in which clones have been separated at the time point indicated by the horizontal axis. We show results from a model with incremental deconstruction in teal and with complete deconstruction in yellow. For each mode of deconstruction, we present three types of plasticity curves. First, we show plasticity in construction (dotted lines and stars) as the Euclidean distance between the number of time points spent constructing. Second,

we show plasticity in deconstruction (dash-dotted lines and squares) as the Euclidean distance between the number of time points spent deconstruction. Third, we show total plasticity (solid lines and circles) as the Euclidean distance between the number of specialization steps towards either target (after accounting for deconstruction). We normalize phenotypic distance in construction and total phenotypes by dividing by the maximally possible Euclidean distance, corresponding to $2 * \sqrt{T = 20}$. The normalization constant for deconstruction is $2 * \sqrt{T/2 = 10}$.

Increasing rewards and increasing penalties

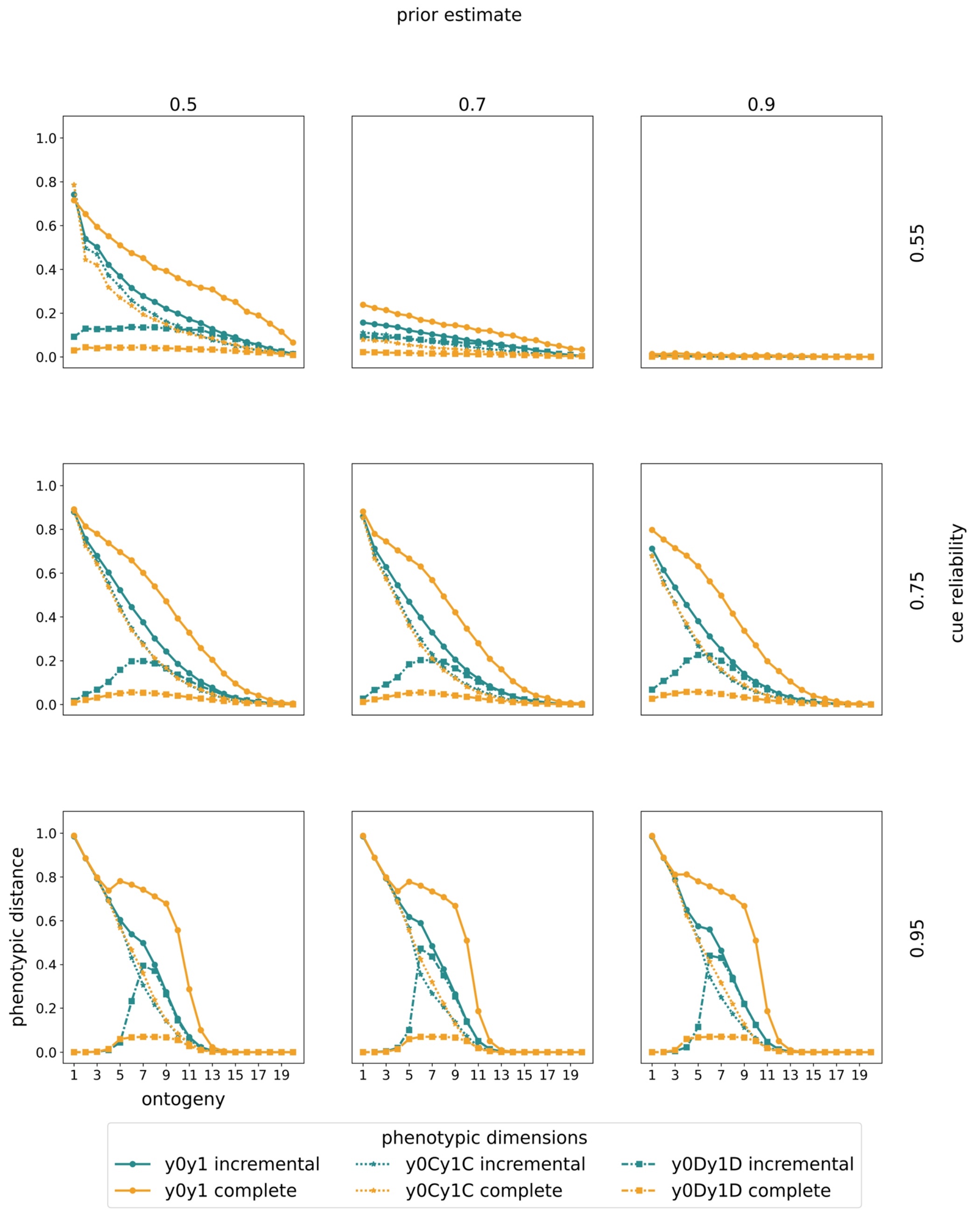

Figure S2.68: Changes in plasticity. Changes in plasticity are shown for increasing rewards and increasing penalties. Columns indicate the prior estimate of being in E_1 and rows indicate the cue reliability. Within each panel, the horizontal axis denotes ontogeny. The vertical axis denotes the normalized average phenotypic distance (our plasticity measure) across 10.000 pairs of simulated clones measured at the end of ontogeny. A specific point on any of the curves corresponds to a simulation experiment in which clones have been separated at the time point indicated by the horizontal axis. We show results from a model with incremental deconstruction in teal and with complete deconstruction in yellow. For each mode of deconstruction, we present three types of plasticity curves. First, we show plasticity in construction (dotted lines and stars) as the Euclidean distance between the number of time points spent constructing. Second, we show plasticity in deconstruction (dash-dotted lines and squares) as the Euclidean distance between the number of time points spent deconstruction. Third, we show total plasticity (solid lines and circles) as the Euclidean distance between the number of specialization steps towards either target (after accounting for deconstruction). We normalize phenotypic distance in construction and total phenotypes by dividing by the maximally possible Euclidean distance, corresponding to $2 * \sqrt{T = 20}$. The normalization constant for deconstruction is $2 * \sqrt{T/2 = 10}$.

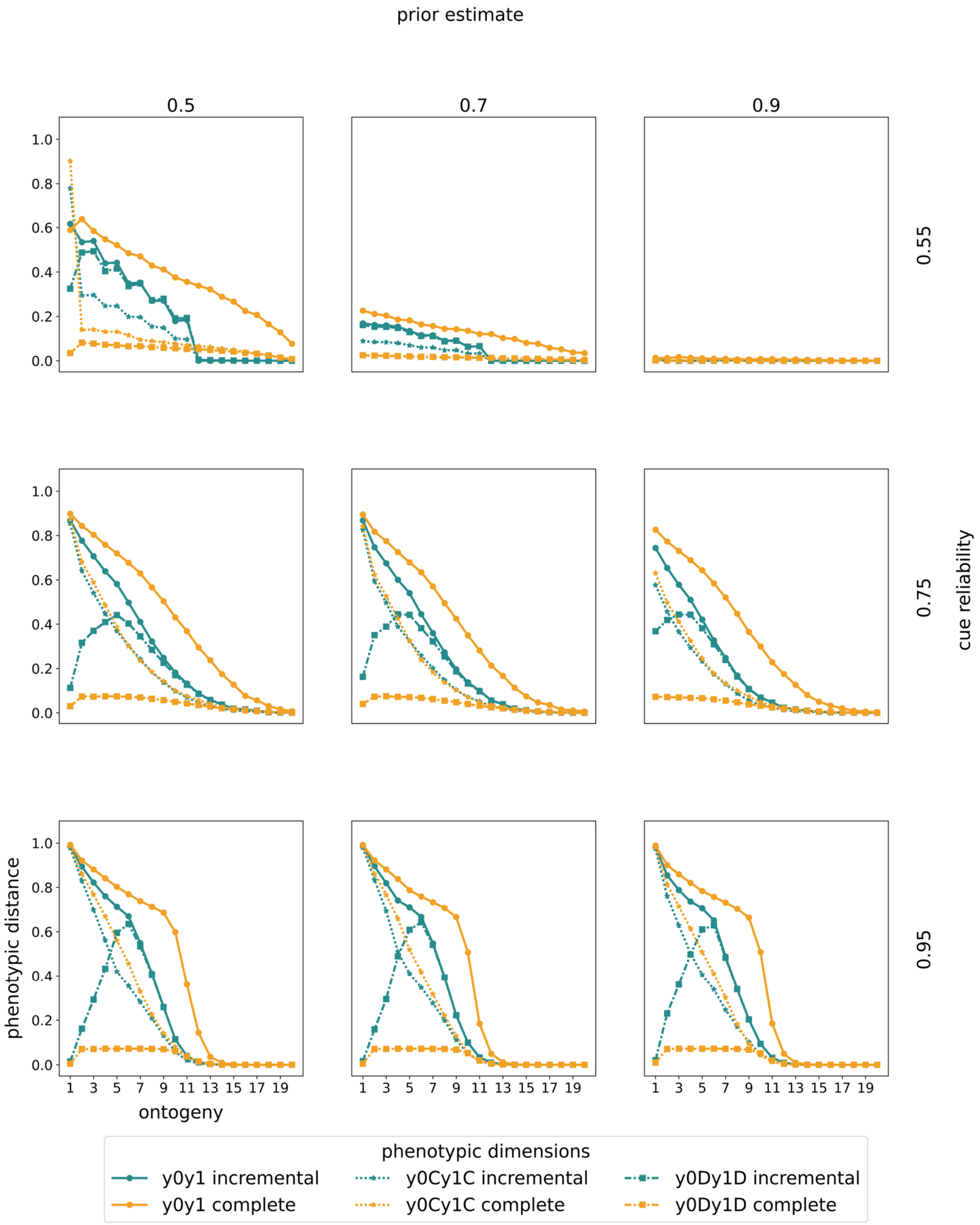

Figure S2.69: Changes in plasticity. Changes in plasticity are shown for increasing rewards and diminishing penalties. Columns indicate the prior estimate of being in E_1 and rows indicate the cue reliability. Within each panel, the horizontal axis denotes ontogeny. The vertical axis denotes the normalized average phenotypic distance (our plasticity measure) across 10.000 pairs of simulated clones measured at the end of ontogeny. A specific point on any of the curves corresponds to a simulation experiment in which clones have been separated at the time point indicated by the horizontal axis. We show results from a model with incremental deconstruction in teal and with complete deconstruction in yellow. For each mode of deconstruction, we present three types of plasticity curves. First, we show plasticity in construction (dotted lines and stars) as the Euclidean distance between the number of time points spent constructing. Second,

we show plasticity in deconstruction (dash-dotted lines and squares) as the Euclidean distance between the number of time points spent deconstruction. Third, we show total plasticity (solid lines and circles) as the Euclidean distance between the number of specialization steps towards either target (after accounting for deconstruction). We normalize phenotypic distance in construction and total phenotypes by dividing by the maximally possible Euclidean distance, corresponding to $2 * \sqrt{T = 20}$. The normalization constant for deconstruction is $2 * \sqrt{T/2 = 10}$.

Diminishing rewards and linear penalties

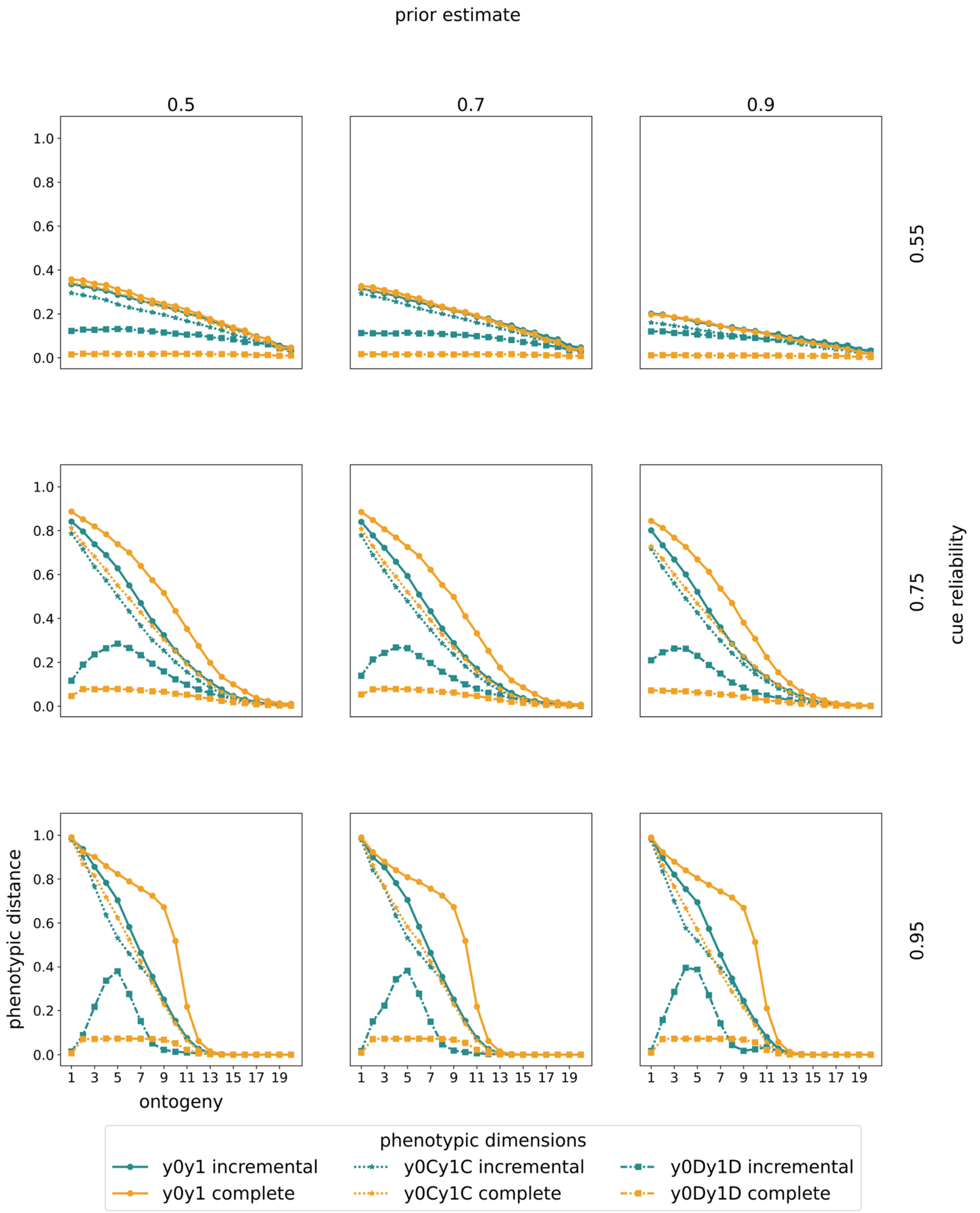

Figure S2.70: Changes in plasticity. Changes in plasticity are shown for diminishing rewards and linear penalties. Columns indicate the prior estimate of being in E_1 and rows indicate the cue reliability. Within each panel, the horizontal axis denotes ontogeny. The vertical axis denotes the normalized average phenotypic distance (our plasticity measure) across 10.000 pairs of simulated clones measured at the end of ontogeny. A specific point on any of the curves corresponds to a simulation experiment in which clones have been separated at the time point indicated by the horizontal axis. We show results from a model with incremental deconstruction in teal and with complete deconstruction in yellow. For each mode of deconstruction, we present three types of plasticity curves. First, we show plasticity in construction (dotted lines and stars) as the Euclidean distance between the number of time points spent constructing. Second, we show plasticity in deconstruction (dash-dotted lines and squares) as the Euclidean distance between the number of time points spent deconstruction. Third, we show total plasticity (solid lines and circles) as the Euclidean distance between the number of specialization steps towards either target (after accounting for deconstruction). We normalize phenotypic distance in construction and total phenotypes by dividing by the maximally possible Euclidean distance, corresponding to $2 * \sqrt{T = 20}$. The normalization constant for deconstruction is $2 * \sqrt{T/2 = 10}$.

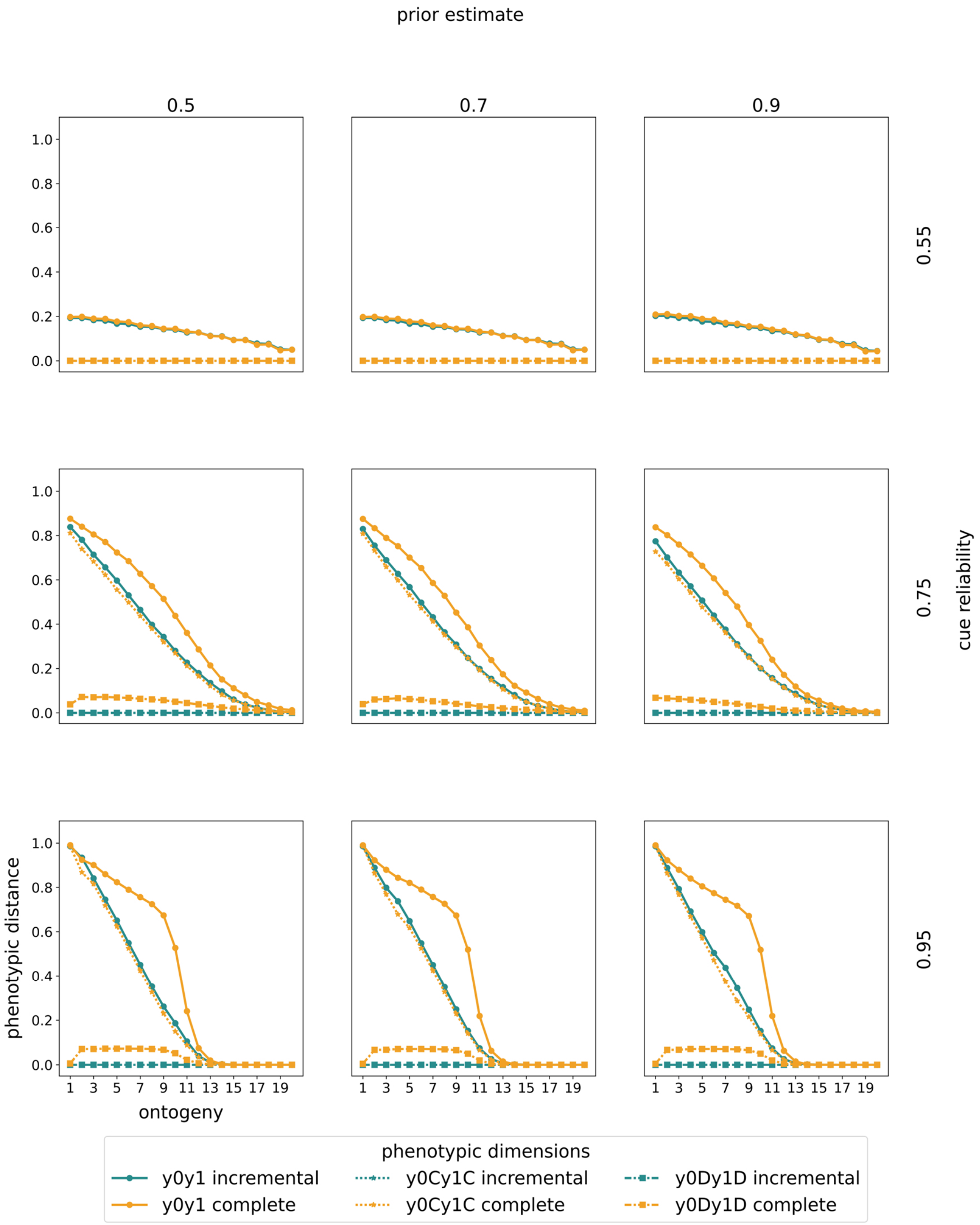

Figure S2.71: Changes in plasticity. Changes in plasticity are shown for diminishing rewards and increasing penalties. Columns indicate the prior estimate of being in E_1 and rows indicate the cue reliability. Within each panel, the horizontal axis denotes ontogeny. The vertical axis denotes the normalized average phenotypic distance (our plasticity measure) across 10.000 pairs of simulated clones measured at the end of ontogeny. A specific point on any of the curves corresponds to a simulation experiment in which clones have been separated at the time point indicated by the horizontal axis. We show results from a model with incremental deconstruction in teal and with complete deconstruction in yellow. For each mode of deconstruction, we present three types of plasticity curves. First, we show plasticity in construction (dotted lines and stars) as the Euclidean distance between the number of time points spent constructing. Second,

we show plasticity in deconstruction (dash-dotted lines and squares) as the Euclidean distance between the number of time points spent deconstruction. Third, we show total plasticity (solid lines and circles) as the Euclidean distance between the number of specialization steps towards either target (after accounting for deconstruction). We normalize phenotypic distance in construction and total phenotypes by dividing by the maximally possible Euclidean distance, corresponding to $2 * \sqrt{T = 20}$. The normalization constant for deconstruction is $2 * \sqrt{T/2 = 10}$.

Diminishing rewards and diminishing penalties

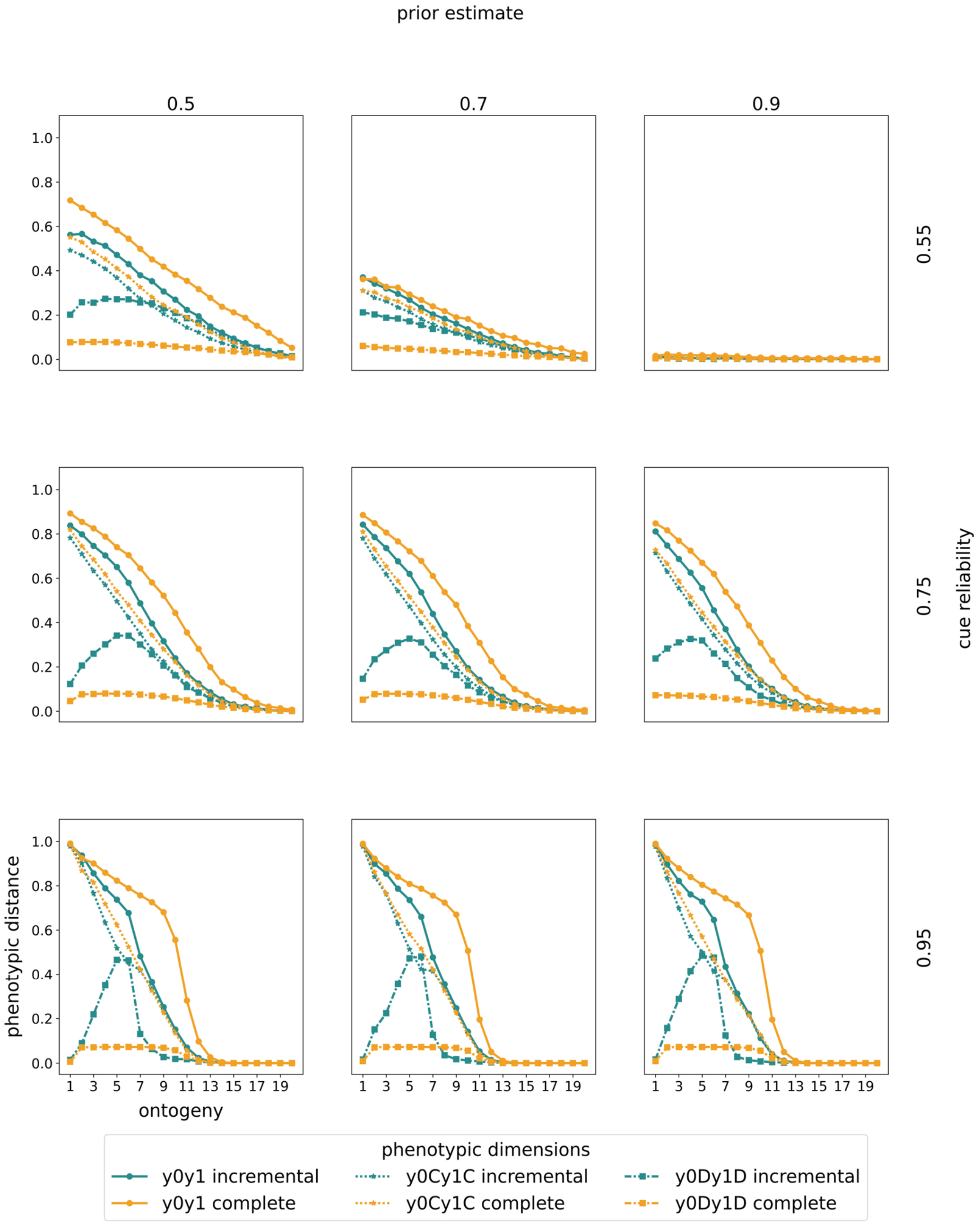

Figure S2.72: Changes in plasticity. Changes in plasticity are shown for diminishing rewards and diminishing penalties. Columns indicate the prior estimate of being in E_1 and rows indicate the cue reliability. Within each panel, the horizontal axis denotes ontogeny. The vertical axis denotes the normalized average phenotypic distance (our plasticity measure) across 10.000 pairs of simulated clones measured at the end of ontogeny. A specific point on any of the curves corresponds to a simulation experiment in which clones have been separated at the time point indicated by the horizontal axis. We show results from a model with incremental deconstruction in teal and with complete deconstruction in yellow. For each mode of deconstruction, we present three types of plasticity curves. First, we show plasticity in construction (dotted lines and stars) as the Euclidean distance between the number of time points spent constructing. Second, we show plasticity in deconstruction (dash-dotted lines and squares) as the Euclidean distance between the number of time points spent deconstruction. Third, we show total plasticity (solid lines and circles) as the Euclidean distance between the number of specialization steps towards either target (after accounting for deconstruction). We normalize phenotypic distance in construction and total phenotypes by dividing by the maximally possible Euclidean distance, corresponding to $2 * \sqrt{T = 20}$. The normalization constant for deconstruction is $2 * \sqrt{T/2 = 10}$.

ESM 3 - Comparison of 5 and 10 time periods of on ontogeny

5 time periods of ontogeny

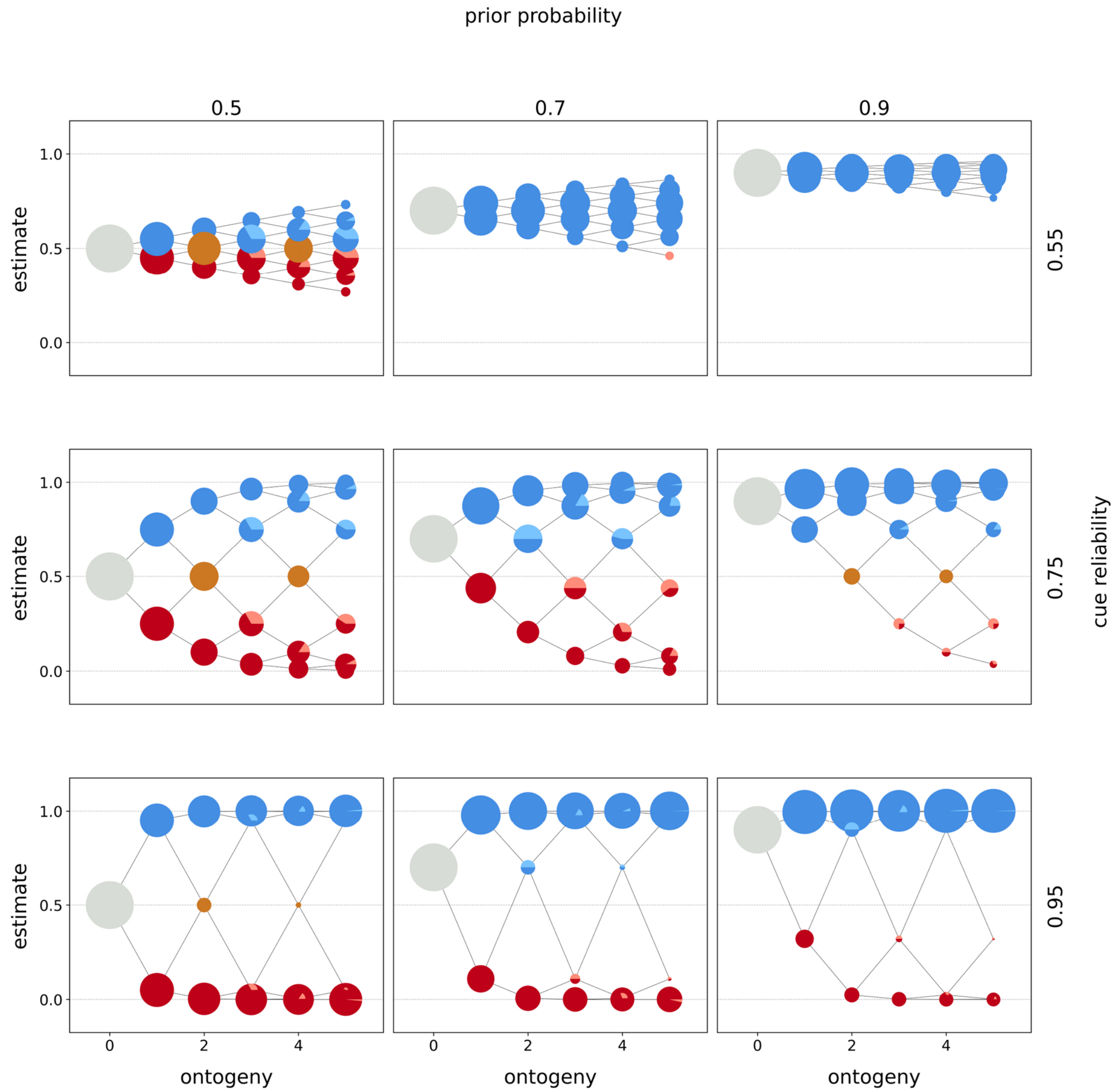

Figure S3.1: Optimal policies. Optimal policies are shown for a model with incremental deconstruction and linear rewards and penalties. Columns indicate the prior estimate of being in E_1 and rows indicate the cue reliability. Within each panel, the horizontal axis denotes ontogeny and the vertical axis the posterior estimates of being in E_1. The entire population starts ontogeny with zero cues sampled and the prior estimate indicated by the column (indicated by the grey circle). In each time period organisms sample a cue (either C_0 or C_1), update their estimate, and make a phenotypic decision (colored circles). Black lines indicate developmental trajectories through this decision space, with lines branching upwards indicating the sampling of C_1 and lines branching downwards indicating the sampling of C_o. Colors denote the optimal, fitness-maximizing phenotypic choice in each state. Pies indicate cases in which organisms with the same posterior estimates make different phenotypic decisions. The area of a circle (pie piece) is proportional the probability of reaching that particular state. Colors indicate the following phenotypic decisions: Black corresponds to waiting, red to constructing P_0, blue to constructing P_1, purple to deconstructing P_0, green to deconstructing P_1, light red to a tie between constructing P_0 and deconstructing P_1, light blue to a tie between constructing P_1 and deconstructing P_0, brown to a tie between constructing either phenotypic

target, yellow to a tie between deconstructing either target, grey to a tie between construction and waiting, dark grey to a tie between deconstruction and waiting, and lastly ochre to a tie between all options.

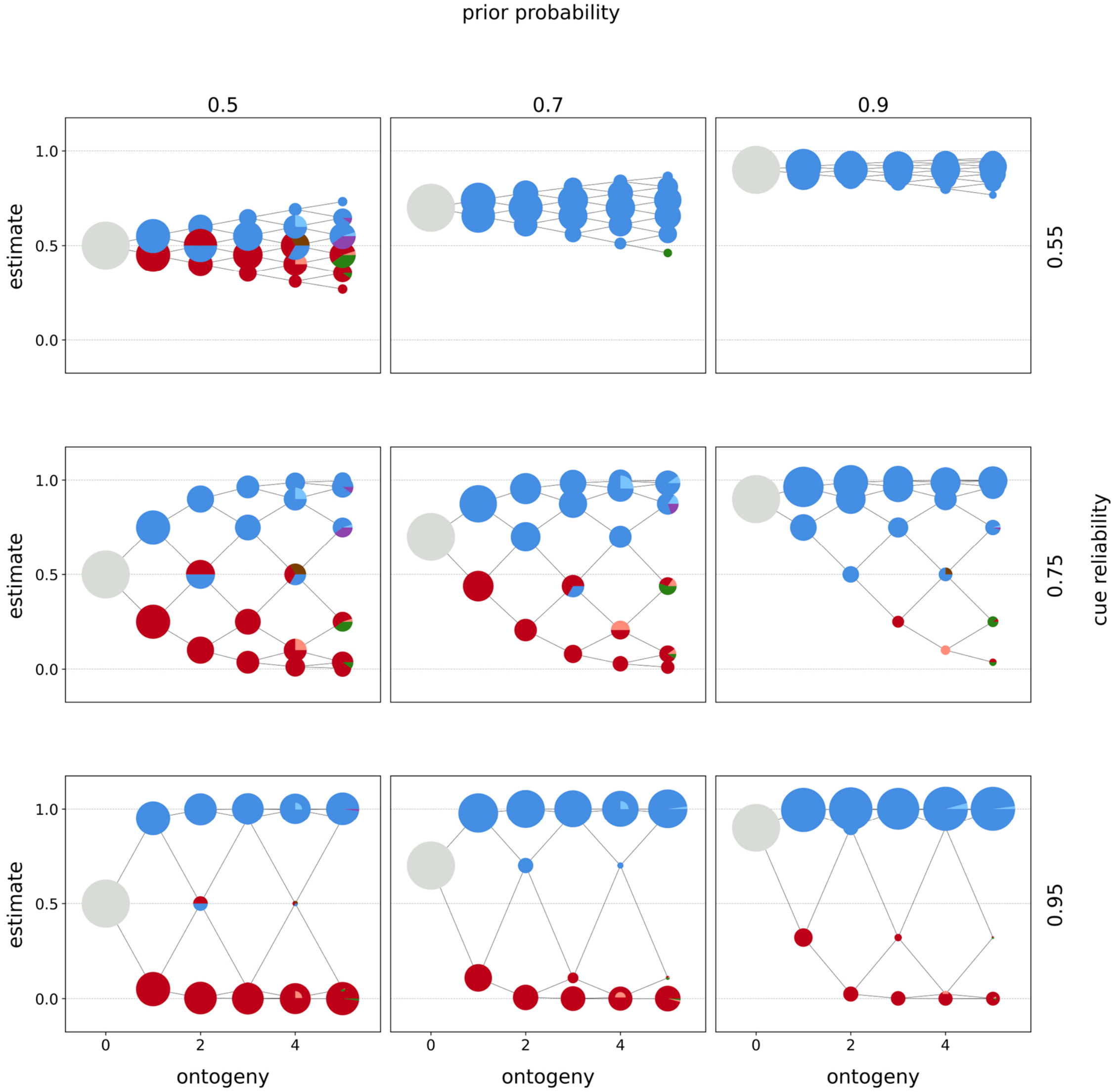

Figure S3.2: Optimal policies. Optimal policies are shown for a model with complete deconstruction and linear rewards and penalties. Columns indicate the prior estimate of being in E_1 and rows indicate the cue reliability. Within each panel, the horizontal axis denotes ontogeny and the vertical axis the posterior estimates of being in E_1. The entire population starts ontogeny with zero cues sampled and the prior estimate indicated by the column (indicated by the grey circle). In each time period organisms sample a cue (either C_0 or C_1), update their estimate, and make a phenotypic decision (colored circles). Black lines indicate developmental trajectories through this decision space, with lines branching upwards indicating the sampling of C_1 and lines branching downwards indicating the sampling of C_o. Colors denote the optimal, fitness-maximizing phenotypic choice in each state. Pies indicate cases in which organisms with the same posterior estimates make different phenotypic decisions. The area of a circle (pie piece) is proportional the probability of reaching that particular state. Colors indicate the following phenotypic decisions: Black corresponds to waiting, red to constructing P_0, blue to constructing P_1, purple to deconstructing P_0, green to deconstructing P_1, light red to a tie between constructing P_0 and deconstructing P_1, light blue to a tie between constructing P_1 and deconstructing P_0, brown to a tie between constructing either phenotypic

target, yellow to a tie between deconstructing either target, grey to a tie between construction and waiting, dark grey to a tie between deconstruction and waiting, and lastly ochre to a tie between all options.

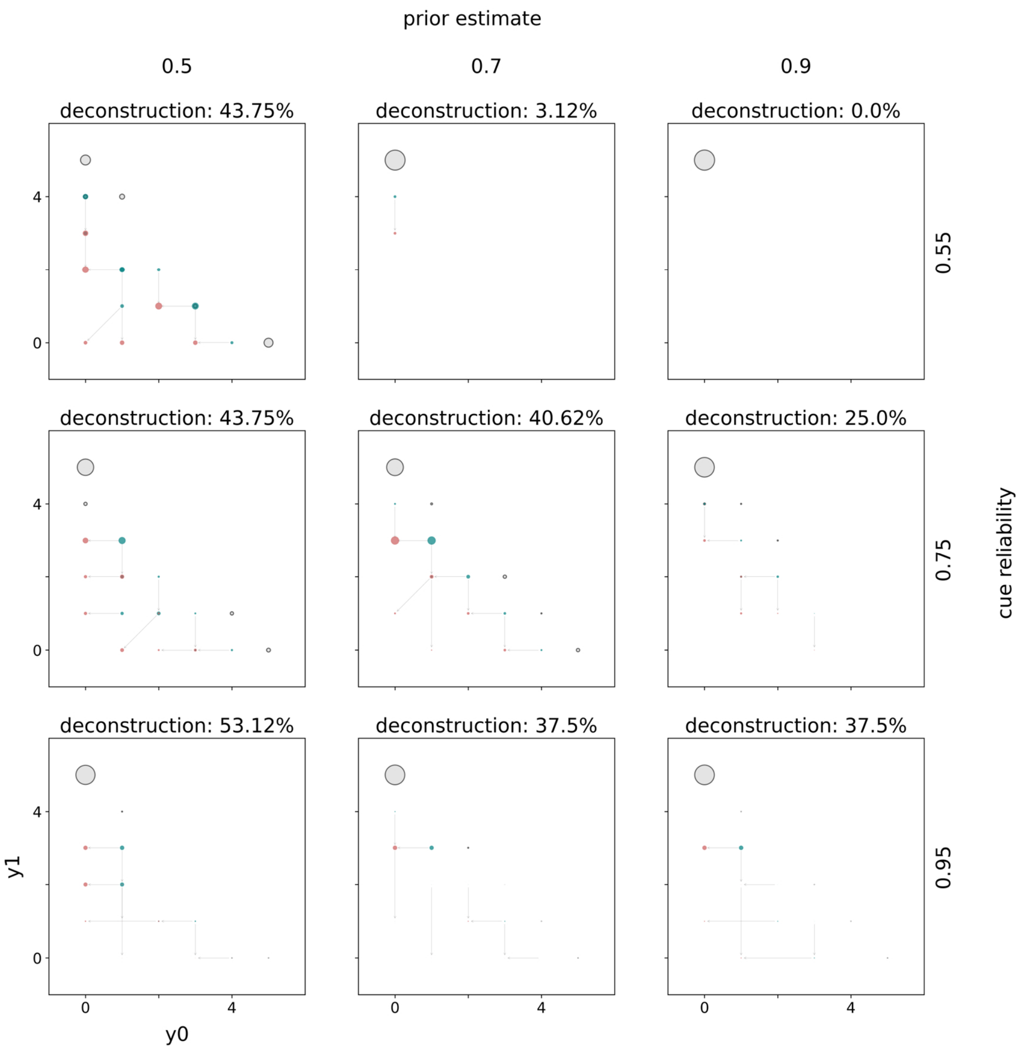

Figure S3.3: Distributions of mature phenotypes. Distributions of mature phenotypes are shown for a model with incremental deconstruction and linear rewards and penalties. Columns indicate the prior estimate of being in E_1 and rows indicate the cue reliability. The populations of mature phenotypes have been simulated in E_1. The title of each panel indicates the percentage of mature phenotypes that have deconstructed at some point during ontogeny. Within each panel the horizontal axis indicates the number of specializations towards E_0 and the vertical axis towards E_1. The lower triangle indicates how much mature phenotypes have constructed (teal circles) and what their phenotype looked like after deconstruction (red circles). Grey arrows connect phenotypes before (teal) and after (red) deconstruction. Grey circles with a black outline belong to mature phenotypes that never deconstructed. The area of a circle is proportional to the number mature organisms with this phenotype. The upper triangle indicates waiting. For each mature phenotype (after deconstruction) below the diagonal the corresponding square above the diagonal highlights the amount of waiting. The color intensity is proportional to the amount of waiting. Black squares indicate phenotypes that waited all of ontogeny (i.e. 20 time periods) and white squares phenotypes that never waited.

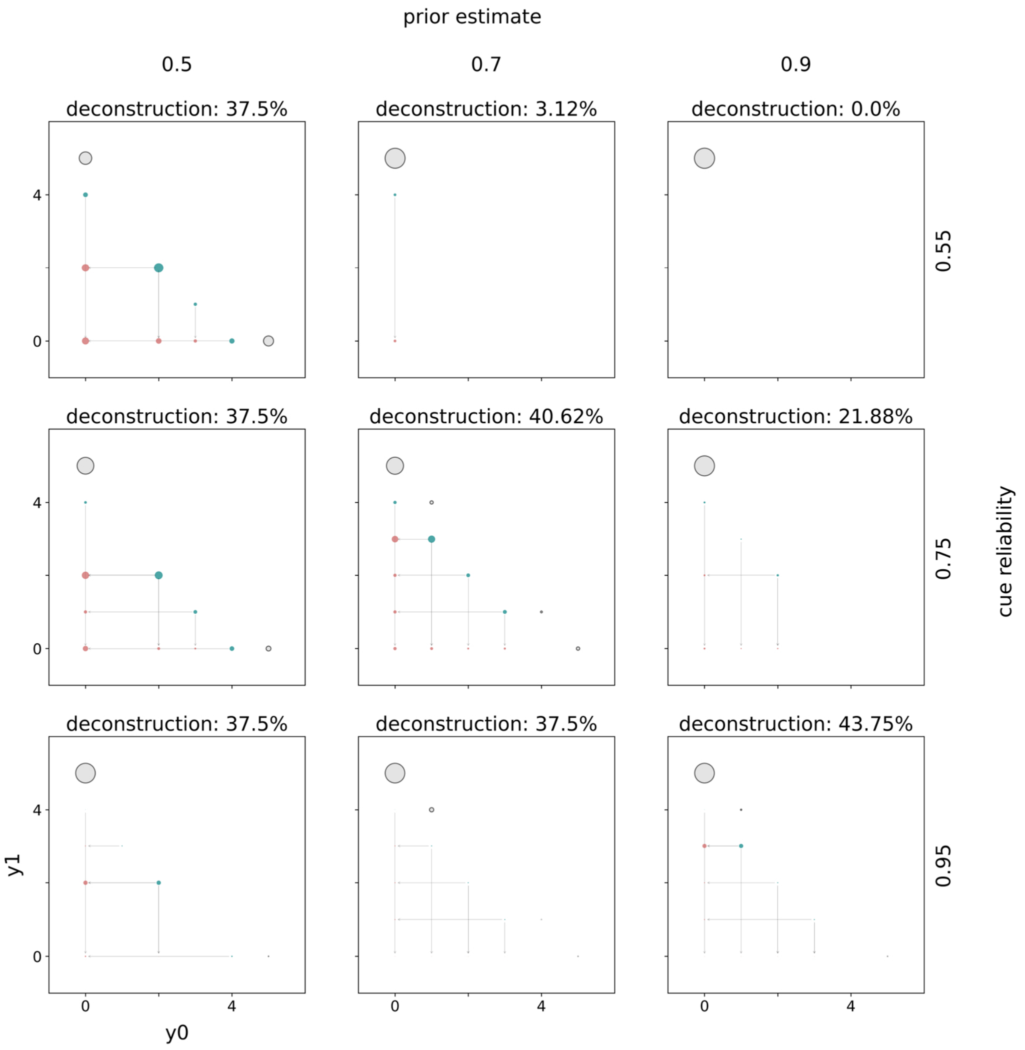

Figure S3.4: Distributions of mature phenotypes. Distributions of mature phenotypes are shown for a model with complete deconstruction and linear rewards and penalties. Columns indicate the prior estimate of being in E_1 and rows indicate the cue reliability. The populations of mature phenotypes have been simulated in E_1. The title of each panel indicates the percentage of mature phenotypes that have deconstructed at some point during ontogeny. Within each panel the horizontal axis indicates the number of specializations towards E_0 and the vertical axis towards E_1. The lower triangle indicates how much mature phenotypes have constructed (teal circles) and what their phenotype looked like after deconstruction (red circles). Grey arrows connect phenotypes before (teal) and after (red) deconstruction. Grey circles with a black outline belong to mature phenotypes that never deconstructed. The area of a circle is proportional to the number mature organisms with this phenotype. The upper triangle indicates waiting. For each mature phenotype (after deconstruction) below the diagonal the corresponding square above the diagonal highlights the amount of waiting. The color intensity is proportional to the amount of waiting. Black squares indicate phenotypes that waited all of ontogeny (i.e. 20 time periods) and white squares phenotypes that never waited.

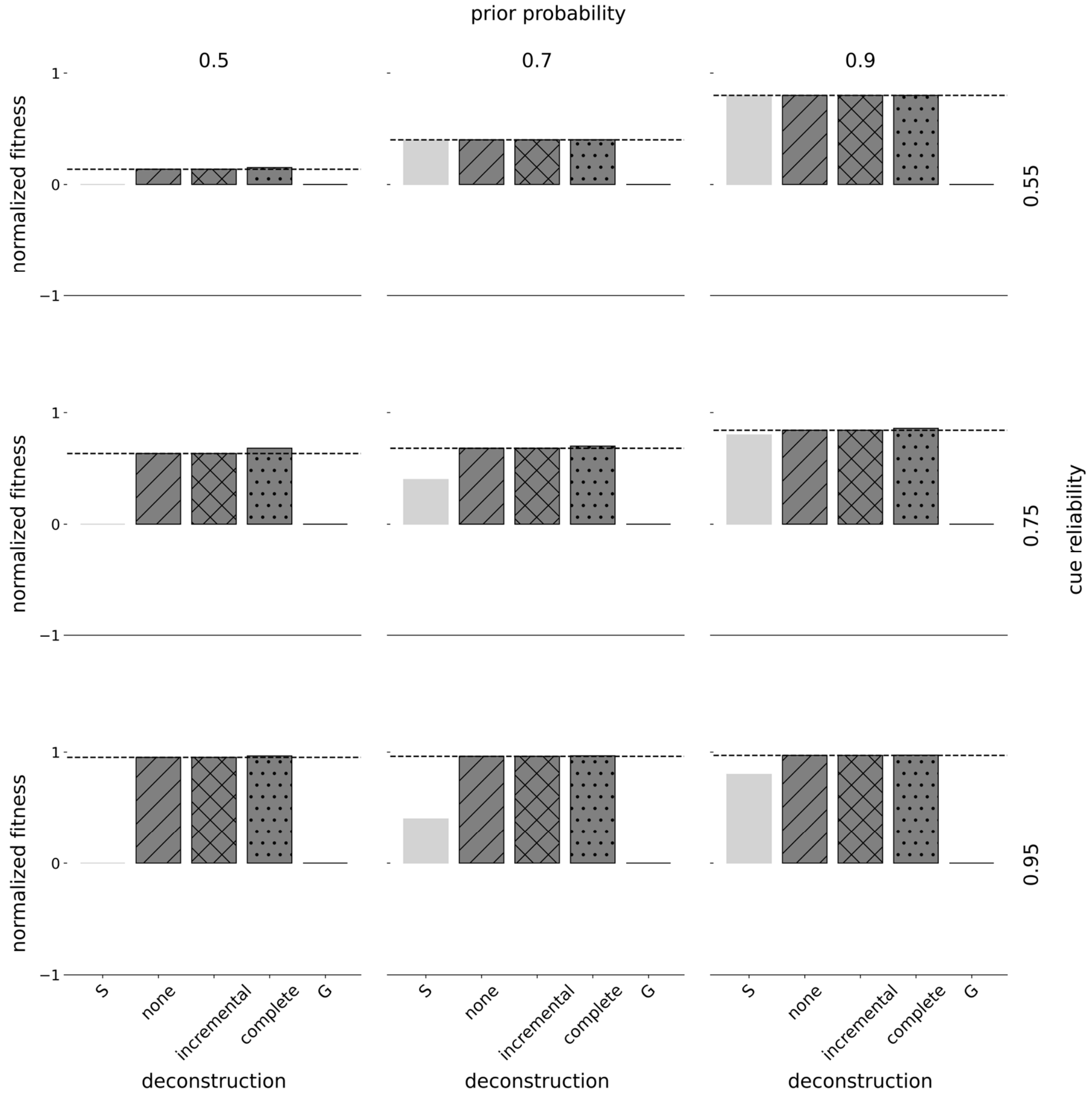

Figure S3.5: Normalized fitness. Normalized fitness is shown for linear rewards and linear penalties. Columns indicate the prior estimate of being in E_1 and rows indicate the cue reliability. Within each panel, the horizontal axis denotes the type of strategy where 'S' corresponds to a pure specialist strategy, 'O' to an optimal policy, and 'G' to a pure generalist strategy. The horizontal axis denotes fitness differences from baseline (corresponding to 0), normalized to range between -1 and 1. We show fitness of three different optimal policies: without deconstruction ('none'), with incremental deconstruction ('incremental'), and complete deconstruction ('complete'). Specialists always fully specialize according to the prior distribution. When priors are uninformative (0.5), half the population fully specializes towards P_0 and the other one towards P_1. Generalists always specialize halfway towards either phenotypic target.

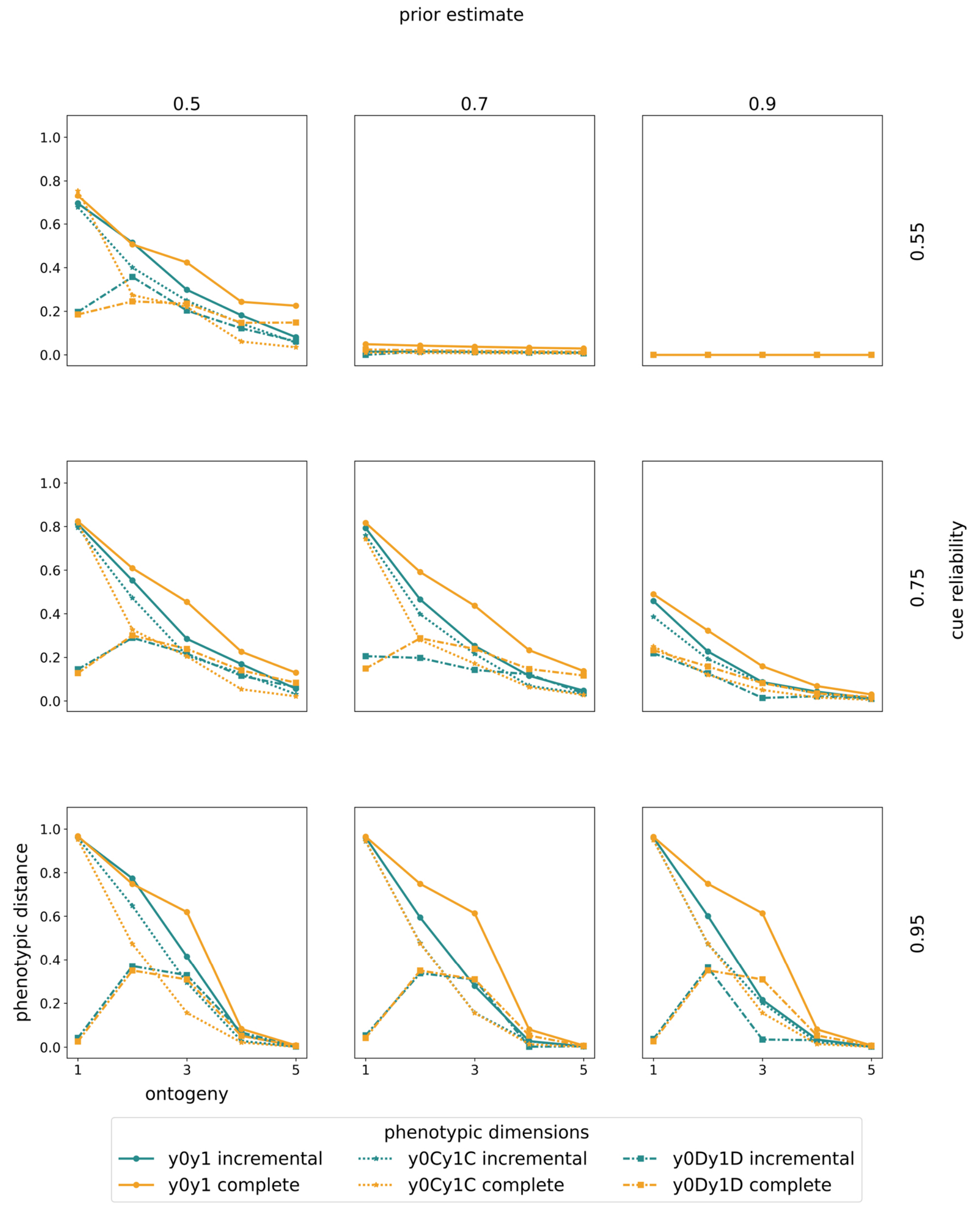

Figure S3.6: Changes in plasticity. Changes in plasticity are shown for linear rewards and linear penalties. Columns indicate the prior estimate of being in E_1 and rows indicate the cue reliability. Within each panel, the horizontal axis denotes ontogeny. The vertical axis denotes the normalized average phenotypic distance (our plasticity measure) across 10.000 pairs of simulated clones measured at the end of ontogeny. A specific point on any of the curves corresponds to a simulation experiment in which clones have been separated at the time point indicated by the horizontal axis. We show results from a model with incremental deconstruction in teal and with complete deconstruction in yellow. For each mode of deconstruction, we present three types of plasticity curves. First, we show plasticity in construction (dotted lines and stars) as the Euclidean distance between the number of time points spent constructing. Second, we show plasticity in deconstruction (dash-dotted lines and squares) as the Euclidean distance between the number of time points spent deconstruction. Third, we show total plasticity (solid lines and circles) as the Euclidean

distance between the number of specialization steps towards either target (after accounting for deconstruction). We normalize phenotypic distance in construction and total phenotypes by dividing by the maximally possible Euclidean distance, corresponding to $2 * \sqrt{T = 5}$. The normalization constant for deconstruction is $2 * \sqrt{T/2 = 2.5}$.

10 time periods of ontogeny

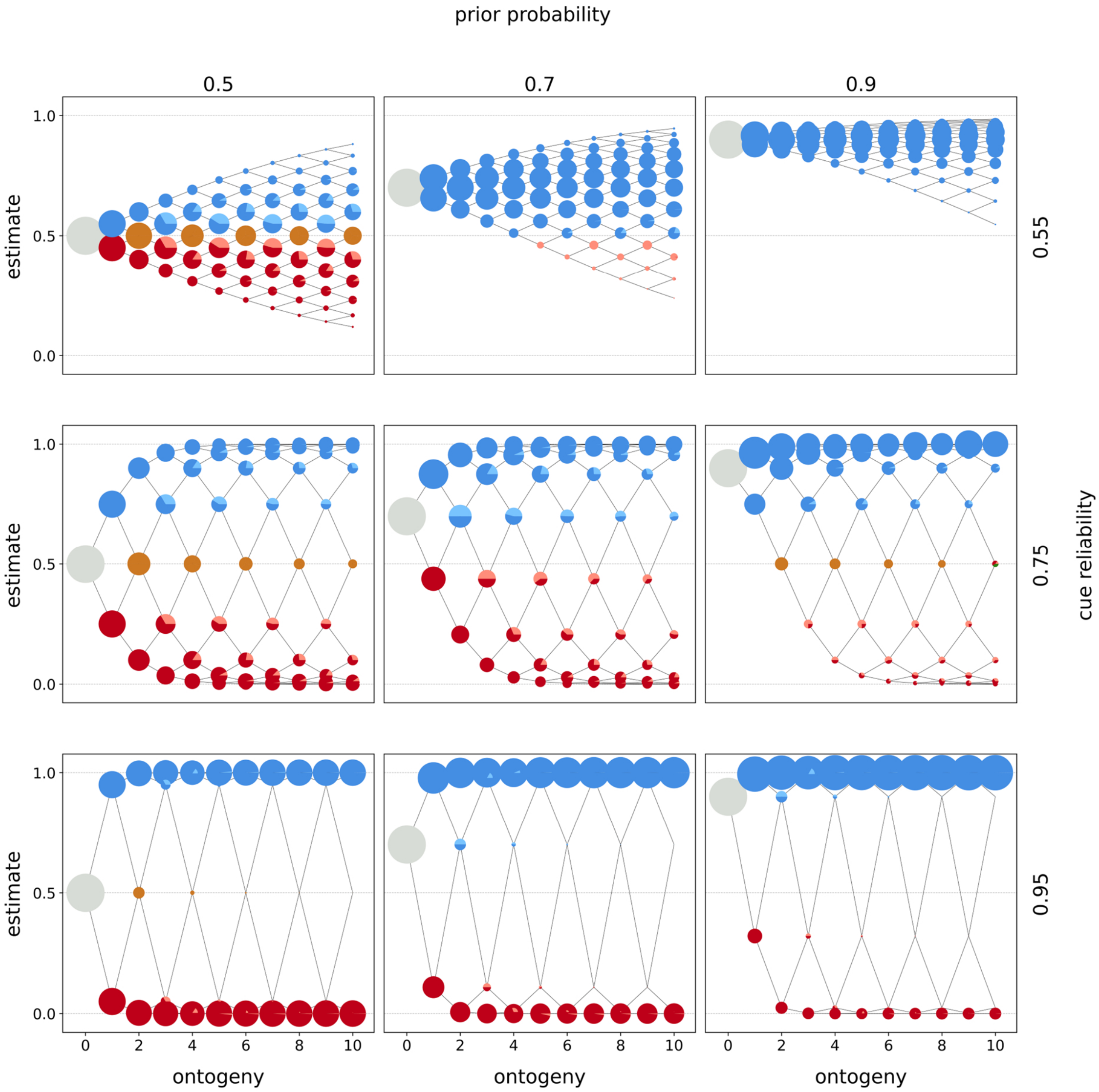

Figure S3.7: Optimal policies. Optimal policies are shown for a model with incremental deconstruction and linear rewards and penalties. Columns indicate the prior estimate of being in E_1 and rows indicate the cue reliability. Within each panel, the horizontal axis denotes ontogeny and the vertical axis the posterior estimates of being in E_1. The entire population starts ontogeny with zero cues sampled and the prior estimate indicated by the column (indicated by the grey circle). In each time period organisms sample a cue (either C_0 or C_1), update their estimate, and make a phenotypic decision (colored circles). Black lines indicate developmental trajectories through this decision space, with lines branching upwards indicating the sampling of C_1 and lines branching downwards indicating the sampling of C_o. Colors denote the optimal, fitness-maximizing phenotypic choice in each state. Pies indicate cases in which organisms with the same posterior estimates make different phenotypic decisions. The area of a circle (pie piece) is proportional the probability of reaching that particular state. Colors indicate the following phenotypic decisions: Black corresponds to waiting, red to constructing P_0, blue to constructing P_1, purple to deconstructing P_0, green to deconstructing P_1, light red to a tie between constructing P_0 and deconstructing P_1, light blue to a tie between constructing P_1 and deconstructing P_0, brown to a tie between constructing either phenotypic

target, yellow to a tie between deconstructing either target, grey to a tie between construction and waiting, dark grey to a tie between deconstruction and waiting, and lastly ochre to a tie between all options.

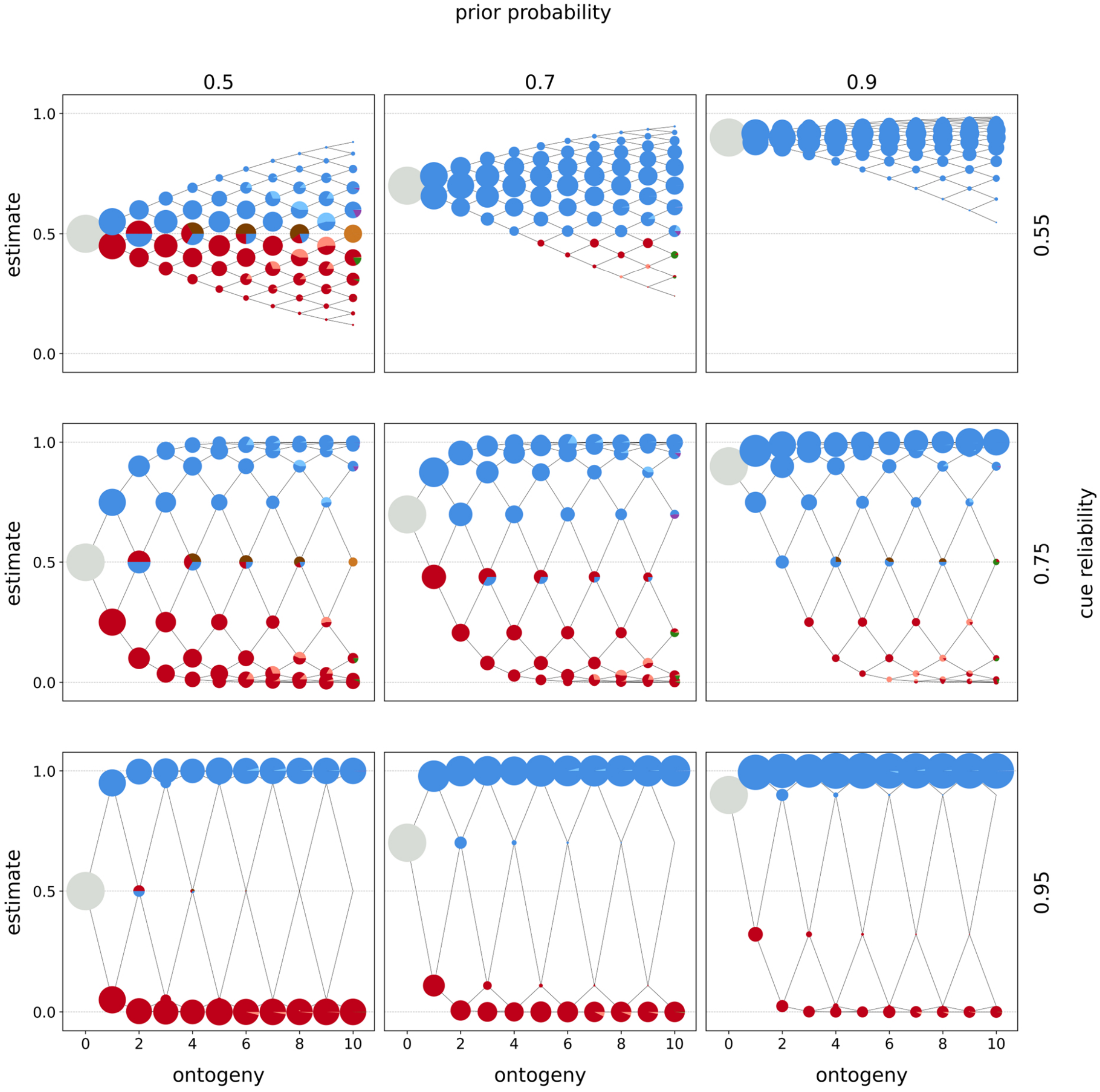

Figure S3.8: Optimal policies. Optimal policies are shown for a model with complete deconstruction and linear rewards and penalties. Columns indicate the prior estimate of being in E_1 and rows indicate the cue reliability. Within each panel, the horizontal axis denotes ontogeny and the vertical axis the posterior estimates of being in E_1. The entire population starts ontogeny with zero cues sampled and the prior estimate indicated by the column (indicated by the grey circle). In each time period organisms sample a cue (either C_0 or C_1), update their estimate, and make a phenotypic decision (colored circles). Black lines indicate developmental trajectories through this decision space, with lines branching upwards indicating the sampling of C_1 and lines branching downwards indicating the sampling of C_o. Colors denote the optimal, fitness-maximizing phenotypic choice in each state. Pies indicate cases in which organisms with the same posterior estimates make different phenotypic decisions. The area of a circle (pie piece) is proportional the probability of reaching that particular state. Colors indicate the following phenotypic decisions: Black corresponds to waiting, red to constructing P_0, blue to constructing P_1, purple to deconstructing P_0, green to deconstructing P_1, light red to a tie between constructing P_0 and deconstructing P_1, light blue to a tie between constructing P_1 and deconstructing P_0, brown to a tie between constructing either phenotypic

target, yellow to a tie between deconstructing either target, grey to a tie between construction and waiting, dark grey to a tie between deconstruction and waiting, and lastly ochre to a tie between all options.

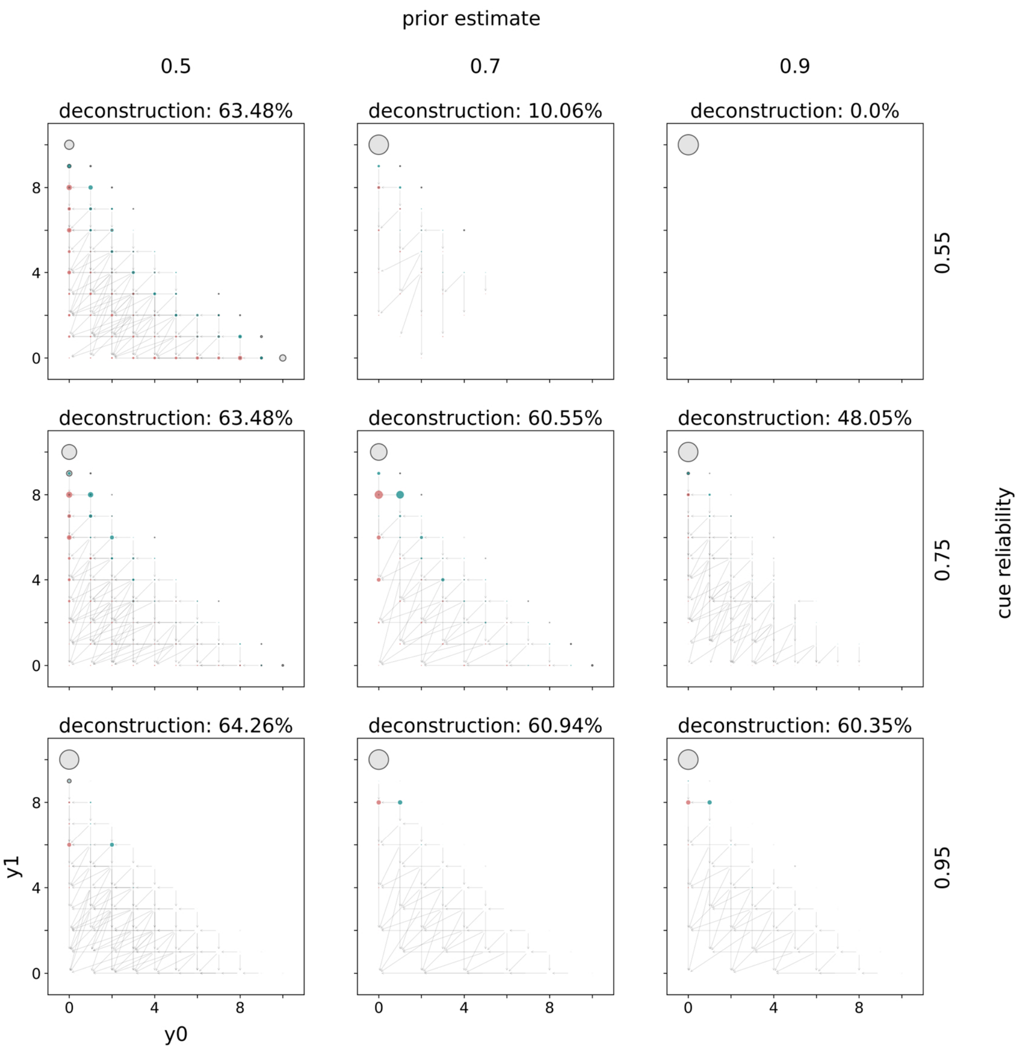

Figure S3.9: Distributions of mature phenotypes. Distributions of mature phenotypes are shown for a model with incremental deconstruction and linear rewards and penalties. Columns indicate the prior estimate of being in E_1 and rows indicate the cue reliability. The populations of mature phenotypes have been simulated in E_1. The title of each panel indicates the percentage of mature phenotypes that have deconstructed at some point during ontogeny. Within each panel the horizontal axis indicates the number of specializations towards E_0 and the vertical axis towards E_1. The lower triangle indicates how much mature phenotypes have constructed (teal circles) and what their phenotype looked like after deconstruction (red circles). Grey arrows connect phenotypes before (teal) and after (red) deconstruction. Grey circles with a black outline belong to mature phenotypes that never deconstructed. The area of a circle is proportional to the number mature organisms with this phenotype. The upper triangle indicates waiting. For each mature phenotype (after deconstruction) below the diagonal the corresponding square above the diagonal highlights the amount of waiting. The color intensity is proportional to the amount of waiting. Black squares indicate phenotypes that waited all of ontogeny (i.e. 20 time periods) and white squares phenotypes that never waited.

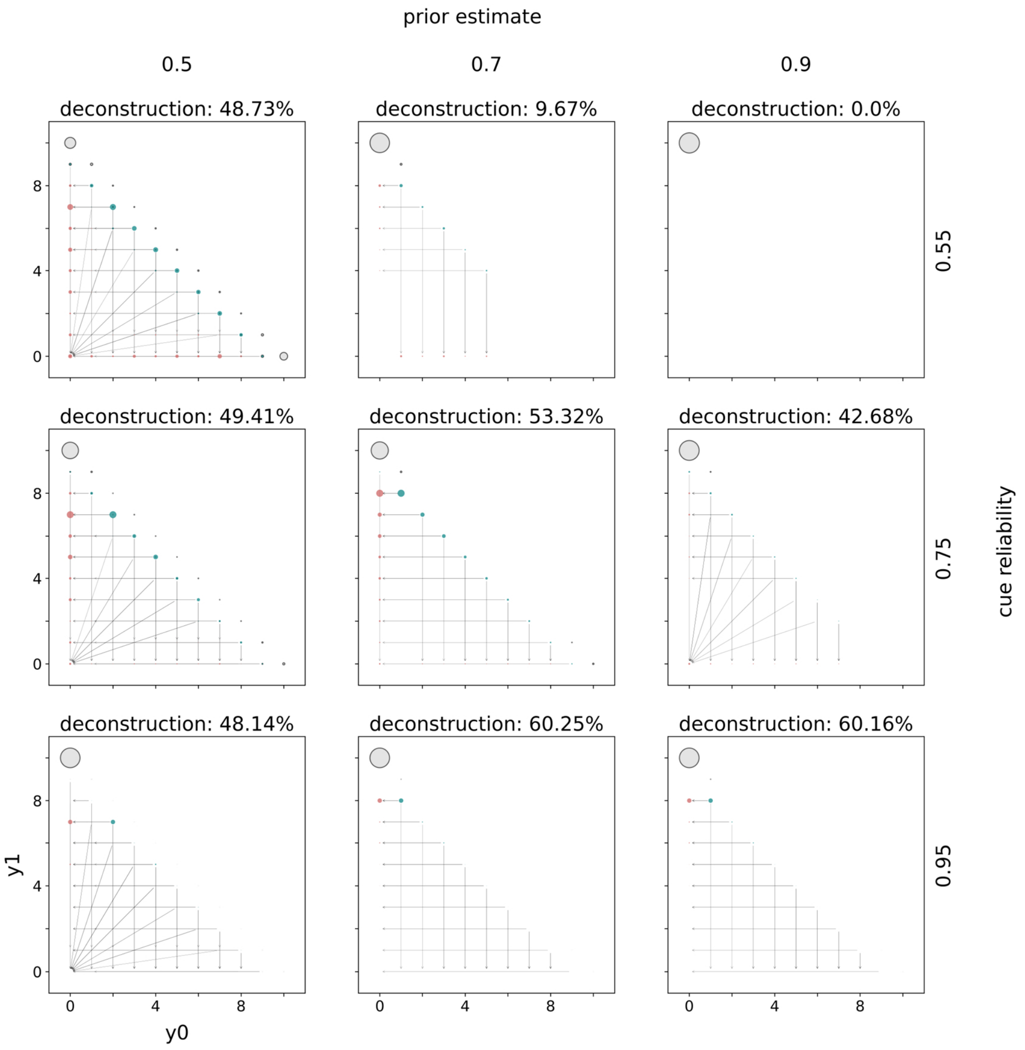

Figure S3.10: Distributions of mature phenotypes. Distributions of mature phenotypes are shown for a model with complete deconstruction and linear rewards and penalties. Columns indicate the prior estimate of being in E_1 and rows indicate the cue reliability. The populations of mature phenotypes have been simulated in E_1. The title of each panel indicates the percentage of mature phenotypes that have deconstructed at some point during ontogeny. Within each panel the horizontal axis indicates the number of specializations towards E_0 and the vertical axis towards E_1. The lower triangle indicates how much mature phenotypes have constructed (teal circles) and what their phenotype looked like after deconstruction (red circles). Grey arrows connect phenotypes before (teal) and after (red) deconstruction. Grey circles with a black outline belong to mature phenotypes that never deconstructed. The area of a circle is proportional to the number mature organisms with this phenotype. The upper triangle indicates waiting. For each mature phenotype (after deconstruction) below the diagonal the corresponding square above the diagonal highlights the amount of waiting. The color intensity is proportional to the amount of waiting. Black squares indicate phenotypes that waited all of ontogeny (i.e. 20 time periods) and white squares phenotypes that never waited.

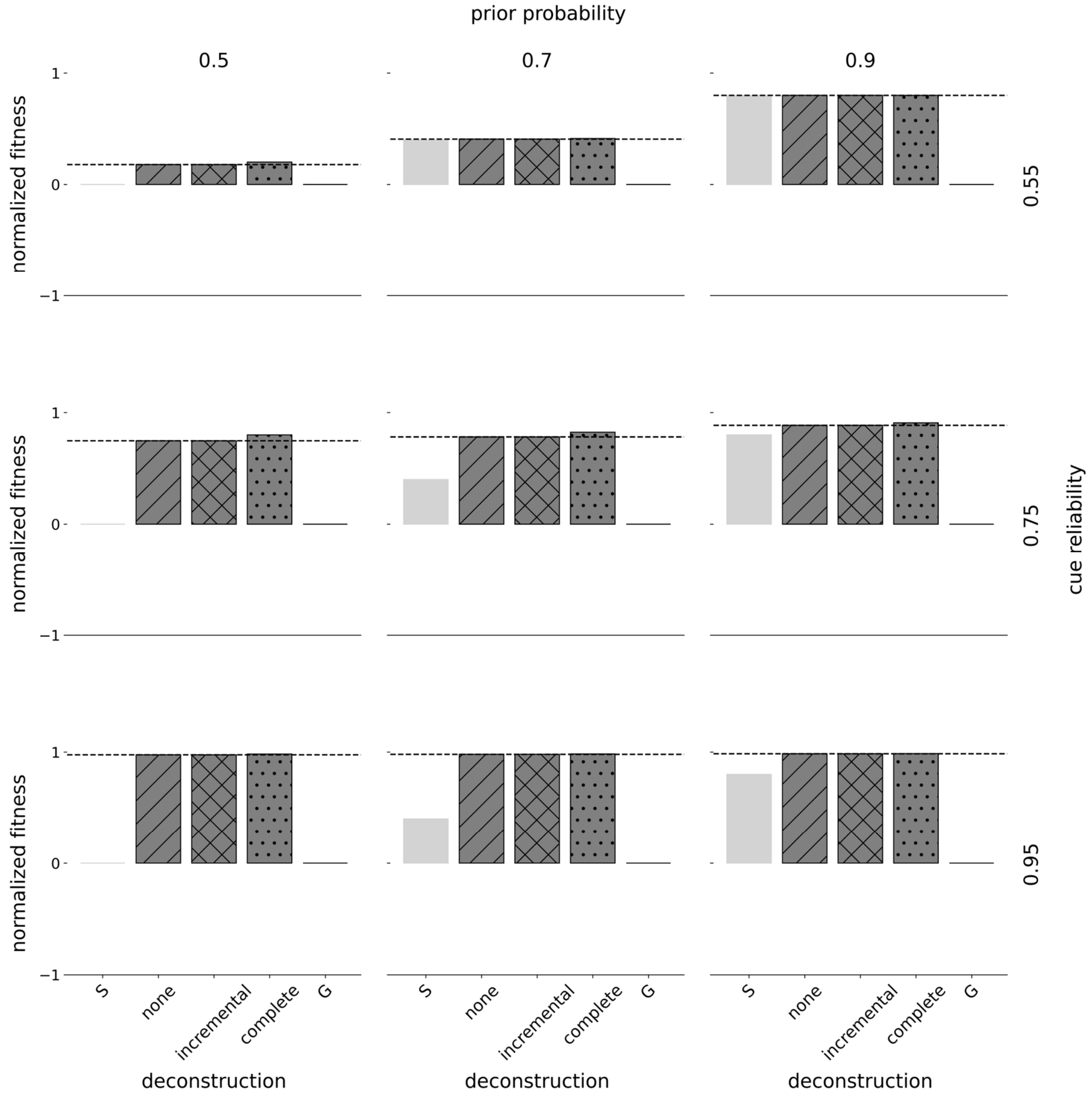

Figure S3.11: Normalized fitness. Normalized fitness is shown for linear rewards and linear penalties. Columns indicate the prior estimate of being in E_1 and rows indicate the cue reliability. Within each panel, the horizontal axis denotes the type of strategy where 'S' corresponds to a pure specialist strategy, 'O' to an optimal policy, and 'G' to a pure generalist strategy. The horizontal axis denotes fitness differences from baseline (corresponding to 0), normalized to range between -1 and 1. We show fitness of three different optimal policies: without deconstruction ('none'), with incremental deconstruction ('incremental'), and complete deconstruction ('complete'). Specialists always fully specialize according to the prior distribution. When priors are uninformative (0.5), half the population fully specializes towards P_0 and the other one towards P_1. Generalists always specialize halfway towards either phenotypic target.

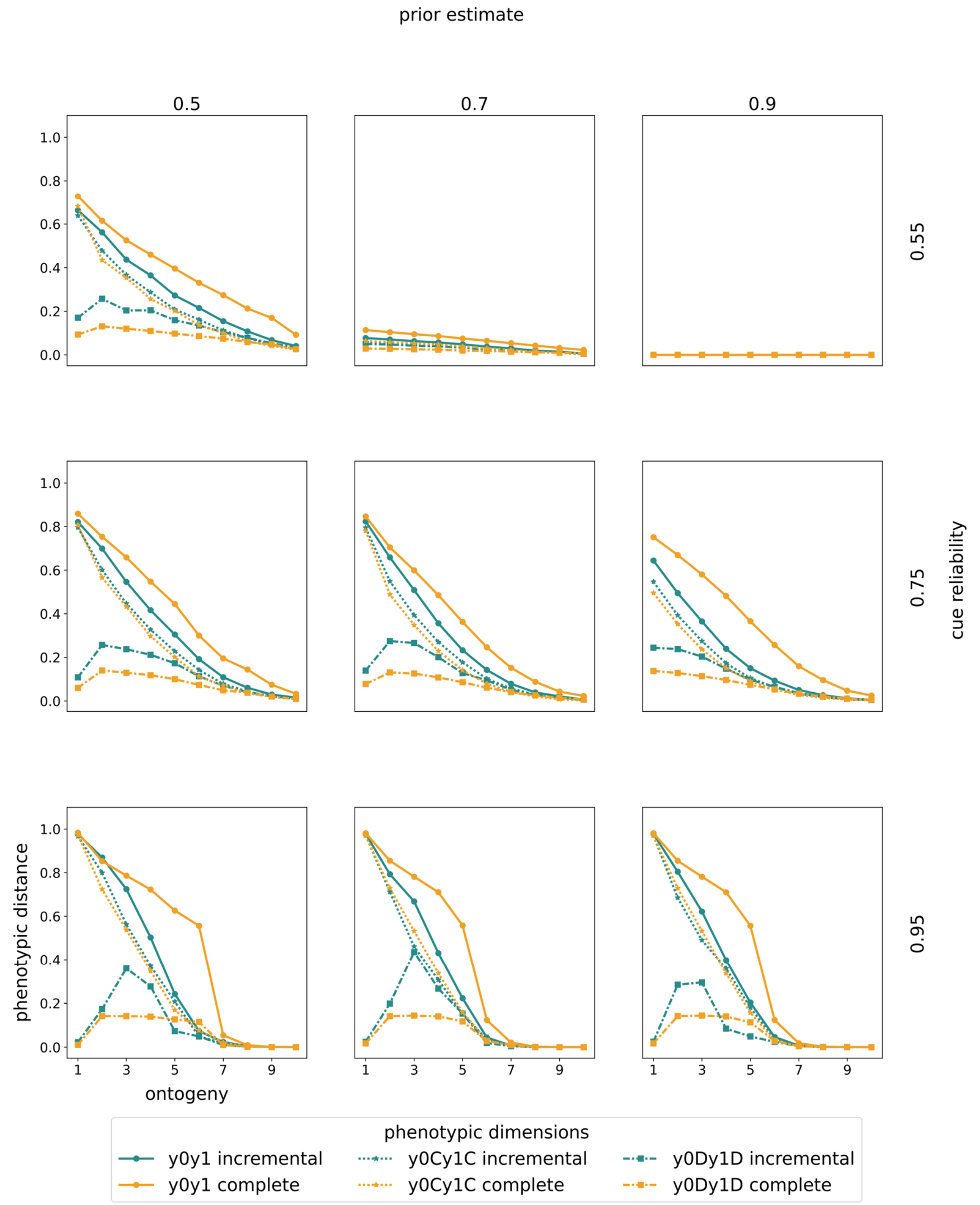

Figure S3.12: Changes in plasticity. Changes in plasticity are shown for linear rewards and linear penalties. Columns indicate the prior estimate of being in E_1 and rows indicate the cue reliability. Within each panel, the horizontal axis denotes ontogeny. The vertical axis denotes the normalized average phenotypic distance (our plasticity measure) across 10.000 pairs of simulated clones measured at the end of ontogeny. A specific point on any of the curves corresponds to a simulation experiment in which clones have been separated at the time point indicated by the horizontal axis. We show results from a model with incremental deconstruction in teal and with complete deconstruction in yellow. For each mode of deconstruction, we present three types of plasticity curves. First, we show plasticity in construction (dotted lines and stars) as the Euclidean distance between the number of time points spent constructing. Second, we show plasticity in deconstruction (dash-dotted lines and squares) as the Euclidean distance between the number of time points spent deconstruction. Third, we show total plasticity (solid lines and circles) as the Euclidean

distance between the number of specialization steps towards either target (after accounting for deconstruction). We normalize phenotypic distance in construction and total phenotypes by dividing by the maximally possible Euclidean distance, corresponding to $2 * \sqrt{T = 10}$. The normalization constant for deconstruction is $2 * \sqrt{T/2 = 5}$.

ESM 4 - Different paradigms for quantifying plasticity (linear rewards and penalties)

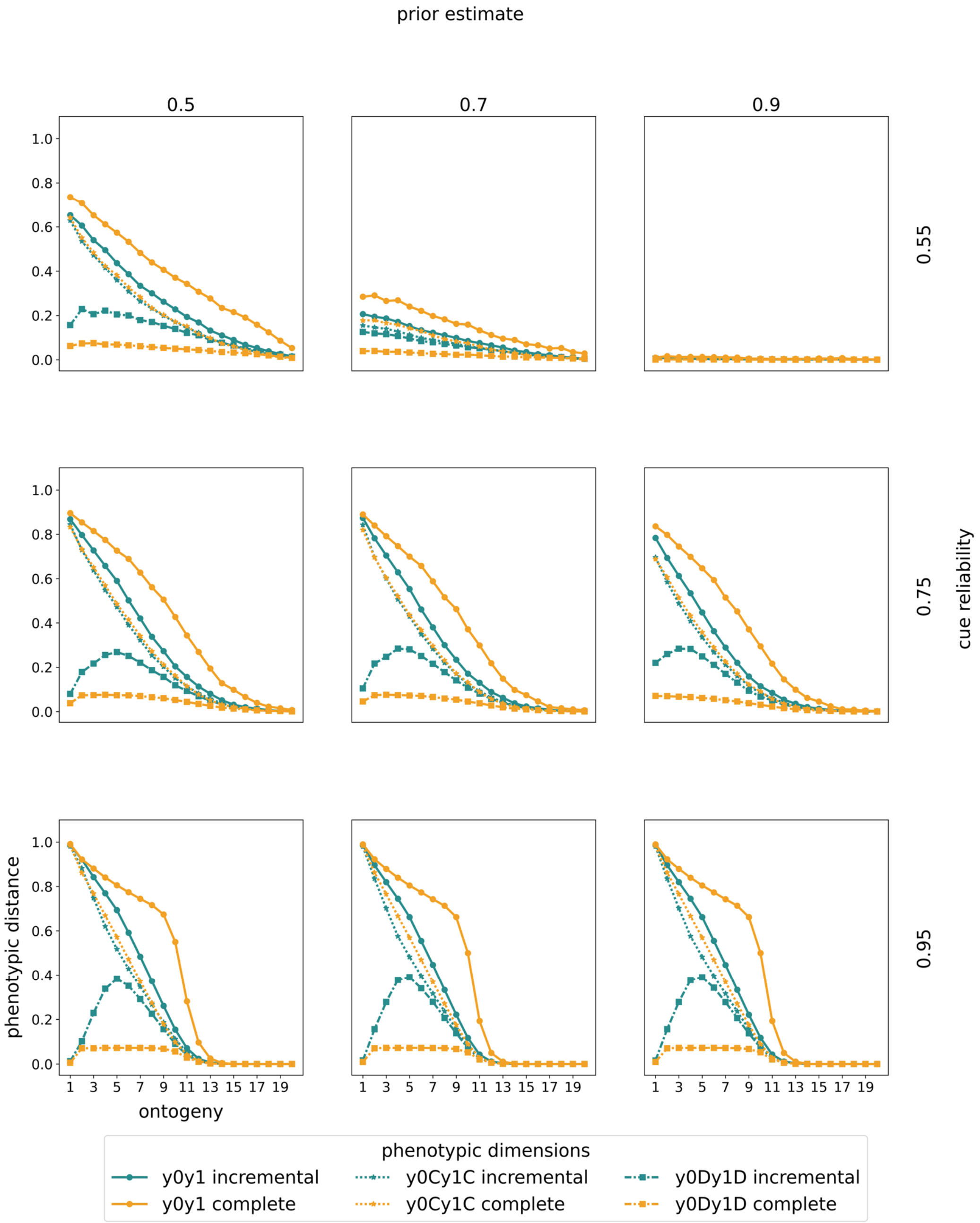

Figure S4.1: Changes in plasticity. Changes in plasticity are shown for linear rewards and linear penalties when clones receive reciprocal, opposite cues. Columns indicate the prior estimate of being in E_1 and rows indicate the cue reliability. Within each panel, the horizontal axis denotes ontogeny. The vertical axis denotes the normalized average phenotypic distance (our plasticity measure) across 10.000 pairs of simulated clones measured at the end of ontogeny. A specific point on any of the curves corresponds to a

simulation experiment in which clones have been separated at the time point indicated by the horizontal axis. We show results from a model with incremental deconstruction in teal and with complete deconstruction in yellow. For each mode of deconstruction, we present three types of plasticity curves. First, we show plasticity in construction (dotted lines and stars) as the Euclidean distance between the number of time points spent constructing. Second, we show plasticity in deconstruction (dash-dotted lines and squares) as the Euclidean distance between the number of time points spent deconstruction. Third, we show total plasticity (solid lines and circles) as the Euclidean distance between the number of specialization steps towards either target (after accounting for deconstruction). We normalize phenotypic distance in construction and total phenotypes by dividing by the maximally possible Euclidean distance, corresponding to $2 * \sqrt{T = 20}$. The normalization constant for deconstruction is $2 * \sqrt{T/2 = 10}$.

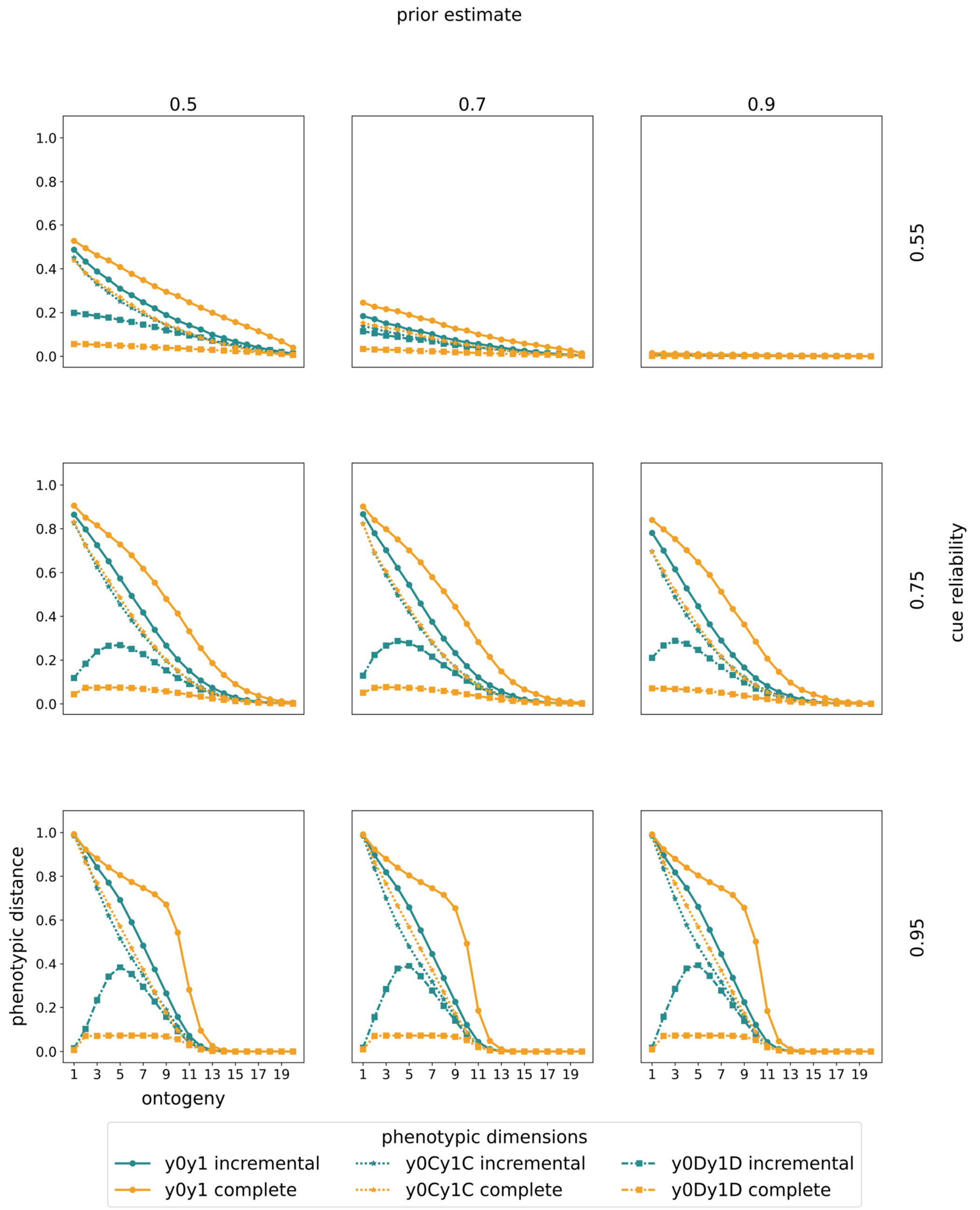

Figure S4.2: Changes in plasticity. Changes in plasticity are shown for linear rewards and linear penalties when clones receive cues from the opposite patch. Columns indicate the prior estimate of being in E_1 and rows indicate the cue reliability. Within each panel, the horizontal axis denotes ontogeny. The vertical axis denotes the normalized average phenotypic distance (our plasticity measure) across 10.000 pairs of simulated clones measured at the end of ontogeny. A specific point on any of the curves corresponds to a simulation experiment in which clones have been separated at the time point indicated by the horizontal axis. We show results from a model with incremental deconstruction in teal and with complete deconstruction in yellow. For each mode of deconstruction, we present three types of plasticity curves. First, we show plasticity in construction (dotted lines and stars) as the Euclidean distance between the number of time points spent constructing. Second, we show plasticity in deconstruction (dash-dotted lines and squares) as the Euclidean distance between the number of time points spent deconstruction. Third, we

show total plasticity (solid lines and circles) as the Euclidean distance between the number of specialization steps towards either target (after accounting for deconstruction). We normalize phenotypic distance in construction and total phenotypes by dividing by the maximally possible Euclidean distance, corresponding to $2 * \sqrt{T = 20}$. The normalization constant for deconstruction is $2 * \sqrt{T/2 = 10}$.

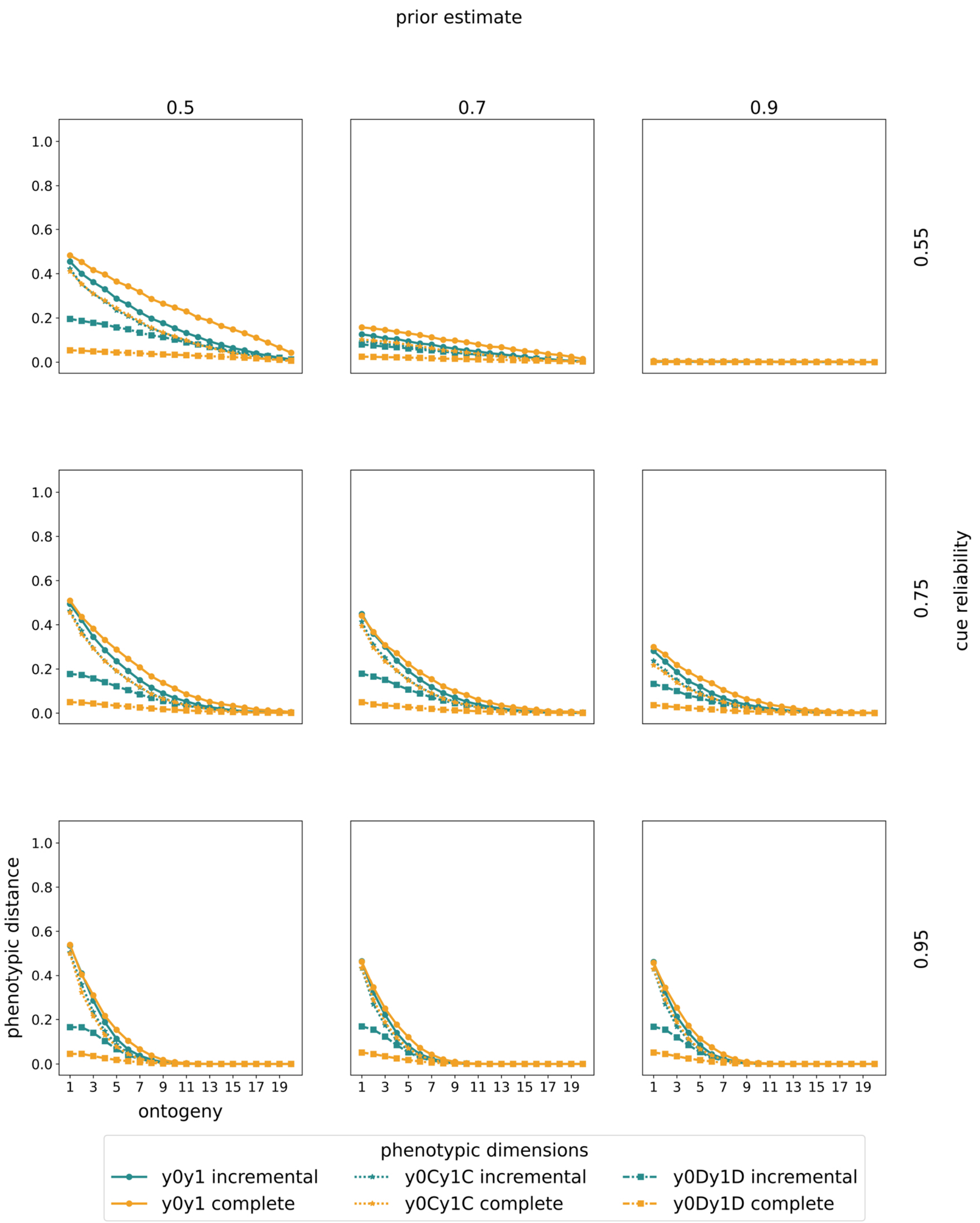

Figure S4.3: Changes in plasticity. Changes in plasticity are shown for linear rewards and linear penalties when clones receive uninformative cues ('deprivation'). Columns indicate the prior estimate of being in E_1 and rows indicate the cue reliability. Within each panel, the horizontal axis denotes ontogeny. The vertical axis denotes the normalized average phenotypic distance (our plasticity measure) across 10.000 pairs of

simulated clones measured at the end of ontogeny. A specific point on any of the curves corresponds to a simulation experiment in which clones have been separated at the time point indicated by the horizontal axis. We show results from a model with incremental deconstruction in teal and with complete deconstruction in yellow. For each mode of deconstruction, we present three types of plasticity curves. First, we show plasticity in construction (dotted lines and stars) as the Euclidean distance between the number of time points spent constructing. Second, we show plasticity in deconstruction (dash-dotted lines and squares) as the Euclidean distance between the number of time points spent deconstruction. Third, we show total plasticity (solid lines and circles) as the Euclidean distance between the number of specialization steps towards either target (after accounting for deconstruction). We normalize phenotypic distance in construction and total phenotypes by dividing by the maximally possible Euclidean distance, corresponding to $2 * \sqrt{T = 20}$. The normalization constant for deconstruction is $2 * \sqrt{T/2 = 10}$.

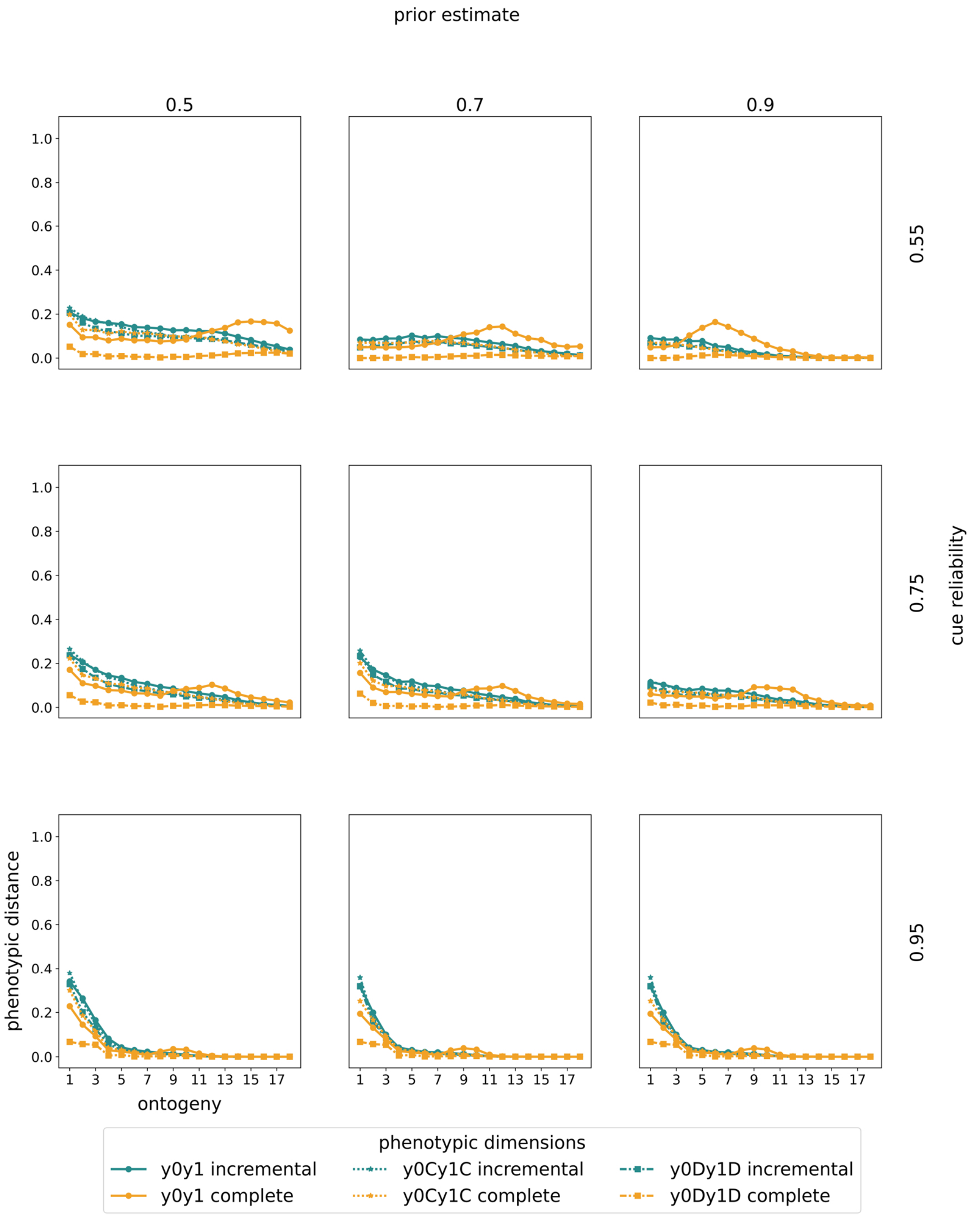

Figure S4.4: Changes in plasticity. Changes in plasticity are shown for linear rewards and linear penalties when clones receive reciprocal, opposite cues during a temporary separation period of 3 time periods. Phenotypic differences are measured at the end of ontogeny. Columns indicate the prior estimate of being in E_1 and rows indicate the cue reliability. Within each panel, the horizontal axis denotes ontogeny. The vertical axis denotes the normalized average phenotypic distance (our plasticity measure) across 10.000 pairs of simulated clones measured at the end of ontogeny. A specific point on any of the curves corresponds to a simulation experiment in which clones have been separated at the time point indicated by the horizontal axis. We show results from a model with incremental deconstruction in teal and with complete deconstruction in yellow. For each mode of deconstruction, we present three types of plasticity curves. First, we show plasticity in construction (dotted lines and stars) as the Euclidean distance between the number of time points spent constructing. Second, we show plasticity in deconstruction (dash-dotted

lines and squares) as the Euclidean distance between the number of time points spent deconstruction. Third, we show total plasticity (solid lines and circles) as the Euclidean distance between the number of specialization steps towards either target (after accounting for deconstruction). We normalize phenotypic distance in construction and total phenotypes by dividing by the maximally possible Euclidean distance, corresponding to $2 * \sqrt{T = 20}$. The normalization constant for deconstruction is $2 * \sqrt{T/2 = 10}$.

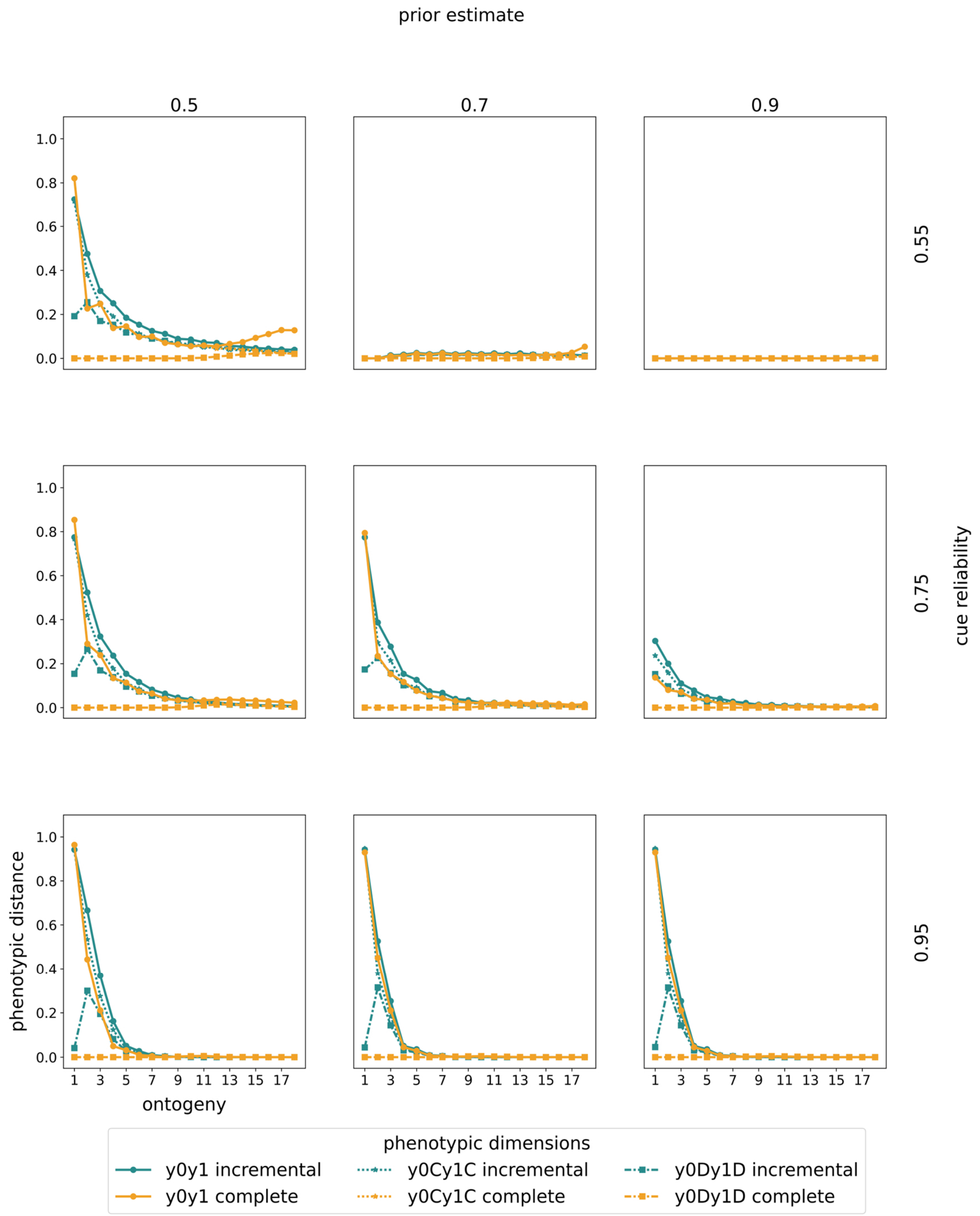

Figure S4.5: Changes in plasticity. Changes in plasticity are shown for linear rewards and linear penalties when clones receive reciprocal, opposite cues during a temporary separation period of three time periods. Phenotypic differences are measured right after the separation. Columns indicate the prior estimate of

being in E_1 and rows indicate the cue reliability. Within each panel, the horizontal axis denotes ontogeny. The vertical axis denotes the normalized average phenotypic distance (our plasticity measure) across 10.000 pairs of simulated clones measured at the end of ontogeny. A specific point on any of the curves corresponds to a simulation experiment in which clones have been separated at the time point indicated by the horizontal axis. We show results from a model with incremental deconstruction in teal and with complete deconstruction in yellow. For each mode of deconstruction, we present three types of plasticity curves. First, we show plasticity in construction (dotted lines and stars) as the Euclidean distance between the number of time points spent constructing. Second, we show plasticity in deconstruction (dash-dotted lines and squares) as the Euclidean distance between the number of time points spent deconstruction. Third, we show total plasticity (solid lines and circles) as the Euclidean distance between the number of specialization steps towards either target (after accounting for deconstruction). We normalize phenotypic distance in construction and total phenotypes by dividing by the maximally possible Euclidean distance, corresponding to $2 * \sqrt{time\ period\ of\ separation + 3}$. The normalization constant for deconstruction is $2 * \sqrt{time\ period\ of\ separation + 3}$.
